# Difluorination of α-(bromomethyl)styrenes *via* I(I)/I(III) catalysis: facile access to electrophilic linchpins for drug discovery†

**DOI:** 10.1039/d1sc01132d

**Published:** 2021-03-26

**Authors:** Joel Häfliger, Keith Livingstone, Constantin G. Daniliuc, Ryan Gilmour

**Affiliations:** Organisch-Chemisches Institut, Westfälische Wilhelms-Universität Münster Corrensstraße 36 48149 Münster Germany ryan.gilmour@uni-muenster.de

## Abstract

Simple α-(bromomethyl)styrenes can be processed to a variety of 1,1-difluorinated electrophilic building blocks *via* I(I)/I(III) catalysis. This inexpensive main group catalysis strategy employs *p*-TolI as an effective organocatalyst when combined with Selectfluor® and simple amine·HF complexes. Modulating Brønsted acidity enables simultaneous *geminal* and *vicinal* difluorination to occur, thereby providing a platform to generate multiply fluorinated scaffolds for further downstream derivatization. The method facilitates access to a tetrafluorinated API candidate for the treatment of amyotrophic lateral sclerosis. Preliminary validation of an enantioselective process is disclosed to access α-phenyl-β-difluoro-γ-bromo/chloro esters.

## Abstract 2

Simple α-(bromomethyl)styrenes can be processed to a variety of 1,1-difluorinated electrophilic building blocks *via* I(I)/I(III) catalysis.

## Graphical abstract

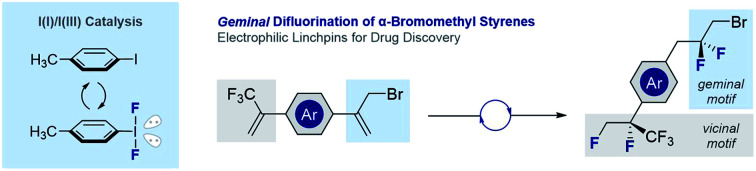

Structural editing with fluorine enables geometric and electronic variation to be explored in functional small molecules whilst mitigating steric drawbacks.^1^ This expansive approach to manipulate structure–function interplay continues to manifest itself in bio-organic and medicinal chemistry.^2^ Of the plenum of fluorinated motifs commonly employed, the *geminal* difluoromethylene group^3^ has a venerable history.^4^ This is grounded in the structural as well as electronic ramifications of CH_2_ → CF_2_ substitution, as is evident from a comparison of propane and 2,2-difluoropropane (Fig. 1, upper). Salient features include localized charge inversion (C–H^*δ*+^ to C–F^*δ*−^) and a widening of the internal angle from 112° to 115.4°.^5^ Consequently, *geminal* difluoromethylene groups feature prominently in the drug discovery repertoire^6^ to mitigate oxidation and modulate physicochemical parameters. Catalysis-based routes to generate electrophilic linchpins that contain the *geminal* difluoromethylene unit have thus been intensively pursued, particularly in the realm of main group catalysis.^7–9^ Motivated by the potential of this motif in contemporary medicinal chemistry, it was envisaged that an I(I)/I(III) catalysis platform could be leveraged to convert simple α-(bromomethyl)styrenes to *gem*-difluorinated linchpins: the primary C(sp^3^)–Br motif would facilitate downstream synthetic manipulations (Fig. 1, lower). To that end, *p*-TolI would function as a catalyst to generate *p*-TolIF_2_*in situ* in the presence of an external oxidant^10^ and an amine·HF complex. Alkene activation (**I**) with subsequent bromonium ion formation (**II**)^11^ would provide a pre-text for the first C–F bond forming process (**III**) with regeneration of the catalyst. A subsequent phenonium ion rearrangement^12^/fluorination sequence (**III** and **IV**) would furnish the *geminal* difluoromethylene group and liberate the desired electrophilic building block.

**Fig. 1:**
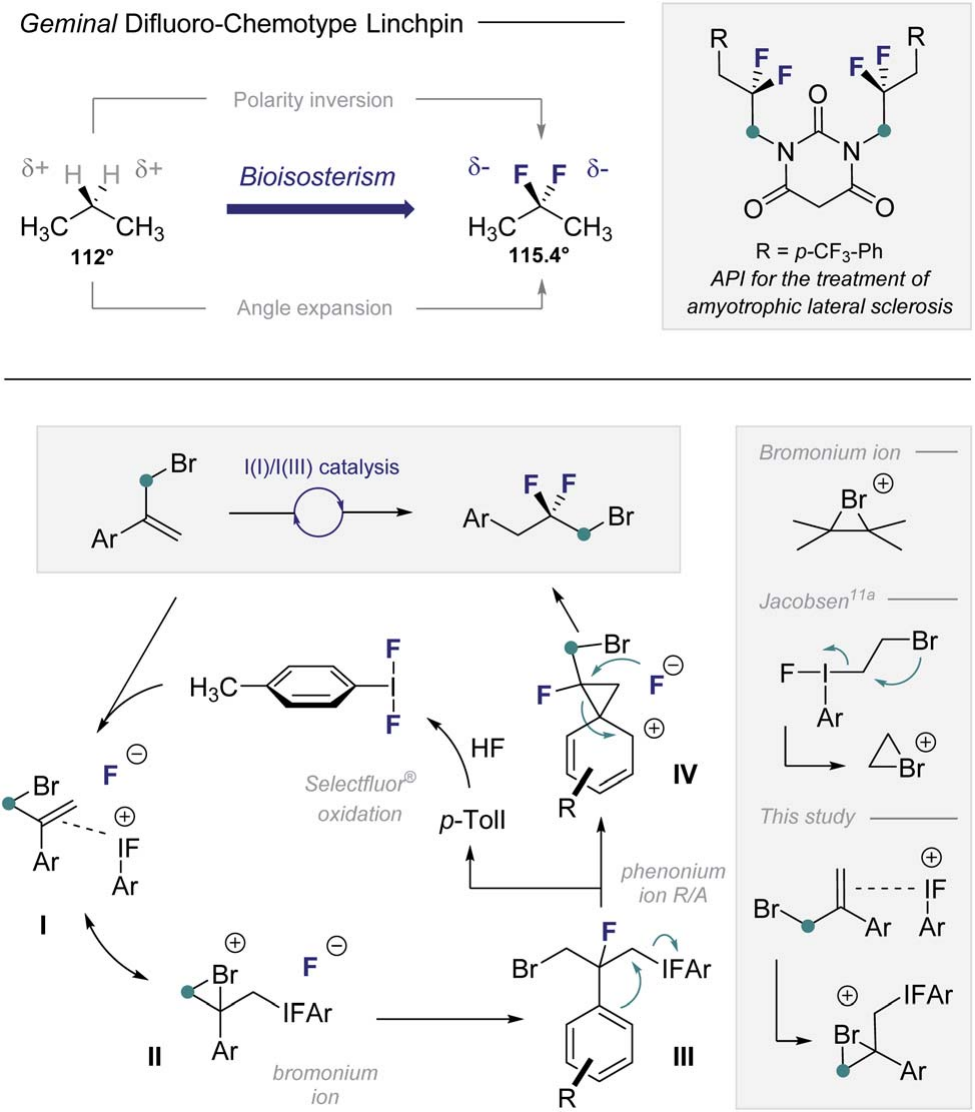
The *geminal* difluoromethylene group: bioisosterism, and catalysis-based access from α-(bromomethyl)styrenes *via* I(I)/I(III) catalysis.

To validate this conceptual framework, a short process of reaction optimization (**1a** → **2a**) was conducted to assess the influence of solvent, amine·HF ratio (Brønsted acidity)^13^ and catalyst loading (Table 1). Initial reactions were performed with *p*-TolI (20 mol%), Selectfluor® (1.5 equiv.) as an oxidant, and CHCl_3_ as the reaction medium. Variation of the amine : HF ratio was conducted to explore the influence of Brønsted acidity on catalysis efficiency (entries 1–4). An optimal ratio of 1 : 6 was observed enabling the product **2a** to be generated in >95% NMR-yield. Although reducing the catalyst loading to 10 and 5 mol% (entries 5 and 6, respectively) led to high levels of efficiency (79% yield with 5 mol%), the remainder of the study was performed with 20 mol% *p*-TolI. Notably, catalytic *vicinal* difluorination was not observed at any point during this optimization, in contrast with previous studies from our laboratory.^9*d*,*i*^ A solvent screen revealed the importance of chlorinated solvents (entries 7 and 8): in contrast, performing the reaction in ethyl trifluoroacetate (ETFA) and acetonitrile resulted in a reduction in yield (9 and 10). Finally, a control reaction in the absence of *p*-TolI confirmed that an I(I)/I(III) manifold was operational (entry 11). An expanded optimization table is provided in the ESI.†

**Table 1:** Reaction optimization^a^

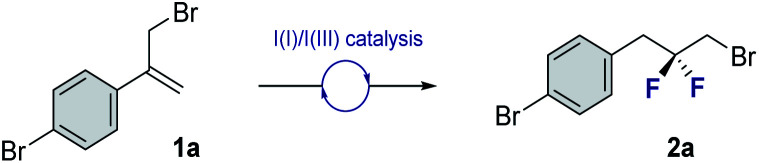				
Entry	Solvent	Amine/HF	Catalyst loading [mol%]	Yield^b^ [%]
1	CHCl_3_	1 : 4.5	20	72
**2**	**CHCl**_**3**_	**1 :** **6.0**	**20**	**>95**
3	CHCl_3_	1 : 7.5	20	94
4	CHCl_3_	1 : 9.23	20	87
5	CHCl_3_	1 : 6.0	10	87
6	CHCl_3_	1 : 6.0	5	79
7	DCM	1 : 6.0	20	>95
8	DCE	1 : 6.0	20	93
9	ETFA	1 : 6.0	20	84
10	MeCN	1 : 6.0	20	50
11	CHCl_3_	1 : 6.0	0	<5

a Standard reaction conditions: **1a** (0.2 mmol), Selectfluor® (1.5 equiv.), amine : HF source (0.5 mL), solvent (0.5 mL), *p*-TolI, 24 h, rt.

b Determined by ^19^F NMR using α,α,α-trifluorotoluene as internal standard.

To explore the scope of this *geminal* difluorination, a series of α-(bromomethyl)styrenes were exposed to the standard reaction conditions (Fig. 2). Gratifyingly, product **2a** could be isolated in 80% yield after column chromatography on silica gel. The parent α-(bromomethyl)styrene was smoothly converted to species **2b**, as were the *p*-halogenated systems that furnished **2c** and **2d** (71 and 79%, respectively). The regioisomeric bromides **2e** and **2f** (70 and 62%, respectively) were also prepared for completeness to furnish a series of linchpins that can be functionalized at both termini by displacement and cross-coupling protocols (**2a**, **2e** and **2f**). Modifying the amine : HF ratio to 1 : 4.5 provided conditions to generate the ^*t*^Bu derivative **2g** in 68% yield.^14^ Electron deficient aryl derivatives were well tolerated as is demonstrated by the formation of compounds **2h–2k** (up to 91%). Disubstitution patterns (**2l**, 81%), sulfonamides (**2m**, 75%) and phthalimides (**2n**, 80%) were also compatible with the standard catalysis conditions. Gratifyingly, compound **2n** was crystalline and it was possible to unequivocally establish the structure by X-ray crystallography (Fig. 2, lower).^15^ The C9–C8–C7 angle was measured to be 112.6° (*cf.* 115.4° for 2,2-difluoropropane).^5^ Intriguingly, the C(sp^3^)–Br bond eclipses the two C–F bonds rather than adopting a conformation in which dipole minimization is satisfied (F1–C8–C9–Br dihedral angle is 56.3°).

**Fig. 2:**
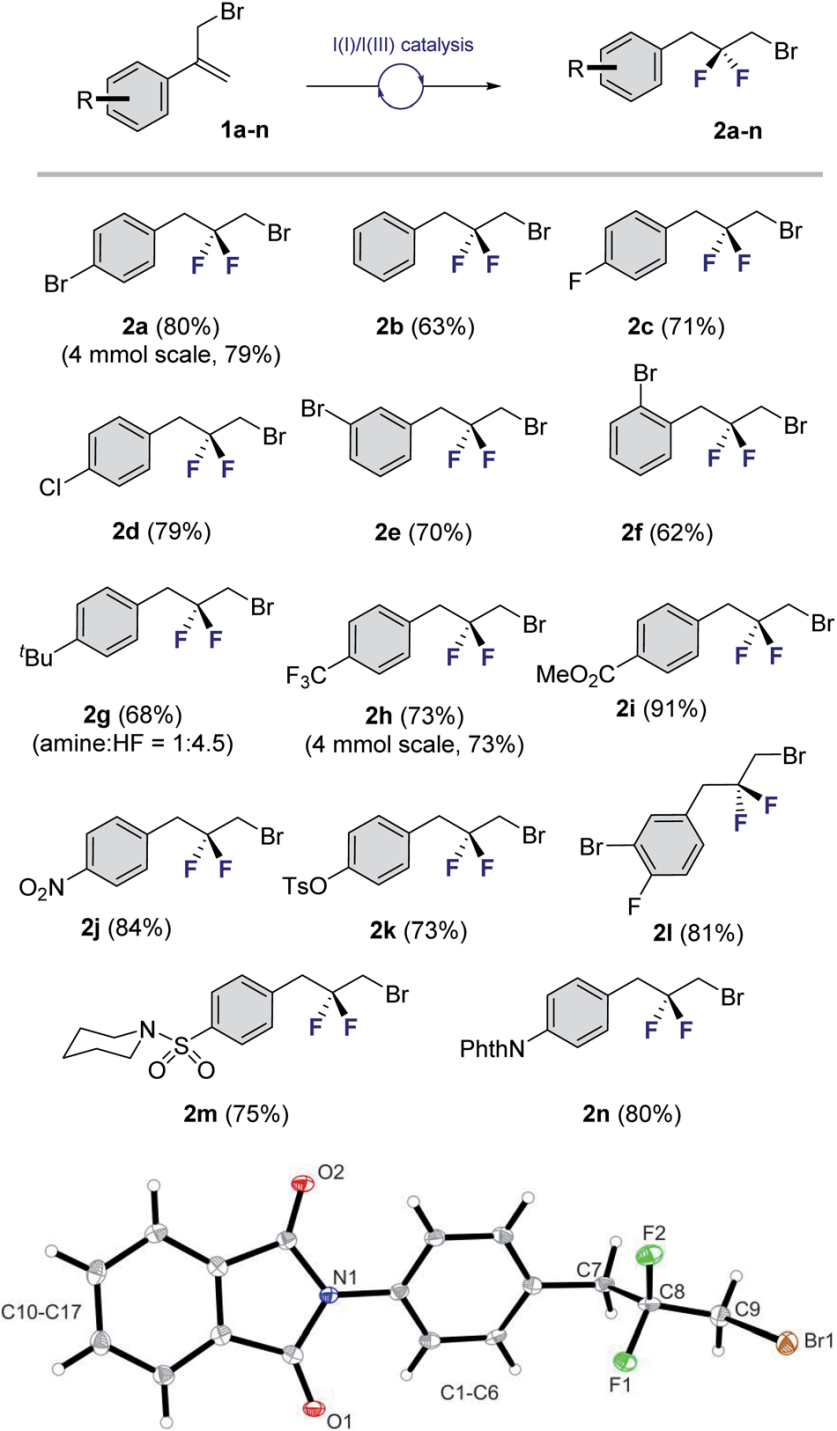
Exploring the scope of the *geminal* difluorinative rearrangement of α-(bromomethyl)styrenes *via* I(I)/I(III) catalysis. Isolated yields after column chromatography on silica gel are reported. X-ray crystal structure of compound **2n** (CCDC 2055892†). Thermal ellipsoids shown at 50% probability.

Cognizant of the influence of Brønsted acidity on the regioselectivity of I(I)/I(III) catalyzed alkene difluorination,^9*d*^ the influence of the amine : HF ratio on the fluorination of electronically non-equivalent divinylbenzene derivatives was explored (Fig. 3, top). Initially, compound **3** bearing an α-(trifluoromethyl)styrene motif was exposed to the standard catalysis conditions with a 1 : 4.5 amine : HF ratio. Exclusive, chemoselective formation of **4** was observed in 79% yield. Simple alteration of the amine : HF ratio to 1 : 7.5 furnished the tetrafluorinated product **5** bearing both the *geminal* and *vicinal* difluoromethylene^16^ groups (55% yield. 20% of the *geminal*–*geminal* product was also isolated. See ESI†). Relocating the electron-withdrawing group (α-CF_3_ → β-CO_2_Me) and repeating the reaction with 1 : 4.5 amine : HF generated the *geminal* CF_2_ species **7** in analogy to compound **4**. However, increasing the amine : HF ratio to 1 : 6.0 led exclusively to double *geminal* difluorination (**8**, 55%).

**Fig. 3:**
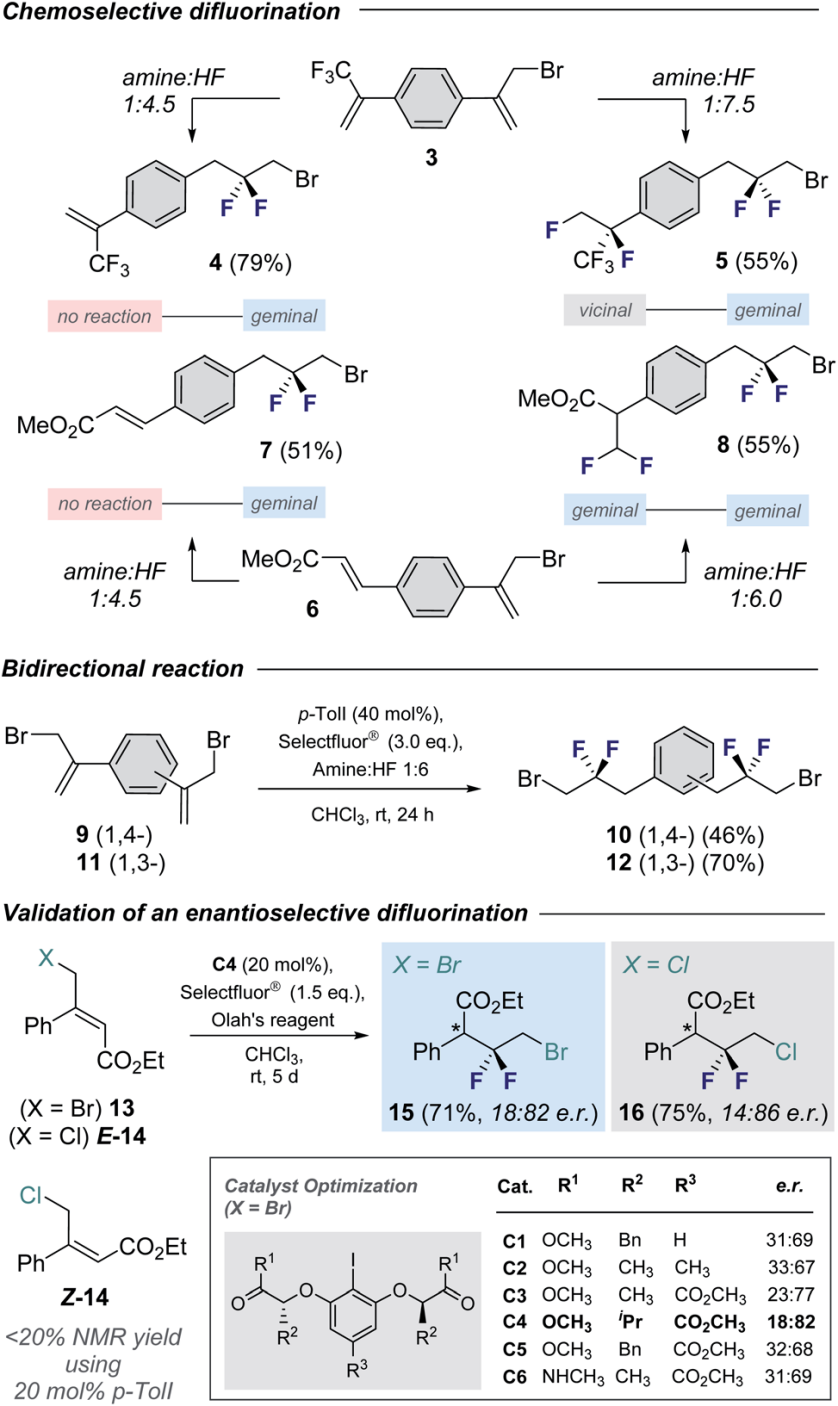
Exploring the synthetic versatility of this platform. (Top) Leveraging Brønsted acidity to achieve chemoselective fluorination. (Centre) Bidirectional functionalization. (Bottom) Preliminary validation of an enantioselective variant.

Similarly, bidirectional *geminal* difluorination of the divinylbenzene derivatives **9** and **11** was efficient, enabling the synthesis of **10** (46%) and **12** (70%), respectively. This enables facile access to bis-electrophilic fluorinated linchpins for application in materials chemistry.

Preliminary validation of an enantioselective variant^8*d*^ was achieved using the trisubstituted alkene **13**. To that end, a series of *C*_2_-symmetric resorcinol-based catalysts were explored (see Fig. 3, inset). This enabled the generation of product **15** in up to 18 : 82 e.r. and 71% isolated yield. It is interesting to note that this catalysis system was also compatible with the chlorinated substrate **E-14**. A comparison of geometric isomers revealed a matched-mismatched scenario: whilst **E-14** was efficiently converted to **16** (75%, 14 : 86 e.r.), **Z-14** was recalcitrant to rearrangement (<20%).

To demonstrate the synthetic utility of the products, chemoselective functionalization of linchpin **2a** was performed to generate **17** (57%) and **18** (87%), respectively (Fig. 4). Finally, this method was leveraged to generate an API for amyotrophic lateral sclerosis. Whereas the reported synthesis^17^ requires the exposure of α-bromoketone **19** to neat DAST over 7 days,^18^ compound **2h** can be generated using this protocol over a more practical timeframe (24 h) on a 4 mmol scale. This key building block was then processed, *via* the amine hydrochloride salt **20**, to API **21**.

**Fig. 4:**
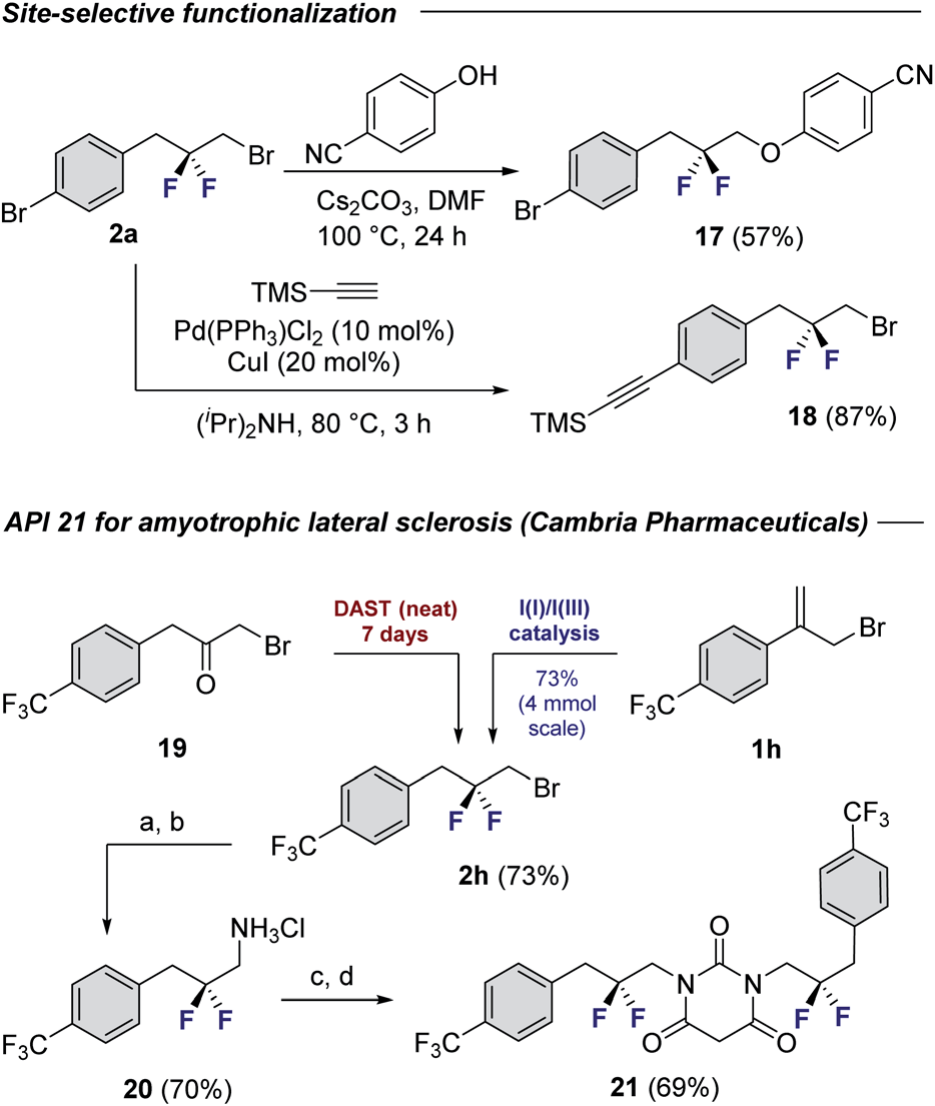
Selected modification of building blocks **2a** and **2h**. Conditions: (a) NaN_3_, DMF, 110 °C, 16 h. (b) Pd(OH)_2_/C (10 mol%), EtOH, 1 M HCl, rt, 24 h; (c) CDI, Et_3_N, THF, 60 °C, 16 h; (d) malonyl chloride, DCM, 0 °C, 2 h.

## Conclusions

In conclusion, an I(I)/I(III) catalysis manifold that facilitates the difluorinative rearrangement of α-(bromomethyl)styrenes is disclosed. In addition to generating electrophiles with a single *geminal* difluoro motif, bidirectional processes are presented together with simultaneous *geminal* and *vicinal* difluorination. Preliminary validation of an enantioselective reaction is demonstrated, to enable the generation of versatile α-phenyl-β-difluoro-γ-bromo/chloro esters. Finally, the transformation has been leveraged to enable the synthesis of an amyotrophic lateral sclerosis drug: this provides an operationally simple alternative to common deoxyfluorinating reagents when preparing *gem*-difluoro linchpins for contemporary medicinal chemistry.

## Author contributions

All authors have given approval to the final version of the manuscript.

## Conflicts of interest

There are no conflicts to declare.

## Note added after first publication

This article replaces the version published on 31st March 2021. The title contained a typesetting error. The oxidation state change in the title was incorrect and should read I(I)/I(III).

## Supplementary Material

SC-012-D1SC01132D-s001

SC-012-D1SC01132D-s002
