# Supplementary material for: Difluorination of α-(bromomethyl)styrenes via I(I)/I(III) catalysis: facile access to electrophilic linchpins for drug discovery
Source: Chem Sci. 2021 Mar 26;12(17):6148–52. doi: 10.1039/d1sc01132d (PMC8098697; doi:10.1039/d1sc01132d)
Supplement: SC-012-D1SC01132D-s001 [file SC-012-D1SC01132D-s001.pdf]

Electronic Supplementary Material (ESI) for Chemical Science.
This journal is © The Royal Society of Chemistry 2021

Supporting Information

Difluorination of α-(Bromomethyl)styrenes via I(I)/I(III) Catalysis: Facile Access to Electrophilic Linchpins for Drug Discovery

Joel Häfliger, Keith Livingstone, Constantin G. Daniliuc and Ryan Gilmour*

**E-mail: ryan.gilmour@uni-muenster.de*

1. General Information 2
2. Reaction Optimization – Extended Table 5
2.1. Racemic Reaction 5
2.2. Enantioselective Reaction 6
3. Synthetic Procedures 7
3.1. Preparation of the Catalysts 7
3.2. Acetophenones 13
3.3 α-(Methyl)styrenes 16
3.4 α-(Bromomethyl)styrenes 30
3.5 Preparation of the *gem*-Difluorinated Products 45
3.6 Functionalizations of the *geminal* Difluorinated Products 69
4. X-Ray Diffraction 75
5. NMR-Spectra 77
6. References 156

1. General Information

All reactions were carried out under anhydrous conditions using flame dried glassware and dry solvents under an argon atmosphere unless otherwise stated. Dichloromethane (DCM) and Tetrahydrofuran (THF) were dried by a Grubbs purification system including columns packed with molecular sieves and aluminium oxide and were stored, if necessary, over dried molecular sieves (4 Å). Chloroform was dried over molecular sieves (4 Å). Solvents used for extraction and purification were purchased as technical grade and were purified by distillation. Commercially available reagents were purchased from abcr, Alfa Aesar, Apollo Scientific, BLD Pharm, Fluorochem, Sigma Aldrich or TCI Europe and were used without further purification. Column chromatography was performed using silica gel (40-63 µm, VWR Chemicals). All the 1D-NMR spectra were acquired by a Bruker AV300, AV400, Agilent DD2 500 or an Agilent DD2 600. 2D-NMR spectra for full characterization of new compounds were measured by an Agilent DD2 500 or an Agilent DD2 600. All the NMR measurements were performed by the NMR service department of the Organisch-Chemisches Institut, Westfälische Wilhelms-Universität Münster. ^{19}F-NMR yields were determined using (trifluoromethyl)benzene as an internal standard. ^{1}H and ^{13}C NMR chemical shifts are reported in parts per million and are calibrated to the residual solvent signal ($CHCl_3$: ^{1}H NMR: δ [ppm] = 7.26, ^{13}C NMR: δ [ppm] = 77.16; DMSO-d6: ^{1}H NMR: δ [ppm] = 2.50, ^{13}C NMR: δ [ppm] = 39.5). The multiplicity of the signals is given as follows: s: singlet, d: doublet, t: triplet, q: quartet, p: quintet, h: septet, m: multiplet, app: apparent. The coupling constants are reported in Hertz [Hz]. Melting points were determined on a Büchi B-545 melting point apparatus. FT-IR data were collected using a Perkin-Elmer 100 FT-IR spectrometer. The absorption maxima were reported in [cm^{-1}] and the relative intensities of the absorption maxima are reported as follows: w (weak), m (medium), s (strong), br (broad). HRMS data was collected by the mass spectrometry service at the Organisch-Chemisches Institut, Westfälische Wilhelms-Universität Münster. GC-EI-MS data was collected using a Triplequad Quattro Micro GC, or a Qp5050 Single Quad. ESI-MS data was obtained using a Bruker Daltonics MicroTof. HPLC measurements for the determination of enantiomeric ratios were preformed using an Agilent Infinity 1260 system, equipped with a diode array detector (DAD) and a chiral OJ-H or AS-H column. The temperature of the chiral column was kept constant between 25 and 30 degrees. A mixture of *i*-PrOH and

hexanes was used as an eluent. The optical rotations were measured on a JASCO P2000 polarimeter.

Preparation Of Amine·HF Mixtures with Different Amine:HF Ratios

Amine·HF sources:

NEt_3·3HF; Supplier: abcr; (MW: 161.21g/mol, ρ = 0.990 g/mL)

Olah's Reagent (70wt% HF: Py·9.23HF); Supplier: Sigma Aldrich; (MW: 263.79 g/mol, ρ = 1.1 g/mL)

Procedure for Calculating Compositions of Amine·HF Mixtures:

Example Amine:HF ratio 1:6:

$$x \cdot 3\,HF + 9.23\,HF = (x+1) \cdot desired\,ratio = (x+1) \cdot 6\,HF$$
$$x = 1.077$$

Introducing amounts of HF sources:

$$\frac{q \cdot x \cdot MW_{NEt_3 \cdot 3HF}}{\rho_{NEt_3 \cdot 3HF}} + \frac{q \cdot MW_{Py \cdot 9.23HF}}{\rho_{Py \cdot 9.23HF}} = V_{tot,Amine:HF}$$

where

$$\frac{q \cdot x \cdot MW_{NEt_3 \cdot 3HF}}{\rho_{NEt_3 \cdot 3HF}} = V_{NEt_3 \cdot 3HF} \quad and \quad \frac{q \cdot MW_{Py \cdot 9.23HF}}{\rho_{Py \cdot 9.23HF}} = V_{Py \cdot 9.23HF}$$

For 1.25 mL of Amine·HF mixture, the calculation is as follows:

$$\frac{q \cdot 1.077 \cdot 161.21\,g/mol}{0.990\,g/mL} + \frac{q \cdot 263.79\,g/mol}{1.1\,g/mL} = 1.25\,mL$$
$$175.4\frac{mL}{mol} + 239.8\frac{mL}{mol} = \frac{1.25\,mL}{q}$$
$$\frac{1\,mol}{332.2} = q$$

Using q and x, the volumes for the components are calculated as follows:

$$0.53\,mL = V_{NEt_3 \cdot 3HF} \quad and \quad 0.72\,mL = V_{Py \cdot 9.23HF}$$

Using the procedure shown above, the compositions for the used Amine·HF mixtures were calculated as follows:

Table 1. Parameters for calculating volumetric compositions of Amine·HF mixtures with different Amine:HF ratios

Amine:HF ratio	Volume [mL]	x	1/q [mol^{-1}]	$NEt_3 \cdot 3HF$ [mL]	Olah's Reagent [mL]
1:4.5	0.5	3.153	1506.4	0.34	0.16
1:5.0	0.5	2.115	1168.4	0.29	0.21
1:5.5	0.5	1.492	965.5	0.25	0.25
1:6.0	0.5	1.076	830.4	0.21	0.29
1:6.5	0.5	0.780	733.6	0.17	0.33
1:7.0	0.5	0.558	661.4	0.14	0.36
1:7.5	0.5	0.384	604.7	0.10	0.40
1:6.0	1.25	1.076	332.2	0.53	0.72

2. Reaction Optimization – Extended Table

2.1. Racemic Reaction

Table 2. Reaction optimization [a]

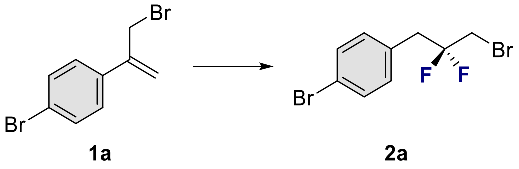

entry	solvent	amine/HF ratio	catalyst	catalyst loading [mol%]	oxidant	yield [%][b]
1	$CHCl_3$	1:3.0	*p*-TolI	20	Selectfluor®	0
2	$CHCl_3$	1:4.5	*p*-TolI	20	Selectfluor®	72
3	$CHCl_3$	1:5.0	*p*-TolI	20	Selectfluor®	81
4	$CHCl_3$	1:5.5	*p*-TolI	20	Selectfluor®	90
5	$CHCl_3$	1:6.0	*p*-TolI	20	Selectfluor®	>95 (80)[c]
6[d]	$CHCl_3$	1:6.0	*p*-TolI	20	Selectfluor®	91
7[e]	$CHCl_3$	1:6.0	*p*-TolI	20	Selectfluor®	39
8	$CHCl_3$	1:6.5	*p*-TolI	20	Selectfluor®	>95
9	$CHCl_3$	1:7.0	*p*-TolI	20	Selectfluor®	>95
10	$CHCl_3$	1:7.5	*p*-TolI	20	Selectfluor®	94
11	$CHCl_3$	1:9.23	*p*-TolI	20	Selectfluor®	87
12	$CHCl_3$	1:6.0	*p*-TolI	10	Selectfluor®	87
13	$CHCl_3$	1:6.0	*p*-TolI	5	Selectfluor®	79
14	MeCN	1:6.0	*p*-TolI	20	Selectfluor®	50
15	DCE	1:6.0	*p*-TolI	20	Selectfluor®	93
16	ETFA	1:6.0	*p*-TolI	20	Selectfluor®	84
17	DCM	1:6.0	*p*-TolI	20	Selectfluor®	>95
18	$CHCl_3$	1:6.0	*p*-TolI	20	Oxone®	66
19	$CHCl_3$	1:6.0	*p*-TolI	20	*m*-CPBA	89
20	$CHCl_3$	1:6.0	4-iodoanisole	20	Selectfluor®	49
21	$CHCl_3$	1:6.0	methyl 4-iodobenzoate	20	Selectfluor®	25
22	$CHCl_3$	1:6.0	-	0	Selectfluor®	0
23	$CHCl_3$	1:6.0	*p*-TolI	20	-	0
24	$CHCl_3$	-	*p*-TolI	20	Selectfluor®	0

[a]Standard reaction conditions: (0.2 mmol) of **1a**, Selectfluor® (1.5 equiv), amine:HF source (0.5 mL), solvent (0.5 mL), *p*-TolI (catalyst), 24h, rt. [b]Determined by ^{19}F NMR using α,α,α-trifluorotoluene as internal standard. [c]Isolated yield [d]Amine:HF source (0.25 mL), solvent (0.75 mL), [e]Amine:HF Source (0.1 mL), solvent (0.9 mL).

2.2. Enantioselective Reaction

Table 3. Reaction optimization [a]

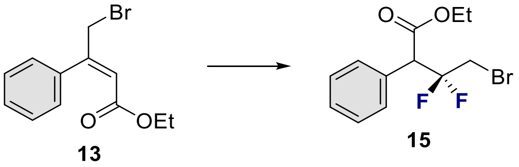

entry	solvent	amine/HF ratio	catalyst	NMR yield **14** [%][b]	e.r	temperature
1	$CHCl_3$	1:9.23	C1	<5	31:69	rt
2	$CHCl_3$	1:9.23	C2	21	33:67	rt
3	$CHCl_3$	1:9.23	C3	75	23:77	rt
4	$CHCl_3$	1:9.23	C4	30	18:82	rt
5[c]	$CHCl_3$	1:9.23	C4	76	18:82	rt
6	$CHCl_3$	1:9.23	C5	26	32:68	rt
7	$CHCl_3$	1:9.23	C6	68	31:69	rt
8	$CHCl_3$	1:9.23	C3	22	23:77	5˚C
9	$CHCl_3$	1:9.23	C4	<5	n.d.	5˚C
10	$CHCl_3$	1:9.23	-	-	-	rt

[a]Standard reaction conditions: (0.1 mmol) of **13**, Selectfluor® (1.5 equiv), amine:HF source (0.25 mL), solvent (0.25 mL), catalyst (0.2 equiv.), 24h. [b]Determined by ^{19}F NMR using α,α,α-trifluorotoluene as internal standard. [c]Reaction time was extended to five days.

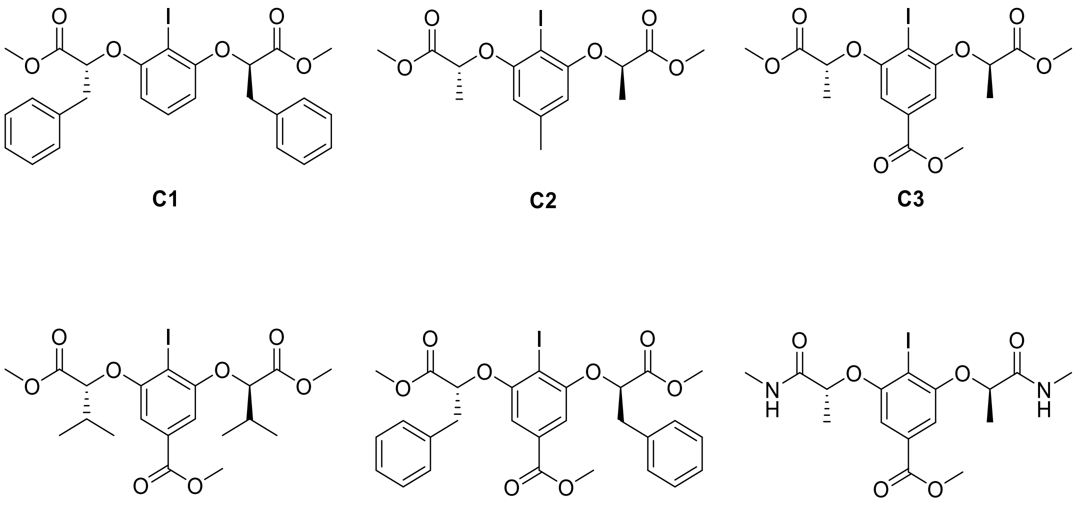

3. Synthetic Procedures

3.1. Preparation of the Catalysts

Methyl 3,5-dihydroxy-4-iodobenzoate (S1)

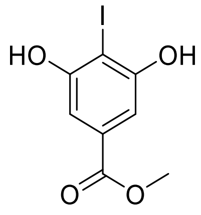

Compound **S1** was prepared according to a modified literature procedure of *Berliner et al.*. [1]

A solution of I_2 (7.60 g, 30.0 mmol, 2.0 eq.) in THF (20 mL) was added dropwise over a time period of 30 minutes to a solution of methyl 3,5-dihydroxybenzoate (2.52 g, 15.0 mmol, 1.0 eq.) and $NaHCO_3$ (3.78 g, 45.0 mmol, 3.0 eq.) in H_2O/THF (10:3, 32.5 mL) at 0˚C. After stirring for one hour at 0˚C, the reaction was quenched using sat. aqueous NaS_2O_3. The reaction mixture was extracted twice with MTBE and the combined organic layers were dried over Na_2SO_4. The solvent was removed and the residual solid was washed multiple times with cold MTBE in order to remove a yellow impurity. Compound **S1** was obtained as an off-white solid (3.18 g, 10.8 mmol, 72%) and was used without further purification.

R*f* = 0.29 (pentane)

^{1}H NMR (400 MHz, DMSO-*d*$_6$): δ [ppm] = 10.54 (s, 2H), 6.93 (s, 2H), 3.80 (s, 3H).

ESI-MS: (*m/z*) requires: [($C_8H_6IO_4$)$^-$] = 292.9316, (*m/z*) found: [($C_8H_6IO_4$)$^-$] = 292.9309.

The analytical data are in good agreement with the literature [1].

Methyl (*S*)-2-hydroxy-3-methylbutanoate ((*S*)-S2)

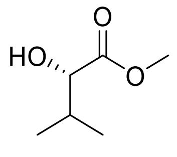

Compound **(*S*)-S2** was prepared according to a modified literature procedure of *Hasuoka et al*. [2]

A solution of *L*-valine (10.0 g, 85.0 mmol, 1.0 eq.) in 1M aqueous H_2SO_4 (170 mL) was cooled to 0˚C. A solution of $NaNO_2$ (35.3 g, 510 mmol, 6.0 eq.) in water (50 mL) was added dropwise over a time period of 2h while stirring at 0˚C. The reaction mixture was stirred for additional 5h at 0˚C. The mixture was warmed to room temperature and extracted twice with Et_2O. The combined organic layers were dried over $MgSO_4$ and the solvent was removed under reduced pressure. The crude (*S*)-2-hydroxy-3-methylbutanoic acid was obtained as a yellow oil. The crude product was

dissolved in MeOH (60 mL). H_2SO_4 (1.2 mL) was added and the mixture was heated to reflux and stirred for 5 h. The mixture was cooled to room temperature and extracted with Et_2O (3x). The combined organic layers were washed with saturated aqueous $NaHCO_3$ (3x) and brine. The combined organic layers were dried over Na_2SO_4 and the solvent was removed under reduced pressure. Crude **(*S*)-S2** was obtained as a yellow oil which was used without further purification (4.06 g, 30.7 mmol, 36%).

R_f = 0.60 (30% EtOAc in pentane)

^{1}H NMR (400 MHz, $CDCl_3$): δ [ppm] = 4.08 (dd, *J* = 6.1, 3.4 Hz, 1H), 3.82 (s, 3H), 2.70 (d, *J* = 6.1 Hz 1H), 2.17 (hd, *J* = 6.9, 3.5 Hz, 1H), 1.04 (d, *J* = 6.9 Hz, 3H), 0.88 (d, *J* = 6.9 Hz, 3H).

ESI-MS: (*m/z*) requires: $[(C_6H_{12}O_3Na)^+]$ = 155.0679, found: $[(C_6H_{12}O_3Na)^+]$ = 155.0676.

The analytical data are in good agreement with the literature [2].

Analytically pure (*S*)-2-hydroxy-3-methylbutanoic acid was obtained by recrystallizing the crude product after the deamination step in a mixture of hexanes and EtOAc (3.5% EtOAc).

^{1}H NMR (400 MHz, $CDCl_3$): δ [ppm] = 4.16 (d, *J* = 3.4 Hz, 1H), 2.17 (hd, *J* = 6.9, 3.5 Hz, 1H), 1.07 (d, *J* = 6.9 Hz, 3H), 0.93 (d, *J* = 6.9 Hz, 3H).

ESI-MS: (*m/z*) requires: $[(C_5H_9O_3)^-]$ = 117.0546, found: $[(C_5H_9O_3)^-]$ = 117.0557.

The analytical data are in good agreement with the literature [2].

Methyl (*R*)-2-hydroxy-3-methylbutanoate ((*R*)-S2)

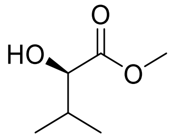

Compound **(*R*)-S2** was prepared according to the procedure followed for compound **(*S*)-S2**. Crude **(*R*)-S2** was obtained as a yellow oil and was used without further purification (631 mg, 4.80 mmol, 48%).

ESI-MS: (*m/z*) requires: $[(C_6H_{12}O_3Na)^+]$ = 155.0679, found: $[(C_6H_{12}O_3Na)^+]$ = 155.0677.

Dimethyl 2,2'-((2-iodo-1,3-phenylene)*bis*(oxy))(2*R*,2'*R*)-*bis*(3-phenylpropanoate) (C1)

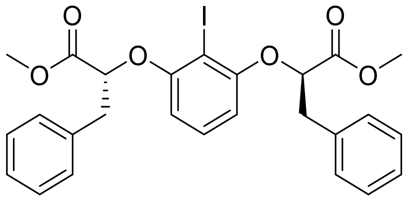

Compound **C1** was prepared previously by this laboratory. [3]

Dimethyl 2,2'-((2-iodo-5-methyl-1,3-phenylene)*bis*(oxy))(2*R*,2'*R*)-dipropionate (C2)

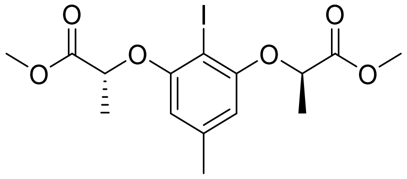

Compound **C2** was prepared previously by this laboratory. [3]

Dimethyl 2,2'-((2-iodo-5-(methoxycarbonyl)-1,3-phenylene)*bis*(oxy))(2*R*,2'*R*)-dipropionate (C3)

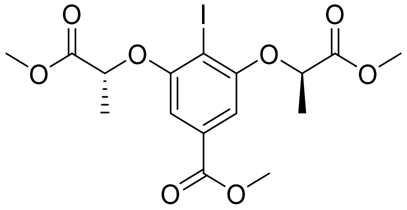

Compound **C3** was prepared according to a literature procedure from this laboratory. [4]

Methyl 3,5-dihydroxy-4-iodobenzoate **S1** (590 mg, 2.00 mmol, 1.0 eq.), methyl (*S*)-lactate (458 mg, 4.40 mmol, 2.2 eq.) and PPh_3 (1.37 g, 4.60 mmol, 2.3 eq.) were dissolved in dry THF and the reaction mixture was cooled to 0˚C. DIAD (0.97 g, 4.80 mmol, 2.4 eq.) was added dropwise and the reaction mixture was slowly warmed to room temperature overnight. The solvent was removed under reduced pressure. The crude material was purified by column chromatography (DCM) to afford compound **C3** as an off-white solid (810 mg, 1.74 mmol, 87%).

R*f* = 0.35 (30% EtOAc in pentane)

^{1}H NMR (400 MHz, $CDCl_3$): δ [ppm] = 7.01 (s, 2H), 4.88 (q, J = 6.8 Hz, 2H), 3.88 (s, 3H), 3.76 (s, 6H), 1.72 (d, J = 6.8 Hz, 6H).

ESI-MS: (*m/z*) requires: [$(C_{16}H_{19}IO_8Na)^+$] = 489.0017, (*m/z*) found: [$(C_{16}H_{19}IO_8Na)^+$] = 489.0012.

The analytical data are in good agreement with the literature [5].

Dimethyl 2,2'-((2-iodo-5-(methoxycarbonyl)-1,3-phenylene)*bis*(oxy))(2*R*,2'*R*)-*bis*(3-methylbutanoate) (C4)

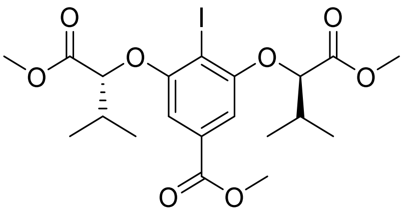

Compound **C4** was prepared according to a literature procedure from this laboratory. [4].

Methyl 3,5-dihydroxy-4-iodobenzoate **S1** (1.18 g, 4.00 mmol, 1.0 eq.), methyl (*S*)-2-hydroxy-3-methylbutanoate **(*S*)-S2** (1.16 g, 8.80 mmol, 2.2 eq.) and PPh_3 (2.74 g, 9.2 mmol, 2.3 eq.) were dissolved in dry THF and the reaction mixture was cooled to 0˚C. DIAD (1.94 g, 9.6 mmol, 2.4 eq.) was added dropwise and the reaction mixture was slowly warmed to room temperature overnight. The solvent was removed under reduced pressure. The crude material was purified by column chromatography (DCM and 20% EtOAc in pentane) to afford compound **C4** as a yellowish oil (1.14 g, 2.18 mmol, 55%). The enantiomeric ratio was determined by chiral HPLC (chiral AS-H column, *n*-hexane : isopropanol 95:5, 0.5 mL/min, t_R = 13.01 min, *e.r.* = 1:>99).

R_f = 0.45 (30% EtOAc in pentane)

ORD ($CHCl_3$, c 1.00) $[\alpha]_D^{25}$ = +36.025

^{1}H NMR (400 MHz, $CDCl_3$): δ [ppm] = 6.90 (s, 2H), 4.58 (d, *J* = 4.5 Hz, 2H), 3.87 (s, 3H), 2.39 (hd, *J* = 6.8, 4.5 Hz, 1H), 1.17 (d, *J* = 6.8 Hz, 3H), 1.14 (d, *J* = 6.8 Hz, 3H).

ESI-MS: (*m/z*) requires: [$(C_{20}H_{27}IO_8Na)^+$] = 545.0643, (*m/z*) found: [$(C_{20}H_{27}IO_8Na)^+$] = 545.0638.

The analytical data are in good agreement with the literature [5].

HPLC trace **C4**

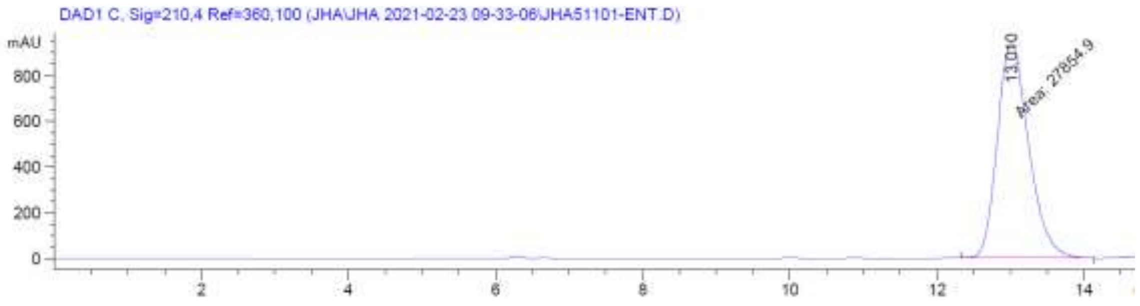

Signal 3: DAD1 C, Sig=210,4 Ref=360,100

Peak #	RetTime [min]	Type	Width [min]	Area [mAU*s]	Height [mAU]	Area %
1	13.010	MM	0.4955	2.78549e4	936.93427	100.0000
Totals :				2.78549e4	936.93427	

Dimethyl 2,2'-((2-iodo-5-(methoxycarbonyl)-1,3-phenylene)*bis*(oxy))(2*S*,2'*S*)-*bis*(3-methylbutanoate) ((*S*,*S*')-C4)

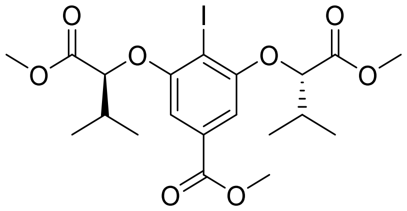

Compound **(*S*,*S*')-C4** was prepared according to a the procedure performed for compound **C4** using methyl (*R*)-2-hydroxy-3-methylbutanoate **(*R*)-S2** (291 mg, 2.20 mmol). The crude material was purified by column chromatography (DCM and 20% EtOAc in pentane) to afford compound **(*S*,*S*')-C4** as a yellowish oil (283 mg, 0.54 mmol, 54%). The enantiomeric ratio was determined by chiral HPLC (chiral AS-H column, *n*-hexane : isopropanol 95:5, 0.5 mL/min, t_R = 11.23 min, *e.r.* = >99:1).

ESI-MS: (*m/z*) requires: $[(C_{20}H_{27}IO_8Na)^+]$ = 545.0643, (*m/z*) found: $[(C_{20}H_{27}IO_8Na)^+]$ = 545.0634.

ORD ($CHCl_3$, c 1.00) $[\alpha]_D^{25}$ = -34.937

HPLC trace **(*S*,*S*')-C4**

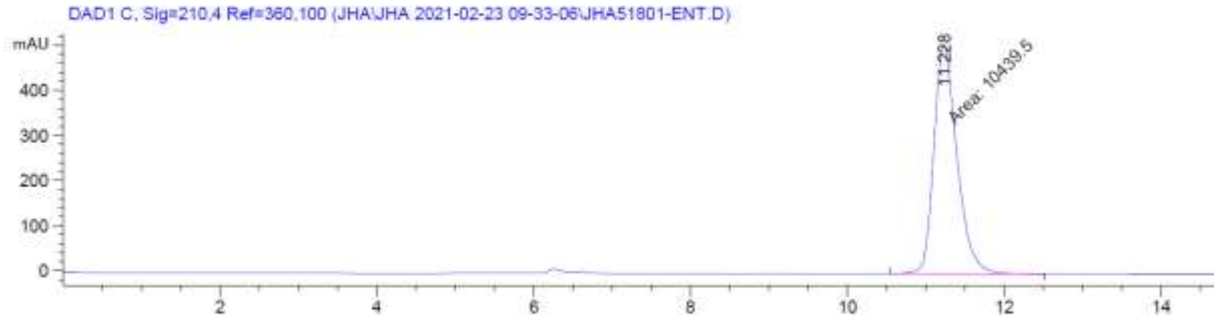

Signal 3: DAD1 C, Sig=210,4 Ref=360,100

Peak #	RetTime [min]	Type	Width [min]	Area [mAU*s]	Height [mAU]	Area %
1	11.228	MM	0.3423	1.04395e4	508.29779	100.0000

Totals : 1.04395e4 508.29779

HPLC trace of a mixture of **C4** and **(*S*,*S*')-C4** (roughly 0.5 mg each in 1 mL 5% *i*-PrOH in hexanes).

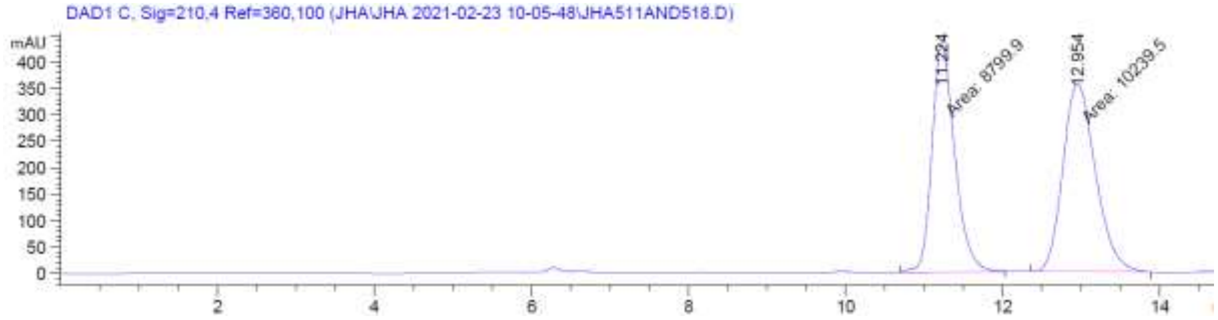

Dimethyl 2,2'-((2-iodo-5-(methoxycarbonyl)-1,3-phenylene)*bis*(oxy))(2*R*,2'*R*)-*bis*(3-phenylpropanoate) (C4)

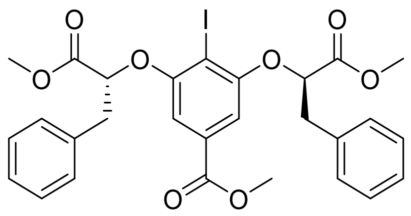

Compound **C5** was prepared according to a literature procedure from this laboratory. [4].

Methyl 3,5-dihydroxy-4-iodobenzoate **S1** (1.18 g, 4.00 mmol, 1.0 eq.), methyl (*S*)-2-hydroxy-3-phenylpropanoate (1.58 g, 8.80 mmol, 2.2 eq.) and PPh_3 (2.74 g, 9.20 mmol, 2.3 eq.) were dissolved in dry THF and the reaction mixture was cooled to 0˚C. DIAD (1.94 g, 9.60 mmol, 2.4 eq.) was added dropwise and the reaction mixture was slowly warmed to room temperature overnight. The solvent was removed under reduced pressure. The crude material was purified by column chromatography (DCM and 20% EtOAc in pentane) to afford the compound **C5** as a white solid (2.07 g, 3.35 mmol, 84%).

R_f = 0.40 (30% EtOAc in pentane)

^{1}H NMR (400 MHz, $CDCl_3$): δ [ppm] = 7.44 – 7.39 (m, 2H), 7.35 – 7.27 (m, 2H), 7.26 – 7.24 (m, 1H), 6.86 (s, 2H), 4.92 (dd, *J* = 7.6, 4.8 Hz, 2H), 3.83 (s, 3H), 3.69 (s, 6H), 3.41 – 3.26 (m, 4H).

ESI-MS: (*m/z*) requires: $[(C_{28}H_{27}IO_8Na)^+]$ = 641.0643, (*m/z*) found: $[(C_{28}H_{27}IO_8Na)^+]$ = 641.0640.

The analytical data are in good agreement with the literature [5].

Methyl 4-iodo-3,5-*bis*(((*R*)-1-(methylamino)-1-oxopropan-2-yl)oxy)benzoate (C6)

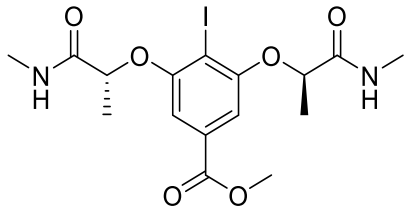

Compound **C6** was prepared previously by this laboratory. [5]

3.2. Acetophenones

4-Acetylphenyl 4-methylbenzenesulfonate (S3)

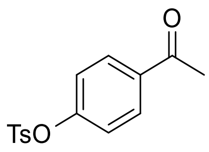

The reaction was performed according to the procedure of *Lei et al* [6].

A solution of K_2CO_3 (2.60 g, 19.0 mmol, 1.9 eq.) in H_2O (25 mL) was added to a pre-stirred solution of 1-(4-hydroxyphenyl)ethan-1-one (1.36 g, 10.0 mmol, 1.0 eq.) in THF (6 mL). The mixture was cooled to 0 °C and a solution of TsCl (1.93 g, 10.1 mmol, 1.01 eq.) in THF (14 mL) was added over a 15 min time period. The mixture was slowly warmed to rt and stirred for 6 h. EtOAc (50 mL) was added and the layers were separated. The organic layer was washed with water, was dried over $MgSO_4$ and the solvent was removed under reduced pressure. The obtained crude product was washed multiple times with hexanes and compound **S3** was obtained as a white solid (2.61 g, 9.00 mmol, 90%), which was used without further purification.

R*f* = 0.18 (20% EtOAc in cyclohexane)

^{1}H NMR (400 MHz, $CDCl_3$): δ[ppm] = 7.90 (d, *J* = 8.8 Hz, 2H), 7.71 (d, *J* = 8.4 Hz, 2H), 7.32 (d, *J* = 8.3 Hz, 2H), 7.09 (d, *J* = 8.8 Hz, 2H), 2.58 (s, 3H), 2.46 (s, 3H).

ESI-MS: (*m/z*) requires: [$(C_{15}H_{14}O_4SNa)^+$] = 313.0505, (*m/z*) found: [$(C_{15}H_{14}O_4SNa)^+$] = 313.0504.

The analytical data are in good agreement with the literature [7].

1-(4-(Piperidin-1-ylsulfonyl)phenyl)ethan-1-one (S4)

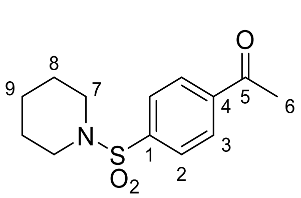

The reaction was performed in accordance with previous patent literature [8].

A mixture of 4-acetylbenzenesulfonyl chloride (1.09 g, 5.00 mmol, 1.0 eq.), piperidine (468 mg, 543 µL, 5.50 mmol, 1.1 eq.) and triethylamine (1.01 g, 1.39 mL, 10.0 mmol, 1.0 eq.) in THF (15 mL) was stirred at room temperature for 4 hours. The mixture was diluted with EtOAc and water and the phases were separated. The aqueous layer was back-washed twice using additional portions of EtOAc, and the combined organic layers were washed with brine, dried over Na_2SO_4

and concentrated under reduced pressure. The crude product did not require further purification, and compound **S4** was isolated as a white solid (1.19 g, 4.40 mmol, 89%).

R*f* = 0.51 (60% Et_2O in pentane).

Melting point: 117-119°C

^{1}H NMR (599 MHz, $CDCl_3$) δ [ppm] 8.10 – 8.05 (m, 2H, H-C3), 7.86 – 7.79 (m, 2H, H-C2), 3.03 – 2.98 (m, 4H, H-C7), 2.65 (s, 3H, H-C6), 1.63 (p, $^3J_{HH}$ = 5.9 Hz, 4H, H-C8), 1.42 (tt, $^3J_{HH}$ = 8.3, $^3J_{HH}$ = 4.7 Hz, 2H, H-C9).

^{13}C NMR (151 MHz, $CDCl_3$) δ [ppm] 197.0 (C5), 140.7 (C1), 140.1 (C4), 128.9 (C3), 128.0 (C2), 47.0 (C7), 27.0 (C6), 25.3 (C8), 23.6 (C9).

ESI-MS: (*m/z*) requires: [($C_{13}H_{17}NO_3SNa$)] = 290.0821, (*m/z*) found: [($C_{13}H_{17}NO_3SNa$)] = 290.0819.

FT-IR ($\tilde{\nu}$ = cm^{-1})**:** 2939 (w), 2849 (w), 1689 (m), 1472 (w) ,1443 (w),1400 (w),1353 (w),1338 (m), 1309 (w), 1285 (m), 1263 (m),1208 (w), 1163 (s), 1096 (m), 1054 (w), 1028 (w), 1013 (w), 964 (w), 927 (m), 860 (w), 833 (m), 776 (w), 730 (w), 715 (m).

Methyl (*E*)-3-(4-acetylphenyl)acrylate (S5)

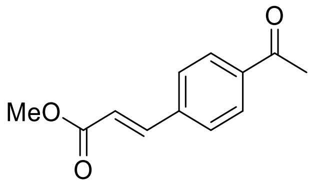

The reaction was performed in accordance with previous patent literature [9].

To 4-bromoacetophenone (1.00 g, 5.00 mmol, 1.0 eq.) in anhydrous DMF (30 mL) was added methyl methacrylate (430 mg, 450 µL, 5.00 mmol, 1.0 eq.), sodium bicarbonate (420 mg, 5.00 mmol, 1.0 eq.), tetrabutylammonium bromide (1.61 mg, 5.00 mmol), and palladium(II) acetate (56.0 mg, 0.25 mmol, 5 mol%). The flask was fitted with a reflux condenser and the reaction mixture was stirred at 130 °C for one hour. The reaction mixture was filtered through Celite and washed with EtOAc. The filtrate was partitioned between EtOAc and saturated ammonium chloride solution, and the phases were separated. The aqueous layer was back-washed with a further portion of EtOAc, and the combined organic layers were washed with brine, dried over sodium sulfate, and concentrated under reduced pressure. The crude material was purified by column chromatography

(0-17.5% EtOAc in pentane) to afford the compound **S5** as a pale-yellow solid (901 mg, 4.4 mmol, 88%).

Melting point: 100-102 °C

R*f* = 0.41 (20% EtOAc in pentane).

^{1}H NMR (400 MHz, $CDCl_3$) δ [ppm] 7.97 (d, *J* = 8.4, 2H), 7.70 (d, *J* = = 16.1 Hz, 1H), 7.60 (d, *J* = 8.3 Hz, 2H), 6.52 (d, *J* = 16.1 Hz, 1H), 3.82 (s, 3H), 2.61 (s, 3H).

ESI-MS: (*m/z*) requires: $[(C_{12}H_{12}O_3Na)^+]$ = 227.0679, (*m/z*) found: $[(C_{12}H_{12}O_3Na)^+]$ = 227.0679.

FT-IR ($\tilde{\nu}$ = cm^{-1})**:** $\tilde{\nu}$ = 3072 (w), 2954 (w), 1711 (m), 1673 (m), 1635 (m), 1603 (w), 1439 (w), 1412 (w), 1362 (w), 1329 (m), 1287 (w), 1264 (m), 1198 (m), 1175 (s), 1076 (w), 1006 (s), 965 (w), 935 (w), 889 (w), 848 (m), 824 (s), 751 (w), 740 (w), 684 (m) cm^{-1}.

The analytical data are in good agreement with the literature. [10]

3.3 α-(Methyl)styrenes

General Procedure A:

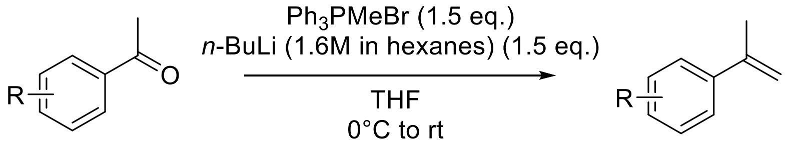

The reaction was performed according to a modified procedure of Overman *et al.* [11]

A Schlenk flask was charged with dry THF (0.4 M) and Ph_3PMeBr (1.5 eq.). *n*-BuLi (1.6M in hexanes, 1.5 eq.) was added portionwise at 0°C and the resulting yellow solution was stirred for 30 min. The acetophenone derivative (1.0 eq.) was added dropwise as a solution in THF or directly as a liquid and the reaction mixture was allowed to warm to ambient temperature overnight. Pentane or EtOAc was added followed by the addition of water. The aqueous layer was extracted with pentane (3x) or EtOAc (3x). The combined organic layers were washed with brine (1x) and dried over $MgSO_4$. The solvent was removed under reduced pressure and the crude mixture was purified by column chromatography.

General Procedure B:

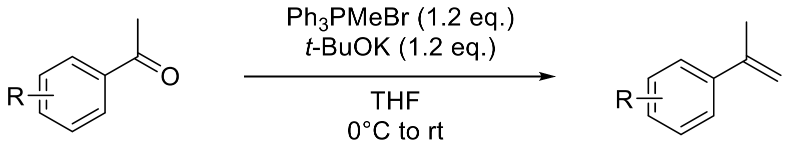

The reaction was performed according to a modified procedure from this laboratory. [12]

A Schlenk flask was charged with dry THF (0.5 M) and Ph_3PMeBr (1.2 eq.). *t*-BuOK (1.2 eq.) was added portionwise at 0 °C and the resulting yellow suspension was stirred for 10 min, then warmed to room temperature and stirred for another hour. The acetophenone derivative (1.0 eq.) was added in portions and the reaction mixture was allowed to warm to ambient temperature overnight. EtOAc and water was added and the aqueous layer was extracted with EtOAc (3x). The combined organic layers were dried over $MgSO_4$, the solvent was removed under reduced pressure and the crude mixture was purified by column chromatography.

1-Bromo-4-(prop-1-en-2-yl)benzene (S6)

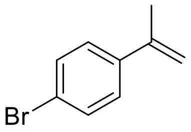

Compound **S6** was prepared according to General Procedure **A** using 1-(4-bromophenyl)ethan-1-one (3.98 g, 20.0 mmol). The crude mixture was purified by column chromatography (100% pentane) to yield the title compound as a colorless oil (3.23 g, 16.4 mmol, 82%).

R*f* = 0.72 (pentane)

^{1}H NMR (400 MHz, $CDCl_3$): δ[ppm] = 7.45 (d, *J* = 8.6 Hz, 2H), 7.33 (d, *J* = 8.6 Hz, 2H), 5.36 (q, *J* = 0.8 Hz, 1H), 5.12 – 5.09 (m, 1H), 2.13 (dd, *J* = 1.5, 0.8 Hz, 3H).

GC-EI-MS: Retention: 6.02 min (*m/z*) requires: [(C_9H_9Br)] = 195.99, (*m/z*) found: [(C_9H_9Br)] = 196.00.

The analytical data are in good agreement with the literature [11].

1-Fluoro-4-(prop-1-en-2-yl)benzene (S7)

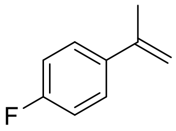

Compound **S7** was prepared according to General Procedure **A** using 1-(4-fluorophenyl)ethan-1-one (1.38 g, 10.0 mmol). The crude mixture was purified by column chromatography (100% pentane) to yield the title compound as a colorless oil (1.07 g, 7.90 mmol, 79%).

R*f* = 0.70 (pentane)

^{1}H NMR (400 MHz, $CDCl_3$): δ[ppm] = 7.46 – 7.40 (m, 2H), 7.05 – 6.97 (m, 2H), 5.30 (s, 1H), 5.08 – 5.05 (m, 1H), 2.14 (s, 3H).

^{19}F NMR (377 MHz, $CDCl_3$): δ[ppm] = -115.40 (tt, *J* = 8.6, 5.4 Hz, 1F).

GC-EI-MS: Retention: 4.45 min (*m/z*) requires: [(C_9H_9F)] = 136.07, (*m/z*) found: [(C_9H_9F)] = 136.12.

The analytical data are in good agreement with the literature [13].

1-Chloro-4-(prop-1-en-2-yl)benzene (S8)

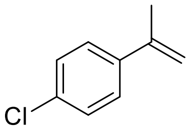

Compound **S8** was prepared according to General Procedure **A** using 1-(4-chlorophenyl)ethan-1-one (775 mg, 5.00 mmol). The crude mixture was purified by column chromatography (100% pentane) to yield the title compound as a colorless oil (677 mg, 4.40 mmol, 88%).

R*f* = 0.75 (pentane)

^{1}H NMR (400 MHz, $CDCl_3$): δ [ppm] = 7.39 (d, *J* = 8.6 Hz, 2H), 7.29 (d, *J* = 8.6 Hz, 2H), 5.35 (s, 1H), 5.11 – 5.08 (m, 1H), 2.13 (s, 3H).

GC-EI-MS: Retention: 6.13 min (*m/z*) requires: [(C_9H_9Cl)] = 152.04, (*m/z*) found: [(C_9H_9Cl)] = 152.04.

The analytical data are in good agreement with the literature [11].

1-Bromo-3-(prop-1-en-2-yl)benzene (S9)

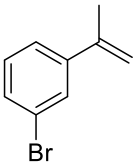

Compound **S9** was prepared according to General Procedure **A** using 1-(3-bromophenyl)ethan-1-one (1.99 g, 10.0 mmol). The crude mixture was purified by column chromatography (100% pentane) to yield the title compound as a colorless oil (1.64 g, 8.40 mmol, 84%).

R*f* = 0.81 (pentane)

^{1}H NMR (400 MHz, $CDCl_3$): δ [ppm] = 7.60 (t, *J* = 1.9 Hz, 1H), 7.41 – 7.37 (m, 2H), 7.20 (t, *J* = 7.9 Hz, 1H), 5.37 (s, 1H), 5.14 – 5.11 (m, 1H), 2.13 (s, 1H).

GC-EI-MS: Retention: 5.97 min (*m/z*) requires: [(C_9H_9Br)] = 195.99, (*m/z*) found: [(C_9H_9Br)] = 196.01.

The analytical data are in good agreement with the literature [11].

1-Bromo-2-(prop-1-en-2-yl)benzene (S10)

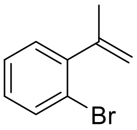

Compound **S10** was prepared according to General Procedure **A** using 1-(2-bromophenyl)ethan-1-one (1.99 g, 10.0 mmol). The crude mixture was

purified by column chromatography (100% pentane) to yield the title compound as a colorless oil (1.58 g, 8.00 mmol, 80%).

R*f* = 0.57 (pentane)

^{1}H NMR (400 MHz, $CDCl_3$): δ [ppm] = 7.55 (dd, *J* = 8.0, 1.2 Hz, 1H), 7.27 (td, *J* = 7.4, 1.2 Hz, 1H), 7.19 (dd, *J* = 7.6, 1.9 Hz, 1H), 7.11 (ddd, *J* = 8.0, 7.3, 1.9 Hz, 1H), 5.23 (app. p, *J* = 1.7 Hz, 1H), 4.94 (dq, *J* = 1.9, 0.9 Hz, 1H), 2.10 (dd, *J* = 1.6, 1.0 Hz, 1H).

GC-EI-MS: Retention: 5.52 min (*m/z*) requires: [(C_9H_9Br)] = 195.99, (*m/z*) found: [(C_9H_9Br)] = 196.01.

The analytical data are in good agreement with the literature [14].

1-(*tert*-Butyl)-4-(prop-1-en-2-yl)benzene (S11)

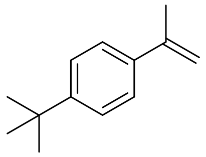

Compound **S11** was prepared according to General Procedure **A** using 1-(4-(*tert*-butyl)phenyl)ethan-1-one (1.41 g, 8.00 mmol). The crude mixture was purified by column chromatography (100% pentane) to yield the title compound as a colorless oil (1.10 g, 6.30 mmol, 79%).

R*f* = 0.62 (pentane)

^{1}H NMR (400 MHz, $CDCl_3$): δ[ppm] = 7.42 (d, *J* = 8.5 Hz, 2H), 7.36 (d, *J* = 8.6 Hz, 2H), 5.36 (s, 1H), 5.06 – 5.04 (m, 1H), 2.15 (s, 3H), 1.33 (s, 9H).

GC-EI-MS: Retention: 6.84 min (*m/z*) requires: [($C_{13}H_{18}$)] = 174.14, (*m/z*) found: [($C_{13}H_{18}$)] = 174.14.

The analytical data are in good agreement with the literature [14].

1-(Prop-1-en-2-yl)-4-(trifluoromethyl)benzene (S12)

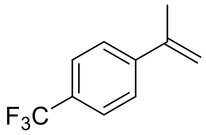

Compound **S12** was prepared according to General Procedure **A** using 1-(4-(trifluoromethyl)phenyl)ethan-1-one (1.88 g, 10.0 mmol). The crude mixture was purified by column chromatography (100% pentane) to yield the title compound as a colorless oil (1.72 g, 9.20 mmol, 92%).

R*f* = 0.72 (pentane)

^{1}H NMR (400 MHz, $CDCl_3$): δ [ppm] = 7.63 – 7.51 (m, 4H), 5.44 (s, 1H), 5.21 – 5.18 (m, 1H), 2.17 (s, 3H).

^{19}F NMR (377 MHz, $CDCl_3$): δ [ppm] = -62.48 (s, 3F).

GC-EI-MS: Retention: 5.14 min (*m/z*) requires: [($C_{10}H_9F_3$)] = 186.07, (*m/z*) found: [($C_{10}H_9F_3$)] = 186.06.

The analytical data are in good agreement with the literature [13].

Methyl 4-(prop-1-en-2-yl)benzoate (S13)

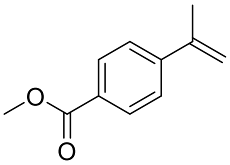

Compound **S13** was prepared according to General Procedure **A** using methyl 4-acetylbenzoate (2.67 g, 15.0 mmol). The crude mixture was purified by column chromatography (5% EtOAc in pentane) to yield the title compound as a white solid (1.16 g, 6.60 mmol, 44%).

R*f* = 0.70 (5% EtOAc in pentane)

^{1}H NMR (400 MHz, $CDCl_3$): δ [ppm] = 7.99 (d, *J* = 8.5 Hz, 2H), 7.52 (d, *J* = 8.5 Hz, 2H), 5.48 – 5.46 (m, 1H), 5.21 – 5.18 (m, 1H), 3.92 (s, 3H), 2.11 (dd, *J* = 1.6, 0.8 Hz, 3H).

ESI-MS: (*m/z*) requires: [($C_{11}H_{12}O_2Na)^+$] = 199.0730, (*m/z*) found: [($C_{11}H_{12}O_2Na)^+$] = 199.0730.

The analytical data are in good agreement with the literature [15].

Methyl 4-(3-hydroxyprop-1-en-2-yl)benzoate (S14)

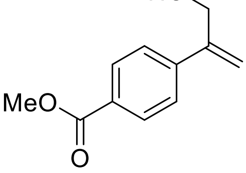

Compound **S14** was prepared according to a procedure of *Schultz et al*. [15].

SeO_2 (125 mg, 1.13 mmol, 0.5 eq.) was suspended in DCM (2 mL) followed by the addition of *t*-BuOOH (5.50 M in nonane, 0.83 mL, 4.54 mmol, 2.0 eq.). After stirring the reaction mixture for 5 minutes at room temperature, a solution of methyl 4-(prop-1-en-2-yl)benzoate **S13** in DCM (2 mL) was

added dropwise. The reaction mixture was stirred for 48 hours at room temperature. DCM (20 mL) and water (20 mL) were added. The organic layer was separated and washed with sat. aq. $NaHCO_3$ (1x) and sat. aq. $Na_2S_2O_3$ (1x). The organic layer was dried over $MgSO_4$ and the crude mixture was purified by column chromatography (0-2% MeOH in DCM) to yield compound **S14** as a white solid (256 mg, 1.33 mmol, 59%).

R*f* = 0.21 (1% MeOH in DCM)

^{1}H NMR (400 MHz, $CDCl_3$): δ [ppm] = 8.02 (d, *J* = 8.5 Hz, 2H), 7.53 (d, *J* = 8.5 Hz, 2H), 5.61 – 5.57 (m, 1H), 5.49 – 5.45 (m, 1H), 4.58 – 4.56 (m, 2H), 3.92 (s, 3H), 1.55 (broad s, 1H).

ESI-MS: (*m/z*) requires: $[(C_{11}H_{12}O_3Na)^+]$ = 215.07, (*m/z*) found: $[(C_{11}H_{12}O_3Na)^+]$ = 215.07.

The analytical data are in good agreement with the literature [15].

1-Nitro-4-(prop-1-en-2-yl)benzene (S15)

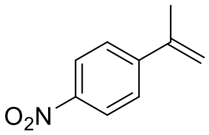

Compound **S15** was prepared according to modified General Procedure **B** using 1-(4-nitrophenyl)ethan-1-one (1.65 g, 10.0 mmol). The mixture was stirred for 22 hours at 60°C. The crude mixture was purified by column chromatography (5% EtOAc in cyclohexane) to yield the title compound as a yellow solid (696 mg, 4.30 mmol, 43%).

R*f* = 0.38 (5% EtOAc in cyclohexane)

^{1}H NMR (400 MHz, $CDCl_3$): δ [ppm] = 8.22 – 8.16 (m, 2H), 7.62 – 7.56 (m, 2H), 5.52 (app. p, *J* = 0.9 Hz, 1H), 5.29 (app. p, *J* = 1.4 Hz, 1H), 2.11 (dd, *J* = 1.5, 0.8 Hz, 3H).

GC-EI-MS: Retention: 7.38 min (*m/z*) requires: $[(C_9H_9NO_2)]$ = 163.06, (*m/z*) found: $[(C_9H_9NO_2)]$ = 163.06.

The analytical data are in good agreement with the literature [12].

4-(Prop-1-en-2-yl)phenyl 4-methylbenzenesulfonate (S16)

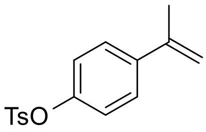

Compound **S16** was prepared according to General Procedure **A** using 4-acetylphenyl 4-methylbenzenesulfonate **S3** (2.32 g, 8.00 mmol). The crude mixture was purified by column chromatography (20% EtOAc in cyclohexane followed by 20% pentane in DCM) to yield the title compound as a white crystalline solid (1.58 g, 5.50 mmol, 69%).

$\mathbf{R}_{f}$ = 0.44 (20% EtOAc in cyclohexane); $\mathbf{R}_{f}$ = 0.88 (DCM)

^{1}H NMR (400 MHz, $CDCl_3$): δ[ppm] = 7.72 (d, *J* = 8.3 Hz, 2H), 7.37 (d, *J* = 8.8 Hz, 2H), 7.31 (d, *J* = 8.1 Hz, 2H), 6.93 (d, *J* = 8.8 Hz, 2H), 5.32 (s, 1H), 5.10 – 5.07 (m, 1H), 2.45 (s, 3H), 2.10 (s, 1H).

ESI-MS: (*m/z*) requires: $[(C_{16}H_{16}O_3SNa)^+]$ = 311.0712, (*m/z*) found: $[(C_{16}H_{16}O_3SNa)^+]$ = 311.0709.

The analytical data are in good agreement with the literature [16].

2-Bromo-1-fluoro-4-(prop-1-en-2-yl)benzene (S17)

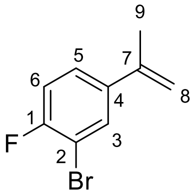

Compound **S17** was prepared according to General Procedure **A** using 1-(3-bromo-4-fluorophenyl)ethan-1-one (2.17 g, 10.0 mmol). The crude mixture was purified by column chromatography (100% pentane) to yield the title compound as a colorless oil (1.83 g, 8.50 mmol, 85%).

$\mathbf{R}_{f}$ = 0.80 (pentane)

^{1}H NMR (599 MHz, $CDCl_3$): δ[ppm] = 7.63 (dd, $^{4}J_{HF}$ = 6.6, $^{4}J_{HH}$ = 2.3 Hz, 1H, H-C3), 7.36 (ddd, $^{3}J_{HH}$ = 8.6, $^{4}J_{HF}$ = 4.7, $^{4}J_{HH}$ = 2.3 Hz, 1H, H-C5), 7.07 (t, $^{3}J_{HH}$ = $^{3}J_{HF}$ = 8.5 Hz, 1H, H-C6), 5.32 (dq, $^{2}J_{HH}$ = 1.4, $^{4}J_{HH}$ = 0.6 Hz, 1H, H^{a}-C8), 5.11 (app. p, $^{2}J_{HH}$ = $^{4}J_{HH}$ = 1.6 Hz, 1H, H^{b}-C8), 2.11 (dd, $^{4}J_{HH}$ = 1.5, $^{4}J_{HH}$ = 0.8, 3H, H-C9).

^{19}F NMR (564 MHz, $CDCl_3$): δ [ppm] = -109.67 (ddd, $^{3}J_{HF}$ = 8.3, $^{4}J_{HF}$ = 6.6, $^{4}J_{HF}$ = 4.6 Hz, 1F).

^{19}F NMR (564 MHz, $CDCl_3$): δ [ppm] = -109.67 (ddd, $^{3}J_{HF}$ = 8.3, $^{4}J_{HF}$ = 6.6, $^{4}J_{HF}$ = 4.6 Hz, 1F, F-C1).

^{19}F{^{1}H} NMR (564 MHz, $CDCl_3$): δ[ppm] = -109.67 (s, 1F, F-C1).

$^{13}C\{^1H\}$ NMR (151 MHz, $CDCl_3$) δ [ppm] 158.6 (d, $^1J_{CF}$ = 247.6 Hz, C1), 141.3 (d, $^4J_{CF}$ = 1.0 Hz, C4), 139.1 (d, $^5J_{CF}$ = 4.0 Hz, C7), 130.8 (s, C3), 126.2 (d, $^3J_{CF}$ = 7.1 Hz, C5), 116.17 (d, $^2J_{CF}$ = 22.3 Hz, C6), 113.61 (d, $^6J_{CF}$ = 1.7 Hz, C8), 109.0 (d, $^2J_{CF}$ = 21.1 Hz, C2), 21.9 (s, C9)

GC-EI-MS: Retention: 6.57 min (*m/z*) requires: [(C_9H_8BrF)] = 213.9788, (*m/z*) found: [(C_9H_8BrF)] = 213.9787.

FT-IR ($\tilde{\nu}$ = cm^{-1})**:** 3088.7 (w), 2977.6 (w), 1630.2 (w), 1596.4 (w), 1575.4 (w), 1495.3 (s), 1453.6 (w), 1438.9 (w), 1382.0 (m), 1302.0 (m), 1262.5 (m), 1240.0 (m), 1161.1 (w), 1140.7 (w), 1115.9 (w), 1069.2 (w), 1045.3 (m), 1006.2 (w), 933.9 (w), 880.8 (s), 847.6 (m), 820.2 (s), 732.5 (m), 711.7 (s), 684.6 (w), 661.1 (m).

1-((4-(Prop-1-en-2-yl)phenyl)sulfonyl)piperidine (S18)

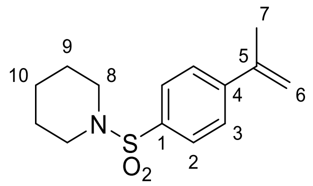

Compound **S18** was prepared according to General Procedure **B** using 1-(4-(piperidin-1-ylsulfonyl)phenyl)ethan-1-one **S4** (1.19 g, 5.34 mmol). The crude mixture was purified by column chromatography (0-6% EtOAc in pentane) to afford the title compound as a white solid (724 mg, 3.26 mmol, 61%).

R*f* = 0.41 (10% EtOAc in pentane).

Melting point: 89-91°C

^1H NMR (500 MHz, $CDCl_3$) δ [ppm] 7.70 (m, 2H, H-C2), 7.58 (m, 2H, H-C3), 5.47 (m, 1H, H^a-C6), 5.23 (app. p, $^2J_{HH}$, $^4J_{HH}$ = 1.5 Hz, 1H, H^b-C6), 3.02 - 2.96 (m, 4H, H-C8), 2.17 (dd, $^4J_{HH}$ = 1.5, $^4J_{HH}$ = 0.8 Hz, 3H, H-C7), 1.68 – 1.59 (m, 4H, H-C9), 1.45 - 1.38 (m, 2H, H-C10).

$^{13}C\{^1H\}$ NMR (126 MHz, $CDCl_3$) δ [ppm] 145.6 (C5), 142.0 (C4), 135.0 (C1), 127.8 (C2), 126.0 (C3), 115.4 (C7), 47.0 (C8), 25.3 (C9), 23.6 (C10), 21.7 (C6).

ESI-MS: (*m/z*) requires: [$(C_{14}H_{19}NO_2SNa)^+$] = 288.1029, (*m/z*) found: [$(C_{14}H_{19}NO_2SNa)^+$] = 288.1024.

FT-IR ($\tilde{\nu}$ = cm^{-1})**:** 2963 (w), 2930 (w), 2850 (w), 1447 (w), 1397 (w), 1358 (w), 1337 (s), 1326 (m), 1310 (m), 1275 (w), 1161 (s), 1147 (m), 1116 (m), 1104 (w), 1089 (m), 1053

(m), 1029 (w), 1012 (w), 932 (s), 909 (m), 898 (m), 857 (w), 838 (s), 773 (m), 752 (w), 716 (s), 677 (w).

4-(Prop-1-en-2-yl)aniline (S19)

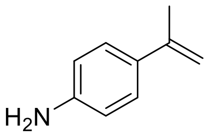

Compound **S19** was prepared according to General Procedure **B** using 1-(4-aminophenyl)ethan-1-one (1.35 g, 10.0 mmol). The crude mixture was purified by column chromatography (DCM) to yield the title compound as an orange oil (952 mg, 7.20 mmol, 72%).

R*f* = 0.43 (DCM)

^{1}H NMR (400 MHz, $CDCl_3$): δ [ppm] = 7.34 – 7.28 (m, 2H), 6.69 – 6.61 (m, 2H), 5.25 (dd, J = 1.6, 0.8 Hz, 1H), 4.29 (app. p, J = 1.5 Hz, 1H), 3.77 (broad s, 2H), 2.11 (dd, J = 1.4, 0.8 Hz, 3H).

ESI-MS: (*m/z*) requires: [$(C_9H_{12}N)^+$] = 134.0964, (*m/z*) found: [$(C_9H_{12}N)^+$] = 134.0963.

The analytical data are in good agreement with the literature [17].

2-(4-(Prop-1-en-2-yl)phenyl)isoindoline-1,3-dione (S20)

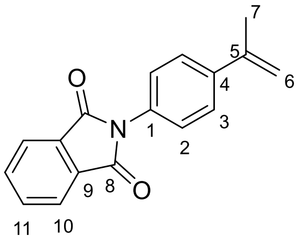

Compound **S20** was prepared according to a procedure of *Cameron et al*. [18]

A solution of 4-(prop-1-en-2-yl)aniline **S19** (665 mg, 5.00 mmol, 1.0 eq.) and phthalic anhydride (778 mg, 5.25 mmol, 1.05 eq.) in acetic acid was refluxed for 5 hours. The mixture was cooled to room temperature and water was added. The precipitated was filtered off and dissolved in DCM. The organic layer was washed with water (3x) and brine (1x). The organic layer was dried over $MgSO_4$. The crude mixture was purified by column chromatography (80% DCM in pentane) to yield compound **S20** as a white solid (401 mg, 1.50 mmol, 30%).

R*f* = 0.54 (80% DCM in pentane)

Melting Point: 216-218°C

^{1}H NMR (599 MHz, $CDCl_3$): δ [ppm] = 7.96 (dd, $^{3}J_{HH}$ = 5.4 Hz, $^{4}J_{HH}$ = 3.1 Hz, 2H, H-C10), 7.80 (dd, $^{3}J_{HH}$ = 5.4 Hz, $^{4}J_{HH}$ = 3.0 Hz, 2H, H-C11), 7.60 (m, 2H, H-C2), 7.42 (m, 2H, H-C3), 5.42 (dq, $^{2}J_{HH}$ = 1.6, $^{4}J_{HH}$ = 0.8 Hz, 1H, H^{a}-C6), 5.30 (app. p, $^{2}J_{HH}$, $^{4}J_{HH}$ = 1.5 Hz, 1H, H^{b}-C6), 2.18 (dd, $^{4}J_{HH}$ = 1.4 Hz, $^{4}J_{HH}$ = 0.8 Hz, 3H, H-C7).

^{13}C{^{1}H} NMR (126 MHz, $CDCl_3$): δ [ppm] = 167.4 (C8), 142.7(C1), 141.3 (C5), 134.5 (C10), 132.0 (C9), 130.9 (C4), 126.4 (C2), 126.4 (C3) 123.9 (C11), 113.5 (C6), 21.9 (C7).

ESI-MS: (*m/z*) requires: [$(C_{17}H_{13}NO_3Na)^+$] = 286.0839, (*m/z*) found: [$(C_{17}H_{13}NO_3Na)^+$] = 286.0839.

FT-IR ($\tilde{\nu}$ = cm^{-1})**:** 1705.0 (s), 1515.3 (w), 1368.4 (m), 1071.2 (w), 1017.6 (w), 882.3 (m), 836.2 (m), 790.9 (m), 715.6 (s), 678.0 (w).

2,2,2-Trifluoro-1-(4-(prop-1-en-2-yl)phenyl)ethan-1-one (S21)

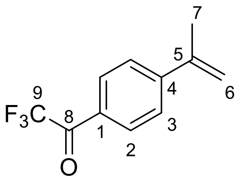

The reaction was performed in accordance with previous patent literature [19]

Magnesium turnings (547 mg, 22.5 mmol, 1.5 eq.) were suspended in dry THF (15 mL). 1-Bromo-4-(prop-1-en-2-yl)benzene **S6** (2.98 g, 15.0 mmol, 1.0 eq.) was added carefully at room temperature. The mixture turned brown and the reaction mixture started to reflux during the addition. After complete addition, the reaction was cooled to room temperature and stirred for 1h. The mixture was diluted with dry THF (15 mL) and subsequently cooled to -78°C. Ethyl 2,2,2-trifluoroacetate (3.20 g, 22.5 mmol, 1.5 eq.) was added dropwise and the reaction was stirred for 1.5 h. The reaction mixture was warmed to 0°C and quenched with 1M aqueous HCl. Et_2O was added, the layers were separated and the aqueous layer was extracted with Et_2O (3x). The combined organic layers were washed with brine (1x), dried over $MgSO_4$ and the crude mixture was purified by column chromatography (0-2% Et_2O in pentane) to yield compound **S21** as a yellow liquid (2.48 g, 11.6 mmol, 77%).

R*f* = 0.23 (1% Et_2O in pentane)

^{1}H NMR (599 MHz, $CDCl_3$): δ [ppm] = 8.04 (m, 2H, H-C2), 7.62 (m, 2H, H-C3), 5.55 (app. p, $^{2}J_{HH}$, $^{4}J_{HH}$ = 0.9 Hz, 1H, H^{a}-C6), 5.30 (app. p, $^{2}J_{HH}$, $^{4}J_{HH}$ = 1.4 Hz, 1H, H^{b}-C6), 2.19 (dd, $^{4}J_{HH}$ = 1.6 Hz, $^{4}J_{HH}$ = 0.8 Hz, 3H, H-C7).

^{19}F NMR (377 MHz, $CDCl_3$): δ [ppm] = -71.32 (s, 3F).

^{19}F{^{1}H} NMR (377 MHz, $CDCl_3$): δ [ppm] = -71.33 (s, 3F).

^{13}C{^{1}H} NMR (151 MHz, $CDCl_3$): δ [ppm] = 180.1 (q, $^{2}J_{CF}$ = 34.9 Hz, C8), 148.4 (s, C1), 142.1 (s, C5), 130.4 (q, $^{5}J_{CF}$ = 2.1 Hz, C3), 128.8 (s, C4), 126.2 (s, C2), 116.9 (q, $^{1}J_{CF}$ = 291.3 Hz, C9), 116.5 (s, C6), 21.5 (s, C7).

GC-EI-MS: Retention: 6.02 min (*m/z*) requires: [($C_{11}H_9OF_3$)] = 214.0600, (*m/z*) found: [($C_{11}H_9OF_3$)] = 214.0601.

FT-IR ($\tilde{\nu}$ = cm^{-1})**:** 3092.6 (w), 2980.0 (w), 1712.9 (s), 1628.1 (w), 1603.4 (m), 1557.3 (w), 1456.7 (w), 1416.9 (w), 1378.2 (w), 1337.3 (w), 1311.7 (w), 1285.6 (w), 1209.5 (m), 1196.5 (m), 1177.1 (s), 1139.0 (s), 1112.4 (s), 1006.1 (w), 939.0 (s), 903.1 (m), 854.3 (m), 778.7 (m), 721.5 (s).

1-(Prop-1-en-2-yl)-4-(3,3,3-trifluoroprop-1-en-2-yl)benzene (S22)

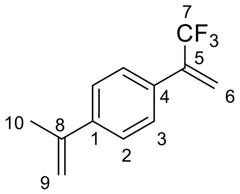

Compound **S22** was prepared according to General Procedure **A** using 2,2,2-trifluoro-1-(4-(prop-1-en-2-yl)phenyl)ethan-1-one **S21** (1.71 g, 8.00 mmol). The crude mixture was purified by column chromatography (100% pentane) to yield the title compound as a colorless oil (1.83 g, 8.5 mmol, 85%).

R*f* = 0.55 (pentane)

^{1}H NMR (599 MHz, $CDCl_3$): δ [ppm] = 7.50 (m, 2H, H-C2), 7.45 (m, 2H, H-C3), 5.96 (q, $^{4}J_{HF}$ = 1.3 Hz, 1H, H^{a}-C6), 5.80 (q, $^{4}J_{HF}$ = 1.7 Hz, 1H, H^{b}-C6), 5.43 (s, 1H, H^{a}-C9), 5.15 (app. p, $^{2}J_{HH}$, $^{4}J_{HH}$ = 1.4 Hz, 1H, H^{b}-C9), 2.17 (s, 3H, H-C10).

^{19}F NMR (377 MHz, $CDCl_3$): δ [ppm] = -64.66 (s, 3F).

^{19}F{^{1}H} NMR (377 MHz, $CDCl_3$): δ [ppm] = -64.67 (s, 3F).

^{13}C{^{1}H} NMR (151 MHz, $CDCl_3$): δ [ppm] = 142.6 (s, C1), 142.0 (s, C8), 138.8 (q, $^{2}J_{CF}$ = 34.9 Hz, C5), 132.7 (s, C4), 127.4 (q, $^{4}J_{CF}$ = 1.2 Hz, C3), 125.8 (s, C2), 123.5 (q, $^{1}J_{CF}$ = 274.0 Hz, C7), 120.1 (q, $^{3}J_{CF}$ = 5.8 Hz, C6), 113.4 (s, C9), 21.8 (s, C10).

GC-EI-MS: Retention: 6.05 min (*m/z*) requires: [($C_{12}H_{11}F_3$)] = 212.0807, (*m/z*) found: [($C_{12}H_{11}F_3$)] = 212.0807.

FT-IR ($\tilde{\nu}$ = cm^{-1})**:** 3090.0 (w), 2976.5 (w), 1795.6 (w), 1629.0 (w), 1555.1 (w), 1516.8 (w), 1452.0 (w), 1403.5 (w), 1376.5 (m), 1351.5 (w), 1318.1 (w), 1271.7 (w), 1190.9 (m), 1164.3 (s), 1115.0 (s), 1076.4 (s), 1016.8 (w), 941.3 (m), 894.4 (m), 842.7 (s), 763.7 (w), 727.0 (w), 697.0 (m).

Methyl (*E*)-3-(4-(prop-1-en-2-yl)phenyl)acrylate (S23)

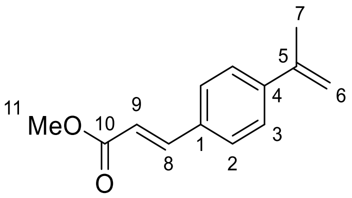

Compound **S23** was prepared according to General Procedure **A** using methyl (*E*)-3-(4-acetylphenyl)acrylate **S5** (1.02 g, 5.00 mmol). The crude mixture was purified by column chromatography (0-2% EtOAc in pentane) to afford the title compound as a white solid (365 mg, 1.80 mmol, 36%).

R*f* = 0.32 (2% EtOAc in pentane).

Melting point: 92-94°C

^{1}H NMR (500 MHz, $CDCl_3$) δ [ppm] 7.70 (d, $^{3}J_{HH}$ = 16.0 Hz, 1H, H-C8), 7.50 (app. s, 4H, H-C2, H-C3), 6.45 (d, $^{3}J_{HH}$ = 16.0 Hz, 1H, H-C9), 5.44 (m, 1H, H^a-C6), 5.16 (app. p, $^{2}J_{HH}$, $^{4}J_{HH}$ = 1.5 Hz, 1H, H^b-C6), 3.82 (s, 3H, H-C11), 2.17 (dd, $^{4}J_{HH}$= 1.5, $^{4}J_{HH}$ = 0.8 Hz, 3H, H-C7).

^{13}C NMR (126 MHz, $CDCl_3$) δ [ppm] 167.6 (C10), 144.5 (C8), 143.2 (C5), 142.6 (C4), 133.6 (C1), 128.2 (C2), 126.1 (C3), 117.6 (C9), 113.8 (C7), 51.8 (C11), 21.7 (C6).

ESI-MS: (*m/z*) requires: [($C_{13}H_{14}O_2$)] = 202.0989, (*m/z*) found: [($C_{13}H_{14}O_2$)] = 202.0988.

FT-IR ($\tilde{\nu}$ = cm^{-1})**:** 2948 (w),1712 (m), 1637 (m), 1600 (w), 1513 (w), 1438 (w), 1378 (w), 1328 (m), 1316 (m), 1274 (w), 1213 (w), 1194 (m), 1170 (m), 1127 (m), 1116 (w), 1009 (w), 982 (m), 958 (w), 934 (w), 901 (m), 832 (s), 765 (w), 741 (w), 715 (w), 687 (w), 676 (w).

1,4-Di(prop-1-en-2-yl)benzene (S24)

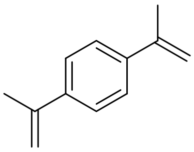

Compound **S24** was prepared according to modified General Procedure **A** using 1,1'-(1,4-phenylene)bis(ethan-1-one) (1.22 g, 7.50 mmol, 0.2M in THF). 3.0 Equivalents of $MePPh_3Br$ and *n*-BuLi were used. The crude mixture was purified by column chromatography (100% pentane) to yield the title compound as a white solid (628 mg, 4.00 mmol, 53%).

R*f* = 0.76 (pentane)

^{1}H NMR (400 MHz, $CDCl_3$): δ [ppm] = 7.45 (s, 4H), 5.40 (s, 2H), 5.11 – 5.07 (m, 2H), 2.17 (s, 6H).

GC-EI-MS: Retention: 6.58 min (*m/z*) requires: [($C_{12}H_{14}$)] = 158.11, (*m/z*) found: [($C_{12}H_{14}$)] = 158.11.

The analytical data are in good agreement with the literature [20].

Ethyl (*E*)-3-phenylbut-2-enoate (S25)

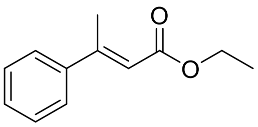

The reaction was performed in accordance with previous literature precedent. [21]

Triethyl phosphonoacetate (2.69 g, 12.0 mmol, 1.2 eq.) was added to a suspension of NaH (60 wt% in mineral oil, 480 mg, 12.0 mmol, 1.2 eq.) in dry THF (30 mL) at 0˚C. The mixture was stirred at 0˚C for 30 minutes. Acetophenone (1.20 g, 10.0 mmol, 1.0 eq.) was added dropwise at 0˚C. The reaction was allowed to warm to room temperature overnight. The reaction was quenched with water and was extracted with DCM (3x). The combined organic layers were dried over Na_2SO_4 and the solvent was removed under reduced pressure. The crude mixture was purified by column chromatography (0-2% Et_2O in pentane) to yield compound **S25** as a yellow oil (1.28 g, 6.71 mmol, 67%).

R*f* = 0.56 (80% DCM in pentane)

^{1}H NMR (400 MHz, $CDCl_3$): δ [ppm] = 7.50 – 7.45 (m, 2H), 7.41 – 7.34 (m, 3H), 6.14 (q, *J* = 1.3 Hz, 1H), 4.22 (q, *J* = 7.1 Hz, 2H), 2.58 (q, *J* = 1.3Hz, 3H), 1.22 (t, *J* = 7.1 Hz, 3H).

ESI-MS: (*m/z*) requires: $[(C_{12}H_{14}O_2Na)^+]$ = 213.0886, (*m/z*) found: $[(C_{12}H_{14}O_2Na)^+]$ = 213.0886.

The analytical data are in good agreement with the literature. [21]

3.4 α-(Bromomethyl)styrenes

General Procedure C:

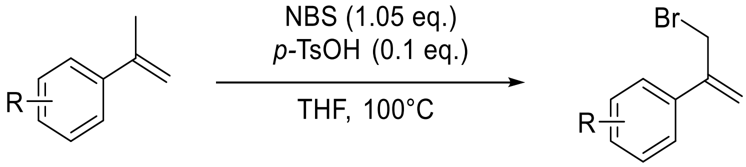

The reaction was performed according to *Loh et al.* [22].

A Schlenk flask was charged with the α-(methyl)styrene derivative and dry THF (0.33 M) under an argon atmosphere. NBS (1.05 eq.) followed by TsOH·H_2O (0.1 eq.) were added to the reaction mixture, which was then refluxed at 100°C for 4 to 8 h. The reaction mixture was cooled to room temperature, quenched with water and diluted with EtOAc or pentane. The layers were separated and the aqueous layer was extracted with EtOAc (3x) or pentane (3x). The combined organic layers were dried over $MgSO_4$ and the crude mixture was purified by column chromatography.

General Procedure D:

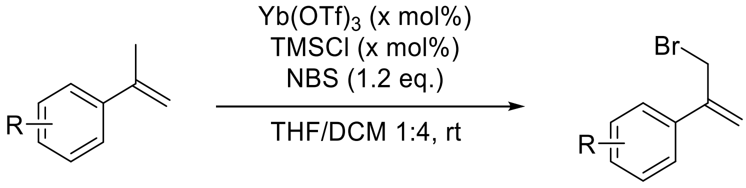

The reaction was performed according to *Overman et al.* [11].

A Schlenk flask was charged with the α-(methyl)styrene derivative (1.0 equiv.). A 4:1 mixture of dry DCM and dry THF (0.3 M) was added. The mixture was stirred at room temperature, while adding TMSCl (x mol%), followed by NBS (1.2 equiv.) and $Yb(OTf)_3$ (x mol%). After stirring for 1h at room temperature, the solvent was removed under reduced pressure. The crude mixture was purified by column chromatography.

1-Bromo-4-(3-bromoprop-1-en-2-yl)benzene (1a)

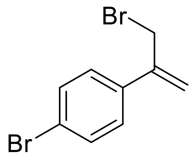

Compound **1a** was prepared according to General Procedure **C** using 1-bromo-4-(prop-1-en-2-yl)benzene **S6** (1.97 g, 10.0 mmol). The crude mixture was purified by column chromatography (100% pentane) to yield the title compound as a pale yellow oil (1.68 g, 6.10 mmol, 61%).

R*f* = 0.40 (pentane)

^{1}H NMR (400 MHz, $CDCl_3$): δ [ppm] = 7.50 (d, *J* = 8.6 Hz, 2H), 7.36 (d, *J* = 8.6 Hz, 2H), 5.55 (s, 1H), 5.51 (s, 1H), 4.35 (s, 2H).

GC-EI-MS: Retention: 7.48 min (*m/z*) requires: [($C_9H_8Br_2$)] = 275.90, (*m/z*) found: [($C_9H_8Br_2$)] = 275.92.

The analytical data are in good agreement with the literature [11].

(3-Bromoprop-1-en-2-yl)benzene (1b)

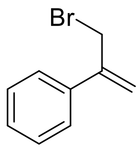

Compound **1b** was prepared according to *Suginome et. al*. [23].

A mixture of commercially available α-(methyl)styrene (570 mg, 5.00 mmol, 1.0 eq.) and NBS (1.02 g, 5.75 mmol, 1.15 eq.) in $CHCl_3$ (2 mL) was refluxed for 8 h. After cooling to room temperature, the solvent was removed under reduced pressure. Subsequently, pentane was added (100 mL) and the solid was filtered off. The solvent was removed under reduced pressure and the crude mixture was purified by column chromatography (100% pentane) to yield the title compound as a colorless oil (450 mg, 2.30 mmol, 46%).

R*f* = 0.51 (pentane)

^{1}H NMR (400 MHz, $CDCl_3$): δ [ppm] = 7.53 – 7.47 (m, 2H), 7.42 – 7.30 (m, 3H), 5.56 (s, 1H), 5.50 (s, 1H), 4.39 (s, 2H).

GC-EI-MS: Retention: 6.78 min (*m/z*) requires: [(C_9H_9Br)] = 195.99, (*m/z*) found: [(C_9H_9Br)] = 195.99.

The analytical data are in good agreement with the literature [23]

1-(3-Bromoprop-1-en-2-yl)-4-fluorobenzene (1c)

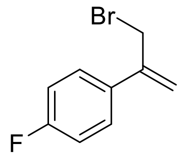

Compound **1c** was prepared according to General Procedure **C** using 1-fluoro-4-(prop-1-en-2-yl)benzene **S7** (545 mg, 4.00 mmol). The crude mixture was purified by column chromatography (100% pentane) to yield the title compound as a pale yellow oil (488 mg, 2.30 mmol, 57%).

$\mathbf{R}_{f}$ = 0.47 (pentane)

^{1}H NMR (400 MHz, $CDCl_3$): δ [ppm] = 7.50 – 7.44 (m, 2H), 7.11 – 7.01 (m, 2H), 5.50 (s, 1H), 5.48 (s, 1H), 4.36 (s, 2H).

^{19}F NMR (377 MHz, $CDCl_3$): δ [ppm] = -113.69 (tt, *J* = 8.6, 5.3 Hz, 1F).

GC-EI-MS: Retention: 6.20 min (*m/z*) requires: [(C_9H_8BrF)] = 213.98, (*m/z*) found: [(C_9H_8BrF)] = 214.03.

The analytical data are in good agreement with the literature [24].

1-(3-Bromoprop-1-en-2-yl)-4-chlorobenzene (1d)

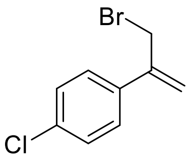

Compound **1d** was prepared according to General Procedure **C** using 1-chloro-4-(prop-1-en-2-yl)benzene **S8** (547 mg, 3.60 mmol). The crude mixture was purified by column chromatography (100% pentane) to yield the title compound as a pale yellow oil (602 mg, 2.60 mmol, 72%).

$\mathbf{R}_{f}$ = 0.38 (pentane)

^{1}H NMR (400 MHz, $CDCl_3$): δ [ppm] = 7.43 (d, *J* = 8.6 Hz, 2H), 7.35 (d, *J* = 8.5 Hz, 2H), 5.54 (s, 1H), 5.50 (s, 1H), 4.35 (s, 2H).

GC-EI-MS: Retention: 7.71 min (*m/z*) requires: [(C_9H_8BrCl)] = 229.95, (*m/z*) found: [(C_9H_8BrCl)] = 229.95.

The analytical data are in good agreement with the literature [11].

1-Bromo-3-(3-bromoprop-1-en-2-yl)benzene (1e)

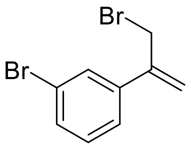

Compound **1e** was prepared according to General Procedure **C** using 1-bromo-3-(prop-1-en-2-yl)benzene **S9** (788 mg, 4.00 mmol). The crude mixture was purified by column chromatography (100% pentane) to yield the title compound as a pale yellow oil (514 mg, 1.90 mmol, 48%).

R*f* = 0.55 (pentane)

^{1}H NMR (400 MHz, $CDCl_3$): δ[ppm] = 7.63 (t, J = 1.9 Hz, 1H), 7.46 (ddd, J = 7.9, 1.9, 1.0 Hz, 1H), 7.41 (ddd, J = 7.9, 1.8, 1.0 Hz, 1H), 7.25 (t, J = 7.9 Hz, 1H), 5.55 (s, 1H), 5.53 (s, 1H), 4.34 (s, 2H).

GC-EI-MS: Retention: 7.41 min (*m/z*) requires: [($C_9H_8Br_2$)] = 275.90, (*m/z*) found: [($C_9H_8Br_2$)] = 275.92.

The analytical data are in good agreement with the literature [11].

1-Bromo-2-(3-bromoprop-1-en-2-yl)benzene (1f)

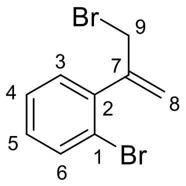

Compound **1f** was prepared according to General Procedure **C** using 1-bromo-2-(prop-1-en-2-yl)benzene **S10** (1.97 g, 10.0 mmol). The crude mixture was purified by column chromatography (100% pentane) to yield the title compound as a colorless oil (329 mg, 1.20 mmol, 12%).

R*f* = 0.56 (pentane)

^{1}H NMR (599 MHz, $CDCl_3$): δ[ppm] = 7.58 (dd, $^3J_{HH}$ = 7.9, $^4J_{HH}$ = 1.1 Hz, 1H, H-C6), 7.34 – 7.30 (m, 1H, H-C4), 7.29 (dd, $^3J_{HH}$ = 7.6, $^4J_{HH}$ = 2.0 Hz, 1H, H-C3), 7.20 (ddd, $^3J_{HH}$ = 8.1, 7.9, $^4J_{HH}$ = 2.0 Hz, 1H, H-C5), 5.65 (app. q, $^4J_{HH}$ = 1.0 Hz, 1H, H^a-C8), 5.22 (app. q, $^4J_{HH}$ = 0.7 Hz, 1H, H^b-C8), 4.36 (s, 2H, H-C9).

^{13}C NMR (151 MHz, $CDCl_3$): δ[ppm] = 145.8 (C7), 140.4 (C2), 132.9 (C6), 131.7 (C3), 129.6 (C5), 127.4 (C4), 122.0 (C1), 120.9 (C8), 35.6 (C9).

GC-EI-MS: Retention: 7.66 min (*m/z*) requires: [($C_9H_8Br_2$)] = 275.8967, (*m/z*) found: [($C_9H_8Br_2$)] = 275.8966.

FT-IR ($\tilde{\nu}$ = cm^{-1})**:** 3054.5 (w), 1634.2 (w), 1588.3 (w), 1560.6 (w), 1469.9 (m), 1424.0 (m), 1397.7 (w), 1309.6 (w), 1252.9 (w), 1208.4 (s), 1116.4 (w), 1038.2 (w), 1025.1 (s), 921.1 (s), 891.6 (w), 891.6 (s), 757.1 (s), 729.0 (s), 664.9 (s), 654.0 (s).

1-(3-Bromoprop-1-en-2-yl)-4-(*tert*-butyl)benzene (1g)

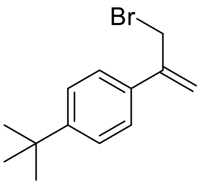

Compound **1g** was prepared according to General Procedure **C** using 1-(*tert*-butyl)-4-(prop-1-en-2-yl)benzene **S11** (697 mg, 4.00 mmol). The crude mixture was purified by column chromatography (100% pentane) to yield the title compound as a colorless oil (512 mg, 2.00 mmol, 50%).

R*f* = 0.38 (pentane)

^{1}H NMR (400 MHz, $CDCl_3$): δ[ppm] = 7.45 (d, *J* = 8.6 Hz, 2H), 7.40 (d, *J* = 8.6 Hz, 2H), 5.56 (s, 1H), 5.46 (s, 1H), 4.39 (s, 2H), 1.33 (s, 9H).

GC-EI-MS: Retention: 8.26 min (*m/z*) requires: [($C_{13}H_{17}Br$)] = 252.05, (*m/z*) found: [($C_{13}H_{17}Br$)] = 252.05.

The analytical data are in good agreement with the literature [14].

1-(3-Bromoprop-1-en-2-yl)-4-(trifluoromethyl)benzene (1h)

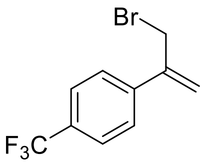

Compound **1h** was prepared according to General Procedure **D** using 1-(prop-1-en-2-yl)-4-(trifluoromethyl)benzene **S12** (559 mg, 3.00 mmol), TMSCl (16.3 mg, 5 mol%) and $Yb(OTf)_3$ (90.0 mg, 5 mol%). The crude mixture was purified by column chromatography (100% pentane) to yield the title compound as a colorless oil (402 mg, 2.00 mmol, 67%).

R*f* = 0.51 (pentane)

^{1}H NMR (400 MHz, $CDCl_3$): δ[ppm] = 7.66 – 7.57 (m, 4H), 5.62 (s, 1H), 5.59 (s, 1H), 4.38 (s, 2H).

^{19}F NMR (377 MHz, $CDCl_3$): δ[ppm] = -62.64 (s, 3F).

GC-EI-MS: Retention: 6.81 min (*m/z*) requires: [($C_{10}H_8BrF_3$)] = 263.98, (*m/z*) found: [($C_{10}H_8BrF_3$)] = 263.98.

The analytical data are in good agreement with the literature [25].

Methyl 4-(3-bromoprop-1-en-2-yl)benzoate (1i)

Compound **1i** was prepared according to a modified procedure of *Toste et al.* [26]

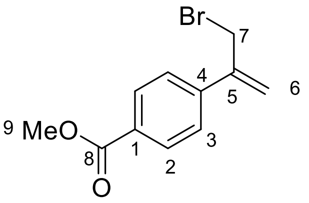

A solution of methyl 4-(3-hydroxyprop-1-en-2-yl)benzoate **S14** (211 mg, 1.10 mmol, 1.0 eq.) in dry DCM (5 mL) was cooled to 0°C. PPh_3 (346 mg, 1.32 mmol, 1.2 eq.) was added in one portion and the reaction mixture was stirred at 0°C for 15 minutes, followed by the addition of NBS (235 mg, 1.32 mmol, 1.2 eq.). The reaction was stirred for an additional hour at 0°C. The solvent was removed under reduced pressure and the crude mixture was purified by column chromatography (0-3% EtOAc in pentane) to yield compound **1i** as a white solid (248 mg, 0.97 mmol, 88%).

R*f* = 0.29 (5% EtOAc in pentane)

Melting Point: 48-50°C

^{1}H NMR (599 MHz, $CDCl_3$): δ [ppm] = 8.04 (m, 2H, H-C2), 7.56 (m, 2H, H-C3), 5.64 (s, 1H, H^a-C6), 5.59 (s, 1H, H^b-C6), 4.39 (s, 2H, H-C7), 3.93 (s, 3H, H-C9).

^{13}C{^{1}H} NMR (151 MHz, $CDCl_3$): δ [ppm] = 166.8 (C8), 143.7 (C4), 142.2 (C5), 130.0 (C1), 130.0 (C2), 126.3 (C3), 119.1 (C6), 52.3 (C9), 33.7 (C7).

ESI-MS: (*m/z*) requires: [($C_{11}H_{11}O_2BrNa$)$^+$] = 276.9835, (*m/z*) found: [($C_{11}H_{11}O_2BrNa$)$^+$] = 276.9836.

FT-IR ($\tilde{\nu}$ = cm^{-1})**:** 2948.8 (w), 1721.5 (s), 1608.8 (m), 1563.9 (w), 1427.0 (m), 1403.0 (w), 1317.7 (w), 1277.7 (s), 1219.3 (m), 1186.7 (s), 1143.5 (w), 1111.5 (s), 1018.5 (m), 967.5 (w), 936.0 (s), 880.9 (w), 857.6 (s), 829.8 (w), 779.6 (s), 746.1 (w), 732.4 (s), 700.8 (m).

1-(3-Bromoprop-1-en-2-yl)-4-nitrobenzene (1j)

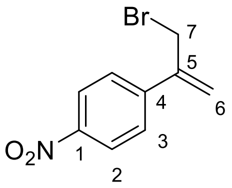

Compound **1j** was prepared according to General Procedure **D** using 1-nitro-4-(prop-1-en-2-yl)benzene **S15** (571 mg, 3.50 mmol), TMSCl (38.0 mg, 10 mol%) and $Yb(OTf)_3$ (217 mg, 10 mol%). The crude mixture was purified by column chromatography (5% EtOAc in pentane) to yield the title compound as a yellow solid (309 mg, 1.30 mmol, 37%).

R*f* = 0.43 (5% EtOAc in pentane)

Melting Point: 48-50°C

^{1}H NMR (500 MHz, $CDCl_3$): δ [ppm] = 8.25 – 8.21 (m, 2H, H-C3), 7.67 – 7.62 (m, 2H, H-C2), 5.69 (s, 1H, H^a-C6), 5.67 (s, 1H, H^b-C6), 4.38 (s, 2H, H-C7).

^{13}C{^{1}H} NMR (126 MHz, $CDCl_3$): δ [ppm] = 147.7 (C1), 144.2 (C4), 142.9 (C5), 127.2 (C2), 124.0 (C3), 120.5 (C6), 33.2 (C7).

GC-EI-MS: Retention: 8.79 min (*m/z*) requires: [($C_9H_8BrNO_2$)] = 240.9733, (*m/z*) found: [($C_9H_8BrNO_2$)] = 240.9734.

FT-IR ($\tilde{\nu}$ = cm^{-1})**:** 1595.2 (w), 1506.7 (s), 1343.8 (s), 1254.5 (w), 1216.1 (m), 1187.8 (m), 1108.5 (w), 1010.7 (w), 931.6 (m), 895.2 (w), 854.6 (s), 804.2 (m), 766.0 (w), 748.9 (m), 721.0 (s).

4-(3-Bromoprop-1-en-2-yl)phenyl 4-methylbenzenesulfonate (1k)

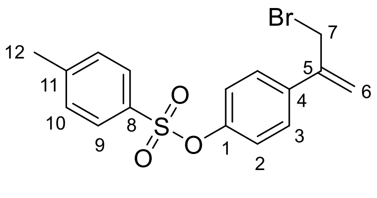

Compound **1k** was prepared according to General Procedure **C** using 4-(prop-1-en-2-yl)phenyl 4-methylbenzenesulfonate **S16** (1.15 g, 4.00 mmol). The crude mixture was purified by column chromatography (10% EtOAc in pentane) to yield the title compound as a pale yellow solid (922 mg, 2.50 mmol, 63%)

R*f* = 0.27 (10% EtOAc in pentane)

Melting Point: 34-36°C

^{1}H NMR (500 MHz, $CDCl_3$): δ [ppm] = 7.72 (m, 2H, H-C9), 7.39 (m, 2H, H-C2), 7.32 (m, 2H, H-C10), 6.98 (m, 2H, H-C3), 5.51 (s, 1H, H^a-C6), 5.50 (s, 1H, H^b-C6), 4.31 (s, 2H, H-C7), 2.45 (s, 3H, H-C12).

^{13}C{^{1}H} NMR (126 MHz, $CDCl_3$): δ [ppm] = 149.6 (C1), 145.6 (C11), 143.2 (C4), 136.7 (C5), 132.5 (C8), 129.9 (C10), 128.6 (C9), 127.5 (C2), 122.5 (C3), 118.2 (C6), 33.9 (C7), 21.9 (C12).

ESI-MS: (*m/z*) requires: $[(C_{16}H_{15}BrO_3SNa)^+]$ = 388.9818, (*m/z*) found: $[(C_{16}H_{15}BrO_3SNa)^+]$ = 388.9817.

FT-IR ($\tilde{\nu}$ = cm^{-1})**:** 3050.5 (w), 2970.9 (w), 1623.8 (w), 1595.7 (m), 1503.1 (m), 1447.1 (w), 1373.6 (s), 1309.5 (w), 1294.3 (w), 1212.1 (m), 1203.2 (m), 1155.5 (s), 1109.0 (s), 1091.0 (m), 1040.4 (w), 1015.0 (m), 944.8 (w), 910.5 (m), 864.0 (s), 848.0 (s), 815.3 (s), 762.7 (s), 743.1 (m), 705.0 (w), 682.9 (s).

2-Bromo-4-(3-bromoprop-1-en-2-yl)-1-fluorobenzene (1l)

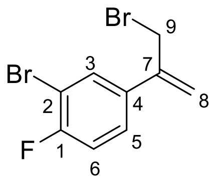

Compound **1l** was prepared according to General Procedure **C** using 2-bromo-1-fluoro-4-(prop-1-en-2-yl)benzene **S17** (860 mg, 4.00 mmol). The crude mixture was purified by column chromatography (100% pentane) to yield the title compound as a pale yellow oil (292 mg, 0.99 mmol, 25%).

R*f* = 0.48 (pentane)

^{1}H NMR (500 MHz, $CDCl_3$): δ [ppm] = 7.68 (dd, $^4J_{HF}$ = 6.5 Hz, $^4J_{HH}$ = 2.4 Hz, 1H, H-C3), 7.40 (ddd, $^3J_{HH}$ = 8.6 Hz, $^4J_{HF}$ = 4.6 Hz, $^3J_{HH}$ = 2.3 Hz, 1H, H-C5), 7.12 (t, $^3J_{HF}$, $^3J_{HH}$ = 8.4 Hz, 1H, H-C6), 5.51 (s, 1H, H^a-C8), 5.50 (s, 1H, H^b-C8), 4.31 (s, 2H, H-C9).

^{19}F NMR (376 MHz, $CDCl_3$): δ [ppm] = −107.89 (ddd, $^3J_{FH}$ = 8.2 Hz, $^4J_{FH}$ = 6.5 Hz, $^4J_{FH}$ = 4.5 Hz, 1F, F-C1).

^{19}F{^{1}H} NMR (376 MHz, $CDCl_3$): δ [ppm] = −107.89 (s, 1F, F-C1).

^{13}C{^{1}H} NMR (126 MHz, $CDCl_3$): δ [ppm] = 159.1 (d, $^1J_{CF}$ = 249.0 Hz, C1), 142.5 (s, C7), 135.5 (d, $^4J_{CF}$ = 4.0 Hz, C4), 131.5 (s, C3), 127.0 (d, $^3J_{CF}$ = 7.4 Hz, C5), 118.3 (s, C8), 116.6 (d, $^2J_{CF}$ = 22.4 Hz, C6), 109.4 (d, $^2J_{CF}$ = 21.2 Hz, C2), 33.8 (s, C9).

GC-EI-MS: Retention: 8.05 min (*m/z*) requires: [($C_9H_7Br_2F$)] = 293.8873, (*m/z*) found: [($C_9H_7Br_2F$)] = 293.8874.

FT-IR ($\tilde{\nu}$ = cm^{-1})**:** 3093.1 (w), 2972.1 (w), 1625.0 (w), 1594.5 (w), 1575.8 (w), 1494.9 (s), 1447.7 (w), 1378.0 (w), 1307.1 (m), 1265.7 (m), 1242.0 (m), 1209.9 (m), 1151.0 (w), 1129.2 (w), 1096.2 (w), 1045.8 (m), 947.8 (w), 915.3 (m), 880.2 (m), 850.3 (m), 820.0 (s), 729.3 (m), 714.3 (s), 663.4 (m).

1-((4-(3-Bromoprop-1-en-2-yl)phenyl)sulfonyl)piperidine (1m)

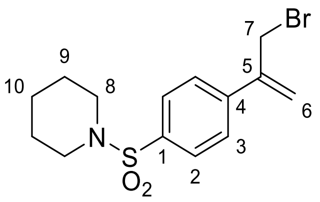

Compound **1m** was prepared according to General Procedure **D** using 1-((4-(prop-1-en-2-yl)phenyl)sulfonyl)piperidine **S18** (663 mg, 2.50 mmol), TMSCl (27.2 mg, 10 mol%) and Yb(OTf)$_3$ (155 mg, 10 mol%). The crude mixture was purified by column chromatography (0-6% EtOAc in pentane) to afford the title compound as a pale yellow solid (396 mg, 1.15 mmol, 46%).

R*f* = 0.17 (10% EtOAc in pentane).

Melting point: 95-97°C

^{1}H NMR (500 MHz, $CDCl_3$) δ [ppm] 7.75 (m, 2H, H-C2), 7.63 (m, 2H, H-C3), 5.66 (s, 1H, H^a-C6), 5.62 (s, 1H, H^b-C6), 4.37 (s, 2H, H-C7), 3.03 - 2.98 (m, 4H, H-C8), 1.68 – 1.61 (m, 4H, H-C9), 1.46 - 1.39 (m, 2H, H-C10).

^{13}C NMR (126 MHz, $CDCl_3$) δ [ppm] 143.1 (C5), 142.0 (C4), 136.1 (C1), 128.0 (C2), 126.8 (C3), 119.8 (C6), 47.0 (C8), 33.4 (C7), 25.3 (C9), 23.6 (C10).

ESI-MS: (*m/z*) requires: [($C_{14}H_{18}BrNO_2SNa$)$^+$] = 368.0114, (*m/z*) found: [($C_{14}H_{18}BrNO_2SNa$)$^+$] = 368.0111.

FT-IR ($\tilde{\nu}$ = cm^{-1})**:** 3055 (w), 2931 (w), 2847 (w), 1597 (w), 1562 (w), 1500 (w), 1474 (w), 1454 (w), 1444 (w), 1432 (w), 1398 (w), 1387 (w), 1361 (w), 1337 (m), 1326 (m), 1317 (m), 1283 (w), 1209 (m), 1161 (s), 1119 (w), 1098 (m), 1081 (m), 1052 (m), 1030 (w), 1016 (w), 946 (w), 920 (s), 898 (w), 861 (w), 842 (m), 829 (w), 777 (m), 715 (s).

2-(4-(3-Bromoprop-1-en-2-yl)phenyl)isoindoline-1,3-dione (1n)

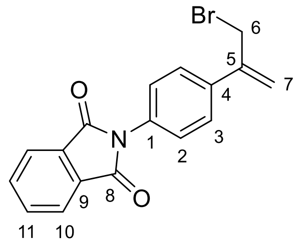

Compound **1n** was prepared according to General Procedure **C** using 2-(4-(prop-1-en-2-yl)phenyl)isoindoline-1,3-dione **S20** (327 mg, 1.24 mmol). The crude mixture was purified by column chromatography (80% DCM in pentane) to yield the title compound as a white solid (220 mg, 0.64 mmol, 52%).

R*f* = 0.59 (80% DCM in pentane)

Melting Point: 150-152°C

^{1}H NMR (599 MHz, $CDCl_3$): δ[ppm] = 7.97 (m, 2H, H-C10), 7.80 (m, 2H, H-C11), 7.63 (m, 2H, H-C2), 7.49 (m, 2H, H-C3), 5.61 (s, 1H, H^a-C7), 5.55 (s, 1H, H^b-C7), 4.39 (s, 2H, H-C6).

^{13}C{^{1}H} NMR (151 MHz, $CDCl_3$): δ[ppm] = 167.3 (s, C8), 143.6 (s, C5), 137.5 (s, C4), 134.6 (s, C11), 131.9 (s, C9), 131.8 (s, C1), 127.0 (s, C2), 126.5 (s, C3), 124.0 (s, C10), 118.3 (s, C7), 33.9 (s, C6).

ESI-MS: (*m/z*) requires: [$(C_{17}H_{12}BrNO_2Na)^+$] = 363.9944, (*m/z*) found: [$(C_{17}H_{12}BrNO_2Na)^+$] = 363.9946.

FT-IR ($\tilde{\nu}$ = cm^{-1})**:** 1710.5 (s), 1515.1 (m), 1373.2 (s), 1217.6 (w), 1071.7 (m), 924.1 (w), 882.4 (m), 834.2 (s), 788.5 (w), 715.3 (s), 694.5 (m), 680.9 (m).

1-(3-Bromoprop-1-en-2-yl)-4-(3,3,3-trifluoroprop-1-en-2-yl)benzene (3)

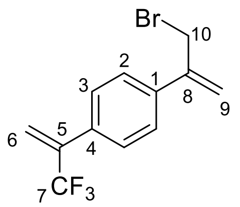

Compound **3** was prepared according to General Procedure **C** using 1-(prop-1-en-2-yl)-4-(3,3,3-trifluoroprop-1-en-2-yl)benzene **S22** (637 mg, 3.00 mmol). The crude mixture was purified by column chromatography (100% pentane) to yield the title compound as a colorless oil (583 mg, 2.00 mmol, 67%).

R*f* = 0.35 (pentane)

^{1}H NMR (500 MHz, $CDCl_3$): δ[ppm] = 7.53 (m, 2H, H-C3), 7.49 (m, 2H, H-C2), 5.98 (app. q, $^4J_{HF}$ = 1.4 Hz, 1H, H^a-C6), 5.82 (app. q, $^4J_{HF}$ = 1.7 Hz, 1H, H^b-C6), 5.61 (s, 1H, H^a-C9), 5.54 (s, 1H, H^b-C9), 4.39 (s, 2H, H-C10).

^{19}F NMR (376 MHz, $CDCl_3$): δ[ppm] = −64.64 (s, 3F, F-C7).

^{19}F{^{1}H} NMR (376 MHz, $CDCl_3$): δ[ppm] = −64.64 (s, 3F, F-C7).

^{13}C{^{1}H} NMR (126 MHz, $CDCl_3$): δ[ppm] = 143.6 (s, C8), 138.6 (q, $^{2}J_{CF}$ = 30.2 Hz, C5), 138.3 (s, C1), 133.5 (s, C4), 127.5 (q, $^{5}J_{CF}$ = 1.2 Hz, C2), 126.4 (s, C3), 123.4 (q, $^{1}J_{CF}$ = 274.2 Hz, C7), 120.6 (q, $^{3}J_{CF}$ = 5.8 Hz, C6), 118.0 (s, C9), 33.9 (s, C10).

GC-EI-MS: Retention: 7.70 min (*m/z*) requires: [($C_{12}H_{10}BrF_3$)] = 289.9913, (*m/z*) found: [($C_{12}H_{10}BrF_3$)] = 289.9910.

FT-IR ($\tilde{\nu}$ = cm^{-1})**:** 2927.9 (w), 1622.9 (w), 1622.9 (w), 1555.8 (w), 1516.8 (w), 1449.3 (w), 1401.5 (w), 1351,5 (m), 1323.2 (w), 1306.0 (w), 1272.6 (w), 1210.4 (w), 1191.4 (s), 1164.2 (s), 1116.5 (s), 1091.4 (s), 1074.5 (s), 1017.0 (w), 943.9 (m), 914.3 (m), 888.2 (w), 842.9 (s), 773.1 (w), 761.0 (w), 710.1 (w), 698.1 (m).

Methyl (*E*)-3-(4-(3-bromoprop-1-en-2-yl)phenyl)acrylate (6)

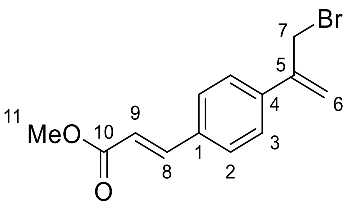

Compound **6** was prepared according to General Procedure **D** using methyl (*E*)-3-(4-(prop-1-en-2-yl)phenyl)acrylate **S23** (360 mg,1.78 mmol), TMSCl (9.80 mg, 5 mol%), and $Yb(OTf)_3$ (55.3 mg, 5 mol%). The crude mixture was purified by column chromatography (0-3.5% EtOAc in pentane) to afford the title compound as a white solid (195 mg, 0.69 mmol, 39%).

R*f* = 0.36 (5% EtOAc in pentane).

Melting point: 61-63°C

^{1}H NMR (500 MHz, $CDCl_3$) δ[ppm] 7.69 (d, $^{3}J_{HH}$ = 16.0 Hz, 1H, H-C8), 7.57 - 7.50 (m, 4H, H-C2, H-C3), 6.46 (d, $^{3}J_{HH}$ = 16.0 Hz, 1H, H-C9), 5.62 (s, 1H, H^{a}-C6), 5.54 (s, 1H, H^{b}-C6), 4.38 (s, 2H, H-C7), 3.81 (s, 3H, H-C11).

^{13}C NMR (126 MHz, $CDCl_3$) δ[ppm] 167.5 (C10), 144.2 (C8), 143.7 (C5), 139.5 (C4), 134.5 (C1), 128.4 (C2), 126.7 (C3), 118.3 (C9), 118.2 (C6), 51.9 (C11), 33.8 (C7).

ESI-MS: [ESI, orbitrap] (*m/z*) requires: [($C_{13}H_{13}BrO_2Na)^+$] = 302.9997, (*m/z*) found: [($C_{13}H_{13}BrO_2Na)^+$] = 302.9991.

FT-IR ($\tilde{\nu}$ = cm^{-1})**:** 3038 (w), 2955 (w), 1720 (s), 1637 (s), 1514 (m), 1449 (w), 1432 (m), 1416 (w), 1330 (m), 1314 (s), 1278 (m), 1209 (s), 1192 (s), 1168 (s), 1128 (m), 1088 (m), 1065 (w), 1035 (w), 1002 (m), 983 (s), 944 (m), 919 (s), 896 (m), 872 (m), 829 (s), 760 (m), 742 (m), 721 (m), 695 (m), 686 (m).

1,4-*Bis*(3-bromoprop-1-en-2-yl)benzene (9)

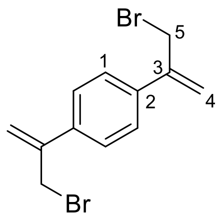

Compound **9** was prepared according to modified General Procedure **C** using 1,4-di(prop-1-en-2-yl)benzene **S24** (474 mg, 3.00 mmol, 0.17 M in THF). The stoichiometry of the reagents was adjusted: 2.1 eq. of NBS and 0.2 eq. of TsOH·H_2O were used. The crude mixture was purified by column chromatography (0-3% DCM in pentane) to yield the title compound as a white solid (372 mg, 1.17 mmol, 39%).

R*f* = 0.32 (5% DCM in pentane)

Melting Point: 84-86°C

^{1}H NMR (500 MHz, $CDCl_3$): δ[ppm] = 7.52 (s, 4H, H-C1), 5.61 (s, 2H, H^a-C4), 5.52 (s, 2H, H^b-C4), 4.39 (s, 4H, H-C5).

^{13}C{^{1}H} NMR (126 MHz, $CDCl_3$): δ[ppm] = 143.7 (C3), 137.5 (C2), 126.4 (C1), 117.6 (C4), 34.1 (C5).

GC-EI-MS: Retention: 9.27 min (*m/z*) requires: [($C_{12}H_{12}Br_2$)] = 315.9280, (*m/z*) found: [($C_{12}H_{12}Br_2$)] = 315.9281.

FT-IR ($\tilde{\nu}$ = cm^{-1})**:** 2955.0 (m), 2921.4 (m), 2853.2 (m), 1612.5 (w), 1515.9 (w), 1448.6 (w), 1399.0 (w), 1313.0 (w), 1207.8 (m), 1092.8 (w), 1010.9 (w), 941.4 (w), 916.3 (s), 890.8 (w), 839.7 (s), 718.9 (m), 660.1 (m).

1,3-*Bis*(3-bromoprop-1-en-2-yl)benzene (11)

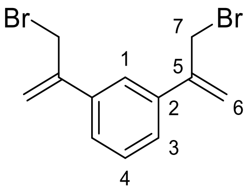

Compound **11** was prepared according to General Procedure **D** using commercially available 1,3-di(prop-1-en-2-yl)benzene (475 mg, 3.00 mmol), TMSCl (3.3 mg, 1 mol%), and Yb(OTf)$_3$ (19.0 mg, 1 mol%). The crude mixture was purified by column

chromatography (100% pentane) to afford the title compound as a yellow liquid (228 mg, 0.72 mmol, 24%)

R*f* = 0.17 (pentane).

^{1}H NMR (500 MHz, $CDCl_3$) δ [ppm] 7.62 (m, 1H, H-C1), 7.47 (dd, $^3J_{HF}$ = 7.3, $^4J_{HF}$ = 1.9 Hz, 2H, H-C3), 7.39 (m, 1H, H-C4), 5.60 (s, 2H, H^a-C6), 5.53 (s, 2H, H^b-C6), 4.40 (s, 4H, H-C7).

^{13}C NMR (126 MHz, $CDCl_3$) δ [ppm] 144.2 (C5), 138.1 (C2), 128.8 (C4), 126.1 (C3), 124.2 (C1), 117.8 (C6), 34.3 (C7).

ESI-MS: (*m/z*) requires: [($C_{12}H_{12}Br_2$)] = 315.9280, (*m/z*) found: [($C_{12}H_{12}Br_2$)] = 315.9280.

FT-IR ($\tilde{\nu}$ = cm^{-1})**:** 2970 (w), 1831 (w), 1684 (w), 1622 (w), 1596 (w), 1574 (w), 1485 (w), 1442 (m), 1394 (m), 1289 (w), 1209 (s), 1101 (w), 912 (s), 886 (m), 829 (w), 799 (s), 721 (s), 701 (m) cm^{-1}.

Ethyl (*E*)-4-bromo-3-phenylbut-2-enoate (13)

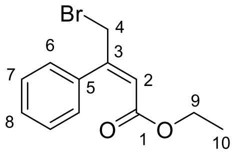

Ethyl (*E*)-3-phenylbut-2-enoate **S25** (190 mg, 1.00 mmol, 1.0 eq.) was dissolved in dry $CHCl_3$ (35 mL). NBS (213 mg, 1.20 mmol, 1.2 eq.) and DBPO (10.0 mg, 0.04 mmol, 4 mol%) were added at room temperature. The mixture was refluxed at 80˚C for 7 hours. The mixture was cooled to room temperature and the solvent was removed under reduced pressure. The crude mixture was purified by column chromatography (40-60% DCM in pentane) to yield compound **13** as a yellow oil (212 mg, 0.79 mmol, 79%).

R*f* = 0.59 (60% DCM in pentane)

^{1}H NMR (500 MHz, $CDCl_3$): δ [ppm] = 7.57 – 7.53 (m, 2H, H-C6), 7.44 – 7.40 (m, 2H, H-C7, H-C8), 6.21 (s, 1H, H-C2), 4.98 (s, 2H, H-C4), 4.27 (q, $^3J_{HH}$ = 7.2 Hz, 2H, H-C9), 1.34 (t, $^3J_{HH}$ = 7.1 Hz, 3H, H-C10).

^{13}C NMR (126 MHz, $CDCl_3$): δ [ppm] = 165.7 (C1), 153.3 (C3), 138.6 (C5), 129.9 (C7), 128.9 (C8), 126.7 (C6), 120.0 (C2), 60.7 (C9), 26.7 (C4), 14.4 (C10).

ESI-MS: (*m/z*) requires: $[(C_{12}H_{13}BrO_2Na)^+]$ = 290.9991, (*m/z*) found: : $[(C_{12}H_{13}BrO_2Na)^+]$ = 290.9992.

FT-IR ($\tilde{\nu}$ = cm^{-1})**:** 3059.3 (w), 2981.7 (w), 2902.8 (w), 1788.5 (w), 1765.8 (s), 1707.8 (s), 1623.2 (m), 1578.1 (w), 1495.5 (w), 1447.9 (m), 1389.6 (w), 1367.1 (m), 1342.8 (m), 1285.9 (s), 1222.1 (w), 1167.2 (s), 1139.8 (s), 1095.1 (w), 1049.9 (m), 996.7 (m), 936.9 (m), 879.3 (s), 790.2 (w), 767.4 (s), 693.9 (s).

Ethyl (*E*)-4-bromo-3-phenylbut-2-enoate (*E*-14) and ethyl (*Z*)-4-bromo-3-phenylbut-2-enoate (*Z*-14)

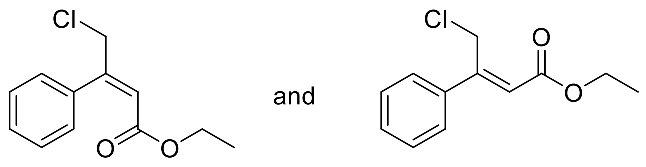

The reaction was performed in accordance with previous literature precedent. [21]

Triethyl phosphonoacetate (2.92 g, 13.0 mmol, 1.3 eq.) was added to a suspension of NaH (60 wt% in mineral oil, 600 mg, 15.0 mmol, 1.5 eq.) in dry THF (25 mL) at 0˚C. The mixture was stirred at 0˚C for 30 minutes. 2-Chloroacetophenone (1.55 g, 10.0 mmol, 1.0 eq.) was dissolved in dry THF (5 mL) and added at 0˚C. The reaction was allowed to warm to room temperature overnight. The reaction was quenched with brine and extracted with Et_2O (3x). The combined organic layers were dried over Na_2SO_4 and the solvent was removed under reduced pressure. The crude mixture was purified by column chromatography (0-5% Et_2O in pentane and 40-60% DCM in pentane) to yield compound ***E*-14** (700 mg, 3.11 mmol, 31%) and compound ***Z*-14** (212 mg, 0.95 mmol, 10%) as yellow oils.

***E*-14:**

R_f = 0.48 (60% DCM in pentane)

^{1}H NMR (400 MHz, $CDCl_3$): δ [ppm] = 7.59 – 7.49 (m, 2H), 7.48 – 7.36 (m, 2H), 6.24 (s, 1H), 5.09 (s, 2H), 4.26 (q, *J* = 7.2 Hz, 2H), 1.34 (t, *J* = 7.1 Hz, 3H).

^{1}H NMR (400 MHz, DMSO-d6): δ [ppm] = 7.69 – 7.64 (m, 2H), 7.48 – 7.43 (m, 2H), 6.32 (s, 1H), 5.22 (s, 2H), 4.20 (q, *J* = 7.1 Hz, 2H), 1.26 (t, *J* = 7.1 Hz, 3H).

ESI-MS: (*m/z*) requires: $[(C_{12}H_{13}O_2ClNa)^+]$ = 247.0496, (*m/z*) found: : $[(C_{12}H_{13}O_2ClNa)^+]$ = 247.0494.

The analytical data are in good agreement with the literature. [27]

***Z*-14:**

R*f* = 0.28 (60% DCM in pentane)

^{1}H NMR (400 MHz, $CDCl_3$): δ [ppm] = 7.36 – 7.29 (m, 5H), 6.48 (s, 1H), 4.13 (q, *J* = 7.1 Hz, 2H), 3.70 (s, 2H), 1.19 (t, *J* = 7.1 Hz, 3H).

ESI-MS: (*m/z*) requires: [($C_{12}H_{13}O_2ClNa)^+$] = 247.0496, (*m/z*) found: : [($C_{12}H_{13}O_2ClNa)^+$] = 247.0493.

.

3.5 Preparation of the *gem*-Difluorinated Products

General procedure E for the *gem*-difluorination

A Teflon® vial was equipped with a 1.0 cm stirring bar. The α-(bromomethyl)styrene derivative (0.50 mmol, 1.0 eq.) was added to the vial, followed by the addition of *p*-iodotoluene (22.0 mg, 0.10 mmol, 0.2 eq.) and $CHCl_3$ (1.25 mL, 0.2 M). $NEt_3{\cdot}3HF$ (0.53 mL) followed by Olah´s reagent (Py·9.23HF, 0.72 mL) were added via syringe to the reaction vessel. Subsequently, Selectfluor® (266 mg, 0.75 mmol, 1.5 eq.) was added in one portion and the vial was capped and stirred at room temperature (350 rpm). The reaction mixture was pre-quenched after 24 h by adding DCM (1 mL) and the dropwise addition of a saturated aqueous solution of $NaHCO_3$ (1 mL). The mixture was poured into an Erlenmeyer flask, which was previously charged with a saturated aqueous solution of $NaHCO_3$ (250 mL). The reaction vessel and the cap were washed with DCM in order to remove all remaining HF. The aqueous layer was extracted with DCM (3x, 200 mL in total), the organic layers were combined and dried over $MgSO_4$. The solvent was removed under reduced pressure and the crude mixture was purified by column chromatography.

1-Bromo-4-(3-bromo-2,2-difluoropropyl)benzene (2a)

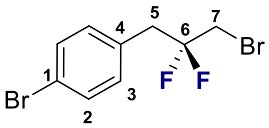

Compound **2a** was prepared according to General Procedure **E** using 1-bromo-4-(3-bromoprop-1-en-2-yl)benzene **1a** (138 mg, 0.50 mmol). The crude mixture was purified by column chromatography (100% pentane) to yield the title compound as a white solid (125 mg, 0.40 mmol, 80%).

4 mmol-scale: Compound **2a** was prepared according to General Procedure **E** using 1-bromo-4-(3-bromoprop-1-en-2-yl)benzene **1a** (1.10 g, 4.00 mmol). The stoichiometry of the reagents and solvents was adapted to the reaction scale (*p*TolI 174 mg, Selectfluor® 2.13 g, amine:HF 10 mL, and $CHCl_3$ 10 mL).The crude mixture was purified by column chromatography (100% pentane) to yield the title compound as a white solid (989 mg, 3.15 mmol, 79%).

R$_f$ = 0.26 (pentane)

Melting Point: 97-99˚C

^{1}H NMR (500 MHz, CDCl$_3$): δ [ppm] = 7.50 – 7.46 (m, 2H, H-C3), 7.21 (d, $^3J_{HH}$ = 8.8 Hz, 2H, H-C2), 3.39 (t, $^3J_{HF}$ = 12.6 Hz, 2H, H-C7), 3.33 (t, $^3J_{HF}$ = 15.6 Hz, 2H, H-C5).

^{19}F NMR (376 MHz, CDCl$_3$): δ [ppm] = −97.17 (tt, $^3J_{FH}$ = 15.6 Hz, $^3J_{FH}$ = 12.5 Hz, 2F, F-C6).

^{19}F{^{1}H} NMR (376 MHz, CDCl$_3$): δ [ppm] = −97.16 (s, 2F, F-C6).

^{13}C{^{1}H} NMR (126 MHz, CDCl$_3$): δ [ppm] = 132.1 (t, $^5J_{CF}$ = 1.0 Hz, C2), 132.0 (s, C3), 131.3 (t, $^3J_{CF}$ = 4.8 Hz, C4), 122.2 (s, C1), 120.6 (t, $^1J_{CF}$ = 244.5 Hz, C6), 40.2 (t, $^2J_{CF}$ = 25.2 Hz, C5), 30.5 (t, $^2J_{CF}$ = 33.7 Hz, C7).

GC-EI-MS: Retention: 7.82 min (*m/z*) requires: [($C_9H_8Br_2F_2$)] = 313.8935, (*m/z*) found: [($C_9H_8Br_2F_2$)] = 313.8935.

FT-IR ($\tilde{\nu}$ = cm^{-1})**:** 1486.5 (m), 1246.8 (w), 1187.5 (m), 1095.5 (m), 1068.2 (m), 1006.8 (s), 891.4 (m), 852.9 (w), 788.5 (s), 719.8 (m), 686.6 (s).

1-(3-Bromo-2,2-difluoropropyl)benzene (2b)

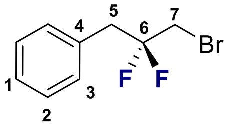

Compound **2b** was prepared according to General Procedure **E** using (3-bromoprop-1-en-2-yl)benzene **1b** (99.0 mg, 0.50 mmol). The crude mixture was purified by column chromatography (100% pentane) to yield the title compound as a white solid (74.0 mg, 0.32 mmol, 63%).

R$_f$ = 0.34 (pentane)

Melting Point: 56-58˚C

^{1}H NMR (500 MHz, CDCl$_3$): δ [ppm] = 7.38 – 7.30 (m, 5H, H-C1, H-C2, H-C3), 3.40 (t, $^3J_{HF}$ = 12.7 Hz, 2H, H-C7), 3.36 (t, $^3J_{HF}$ = 15.6 Hz, 2H, H-C5).

^{19}F NMR (376 MHz, CDCl$_3$): δ [ppm] = −97.00 (tt, $^3J_{FH}$ = 15.6 Hz, $^3J_{FH}$ = 12.7 Hz, 2F, F-C6).

^{19}F{^{1}H} NMR (376 MHz, CDCl$_3$): δ [ppm] = −97.00 (s, 2F, F-C6).

^{13}**C{^{1}H} NMR** (126 MHz, $CDCl_3$): δ[ppm] = 132.4 (t, $^{3}J_{CF}$ = 5.0 Hz, C4), 130.4 (t, $^{5}J_{CF}$ = 0.9 Hz, C2), 128.8 (s, C3), 127.9 (s, C1), 120.9 (t, $^{1}J_{CF}$ = 244.4 Hz, C6), 40.9 (t, $^{2}J_{CF}$ = 25.0 Hz, C5), 30.7 (t, $^{2}J_{CF}$ = 33.4 Hz, C7).

GC-EI-MS: Retention: 6.44 min (*m/z*) requires: [($C_9H_9BrF_2$)] = 233.9850, (*m/z*) found: [($C_9H_9BrF_2$)] = 233.9851.

FT-IR ($\tilde{\nu}$ = cm^{-1})**:** 3033.6 (w), 2940.9 (w), 1606.8 (w), 1495.7 (m), 1454.8 (w), 1429.5 (w), 1419.1 (m), 1343.3 (m), 1275.3 (w), 1253.2 (m), 1188.9 (s), 1167.1 (w), 1094.6 (s), 1079.3 (m), 1032.8 (w), 1006.3 (s), 889.7 (m), 851.2 (s), 810.0 (s), 758.7 (m), 750.8 (s), 700.2 (s), 654.4 (s).

1-(3-Bromo-2,2-difluoropropyl)-4-fluorobenzene (2c)

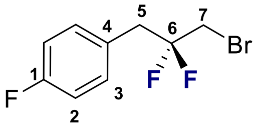

Compound **2c** was prepared according to General Procedure **E** using 1-(3-bromoprop-1-en-2-yl)-4-fluorobenzene **1c** (107 mg, 0.50 mmol). The crude mixture was purified by column chromatography (100% pentane) to yield the title compound as a white solid (90.0 mg, 0.36 mmol, 71%).

R*f* = 0.38 (pentane)

Melting Point: 74-76°C

^{1}**H NMR** (500 MHz, $CDCl_3$): δ[ppm] = 7.30 (dd, $^{3}J_{HH}$ = 8.4 Hz, $^{4}J_{HF}$ 5.4 Hz, 2H, H-C3), 7.04 (t, $^{3}J_{HH}$, $^{3}J_{HF}$ = 8.7 Hz, 2H, H-C2), 3.39 (t, $^{3}J_{HF}$ = 12.6 Hz, 2H, H-C7), 3.34 (t, $^{3}J_{HF}$ = 15.7 Hz, 2H, H-C5).

^{19}**F NMR** (376 MHz, $CDCl_3$): δ[ppm] = −97.39 (tt, $^{3}J_{FH}$ = 15.7 Hz, $^{3}J_{FH}$ = 12.5 Hz, 2F, F-C6), −114.54 (tt, $^{3}J_{FH}$ = 8.7 Hz, $^{4}J_{FH}$ = 5.4 Hz, 1F, F-C1).

^{19}**F{^{1}H} NMR** (376 MHz, $CDCl_3$): δ[ppm] = −97.39 (s, 2F, F-C6), −114.55 (s, 1F, F-C1).

^{13}**C{^{1}H} NMR** (151 MHz, $CDCl_3$): δ[ppm] = 162.6 (d, $^{1}J_{CF}$ = 246.6 Hz, C1), 132.0 (d, $^{3}J_{CF}$ = 8.1 Hz, C3), 128.1 (td, $^{3}J_{CF}$ = 4.7 Hz, $^{4}J_{CF}$ = 3.2 Hz, C4), 120.8 (td, $^{1}J_{CF}$ = 244.3 Hz, $^{6}J_{CF}$ = 1.5 Hz, C6), 115.8 (d, $^{3}J_{CF}$ = 21.4 Hz, C2), 40.0 (t, $^{2}J_{CF}$ = 25.2 Hz, C5), 30.5 (t, $^{2}J_{CF}$ = 33.7 Hz, C7).

GC-EI-MS: Retention: 6.46 min (*m/z*) requires: [($C_9H_8BrF_3$)] = 251.9756, (*m/z*) found: [($C_9H_8BrF_3$)] = 251.9757.

FT-IR ($\tilde{\nu}$ = cm^{-1})**:** 1612.0 (w), 1507.2 (s), 1419.1 (w), 1348.4 (w), 1250.0 (m), 1222.6 (s), 1214.7 (s), 1188.6 (s), 1158.1 (m), 1095.1 (s), 1009.2 (s), 892.0 (s), 863.4 (w), 851.1 (m), 796.3 (s), 775.3 (s), 677.9 (w).

1-(3-Bromo-2,2-difluoropropyl)-4-chlorobenzene (2d)

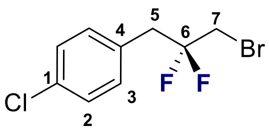

Compound **2d** was prepared according to General Procedure **E** using 1-(3-bromoprop-1-en-2-yl)-4-chlorobenzene **1d** (116 mg, 0.50 mmol). The crude mixture was purified by column chromatography (100% pentane) to yield the title compound as a white solid (107 mg, 0.40 mmol, 79%).

R*f* = 0.38 (pentane)

Melting Point: 89-91˚C

^{1}H NMR (500 MHz, $CDCl_3$): δ [ppm] = 7.34 – 7.31 (m, 2H, H-C3), 7.26 (d, $^3J_{HH}$ = 8.4 Hz, 2H, H-C2), 3.39 (t, $^3J_{HF}$ = 12.5 Hz, 2H, H-C7), 3.34 (t, $^3J_{HF}$ = 15.6 Hz, 2H, H-C5).

^{19}F NMR (376 MHz, $CDCl_3$): δ [ppm] = −97.20 (tt, $^3J_{FH}$ = 15.7 Hz, $^3J_{FH}$ = 12.5 Hz, 2F, F-C6).

^{19}F{^{1}H} NMR (376 MHz, $CDCl_3$): δ [ppm] = −97.20 (s, 2F, F-C6).

^{13}C{^{1}H} NMR (126 MHz, $CDCl_3$): δ [ppm] = 134.1 (s, C1), 131.7 (t, $^5J_{CF}$ = 0.8 Hz, C2), 130.8 (t, $^3J_{CF}$ = 4.8 Hz, C4), 129.0 (s, C3), 120.7 (t, $^1J_{CF}$ = 244.5 Hz, C6), 40.1 (t, $^2J_{CF}$ = 25.2 Hz, C5), 30.5 (t, $^2J_{CF}$ = 33.7 Hz, C7).

GC-EI-MS: Retention: 7.39 min (*m/z*) requires: [($C_9H_8BrClF_2$)] = 269.9438, (*m/z*) found: [($C_9H_8BrClF_2$)] = 269.9438.

FT-IR ($\tilde{\nu}$ = cm^{-1})**:** 1491.6 (w), 1420.5 (w), 1347.2 (w), 1248.5 (w), 1187.7 (m), 1095.7 (m), 1007.3 (s), 891.8 (m), 847.9 (w), 791.5 (s), 725.4 (m), 694.6 (w), 666.8 (w).

1-Bromo-3-(3-bromo-2,2-difluoropropyl)benzene (2e)

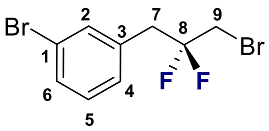

Compound **2e** was prepared according to General Procedure **E** using 1-bromo-3-(3-bromoprop-1-en-2-yl)benzene **1e** (138 mg, 0.50 mmol). The crude mixture was purified by column chromatography (100% pentane) to yield the title compound as a white solid (110 mg, 0.35 mmol, 70%).

R*f* = 0.33 (pentane)

Melting Point: 38-40°C

^{1}H NMR (500 MHz, $CDCl_3$): δ [ppm] = 7.48 (broad s, 1H, H-C2), 7.46 (dt, $^3J_{HH}$ = 7.8, $^4J_{HH}$ = 1.6 Hz, 1H, H-C6), 7.27 (d, $^3J_{HH}$ = 7.8 Hz, 1H, H-C4), 7.22 (t, $^3J_{HH}$ = 7.7 Hz, 1H, H-C5), 3.41 (t, $^3J_{HF}$ = 12.6 Hz, 2H, H-C9), 3.34 (t, $^3J_{HF}$ = 15.6 Hz, 2H, H-C7).

^{19}F NMR (376 MHz, $CDCl_3$): δ [ppm] = −97.00 (tt, $^3J_{FH}$ = 15.5 Hz, $^3J_{FH}$ = 12.5 Hz, 2F, F-C8).

^{19}F{^{1}H} NMR (376 MHz, $CDCl_3$): δ [ppm] = −97.00 (s, 2F, F-C8).

^{13}C{^{1}H} NMR (126 MHz, $CDCl_3$): δ [ppm] = 134.5 (t, $^3J_{CF}$ = 4.7 Hz, C3), 133.4 (t, $^5J_{CF}$ = 0.8 Hz, C2), 131.2 (s, C6), 130.4 (s, C5), 129.1 (s, C4), 122.8 (s, C1), 120.6 (t, $^1J_{CF}$ = 244.7 Hz, C8), 40.4 (t, $^2J_{CF}$ = 25.3 Hz, C7), 30.5 (t, $^2J_{CF}$ = 33.5 Hz, C9).

GC-EI-MS: Retention: 7.74 min (*m/z*) requires: [($C_9H_8Br_2F_2$)] = 313.8935, (*m/z*) found: [($C_9H_8Br_2F_2$)] = 313.8935.

FT-IR ($\tilde{\nu}$ = cm^{-1})**:** 3056.7 (w), 1593.4 (w), 1568.5 (w), 1475.1 (m), 1423.2 (m), 1348.9 (w), 1299.1 (w), 1271.0 (m), 1256.7 (m), 1188.4 (m), 1096.2 (m), 1071.3 (m), 1008.5 (s), 896.1 (m), 854.9 (m), 813.1 (m), 776.2 (s), 705.2 (s), 691.5 (m), 670.1 (w), 652.6 (s).

1-Bromo-2-(3-bromo-2,2-difluoropropyl)benzene (2f)

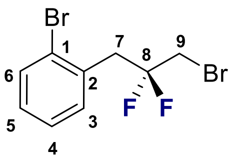

Compound **2f** was prepared according to General Procedure **E** using 1-bromo-2-(3-bromoprop-1-en-2-yl)benzene **1f** (138 mg, 0.50 mmol). The crude mixture was purified by column

chromatography (100% pentane) to yield the title compound as a white solid (97.0 mg, 0.31 mmol, 62%).

R*f* = 0.46 (pentane)

Melting Point: 68-70°C

^{1}H NMR (500 MHz, $CDCl_3$): δ[ppm] = 7.62 (dd, $^3J_{HH}$ = 8.1, $^4J_{HH}$ = 1.2 Hz, 1H, H-C3), 7.43 (dd, $^3J_{HH}$ = 7.7, $^4J_{HH}$ = 1.5 Hz, 1H, H-C6), 7.32 (td, $^3J_{HH}$ = 7.5, $^4J_{HH}$ = 1.2 Hz, 1H, H-C5), 7.20 (td, $^3J_{HH}$ = 7.5, $^4J_{HH}$ = 1.2 Hz,1H, H-C4), 3.62 (t, $^3J_{HF}$ = 15.7 Hz, 2H, H-C7), 3.58 (t, $^3J_{HF}$ = 13.6 Hz, 2H, H-C9).

^{19}F NMR (376 MHz, $CDCl_3$): δ[ppm] = −96.91 (tt, $^3J_{FH}$ = 15.7 Hz, $^3J_{FH}$ = 13.6 Hz, 2F, F-C6).

^{19}F{^{1}H} NMR (376 MHz, $CDCl_3$): δ[ppm] = −96.90 (s, 2F, F-C8).

^{13}C{^{1}H} NMR (126 MHz, $CDCl_3$): δ[ppm] = 133.4 (s, C3), 132.4 (s, C6), 132.3 (t, $^3J_{CF}$ = 4.0 Hz, C2), 129.6 (s, C5), 127.8 (s, C4), 125.9 (s, C1), 120.1 (t, $^1J_{CF}$ = 245.2 Hz, C8), 40.7 (t, $^2J_{CF}$ = 25.4 Hz, C7), 31.6 (t, $^2J_{CF}$ = 31.5 Hz, C9).

GC-EI-MS: Retention: 7.79 min (*m/z*) requires: [($C_9H_8Br_2F_2$)] = 313.8935, (*m/z*) found: [($C_9H_8Br_2F_2$)] = 313.8935.

FT-IR ($\tilde{\nu}$ = cm^{-1})**:** 2948.7 (w), 1721.1 (s), 1609.0 (w), 1567.8 (w), 1470.0 (m), 1436.5 (m), 1346.5 (w), 1276.1 (s), 1192.2 (m), 1121.0 (m), 1095.8 (s), 1029.5 (s), 1004.8 (s), 901.8 (w), 888.8 (m), 854.0 (m), 810.2 (m), 782.6 (m), 756.8 (s), 724.6 (s), 688.1 (w), 664.5 (s).

1-(3-Bromo-2,2-difluoropropyl)-4-(*tert*-butyl)benzene (2g)

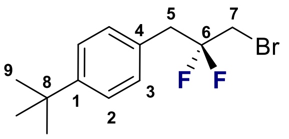

Compound **2g** was prepared according to modified General Procedure **E** using an amine:HF ratio of 1:4.5 (NEt_3·3HF 0.86 mL, Olah´s reagent 0.39 mL) and 1-(3-bromoprop-1-en-2-yl)-4-(*tert*-butyl)benzene **1g** (126 mg, 0.50 mmol). The crude mixture was purified by column chromatography (pentane) to yield the title compound as a colorless oil (99.0 mg, 0.34 mmol, 68%).

R*f* = 0.36 (pentane)

^{1}**H NMR** (500 MHz, $CDCl_3$): δ [ppm] = 7.36 (m, 2H, H-C2), 7.25 (m, 2H, H-C3), 3.41 (t, $^{3}J_{HF}$ = 12.8 Hz, 2H, H-C7), 3.34 (t, $^{3}J_{HF}$ = 15.6 Hz, 2H, H-C5), 1.32 (s, 9H, H-C9).

^{19}**F NMR** (376 MHz, $CDCl_3$): δ [ppm] = −97.04 (tt, $^{3}J_{FH}$ = 15.6 Hz, $^{3}J_{FH}$ = 12.8 Hz, 2F, F-C6).

^{19}**F{^{1}H} NMR** (376 MHz, $CDCl_3$): δ [ppm] = −97.04 (s, 2F, F-C6).

^{13}**C{^{1}H} NMR** (126 MHz, $CDCl_3$): δ [ppm] = 150.9 (s, C1), 130.0 (t, $^{4}J_{CF}$ = 0.9 Hz, C3), 129.3 (t, $^{3}J_{CF}$ = 5.0 Hz, C4), 125.8 (s, C2), 121.0 (t, $^{1}J_{CF}$ = 244.2 Hz, C6), 40.4 (t, $^{2}J_{CF}$ = 25.2 Hz, C5), 34.7 (s, C8), 31.5 (s, C9), 30.8 (t, $^{2}J_{CF}$ = 33.3 Hz, C7).

GC-EI-MS: Retention: 7.91 min (*m/z*) requires: [($C_{13}H_{17}BrF_2$)] = 290.0476, (*m/z*) found: [($C_{13}H_{17}BrF_2$)] = 290.0477.

FT-IR ($\tilde{\nu}$ = cm^{-1})**:** 2962.7 (s), 1515.7 (m), 1465.1 (w), 1422.4 (m), 1364.4 (m), 1343.2 (w), 1302.6 (m), 1269.8 (m), 1253.0 (m), 1203.8 (m), 1140.3 (s), 1122.5 (m), 1109.8 (w), 1068.7 (s), 1029.6 (s), 894.8 (w), 856.1 (m), 838.6 (s), 791.6 (s), 740.3 (w), 698.4 (s), 681.8 (w).

1-(3-Bromo-2,2-difluoropropyl)-4-(trifluoromethyl)benzene (2h)

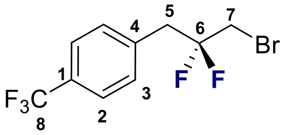

Compound **2h** was prepared according to General Procedure **E** using 1-(3-bromoprop-1-en-2-yl)-4-(trifluoromethyl)benzene **1h** (133 mg, 0.50 mmol). The crude mixture was purified by column chromatography (100% pentane) to yield the title compound as a white solid (111 mg, 0.37 mmol, 73%).

4 mmol-scale: Compound **2h** was prepared according to General Procedure **E** using 1-(3-bromoprop-1-en-2-yl)-4-(trifluoromethyl)benzene **1h** (1.06 g, 4.00 mmol). The stoichiometry of the reagents and solvents was adapted to the reaction scale (*p*TolI 174 mg, Selectfluor® 2.13 g, amine:HF 10 mL, and $CHCl_3$ 10 mL).The crude mixture was purified by column chromatography (100% pentane) to yield the title compound as a white solid (883 mg, 2.91 mmol, 73%).

R*f* = 0.40 (pentane)

Melting Point: 52-54˚C

^{1}**H NMR** (599 MHz, $CDCl_3$): δ [ppm] = 7.62 (d, $^{3}J_{HH}$ = 7.9 Hz, 2H, H-C), 7.46 (d, $^{3}J_{HH}$ = 7.9 Hz, 2H, H-C), 3.44 (t, $^{3}J_{HF}$ = 15.7 Hz, 2H, H-C5), 3.41 (t, $^{3}J_{HF}$ = 12.6 Hz, 2H, H-C7).

^{19}**F NMR** (376 MHz, $CDCl_3$): δ [ppm] = −62.67 (s, 3F, F-C8), −97.00 (tt, $^{3}J_{FH}$ = 15.9 Hz, $^{3}J_{FH}$ = 12.5 Hz, 2F, F-C6).

^{19}**F{^{1}H} NMR** (376 MHz, $CDCl_3$): δ [ppm] = −62.67 (s, 3F, F-C8), −97.00 (s, 2F, F-C6).

^{13}**C{^{1}H} NMR** (151 MHz, $CDCl_3$): δ [ppm] = 136.3 (broad s, C4), 130.9 (s, C3), 130.4 (q, $^{2}J_{CF}$ = 32.4 Hz, C1), 125.8 (q, $^{3}J_{CF}$ = 3.8 Hz, C2), 124.2 (q, $^{1}J_{CF}$ = 272.0 Hz, C8), 120.6 (t, $^{1}J_{CF}$ = 244.9 Hz, C6), 40.6 (t, $^{2}J_{CF}$ = 25.2 Hz, C5), 30.5 (t, $^{2}J_{CF}$ = 33.7 Hz, C7).

GC-EI-MS: Retention: 6.50 min (*m/z*) requires: [($C_{10}H_8BrF_5$)] = 301.9724, (*m/z*) found: [($C_{10}H_8BrF_5$)] = 301.9724.

FT-IR ($\tilde{\nu}$ = cm^{-1})**:** 1622.3 (w), 1419.2 (w), 1324.7 (s), 1253.2 (w), 1109.5 (s), 1066.9 (s), 1014.7 (m), 956.0 (w), 891.1 (m), 848.9 (m), 796.6 (s), 737.2 (m), 687.2 (w), 666.6 (m).

Methyl 4-(3-bromo-2,2-difluoropropyl)benzoate (2i)

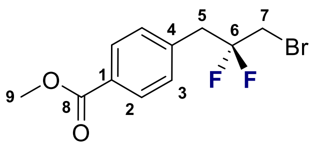

Compound **2i** was prepared according to General Procedure **E** using methyl 4-(3-bromoprop-1-en-2-yl)benzoate **1i** (128 mg, 0.50 mmol). The crude mixture was purified by column chromatography (100% pentane) to yield the title compound as a white solid (133 mg, 0.45 mmol, 91%).

R*f* = 0.29 (5% EtOAc in pentane)

Melting Point: 80-82°C

^{1}**H NMR** (500 MHz, $CDCl_3$): δ [ppm] = 8.01 (m, 2H, H-C2), 7.41 (m, 2H, H-C3), 3.92 (s, 3H, H-C9), 3.42 (t, $^{3}J_{HF}$ = 15.7 Hz, 2H, H-C5), 3.40 (t, $^{3}J_{HF}$ = 12.6 Hz, 2H, H-C7).

^{19}**F NMR** (376 MHz, $CDCl_3$): δ [ppm] = −97.78 (tt, $^{3}J_{FH}$ = 15.6 Hz, $^{3}J_{FH}$ = 12.5 Hz, 2F, F-C6).

^{19}**F{^{1}H} NMR** (376 MHz, $CDCl_3$): δ [ppm] = −97.78 (s, 2F, F-C6).

^{13}C{^{1}H} NMR (126 MHz, $CDCl_3$): δ [ppm] = 166.9 (s, C8), 137.4 (t, $^{3}J_{CF}$ = 4.6 Hz, C4), 130.5 (t, $^{5}J_{CF}$ = 0.8 Hz, C2), 130.0 (s, C3), 129.9 (s, C1), 120.6 (t, $^{1}J_{CF}$ = 244.8 Hz, C6), 52.3 (s, C9), 40.8 (t, $^{2}J_{CF}$ = 25.2 Hz, C5), 30.6 (t, $^{2}J_{CF}$ = 33.6 Hz, C7).

ESI-MS: (*m/z*) requires: [($C_{11}H_{11}BrF_2O_2Na)^+$] = 314.9803, (*m/z*) found: [($C_{11}H_{11}BrF_2O_2Na)^+$] = 314.9804.

FT-IR ($\tilde{\nu}$ = cm^{-1})**:** 1723.7 (s), 1613.6 (w), 1427.9 (m), 1418.9 (m), 1347.8 (w), 1308.1 (w), 1273.3 (s), 1188.0 (s), 1103.16 (s), 1093.0 (s), 1020.4 (s), 965.5 (w), 900.4 (m), 859.8 (w), 810.8 (m), 763.2 (s), 707.2 (s), 687.0 (m), 663.9 (m).

1-(3-Bromo-2,2-difluoropropyl)-4-nitrobenzene (2j)

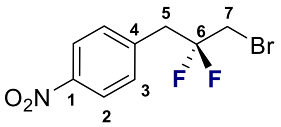

Compound **2j** was prepared according to General Procedure **E** using 1-(3-bromoprop-1-en-2-yl)-4-nitrobenzene **1j** (121 mg, 0.50 mmol). The crude mixture was purified by column chromatography (50% DCM in pentane) to yield the title compound as a yellow solid (118 mg, 0.42 mmol, 84%).

R*f* = 0.43 (50% DCM in pentane)

Melting Point: 95-97°C

^{1}H NMR (500 MHz, $CDCl_3$): δ [ppm] = 8.21 (m, 2H, H-C2), 7.52 (m, 2H, H-C3), 3.49 (t, $^{3}J_{HF}$ = 15.8 Hz, 2H, H-C5), 3.34 (t, $^{3}J_{HF}$ = 12.4 Hz, 2H, H-C7).

^{19}F NMR (376 MHz, $CDCl_3$): δ [ppm] = −96.96 (tt, $^{3}J_{FH}$ = 15.8 Hz, $^{3}J_{FH}$ = 12.4 Hz, 2F, F-C6).

^{19}F{^{1}H} NMR (376 MHz, $CDCl_3$): δ [ppm] = −96.95 (s, 2F, F-C6).

^{13}C{^{1}H} NMR (126 MHz, $CDCl_3$): δ [ppm] = 147.9 (s, C1), 139.6 (t, $^{3}J_{CF}$ = 4.1 Hz, C4), 131.5 (t, $^{4}J_{CF}$ = 0.9 Hz, C3), 123.9 (s, C2), 120.3 (t, $^{1}J_{CF}$ = 245.1 Hz, C6), 40.5 (t, $^{2}J_{CF}$ = 25.3 Hz, C5), 30.5 (t, $^{2}J_{CF}$ = 33.8 Hz, C7).

GC-EI-MS: Retention: 8.50 min (*m/z*) requires: [($C_9H_8BrF_2NO_2$)] = 278.9701, (*m/z*) found: [($C_9H_8BrF_2NO_2$)] = 278.9703.

FT-IR ($\tilde{\nu}$ = cm^{-1})**:** 1602.8 (w), 1514.3 (s), 1420.1 (w), 1346.6 (s), 1253.9 (m), 1186.9 (s), 1095.7 (s), 1008.8 (s), 894.8 (m), 852.0 (m), 803.8 (s), 748.5 (s), 704.6 (s), 659.3 (m).

4-(3-Bromo-2,2-difluoropropyl)phenyl 4-methylbenzenesulfonate (2k)

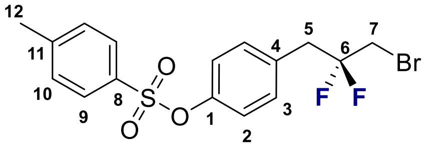

Compound **2k** was prepared according to General Procedure **E** using 4-(3-bromoprop-1-en-2-yl)phenyl 4-methylbenzenesulfonate **1k** (183 mg, 0.50 mmol). The crude mixture was purified by column chromatography (10% EtOAc in pentane) to yield the title compound as a white solid (148 mg, 0.37 mmol, 73%).

R*f* = 0.26 (10% EtOAc in pentane)

Melting Point: 92-94°C

^{1}H NMR (599 MHz, $CDCl_3$): δ [ppm] = 7.71 (m, 2H, H-C9), 7.32 (m, 2H, H-C10), 7.25 (m, 2H, H-C3), 6.97 (m, 2H, H-C2), 3.37 (t, $^{3}J_{HF}$ = 12.5 Hz, 2H, H-C7), 3.32 (t, $^{3}J_{HF}$ = 15.7 Hz, 2H, H-C5), 2.45 (s, 3H, H-C12).

^{19}F NMR (376 MHz, $CDCl_3$): δ [ppm] = −97.22 (tt, $^{3}J_{FH}$ = 15.6 Hz, $^{3}J_{FH}$ = 12.5 Hz, 2F, F-C6).

^{19}F{^{1}H} NMR (376 MHz, $CDCl_3$): δ [ppm] = −97.22 (s, 2F, F-C6).

^{13}C{^{1}H} NMR (151 MHz, $CDCl_3$): δ [ppm] = 149.4 (s, C1), 145.6 (s, C11), 132.5 (s, C8), 131.7 (t, $^{4}J_{CF}$ = 0.8 Hz, C3) 131.3 (t, $^{3}J_{CF}$ = 4.7 Hz, C4), 129.9 (s, C10), 128.6 (s, C9), 122.7 (s, C2), 120.6 (t, $^{1}J_{CF}$ = 244.5 Hz, C6), 40.1 (t, $^{2}J_{CF}$ = 25.2 Hz, C5), 30.5 (t, $^{2}J_{CF}$ = 33.5 Hz, C7), 21.9 (s, C12).

ESI-MS: (*m/z*) requires: [$(C_{16}H_{15}Br_2F_2O_3SNa)^+$] = 426.9786, (*m/z*) found: [$(C_{16}H_{15}Br_2F_2O_3SNa)^+$] = 426.9784.

FT-IR ($\tilde{\nu}$ = cm^{-1})**:** 1594.8 (w), 1502.9 (m), 1432.8 (w), 1419.7 (w), 1364.5 (s), 1349.7 (m), 1298.5 (w), 1259.5 (m), 1198.0 (s), 1175.0 (s), 1151.6 (s), 1120.5 (w), 1105.7 (m), 1091.1 (s), 1025.3 (s), 896.0 (m), 864.0 (s), 850.9 (s), 825.3 (s), 805.5 (s), 738.1 (w), 725.7 (s), 681.4 (w), 661.7 (s).

2-Bromo-4-(3-bromo-2,2-difluoropropyl)-1-fluorobenzene (2l)

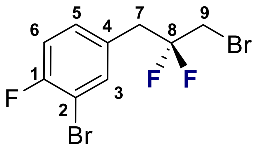

Compound **2l** was prepared according to General Procedure **E** using 2-bromo-4-(3-bromoprop-1-en-2-yl)-1-fluorobenzene **1l** (147 mg, 0.50 mmol). The crude mixture was purified by column chromatography (100% pentane) to yield the title compound as a white solid (125 mg, 0.41 mmol, 81%).

R*f* = 0.29 (pentane)

Melting Point: 48-50°C

^{1}H NMR (500 MHz, $CDCl_3$): δ[ppm] = 7.53 (dd, $^{4}J_{HF}$ = 6.5, $^{4}J_{HH}$ = 2.2 Hz, 1H, H-C3), 7.28 – 7.23 (m, 1H, H-C5), 7.10 (t, $^{3}J_{HF}$, $^{3}J_{HH}$ = 8.4 Hz, 1H, H-C6), 3.42 (t, $^{3}J_{HF}$ = 12.5 Hz, 2H, H-C9), 3.33 (t, $^{3}J_{HF}$ = 15.6 Hz, 2H, H-C7).

^{19}F NMR (376 MHz, $CDCl_3$): δ[ppm] = −97.39 (tt, $^{3}J_{FH}$ = 15.6 Hz, $^{3}J_{FH}$ = 12.4 Hz, 2F, F-C8), −108.49 (ddd, $^{2}J_{FH}$ = 8.4, $^{3}J_{FH}$ = 6.5, $^{3}J_{FH}$ = 4.5 Hz, 1F, F-C1).

^{19}F{^{1}H} NMR (376 MHz, $CDCl_3$): δ[ppm] = −97.39 (s, 2F, F-C8), −108.49 (s, 1F, F-C1).

^{13}C{^{1}H} NMR (126 MHz, $CDCl_3$): δ[ppm] = 159.0 (d, $^{1}J_{CF}$ = 248.1 Hz, C1), 135.4 (d, $^{3}J_{CF}$ = 1.1 Hz, C3), 131.0 (d, $^{3}J_{CF}$ = 7.2 Hz, C5), 130.1 – 129.1 (m, C4), 121.5 (td, $^{1}J_{CF}$ = 248.1, $^{6}J_{CF}$ 1.5 Hz, C8), 116.8 (d, $^{2}J_{CF}$ = 22.4 Hz, C6), 109.4 (d, $^{2}J_{CF}$ = 22.1 Hz, C2), 39.7 (t, $^{2}J_{CF}$ = 25.4 Hz, C7), 30.5 (t, $^{2}J_{CF}$ = 33.6 Hz, C9).

GC-EI-MS: Retention: 7.76 min (*m/z*) requires: [($C_9H_7Br_2F_3$)] = 331.8841, (*m/z*) found: [($C_9H_7Br_2F_3$)] = 331.8832.

FT-IR ($\tilde{\nu}$ = cm^{-1})**:** 3060.0 (w), 2958.5 (br), 1894.2 (w), 1733.5 (m), 1600.7 (w), 1492.8 (s), 1434.8 (m), 1423.1 (m), 1348.5 (m), 1301.9 (w), 1246.0 (s), 1204.8 (w), 1188.9 (s), 1125.7 (m), 1051.9 (s), 1033.00 (w), 1009.3 (s), 957.0 (w), 902.0 (w), 894.4 (m), 875.0 (m), 856.2 (m), 830.7 (m), 799.7 (s), 779.7 (s), 712.9 (m), 685.3 (m), 674.2 (m).

1-((4-(3-Bromo-2,2-difluoropropyl)phenyl)sulfonyl)piperidine (2m)

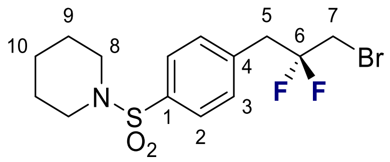

Compound **2m** was prepared according to General Procedure **E** using 1-((4-(3-bromoprop-1-en-2-yl)phenyl)sulfonyl)piperidine **1m** (172 mg, 0.50 mmol). The crude mixture was purified by column chromatography (0-14% EtOAc in pentane) to afford the title compound as a white solid (144 mg, 0.38 mmol, 75 %).

R*f* = 0.32 (20% EtOAc in pentane).

Melting point: 62-64°C

^{1}H NMR (500 MHz, $CDCl_3$) δ [ppm] 7.73 (m, 2H, H-C2), 7.49 (m, 2H, H-C3), 3.44 (t, $^3J_{HF}$ = 15.8 Hz, 2H, H-C5), 3.43 (t, $^3J_{HF}$ = 12.5 Hz, 2H, H-C7)3.08 - 2.95 (m, 4H, H-C8), 1.69 – 1.57 (m, 4H, H-C9), 1.50 - 1.39 (m, 2H, H-C10).

^{19}F NMR (377 MHz, $CDCl_3$) δ [ppm] -97.00 (tt, $^3J_{FH}$ = 15.6 Hz, $^3J_{FH}$ = 12.5 Hz, 2F, F-C6).

^{19}F{^{1}H} NMR (377 MHz, $CDCl_3$) δ [ppm] -97.00 (s, 2F, F-C6).

^{13}C{^{1}H} NMR (126 MHz, $CDCl_3$) δ [ppm] 137.2 (t, $^3J_{CF}$ = 4.3 Hz, C4), 136.3 (C1), 131.0 (C3), 128.1 (C2), 120.4 (t, $^1J_{CF}$ = 244.9 Hz, C6), 47.1 (C8), 40.6 (t, $^2J_{CF}$ = 25.2 Hz, C5), 30.6 (t, $^2J_{CF}$ = 33.6 Hz, C7), 25.3 (C9), 23.6 (C10).

ESI-MS: (*m/z*) requires: [$(C_{14}H_{18}NO_2SBrNa)^+$] = 368.0114, (*m/z*) found: [$(C_{14}H_{18}NO_2SBrNa)^+$] = 368.0111.

FT-IR ($\tilde{\nu}$ = cm^{-1})**:** 3043 (w), 2936 (w), 2850 (w), 1604 (w), 1498 (w), 1476 (w), 1453 (w), 1445 (w), 1432 (w), 1421 (w), 1412 (w), 1383 (w), 1361 (w), 1334 (m), 1280 (w), 1258 (m), 1203 (w), 1152 (m), 1096 (m), 1054 (w), 1026 (s), 931 (m), 913 (w), 894 (w), 856 (m), 840 (w), 810 (w), 793 (m), 766 (w), 748 (m), 718 (m), 694 (m), 671 (m).

2-(4-(3-Bromo-2,2-difluoropropyl)phenyl)isoindoline-1,3-dione (2n)

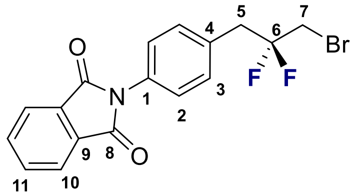

Compound **2n** was prepared according to General Procedure **E** using 2-(4-(3-bromoprop-1-en-2-yl)phenyl)isoindoline-1,3-dione **1n** (171 mg, 0.50 mmol). The crude mixture was purified by column chromatography

(80% DCM in pentane) to yield the title compound as a yellow solid (152 mg, 0.40 mmol, 80%).

R*f* = 0.45 (80% DCM in pentane)

Melting Point: 145-147°C

^{1}H NMR (500 MHz, $CDCl_3$): δ [ppm] = 7.96 (m, 2H, H-C10), 7.80 (m, 2H, H-C11), 7.48 (m, 2H, H-C3), 7.45 (m, 2H, H-C2), 3.45 (t, $^3J_{HF}$ = 12.6 Hz, 2H, H-C7), 3.43 (t, $^3J_{HF}$ = 15.5 Hz, 2H, H-C5).

^{19}F NMR (376 MHz, $CDCl_3$): δ [ppm] = −96.77 (tt, $^3J_{FH}$ = 15.4 Hz, $^3J_{FH}$ = 12.6 Hz, 2F, F-C6).

^{19}F{^{1}H} NMR (376 MHz, $CDCl_3$): δ [ppm] = −96.77 (s, 2F, F-C6).

^{13}C{^{1}H} NMR (126 MHz, $CDCl_3$): δ [ppm] = 167.3 (s, C8), 134.6 (s, C11), 132.2 (t, $^3J_{CF}$ = 5.1 Hz, C4), 131.9 (s, C9), 131.5 (s, C1), 131.2 (s, C3), 126.8 (s, C2), 124.0 (s, C10), 120.8 (t, $^1J_{CF}$ = 244.8 Hz, C6), 40.4 (t, $^2J_{CF}$ = 25.2 Hz, C5), 30.6 (t, $^2J_{CF}$ = 33.4 Hz, C7).

ESI-MS: (*m/z*) requires: [$(C_{16}H_{12}BrF_2NO_2Na)^+$] = 401.9912, (*m/z*) found: [$(C_{16}H_{12}BrF_2NO_2Na)^+$] = 401.9909.

FT-IR ($\tilde{\nu}$ = cm^{-1})**:** 1714.5 (s), 1514.9 (w), 1367.6 (s), 1262.1 (m), 1194.6 (m), 1126.5 (w), 1093.7 (m), 1069.3 (m), 1017.5 (s), 882.1 (m), 846.5 (m), 790.3 (s), 735.0 (w), 714.1 (s).

1-(3-Bromo-2,2-difluoropropyl)-4-(3,3,3-trifluoroprop-1-en-2-yl)benzene (4)

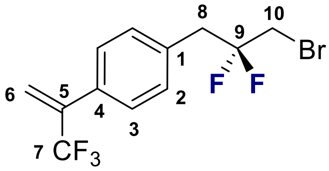

Compound 4 was prepared according to modified General Procedure **E** using an amine:HF ratio of 1:4.5 ($NEt_3 \cdot 3HF$ 0.43 mL, Olah´s reagent 0.20 mL) and 1-(3-bromoprop-1-en-2-yl)-4-(3,3,3-trifluoroprop-1-en-2-yl)benzene **3** (73.0 mg, 0.25 mmol). The stoichiometry of the reagents and solvents was adapted to the reaction scale (*p*TolI 11 mg, Selectfluor® 133 mg, amine:HF 0.63 mL, and $CHCl_3$ 0.63 mL). The crude mixture was purified by column chromatography (0-2% DCM in pentane) to yield the title compound as a colorless oil (65.0 mg, 0.20 mmol, 79%).

R*f* = 0.30 (2% DCM in pentane)

^{1}H NMR (599 MHz, $CDCl_3$): δ[ppm] = 7.45 (m, 2H, H-C3), 7.36 (m, 2H, H-C2), 5.98 (q, $^{4}J_{HF}$ = 1.4 Hz, 1H, H^a-C6), 5.79 (q, $^{4}J_{HF}$ = 1.7 Hz, 1H, H^b-C6), 3.42 (t, $^{3}J_{HF}$ = 12.6 Hz, 2H, H-C10), 3.38 (t, $^{3}J_{HF}$ = 15.6 Hz, 2H, H-C8).

^{19}F NMR (376 MHz, $CDCl_3$): δ[ppm] = −64.75 (s, 3F, F-C7), −97.10 (tt, $^{3}J_{FH}$ = 15.6 Hz, $^{3}J_{FH}$ = 12.6 Hz, 2F, F-C9).

^{19}F{^{1}H} NMR (376 MHz, $CDCl_3$): δ[ppm] = −64.75 (s, 3F, F-C7), −96.98 (s, 2F, F-C9).

^{13}C{^{1}H} NMR (151 MHz, $CDCl_3$): δ[ppm] = 138.6 (q, $^{2}J_{CF}$ = 30.1 Hz, C5), 133.3 (s, C4), 133.2 (t, $^{3}J_{CF}$ = 4.8 Hz, C1), 130.6 (s, C3), 127.8 (q, $^{5}J_{CF}$ = 1.3 Hz, C2), 123.4 (q, $^{1}J_{CF}$ = 274.0 Hz, C7), 120.8 (q, $^{3}J_{CF}$ = 5.8 Hz, C6), 120.7 (t, $^{1}J_{CF}$ = 244.6 Hz, C9), 40.5 (t, $^{2}J_{CF}$ = 25.1 Hz, C8), 30.6 (t, $^{2}J_{CF}$ = 33.5 Hz, C10).

GC-EI-MS: Retention: 7.18 min (*m/z*) requires: [($C_{12}H_{10}BrF_5$)] = 327.9881, (*m/z*) found: [($C_{12}H_{10}BrF_5$)] = 327.9877.

FT-IR ($\tilde{\nu}$ = cm^{-1})**:** 2937.0 (w), 1517.7 (w), 1425.1 (w), 1352.9 (m), 1301.5 (w), 1273.2 (w), 1254.1 (m), 1185.7 (m), 1164.8 (s), 1116.3 (s), 1077.9 (s), 1069.0 (s), 1028.2 (s), 946.3 (m), 907.4 (w), 896.7 (w), 844.4 (m), 829.1 (w), 804.7 (w), 794.8 (m), 752.2 (w), 732.9 (w), 695.0 (w), 658.8 (w).

1-(3-Bromo-2,2-difluoropropyl)-4-(1,1,1,2,3-pentafluoropropan-2-yl)benzene (5)

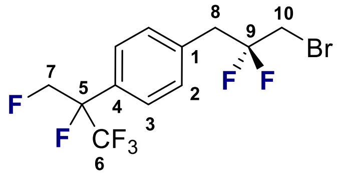

Compound **5** was prepared according to modified General Procedure **E** using an amine:HF ratio of 1:7.5 (NEt_3·3HF 0.52 mL, Olah´s reagent 1.98 mL) and 1-(3-bromoprop-1-en-2-yl)-4-(3,3,3-trifluoroprop-1-en-2-yl)benzene **3** (146 mg, 0.50 mmol). The amounts of the reagents and solvents were adapted to the reaction scale (*p*TolI 44.0 mg, Selectfluor® 532 mg, amine:HF 2.5 mL, $CHCl_3$ 2.5 mL). The crude mixture was purified by column chromatography (0-5% DCM in pentane) to yield the title compound as a colorless oil (101 mg, 0.28 mmol, 55%).

R*f* = 0.23 (5% DCM in pentane)

^{1}H NMR (599 MHz, $CDCl_3$): δ[ppm] = 7.50 (m, 2H, H-C3), 7.45 (m, 2H, H-C2), 4.94 (ddd, $^{2}J_{HF}$ = 47.5, $^{3}J_{HF}$ = 20.6, $^{3}J_{HF}$ = 11.2 Hz, 1H, H^a-C7), 4.86 (dddq, $^{2}J_{HF}$ = 46.0,

$^{3}J_{HF}$ = 21.8, $^{2}J_{HH}$ = 11.1 Hz, $^{4}J_{HF}$ = 1.3 Hz 1H, H^{b}-C7), 3.42 (t, $^{3}J_{HF}$ = 12.7 Hz, 2H, H-C10), 3.42 (t, $^{3}J_{HF}$ = 15.7 Hz, 2H, H-C8).

^{19}F NMR (376 MHz, CDCl$_3$): δ [ppm] = −77.31 (t, $^{4}J_{FF}$, $^{3}J_{FF}$ = 7.7 Hz, 3F, F-C6)*, −97.01 (tt, $^{3}J_{FH}$ = 15.6 Hz, $^{3}J_{FH}$ = 12.6 Hz, 2F, F-C9), −179.47 (tdq, $^{3}J_{FH}$ = 27.7, $^{3}J_{FF}$ = 13.9, $^{3}J_{FF}$ = 7.1 Hz, 1F, F-C5), −233.95 (tdq, $^{2}J_{FH}$ = 46.7, $^{3}J_{FF}$ = 13.1, $^{4}J_{FF}$ = 8.3 Hz, 1F, F-C7).

^{19}F{^{1}H} NMR (376 MHz, CDCl$_3$): δ [ppm] = −77.32 (dd, $^{4}J_{FF}$ = 8.3, $^{3}J_{FF}$ = 7.0 Hz, 3F, F-C6), −97.01 (s, 2F, F-C9), −179.47 (dq, $^{3}J_{FF}$ = 13.6, $^{3}J_{FF}$ = 7.2 Hz, 1F, F-C5), −233.95 (dq, $^{3}J_{FF}$ = 13.2, $^{4}J_{FF}$ = 8.3 Hz, 1F, F-C7).

^{13}C{^{1}H} NMR (151 MHz, CDCl$_3$): δ [ppm] = 134.52 (t, $^{3}J_{CF}$ = 4.8 Hz, C1), 130.9 (s, C2), 130.1 (dd, $^{2}J_{CF}$ = 21.4, $^{3}J_{CF}$ = 3.0 Hz, C4), 126.1 (d, $^{3}J_{CF}$ = 9.6 Hz, C3), 122.3 (qdd, $^{1}J_{CF}$ = 285.2, $^{2}J_{CF}$ = 29.7, $^{3}J_{CF}$ = 4.1 Hz, C6), 120.6 (t, $^{1}J_{CF}$ = 244.6 Hz, C9), 93.76 (dqd, $^{1}J_{CF}$ = 192.0, $^{2}J_{CF}$ = 31.4, $^{2}J_{CF}$ = 19.0 Hz, C5), 81.6 (ddq, $^{1}J_{CF}$ = 185.7, $^{2}J_{CF}$ = 24.1 Hz, C7), 40.4 (t, $^{2}J_{CF}$ = 25.2 Hz, C8), 30.6 (t, $^{2}J_{CF}$ = 33.6 Hz, C10).

* unresolved dddd, see [5]

GC-EI-MS: Retention: 7.18 min (*m/z*) requires: [($C_{12}H_{10}BrF_7$)] = 365.9849, (*m/z*) found: [($C_{12}H_{10}BrF_7$)] = 365.9848.

FT-IR ($\tilde{\nu}$ = cm^{-1})**:** 2975.1 (w), 1619.3 (w), 1518.9 (w), 1414.1 (w), 1395.0 (w), 1318.2 (m), 1294.8 (m), 1254.6 (m), 1237.3 (m), 1178.4 (s), 1151.9 (s), 1117.6 (m), 1099.4 (m), 1070.0 (m), 1052.7 (m), 1029.0 (s), 970.3 (m), 954.6 (m), 908.8 (m), 897.2 (m), 846.3 (m), 832.2 (m), 790.5 (m), 760.7 (w), 746.4 (w), 734.0 (m), 722.0 (w), 697.4 (m), 672.0 (w), 653.4 (w).

1-(3-Bromo-2,2-difluoropropyl)-4-(2,2,3,3,3-pentafluoropropyl)benzene (5-*gem*)

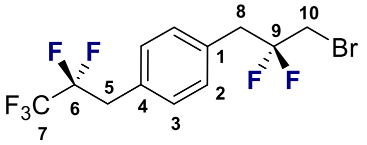

Compound **5-*gem*** was prepared according to modified General Procedure **E** using an amine:HF ratio of 1:7.5 (NEt_3·3HF 0.52 mL, Olah´s reagent 1.98 mL) and 1-(3-bromoprop-1-en-2-yl)-4-(3,3,3-trifluoroprop-1-en-2-yl)benzene **3** (146 mg, 0.5 mmol). The stoichiometry of the reagents and solvents was adapted to the reaction scale (*p*TolI 44.0 mg, Selectfluor® 532 mg, amine:HF 2.5 mL,

and $CHCl_3$ 2.5 mL). The crude mixture was purified by column chromatography (0-5% DCM in pentane) to yield the title compound as a white solid (37.0 mg, 0.10 mmol, 20%).

R*f* = 0.43 (5% DCM in pentane)

Melting Point: 80-82°C

^{1}H NMR (599 MHz, $CDCl_3$): δ[ppm] = 7.33 (m, 2H, H-C3), 7.28 (m, 2H, H-C2), 3.41 (t, $^3J_{HF}$ = 12.7 Hz, 2H, H-C10), 3.38 (t, $^3J_{HF}$ = 15.7 Hz, 2H, H-C8), 3.32 (t, $^3J_{HF}$ = 18.1 Hz, 2H, H-C5).

^{19}F NMR (376 MHz, $CDCl_3$): δ[ppm] = −84.72 (t, $^4J_{FH}$ = 1.0 Hz, 3F, F-C7), −97.05 (tt, $^3J_{FH}$ = 15.6 Hz, $^3J_{FH}$ = 12.6 Hz, 2F, F-C9), −116.94 (t, $^3J_{FH}$ = 18.3 Hz, 2F, F-C6).

^{19}F{^{1}H} NMR (376 MHz, $CDCl_3$): δ[ppm] = −84.72 (s, 3F, F-C7), −97.05 (s, 2F, F-C9), −116.94 (s, 2F, F-C6).

^{13}C{^{1}H} NMR (151 MHz, $CDCl_3$): δ[ppm] = 132.40 (t, $^3J_{CF}$ = 4.8 Hz, C1), 131.1 (s, C3), 130.7 (s, C2), 128.9 (t, $^3J_{CF}$ = 2.3 Hz, C4), 120.8 (t, $^1J_{CF}$ = 244.5 Hz, C9), 119.3 (qt, $^1J_{CF}$ = 286.1, $^2J_{CF}$ = 36.1 Hz, C7), 114.5 (tq, $^1J_{CF}$ = 252.7, $^2J_{CF}$ = 37.3 Hz, C6), 40.5 (t, $^2J_{CF}$ = 25.1 Hz, C8), 36.8 (t, $^2J_{CF}$ = 22.2 Hz, C5), 30.6 (t, $^2J_{CF}$ = 33.5 Hz, C10).

GC-EI-MS: Retention: 6.86 min (*m/z*) requires: [($C_{12}H_{10}BrF_7$)] = 365.9849, (*m/z*) found: [($C_{12}H_{10}BrF_7$)] = 365.9835.

FT-IR ($\tilde{\nu}$ = cm^{-1})**:** 1516.6 (w), 1419.6 (w), 1318.2 (m), 1240.0 (w), 1183.5 (s), 1112.5 (w), 1095.8 (m), 1072.9 (m), 1031.7 (s), 1021.8 (s), 887.5 (m), 851.5 (m), 813.3 (w), 793.3 (w), 774.6 (s), 749.7 (w), 704.3 (m).

Methyl (*E*)-3-(4-(3-bromo-2,2-difluoropropyl)phenyl)acrylate (7)

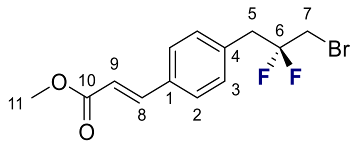

Compound **7** was prepared according to modified General Procedure **E** using an amine:HF ratio of 1:4.5 ($NEt_3{\cdot}3HF$ 0.85 mL, Olah´s reagent 0.40 mL) and methyl (*E*)-3-(4-(3-bromoprop-1-en-2-yl)phenyl)acrylate **6** (70.0 mg, 0.25 mmol). The stoichiometry of the reagents and solvents was adapted to the reaction scale (*p*TolI 22.0 mg, Selectfluor® 266 mg, amine:HF 1.25 mL, and $CHCl_3$ 1.25 mL). The crude mixture was purified by

column chromatography (0-7% EtOAc in pentane) to afford the title compound as a white solid (41.0 mg, 0.13 mmol, 51%).

R*f* = 0.52 (10% EtOAc in pentane).

Melting point: 95-97°C

^{1}H NMR (599 MHz, $CDCl_3$) δ [ppm] 7.68 (d, $^3J_{HH}$ = 16.0 Hz, 1H, H-C8), 7.50 (m, 2H, H-C2), 7.35 (m, 2H, H-C3), 6.44 (d, $^1J_{CF}$ = 16.0 Hz, 1H, H-C9), 3.81 (s, 3H, H-C11), 3.41 (t, $^3J_{HF}$ = 12.6 Hz, H-C7), 3.39 (t, $^3J_{HF}$ = 15.6 Hz, H-C5).

^{19}F NMR (564 MHz, $CDCl_3$) δ [ppm] -96.90 (tt, $^3J_{FH}$ = 15.6 Hz, $^3J_{FH}$ = 12.5 Hz, 2F, F-C6).

^{19}F{^{1}H} NMR (377 MHz, $CDCl_3$) δ [ppm] -96.86 (s, 2F, F-C6).

^{13}C{^{1}H} NMR (151 MHz, $CDCl_3$) δ [ppm] 167.4 (C10), 144.2 (C8), 134.6 (t, $^3J_{CF}$ = 4.7 Hz, C4), 134.1 (C1), 131.0 (C3), 128.4 (C2), 120.7 (t, $^1J_{CF}$ = 244.7 Hz, C6), 118.4 (C9), 51.9 (C11), 40.6 (t, $^2J_{CF}$ = 25.1 Hz, C5), 30.6 (t, $^2J_{CF}$ = 33.6 Hz, C7).

ESI-MS: (*m/z*) requires: $[(C_{13}H_{13}O_2F_2BrNa)^+]$ = 340.9959, (*m/z*) found: $[(C_{13}H_{13}O_2F_2BrNa)^+]$ = 340.9961.

FT-IR ($\tilde{\nu}$ = cm^{-1})**:** 3055 (w), 2963 (w), 1717 (s), 1637 (m), 1610 (w), 1570 (w), 1512 (w), 1437 (m), 1418 (m), 1343 (w), 1321 (m), 1287 (w), 1276 (m), 1254 (m), 1203 (m), 1190 (s), 1166 (s), 1113 (w), 1092 (m), 1011 (s), 976 (m), 952 (w), 936 (w), 891 (m), 871 (w), 857 (m), 840 (m), 796 (s), 748 (w), 721 (w), 704 (w), 679 (w).

Methyl 2-(4-(3-bromo-2,2-difluoropropyl)phenyl)-3,3-difluoropropanoate (8)

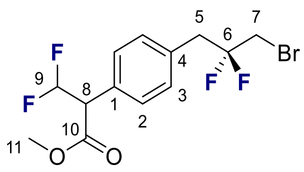

Compound **8** was prepared according to modified General Procedure **E** using methyl (*E*)-3-(4-(3-bromoprop-1-en-2-yl)phenyl)acrylate **6** (70.0 mg, 0.25 mmol). The stoichiometry of the reagents and solvents was adapted to the reaction scale (*p*TolI 22 mg, Selectfluor® 266 mg, amine:HF 1.25 mL, and $CHCl_3$ 1.25 mL). The crude mixture was purified by column chromatography (0-40% DCM in pentane) to afford the title compound as a colorless liquid. (49.0 mg, 0.14 mmol, 55%).

R*f* = 0.55 (60% DCM in pentane).

^{1}H NMR (500 MHz, $CDCl_3$) δ [ppm] 7.38 – 7.30 (m, 4H, H-C2, H-C3), 6.24 (td, $^2J_{HF}$ = 55.9 Hz, $^3J_{HH}$ = 6.6 Hz, 1H, H-C9), 4.04 (ddd, $^3J_{HF}$ = 12.4, $^3J_{HF}$ = 10.2, $^3J_{HH}$ = 6.6 Hz, 1H, H-C8), 3.76 (s, 3H, H-C11), 3.41 (t, $^3J_{FH}$ = 12.6 Hz, 2H, H-C7), 3.37 (t, $^3J_{FH}$ = 15.6 Hz, 2H, H-C5).

^{19}F NMR (470 MHz, $CDCl_3$) δ [ppm] -97.03 (tt, $^3J_{FH}$ = 15.6, $^3J_{FH}$ = 12.6 Hz, 2F, F-C6), -117.32 (ddd, $^2J_{FF}$ = 283.0, $^2J_{FH}$ = 55.2, $^3J_{FH}$ = 10.2 Hz, 1F, F^a-C9), -123.63 (ddd, $^2J_{FF}$ = 283.0, $^2J_{FH}$ = 55.8, $^3J_{FH}$ = 12.4 Hz, 1F, F^b-C9).

^{19}F{^{1}H} NMR (470 MHz, $CDCl_3$) δ [ppm] -97.03 (s, 2F, F-C6), -117.32 (d, $^2J_{FF}$ = 283.0 Hz, 1F, F^a-C9), -123.63 (d, $^2J_{FF}$ = 283.1 Hz,1F, F^b-C9).

^{13}C{^{1}H} NMR (126 MHz, $CDCl_3$) δ [ppm] 169.1 (dd, $^3J_{CF}$ = 11.0, $^3J_{CF}$ = 2.4 Hz, C10), 133.0 (t, $^3J_{CF}$ = 4.8 Hz, C4), 131.1 (C3), 130.8 (dd, $^3J_{CF}$ = 7.6, $^3J_{CF}$ = 0.8 Hz, C1), 129.3 (s, C2), 120.7 (t, $^1J_{CF}$ = 244.5 Hz, C6), 115.5 (t, $^1J_{CF}$ = 244.4 Hz, C9), 55.6 (t, $^2J_{CF}$ = 23.9 Hz, C8), 52.8 (C11), 40.4 (t, $^2J_{CF}$ = 25.1 Hz, C5), 30.6 (t, $^2J_{CF}$ = 33.5 Hz, C7).

ESI-MS: (*m/z*) requires: [$(C_{13}H_{13}O_2F_4BrNa)^+$] = 378.9927, (*m/z*) found: [$(C_{13}H_{13}O_2F_2BrNa)^+$] = 378.9926.

FT-IR ($\tilde{\nu}$ = cm^{-1})**:** 2958 (w), 1738 (s), 1517 (s), 1437 (m), 1389 (s), 1319 (m), 1254 (m), 1213 (m), 1169 (w), 1138 (m), 1068 (s), 1028 (s), 898 (w), 853 (w), 793 (w), 763 (w), 699 (w) cm^{-1}.

1,4-*Bis*(3-bromo-2,2-difluoropropyl)benzene (10)

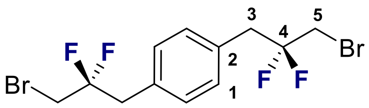

Compound **10** was prepared according to modified General Procedure **E** using 1,4-*bis*(3-bromoprop-1-en-2-yl)benzene **9** (79.0 mg, 0.25 mmol). The stoichiometry of the reagents and solvents was adapted to the reaction scale (*p*TolI 22 mg, Selectfluor® 266 mg, amine:HF 1.25 mL, and $CHCl_3$ 1.25 mL). The crude mixture was purified by column chromatography (0-2% EtOAc in pentane) to yield the title compound as a white solid (45.0 mg, 0.12 mmol, 46%)

R*f* = 0.38 (2% EtOAc in pentane)

Melting Point: 167-169°C

^{1}H NMR (599 MHz, $CDCl_3$): δ [ppm] = 7.31 (s, 4H, H-C1), 3.41 (t, $^{3}J_{HF}$ = 12.6 Hz, 4H, H-C7), 3.36 (t, $^{3}J_{HF}$ = 15.7 Hz, 4H, H-C5).

^{19}F NMR (376 MHz, $CDCl_3$): δ [ppm] = −97.10 (tt, $^{3}J_{FH}$ = 15.7, $^{3}J_{FH}$ = 12.7 Hz, 4F, F-C4).

^{19}F{^{1}H} NMR (376 MHz, $CDCl_3$): δ [ppm] = −97.10 (s, 4F, F-C4).

^{13}C{^{1}H} NMR (151 MHz, $CDCl_3$): δ [ppm] = 131.9 (t, $^{3}J_{CF}$ = 4.8 Hz, C2), 130.7 (s, C1), 120.8 (t, $^{1}J_{CF}$ = 244.4 Hz, C4), 40.5 (t, $^{2}J_{CF}$ = 25.1 Hz, C5), 30.7 (t, $^{2}J_{CF}$ = 33.5 Hz, C7).

GC-EI-MS: Retention: 8.68 min (*m/z*) requires: [($C_{12}H_{12}Br_2F_4$)] = 391.9216, (*m/z*) found: [($C_{12}H_{12}Br_2F_4$)] = 391.9213.

FT-IR ($\tilde{\nu}$ = cm^{-1})**:** 3058.5 (w), 1513.7 (w), 1420.0 (m), 1347.0 (m), 1272.1 (w), 1245.3 (m), 1187.2 (s), 1108.8 (w), 1094.7 (s), 1023.7 (w), 1002.2 (s), 884.8 (m), 853.3 (m), 801.5 (m), 776.7 (s), 684.9 (w).

1,3-*Bis*(3-bromo-2,2-difluoropropyl)benzene (12)

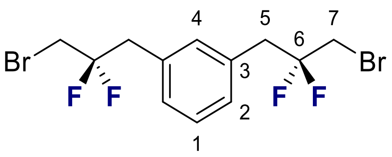

Compound **12** was prepared according to modified General Procedure **E** using 1,3-*bis*(3-bromoprop-1-en-2-yl)benzene **11** (87.0 mg, 0.25 mmol). The stoichiometry of the reagents and solvents was adapted to the reaction scale (*p*TolI 22 mg, Selectfluor® 266 mg, amine:HF 1.25 mL, and $CHCl_3$ 1.25 mL). The crude mixture was purified by column chromatography (0-2% EtOAc in pentane) to afford the title compound as a yellow solid (69.0 mg, 0.18 mmol, 70%).

R*f* = 0.45 (2% EtOAc in pentane).

Melting point: 65-67°C

^{1}H NMR (599 MHz, $CDCl_3$) δ [ppm] 7.36 - 7.32 (m, 1H, H-C4), 7.31 - 7.27 (m, 3H, H-C1, H-C3), 3.41 (t, $^{3}J_{HF}$ = 12.7 Hz, 4H, H-C7), 3.38 (t, $^{3}J_{HF}$ = 15.7 Hz, 4H, H-C5).

^{19}F NMR (564 MHz, $CDCl_3$) δ [ppm] -97.06 (tt, $^{3}J_{FH}$ = 15.6, $^{3}J_{FH}$ = 12.6 Hz, 4F, F-C6).

^{19}F{^{1}H} NMR (377 MHz, $CDCl_3$) δ [ppm] -97.04 (s, 4F, F-C6).

^{13}C{^{1}H} NMR (151 MHz, $CDCl_3$) δ[ppm] 132.8 (t, $^{3}J_{CF}$ = 4.7 Hz, C2), 132.4 (C1), 129.8 (C3), 129.2 (C4), 120.7 (t, $^{1}J_{CF}$ = 244.5 Hz, C6), 40.7 (t, $^{2}J_{CF}$ = 25.0 Hz, C5), 30.7 (t, $^{2}J_{CF}$ = 33.5 Hz, C7).

ESI-MS: (*m/z*) requires: [($C_{12}H_{12}F_4Br_2$)] = 391.9216, (*m/z*) found: [($C_{12}H_{12}F_4Br_2$)] = 391.9214.

FT-IR ($\tilde{\nu}$ = cm^{-1})**:** 3050 (w), 2987 (w), 2946 (w), 1610 (w), 1595 (w), 1490 (w), 1449 (w), 1430 (w), 1419 (w), 1357 (w), 1268 (m), 1251 (w), 1191 (m), 1175 (w), 1153 (w), 1094 (s), 1011 (s), 945 (w), 912 (w), 881 (m), 852 (m), 818 (m), 777 (s), 708 (s), 690 (w), 667 (w), 651 (s) cm^{-1}.

Ethyl 4-bromo-3,3-difluoro-2-phenylbutanoate (15)

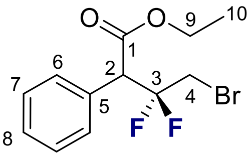

Compound **15** was prepared according to modified General Procedure **E** using an amine:HF ratio of 1:9.23 (Olah´s reagent 1.25 mL) and ethyl (*E*)-4-bromo-3-phenylbut-2-enoate **13** (135 mg, 0.50 mmol). The crude mixture was purified by column chromatography (0-2% Et_2O in pentane) to yield the title compound as a colorless oil (127 mg, 0.41 mmol, 83%).

R*f* = 0.21 (2% Et_2O in pentane)

^{1}H NMR (599 MHz, $CDCl_3$): δ[ppm] = 7.44 (m, 2H, H-C7), 7.38 (m, 3H, H-C6, H-C8), 4.47 (dd, $^{3}J_{HF}$ = 13.8, $^{3}J_{HF}$ = 12.4 Hz, 1H, H-C2), 4.26 (dq, $^{2}J_{HH}$ = 10.8, $^{3}J_{HH}$ = 7.2 Hz, 1H, H^{a}-C9), 4.18 (dq, $^{2}J_{HH}$ = 10.8, $^{3}J_{HH}$ = 7.1 Hz, 1H, H^{b}-C9), 3.91 (ddd, $^{3}J_{HF}$ = 17.4, $^{2}J_{HH}$ = 11.9, $^{3}J_{HF}$ = 10.6 Hz, 1H, H^{a}-C4), 3.40 (dt, $^{3}J_{HF}$ = 16.5, $^{2}J_{HH}$, $^{3}J_{HF}$ = 11.8 Hz, 1H, H^{b}-C4), 1.25 (t, $^{3}J_{HH}$ = 7.1 Hz, 3H, H-C10).

^{19}F NMR (376 MHz, $CDCl_3$): δ[ppm] = −99.05 (ddt, $^{2}J_{FF}$ = 250.1, $^{3}J_{HF}$ = 17.3, $^{3}J_{HF}$ = 12.2 Hz, 1F, F^{a}-C3), −99.05 (dddd, $^{2}J_{FF}$ = 250.1, $^{3}J_{HF}$ = 16.4, $^{3}J_{HF}$ = 13.8, $^{3}J_{HF}$ = 10.8 Hz, 1F, F^{b}-C3).

^{19}F{^{1}H} NMR (376 MHz, $CDCl_3$): δ[ppm] = −99.58 (d, $^{2}J_{FF}$ = 250.0 Hz, 1F, F^{a}-C3), −100.82 (d, $^{2}J_{FF}$ = 249.9 Hz, 1F, F^{b}-C3).

^{13}C{^{1}H} NMR (151 MHz, $CDCl_3$): δ[ppm] = 167.9 (t, $^{3}J_{CF}$ = 4.5 Hz, C1), 130.9 (t, $^{3}J_{CF}$ = 3.3 Hz, C5), 129.6 (s, C7), 128.9 (s, C6), 128.8 (s, C8), 118.8 (t, $^{1}J_{CF}$ = 248.7 Hz,

C3), 61.8 (s, C9), 55.6 (t, $^2J_{CF}$ = 25.0 Hz, C2), 30.4 (t, $^2J_{CF}$ = 30.8 Hz, C4), 13.9 (s, C10).

ESI-MS: (*m/z*) requires: [$(C_{12}H_{13}BrF_2O_2Na)^+$] = 328.9959, (*m/z*) found: [$(C_{12}H_{13}BrF_2O_2Na)^+$] = 328.9960.

FT-IR ($\tilde{\nu}$ = cm^{-1})**:** 2985.7 (w), 1733.3 (s), 1603.4 (w), 1498.2 (w), 1456.3 (w), 1423.8 (w), 1393.0 (w), 1371.9 (m), 1341.5 (m), 1302.9 (w), 1262.4 (m), 1232.9 (m), 1203.9 (s), 1155.3 (s), 1108.6 (s), 1021.1 (s), 967.7 (w), 920.0 (w), 857.4 (w), 825.2 (w), 745.9 (m), 728.9 (m), 697.6 (s), 669.2 (w).

Enantioselective Version:

Compound **15** was prepared according to modified General Procedure **E** using an amine:HF ratio of 1:9.23 (Olah´s reagent 1.25 mL), **C4** (52.0 mg, 0.10 mmol, 0.2 eq.) as a catalyst and ethyl (*E*)-4-bromo-3-phenylbut-2-enoate **13** (135 mg, 0.50 mmol). The reaction was stirred for five days at room temperature. The crude mixture was purified by column chromatography (50% DCM in pentane) to yield the title compound as a colorless oil (109 mg, 0.36 mmol, 71%). The enantiomeric ratio was determined by chiral HPLC (chiral OJ-H column, *n*-hexane: isopropanol 95:5, 1.0 mL/min, t_R minor = 9.1 min, t_R major = 10.3 min, *e.r.* = 18:82.

ORD ($CHCl_3$, c 1.00) $[\alpha]_D^{25}$ = +47.374

HPLC trace ***rac*-15**

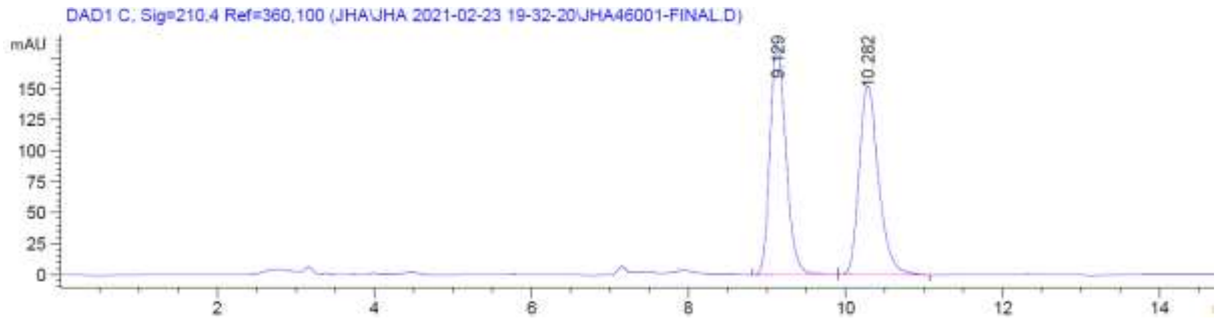

Signal 3: DAD1 C, Sig=210,4 Ref=360,100

Peak #	RetTime [min]	Type	Width [min]	Area [mAU*s]	Height [mAU]	Area %
1	9.129	BB	0.2163	2603.60474	184.82697	49.9839
2	10.282	BB	0.2608	2605.28442	152.92058	50.0161

Totals : 5208.88916 337.74754

HPLC trace ***ent*-15**

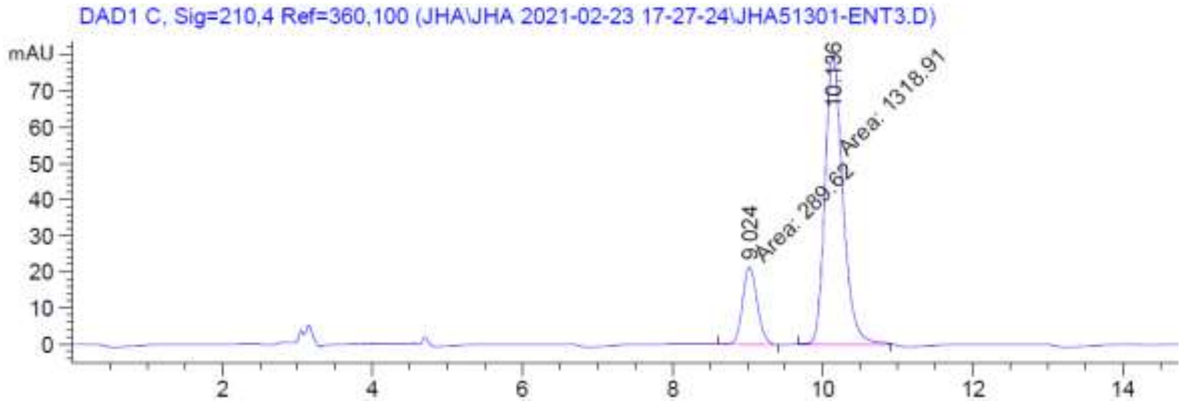

Signal 3: DAD1 C, Sig=210,4 Ref=360,100

Peak #	RetTime [min]	Type	Width [min]	Area [mAU*s]	Height [mAU]	Area %
1	9.024	MM	0.2242	289.61970	21.52632	18.0052
2	10.136	MM	0.2754	1318.91357	79.82316	81.9948
Totals :				1608.53336	101.34948	

Ethyl 4-chloro-3,3-difluoro-2-phenylbutanoate (16)

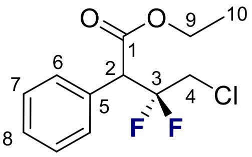

Compound **16** was prepared according to modified General Procedure **E** using an amine:HF ratio of 1:9.23 (Olah´s reagent 1.25 mL) and ethyl (*E*)-4-chloro-3-phenylbut-2-enoate ***E*-14** (112 mg, 0.50 mmol). The crude mixture was purified by column chromatography (0-2% Et_2O in pentane) to yield the title compound as a yellow oil (87.0 mg, 0.33 mmol, 66%).

R*f* = 0.36 (2% Et_2O in pentane)

^{1}H NMR (500 MHz, $CDCl_3$): δ[ppm] = 7.44 (m, 2H, H-C7), 7.40 – 7.34 (m, 3H, H-C, H-C), 4.43 (dd, $^3J_{HF}$ = 14.7, $^3J_{HF}$ = 12.8 Hz, 1H, H-C2), 4.25 (dq, $^2J_{HH}$ = 10.8, $^3J_{HH}$ = 7.1 Hz, 1H, H^a-C9), 4.18 (dq, $^2J_{HH}$ = 10.8, $^3J_{HH}$ = 7.1 Hz, 1H, H^b-C9), 4.00 (ddd, $^3J_{HF}$ = 14.2, $^2J_{HH}$ = 12.5, $^3J_{HF}$ = 11.7 Hz, 1H, H^a-C4), 3.54 (dt, $^3J_{HF}$ = 13.5, $^2J_{HH}$, $^3J_{HF}$ = 12.7 Hz, 1H, H^b-C4), 1.25 (t, $^3J_{HH}$ = 7.1 Hz, 3H, H-C10).

^{19}F NMR (377 MHz, $CDCl_3$): δ[ppm] = −103.1 (dq, $^2J_{FF}$ = 252.5, $^3J_{HF}$ = 13.2 Hz, 1F, F^a-C3), −104.1 (dq, $^2J_{FF}$ = 252.6, $^3J_{HF}$ = 13.4 Hz, 1F, F^b-C3).

^{19}F{^{1}H} NMR (377 MHz, $CDCl_3$): δ[ppm] = −103.1 (d, $^2J_{FF}$ = 252.7 Hz, 1F, F^a-C3), −104.1 (d, $^2J_{FF}$ = 252.5 Hz, 1F, F^b-C3).

$^{13}C\{^{1}H\}$ NMR (126 MHz, $CDCl_3$): δ [ppm] = 168.1 (t, $^{3}J_{CF}$ = 4.1 Hz, C1), 130.9 (dd, $^{3}J_{CF}$ = 3.8, $^{3}J_{CF}$ = 3.8 Hz, C5), 129.8 (t, $^{5}J_{CF}$ = 1.1 Hz, C7), 129.0 (s, C6), 129.0 (s, C8), 119.6 (t, $^{1}J_{CF}$ = 249.2 Hz, C3), 61.9 (s, C9), 55.1 (t, $^{2}J_{CF}$ = 24.4 Hz, C2), 43.4 (t, $^{2}J_{CF}$ = 31.0 Hz, C4), 14.1 (s, C10).

ESI-MS: (*m/z*) requires: [$(C_{12}H_{13}ClF_2O_2Na)^+$] = 285.0464, (*m/z*) found: [$(C_{12}H_{13}ClF_2O_2Na)^+$] = 285.0463.

FT-IR ($\tilde{\nu}$ = cm^{-1})**:** 3036.0 (w), 2984.4 (w), 1733.9 (s), 1604.3 (w), 1498.5 (w), 1456.6 (w), 1430.5 (w), 1393.1 (w), 1372.3 (m), 1342.9 (m), 1307.3 (m), 1267.1 (m), 1242.6 (w), 1209.4 (m), 1156.3 (s), 1119.1 (s), 1096.2 (w), 1080.2 (w), 1066.6 (m), 1045.5 (s), 1022.8 (s), 966.4 (w), 919.9 (w), 880.6 (m), 827.2 (m), 787.2 (m), 765.9 (m), 738.5 (m), 698.8 (s).

Enantioselective Version:

Compound **16** was prepared according to modified General Procedure **E** using an amine:HF ratio of 1:9.23 (Olah´s reagent 1.25 mL), **C4** (52.0 mg, 0.10 mmol, 0.2 eq.) as a catalyst and ethyl (*E*)-4-chloro-3-phenylbut-2-enoate ***E*-14** (112 mg, 0.50 mmol). The reaction was stirred for five days at room temperature. The crude mixture was purified by column chromatography (50% DCM in pentane) to yield the title compound as a colorless oil (99.0 mg, 0.38 mmol, 75%). The enantiomeric ratio was determined by chiral HPLC (chiral OJ-H column, *n*-hexane: isopropanol 95:5, 1.0 mL/min, t_R minor = 9.1 min, t_R major = 10.2 min, *e.r.* = 14:86.

ORD ($CHCl_3$, c 1.00) $[\alpha]_D^{25}$ = +49.681

HPLC trace ***rac*-16**

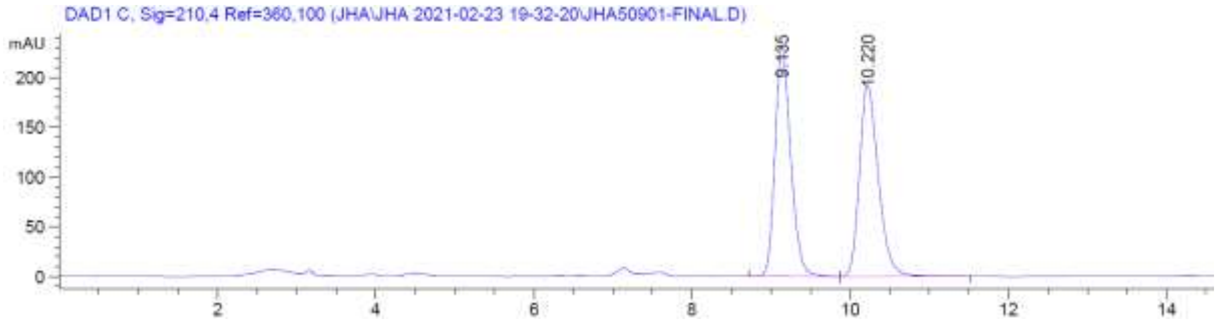

Signal 3: DAD1 C, Sig=210,4 Ref=360,100

Peak #	RetTime [min]	Type	Width [min]	Area [mAU*s]	Height [mAU]	Area %
1	9.135	BB	0.2131	3200.70435	231.83179	50.0602
2	10.220	BB	0.2554	3193.01001	192.58687	49.9398
Totals :				6393.71436	424.41866	

HPLC trace ***ent*-16**

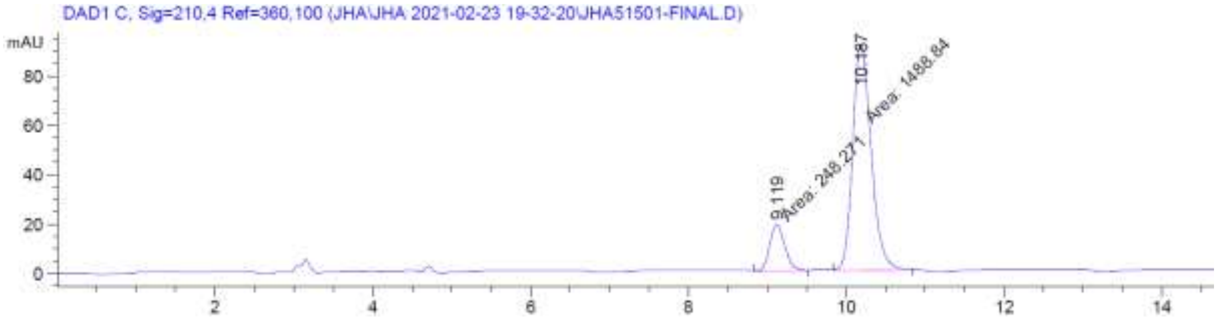

Signal 3: DAD1 C, Sig=210,4 Ref=360,100

Peak #	RetTime [min]	Type	Width [min]	Area [mAU*s]	Height [mAU]	Area %
1	9.119	MM	0.2224	248.27087	18.60201	14.2922
2	10.187	MM	0.2707	1488.84216	91.66241	85.7078
Totals :				1737.11304	110.26442	

3.6 Functionalizations of the *geminal* Difluorinated Products

4-(3-(4-Bromophenyl)-2,2-difluoropropoxy)benzonitrile (17)

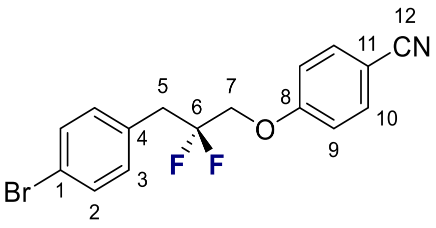

1-Bromo-4-(3-bromo-2,2-difluoropropyl)benzene (**2a**, 78.2 mg, 0.25 mmol, 1.0 eq.) was added to a solution of 4-hydroxybenzonitrile (44.7 mg, 0.38 mmol, 1.5 eq.) and cesium carbonate (163 mg, 0.50 mmol, 2 eq.) in DMF (1 mL), and the mixture was heated at 100 °C for 24 hours. Upon cooling to room temperature, the solution was diluted with EtOAc and washed with 5% w/w lithium chloride solution. The aqueous layer was back-washed with a further portion of EtOAc, and the combined organic layers were washed with brine, dried over sodium sulfate, and concentrated under vacuum. The crude mixture was purified by column chromatography (0-10% EtOAc in pentane) to afford the compound **17** as a white solid (49.9 mg, 0.14 mmol, 57%).

R*f* = 0.59 (15% EtOAc in pentane).

Melting point: 75-77°C

^{1}H NMR (500 MHz, $CDCl_3$) δ [ppm] 7.65 – 7.59 (m, 2H, H-C10), 7.47 – 7.41 (m, 2H, H-C3), 7.17 – 7.11 (m, 2H, H-C2), 7.00 – 6.94 (m, 2H, H-C9), 4.05 (t, $^3J_{HF}$ = 11.0 Hz, 2H, H-C7), 3.33 (t, $^3J_{HF}$ = 16.3 Hz, 2H, H-C5).

^{19}F NMR (470 MHz, $CDCl_3$) δ [ppm] -103.65 (tt, $^3J_{FH}$ = 16.3, $^3J_{FH}$ = 11.0 Hz, 2F, F-C6).

^{19}F{^{1}H} NMR (377 MHz, $CDCl_3$) δ [ppm] -103.65 (s, 2F, F-C6).

^{13}C{^{1}H} NMR (126 MHz, $CDCl_3$) δ [ppm] 160.8 (s, C8), 134.3 (s, C10), 132.1 (s, C2), 132.0 (s, C3), 131.2 (t, $^3J_{CF}$ = 4.9 Hz, C4), 122.1 (s, C1), 120.9 (t, $^1J_{CF}$ = 244.0 Hz, C6), 118.8 (s, C12), 115.5 (s, C9), 105.7 (s, C11), 67.5 (t, $^2J_{CF}$ = 35.1 Hz, C7), 39.7 (t, $^2J_{CF}$ = 24.6 Hz, C5).

ESI-MS: (*m/z*) requires: [$(C_{16}H_{12}NOF_2BrNa)^+$] = 373.9963, (*m/z*) found: [$(C_{16}H_{12}NOF_2BrNa)^+$] = 373.9964.

FT-IR ($\tilde{\nu}$ = cm^{-1})**:** 3102 (w), 2045 (w), 2972 (w), 2943 (w), 2224 (m), 1908 (w), 1606 (s), 1577 (m), 1508 (s), 1488 (s), 1456 (w), 1430 (w), 1415 (w), 1406 (w), 1345 (w),

1304 (m), 1258 (s), 1222 (s), 1172 (s), 1161 (s), 1128 (w), 1109 (m), 1070 (s), 1032 (s), 1011 (s), 971 (w), 958 (m), 943 (w), 896 (m), 852 (w), 845 (w), 837 (s), 817 (m), 784 (s), 733 (s), 712 (w), 667 (m) cm^{-1}.

((4-(3-Bromo-2,2-difluoropropyl)phenyl)ethynyl)trimethylsilane (18)

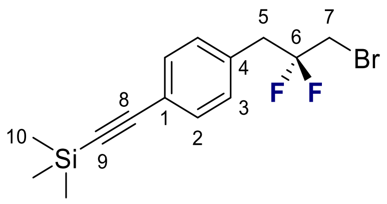

The reaction was performed in accordance with previous literature precedent. [28]

A mixture of 1-bromo-4-(3-bromo-2,2-difluoropropyl)benzene **2a** (78.5 mg, 0.25 mmol, 1.0 eq.), *bis*(triphenylphosphine)palladium(II)-dichloride (17.5 mg, 0.025 mmol, 10 mol%), trimethylsilylacetylene (36.8 mg, 53.4 µL, 0.38 mmol, 1.5 eq.) and copper(I) iodide (9.50 mg, 0.05 mmol, 20 mol%) in diisopropylamine (0.5 mL) was stirred at 80 °C for 3 hours. The resulting mixture was passed through a pad of silica gel using pentane as the eluant. The filtrate was concentrated under vacuum and further purified by column chromatography (100% pentane) to afford compound **18** as a yellow oil (72.0 mg, 0.22 mmol, 87%).

R*f* = 0.20 (pentane).

^{1}H NMR (599 MHz, $CDCl_3$) δ[ppm] = 7.44 (m, 2H, H-C2), 7.26 (m, 2H, H-C3), 3.37 (t, $^{3}J_{HF}$ = 12.6 Hz, 2H, H-C7), 3.35 (t, $^{3}J_{HF}$ = 15.6 Hz, 2H, H-C5), 0.25 (s, 9H, H-C10).

^{19}F NMR (564 MHz, $CDCl_3$) δ[ppm] = -96.86 (tt, $^{3}J_{FH}$ = 15.5, $^{3}J_{FH}$ = 12.6 Hz, 2F, F-C6).

^{19}F{^{1}H} NMR (564 MHz, $CDCl_3$) δ[ppm] = -96.86 (s, 2F, F-C6).

^{13}C{^{1}H} NMR (151 MHz, $CDCl_3$) δ[ppm] = 132.7 (t, $^{3}J_{C,F}$ = 4.8 Hz, C4), 132.4 (s, C2), 130.3 (s, C3), 122.9 (s, C1), 120.7 (t, $^{1}J_{C,F}$ = 244.7 Hz, C6), 104.6 (s, C8), 95.1 (s, C9), 40.7 (t, $^{2}J_{CF}$ = 25.1 Hz, C5), 30.5 (t, $^{2}J_{CF}$ = 33.5 Hz, C7), 0.1 (s, C10).

ESI-MS: (*m/z*) requires: [$(C_{14}H_{17}F_2SiBr)^+$] = 330.0246, (*m/z*) found: [$(C_{14}H_{17}F_2SiBr)^+$] = 330.0248.

FT-IR ($\tilde{\nu}$ = cm^{-1})**:** 2960 (w), 2159 (w), 1509 (w), 1424 (w), 1344 (w), 1299 (w), 1250 (m), 1214 (m), 1139 (w), 1111 (w), 1069 (m), 1028 (s), 861 (s), 836 (s), 803 (m), 784 (m), 759 (s), 725 (w), 695 (m).

2,2-Difluoro-3-(4-(trifluoromethyl)phenyl)propan-1-aminium chloride (20)

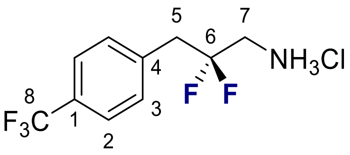

Compound **20** was prepared in accordance with previous literature precedent. [29]

1-(3-bromo-2,2-difluoropropyl)-4-(trifluoromethyl)benzene **2h** (758 mg, 2.50 mmol, 1.0 eq.) was dissolved in DMF (2.5 mL), and sodium azide (325 mg, 5.00 mmol, 2.0 eq.) was added. The resulting mixture was stirred at 110°C for 18 hours. Upon cooling to room temperature, the mixture was partitioned between EtOAc and 5% w/w lithium chloride solution. The aqueous layer was back-washed with a further portion of EtOAc, and the combined organic layers were washed with brine, dried over sodium sulfate, and concentrated under reduced pressure. The crude azide was dissolved in ethanol (5 mL), and palladium hydroxide (10% w/w, 351 mg, 0.25 mmol, 10 mol%) was added, followed by 1M HCl solution (2.5 mL). The resulting mixture was stirred under a hydrogen atmosphere for 24 hours. The solution was filtered through celite and concentrated under vacuum. The crude product was washed with acetonitrile to afford compound **20** as a yellow solid (503 mg, 1.76 mmol, 70%).

Melting point: >250°C

^{1}H NMR (599 MHz, DMSO-*d*6) δ [ppm] δ 8.86 (s, 3H, NH), 7.73 (d, $^3J_{HH}$ = 8.0 Hz, 2H, H-C2), 7.55 (d, $^3J_{HH}$ = 8.0 Hz, 2H, H-C3), 3.57 (t, $^3J_{HF}$ = 17.9 Hz, 2H, H-C5), 3.40 (t, $^3J_{HF}$ = 15.2 Hz, 2H, H-C7).

^{19}F NMR (564 MHz, DMSO-*d*6) δ [ppm] -61.07 (s, 3F, F-C8), -101.82 (app. p, $^3J_{HF}$ = 16.7 Hz, 2F, F-C6).

^{19}F{^{1}H} NMR (564 MHz, DMSO-*d*6) δ [ppm] -61.07 (s, 3F, F-C8), -101.81 (s, 2F, F-C6).

^{13}C{^{1}H} NMR (151 MHz, DMSO-*d*6) δ [ppm] 136.8 (s, C4), 131.4 (s, C3), 128.2 (q, $^2J_{CF}$ = 31.8 Hz, C1), 125.2 (q, $^3J_{CF}$ = 3.8 Hz, C2), 124.4 (q, $^1J_{CF}$ = 272.0 Hz, C8), 120.6 (t, $^1J_{CF}$ = 244.2 Hz, C6), 42.1 (t, $^2J_{CF}$ = 26.3 Hz, C7), 39.5 (t, $^2J_{CF}$ = 23.3 Hz, C5).

ESI-MS: (*m/z*) requires: [($C_{10}H_{11}NF_5$)$^+$] = 240.0806, (*m/z*) found: [($C_{10}H_{11}NF_5$)$^+$] = 240.0802.

FT-IR ($\tilde{\nu}$ = cm^{-1})**:** 2985 (w), 1698 (m), 1600 (m), 1422 (m), 1369 (w), 1325 (s), 1163 (m), 1114 (s), 1067 (s), 1035 (w), 1021 (m), 936 (w), 899 (w), 856 (w), 833 (w), 810 (w), 795 (w), 684 (w) cm^{-1}.

1,3-*Bis*(2,2-difluoro-3-(4-(trifluoromethyl)phenyl)propyl)urea (20-2)

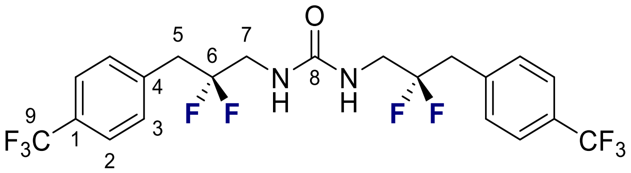

Compound **20-2** was prepared in accordance with previous literature precedent. [29]

2,2-Difluoro-3-(4-(trifluoromethyl)phenyl)propan-1-aminium chloride **20** (138 mg, 0.50 mmol, 1.0 eq.), carbonyldiimidazole (40.5 mg, 0.25 mmol, 0.5 eq.) and triethylamine (69.7 µL, 0.50 mmol, 1.0 eq.) were heated at 60°C in THF (1 mL) for 16 hours. Upon cooling to room temperature, the reaction mixture was partitioned between EtOAc and water. The aqueous layer was back-washed with an additional portion of EtOAc, and the combined organic layers were washed with brine, dried over sodium sulfate and concentrated under reduced pressure. The crude product was purified by column chromatography (0-50% EtOAc in pentane) to afford compound **20-2** as a white solid (102 mg, 0.20 mmol, 80%).

R*f* = 0.37 (50% EtOAc in pentane).

Melting point: 204-206°C

^{1}H NMR (599 MHz, DMSO-*d*6) δ[ppm] 7.68 (d, $^{3}J_{HH}$ = 7.9 Hz, 4H, H-C2), 7.51 (d, $^{3}J_{HH}$ = 8.0 Hz, 4H, H-C3), 6.48 (t, $^{3}J_{HH}$ = 6.3 Hz, 2H, NH), 3.53 (td, $^{3}J_{HF}$ = 14.7, $^{3}J_{HH}$ = 6.3 Hz, 4H, H-C7), 3.30 (t, $^{3}J_{HF}$ = 17.6 Hz, 4H, H-C5).

^{19}F NMR (564 MHz, DMSO-*d*6) δ[ppm] -56.25 (s, 6F, F-C9), -97.56 – -97.78 (app. p, $^{3}J_{HF}$ = 16.4 Hz, 4F, F-C6).

^{19}F{^{1}H} NMR (377 MHz, DMSO-*d*6) δ[ppm] -56.25 (s, 6F, F-C9), -97.66 (s, 4F, F-C6).

^{13}C{^{1}H} NMR (151 MHz, DMSO-*d*6) δ[ppm] 158.0 (s, C8), 138.1 (s, C4), 131.8 (s, C3), 128.4 (q, $^{2}J_{CF}$ = 31.9 Hz, C1), 125.5 (q, $^{3}J_{CF}$ = 3.8 Hz, C2), 124.7 (q, $^{1}J_{CF}$ = 272.0 Hz, C9), 122.7 (t, $^{1}J_{CF}$ = 243.3 Hz, C6), 43.8 (t, $^{2}J_{CF}$ = 27.7 Hz, C7), 39.9 (t, $^{2}J_{CF}$ = 24.3 Hz, C5).

ESI-MS: (*m/z*) requires: $[(C_{21}H_{18}N_2OF_{10}Na)^+]$ = 527.1150, (*m/z*) found: $[(C_{21}H_{18}N_2OF_{10}Na)^+]$ = 527.1152.

FT-IR ($\tilde{\nu}$ = cm^{-1})**:** 3337 (m), 2923 (w), 2852 (w), 1631 (m), 1581 (s), 1435 (w), 1420 (w), 1396 (w), 1354 (w), 1321 (s), 1299 (w), 1260 (w), 1225 (w), 1209 (m), 1197 (m), 1173 (m), 1124 (s), 1113 (s), 1063 (s), 1019 (m), 1003 (w), 961 (w), 943 (w), 887 (m), 870 (m), 860 (m), 830 (w), 805 (s), 740 (m), 693 (m) cm^{-1}.

1,3-*Bis*(2,2-difluoro-3-(4-(trifluoromethyl)phenyl)propyl)pyrimidine-2,4,6(1*H*,3*H*,5*H*)-trione (21)

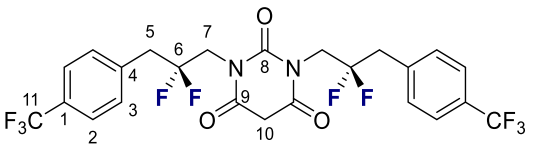

Compound **21** was prepared in accordance with previous literature precedent. [29]

A solution of 1,3-*bis*(2,2-difluoro-3-(4-(trifluoromethyl)phenyl)propyl)urea **20-2** (30.4 mg, 0.10 mmol, 1.0 eq.) in DCM (0.5 mL) was cooled to 0°C, and malonyl chloride (5.80 µL, 0.10 mmol, 1.0 eq.) was added. The solution was brought to room temperature and stirred for a further 2 hours, at which point it was diluted with EtOAc and washed with water. The aqueous layer was back-washed with an additional portion of EtOAc, and the combined organic layers were washed with brine, dried over sodium sulfate and concentrated under vacuum. The crude product was loaded onto a silica plug, which was washed successively with pentane and diethyl ether. Subsequent washes of 10% MeOH/DCM then enabled elution of the product, which was concentrated under vacuum to afford compound **21** as a yellow solid (29.5 mg, 86.0 µmol, 86%).

$\mathbf{R}_f$ = 0.07 (50% EtOAc in pentane).

Melting point: 137-139°C

^{1}H NMR (599 MHz, $CDCl_3$) δ [ppm] 7.60 (d, $^3J_{HH}$ = 8.1 Hz, 4H, H-C2), 7.42 (d, $^3J_{HH}$ = 8.0 Hz, 4H. H-C3), 4.36 (t, $^3J_{HF}$ = 13.7 Hz, 4H, H-C7), 3.76 (s, 2H, H-C10), 3.29 (t, $^3J_{HF}$ = 16.6 Hz, 4H, H-C5).

^{19}F NMR (377 MHz, $CDCl_3$) δ [ppm] -62.67 (s, 6F, F-C11), -100.20 (app. p, $^3J_{HF}$ = 15.1 Hz, 4F, F-C6).

^{19}F NMR (377 MHz, $CDCl_3$) δ [ppm] -62.68 (s, 6F, F-C11), -100.19 (s, 4F, F-C6).

^{13}C{^{1}H} NMR (151 MHz, $CDCl_3$) δ [ppm] 163.8 (s, C9), 151.1 (s, C8), 135.7 (s, C4), 130.9 (s, C3), 130.3 (q, $^{2}J_{CF}$ = 32.6 Hz, C1), 125.7 (q, $^{3}J_{CF}$ = 3.8 Hz, C2), 124.2 (q, $^{1}J_{CF}$ = 272.1 Hz, C11), 120.3 (t, $^{1}J_{CF}$ = 247.5 Hz, C6), 44.7 (t, $^{2}J_{CF}$ = 27.3 Hz, C7), 41.9 (t, $^{2}J_{CF}$ = 24.5 Hz, C5), 39.6 (s, C10).

ESI-MS: (*m/z*) requires: [$(C_{24}H_{18}N_2O_3F_{10}Na)^+$] = 595.1050, (*m/z*) found: [$(C_{24}H_{18}N_2O_3F_{10}Na)^+$] = 595.1062.

FT-IR ($\tilde{\nu}$ = cm^{-1})**:** 2985 (w), 1698 (m), 1600 (m), 1422 (m), 1369 (w), 1325 (s), 1163 (m), 1114 (s), 1068 (s), 1035 (w), 1021 (w), 936 (w), 899 (w), 856 (w), 833 (w), 810 (m), 795 (m), 684 (w) cm^{-1}.

4. X-Ray Diffraction

X-Ray diffraction: Data sets for compound **2n** were collected with a Bruker D8 Venture CMOS diffractometer. Programs used: data collection: APEX3 V2016.1-0 (Bruker AXS Inc., **2016**); cell refinement: SAINT V8.37A (Bruker AXS Inc., **2015**); data reduction: SAINT V8.37A (Bruker AXS Inc., **2015**); absorption correction, SADABS V2014/7 (Bruker AXS Inc., **2014**); structure solution *SHELXT-2015* (Sheldrick, G. M. *Acta Cryst.,* **2015**, *A71*, 3-8); structure refinement *SHELXL-2015* (Sheldrick, G. M. *Acta Cryst.,* **2015**, *C71* (1), 3-8) and graphics, *XP* (Version 5.1, Bruker AXS Inc., Madison, Wisconsin, USA, **1998**). *R*-values are given for observed reflections, and wR^2 values are given for all reflections.

X-ray crystal structure analysis of 2n (gil9967): A colorless plate-like specimen of $C_{17}H_{12}BrF_2NO_2$, approximate dimensions 0.043 mm x 0.085 mm x 0.149 mm, was used for the X-ray crystallographic analysis. The X-ray intensity data were measured on a Bruker D8 Venture Bruker D8 Venture Photon III Diffractometer system equipped with a micro focus tube MoKα (MoKα, λ = 0.71073 Å) and a MX mirror monochromator. A total of 831 frames were collected. The total exposure time was 4.62 hours. The frames were integrated with the Bruker SAINT software package using a narrow-frame algorithm. The integration of the data using a triclinic unit cell yielded a total of 14235 reflections to a maximum θ angle of 25.35° (0.83 Å resolution), of which 2620 were independent (average redundancy 5.433, completeness = 97.5%, R_{int} = 5.45%, R_{sig} = 3.91%) and 2325 (88.74%) were greater than $2\sigma(F^2)$. The final cell constants of a = 4.7731(8) Å, b = 11.3543(17) Å, c = 14.650(2) Å, α = 67.712(5)°, β = 85.665(5)°, γ = 88.114(6)°, volume = 732.5(2) Å^3, are based upon the refinement of the XYZ-centroids of 6241 reflections above 20 σ(I) with 5.739° < 2θ < 53.69°. Data were corrected for absorption effects using the multi-scan method (SADABS). The ratio of minimum to maximum apparent transmission was 0.770. The calculated minimum and maximum transmission coefficients (based on crystal size) are 0.6770 and 0.8880. The structure was solved and refined using the Bruker SHELXTL Software Package, using the space group *P*-1, with Z = 2 for the formula unit, $C_{17}H_{12}BrF_2NO_2$. The final anisotropic full-matrix least-squares refinement on F^2 with 208 variables converged at R1 = 3.33%, for the observed data and wR2 = 7.79% for all data. The goodness-of-fit was 1.068. The largest peak in the final difference electron density synthesis was 0.613 $e^-/Å^3$ and the

largest hole was -0.485 $e^-/Å^3$ with an RMS deviation of 0.083 $e^-/Å^3$. On the basis of the final model, the calculated density was 1.724 g/cm^3 and F(000), 380 e^-. CCDC number: 2055892.

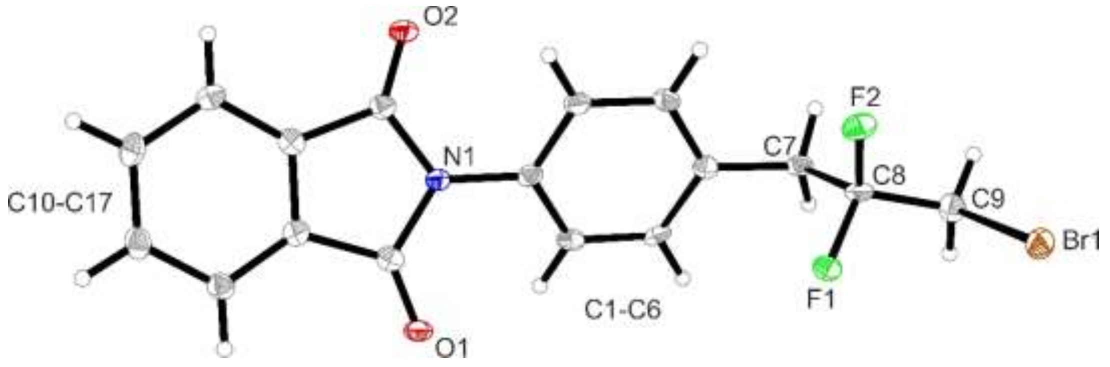

Figure 1: Crystal structure of compound **2n**.

Thermal ellipsoids are shown at 50% probability.

1. *APEX3* (**2016**), *SAINT* (**2015**) and *SADABS* (**2015**), Bruker AXS Inc., Madison, Wisconsin, USA.
2. G. M. Sheldrick, *Acta Cryst.* 2015, **A71**, 3.
3. G. M. Sheldrick, *Acta Cryst.*, 2015, **C71(1)**, 3.
4. *XP – Interactive molecular graphics, Version 5.1,* Bruker AXS Inc., Madison, Wisconsin, USA, **1998**.

5. NMR-Spectra

1-(4-(Piperidin-1-ylsulfonyl)phenyl)ethan-1-one (S4)

^{1}H NMR (599 MHz, $CDCl_3$):

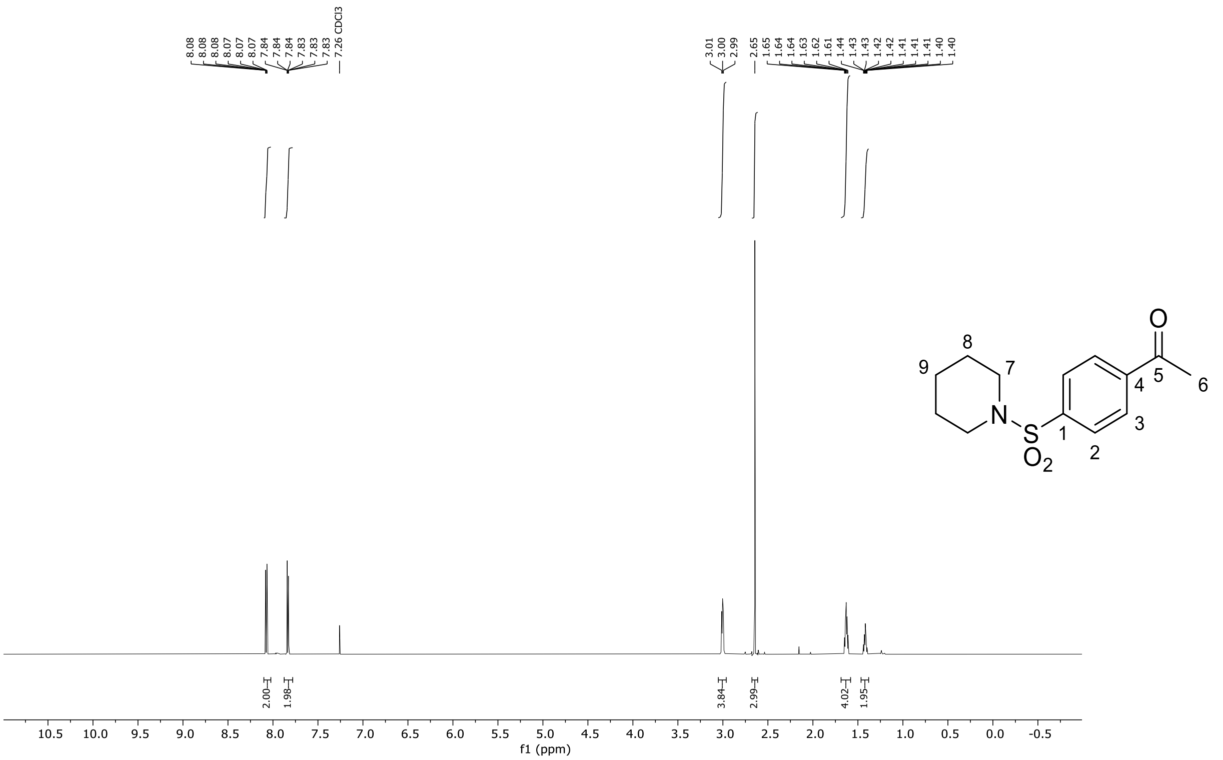

$^{13}C\{^1H\}$ NMR (151 MHz, $CDCl_3$):

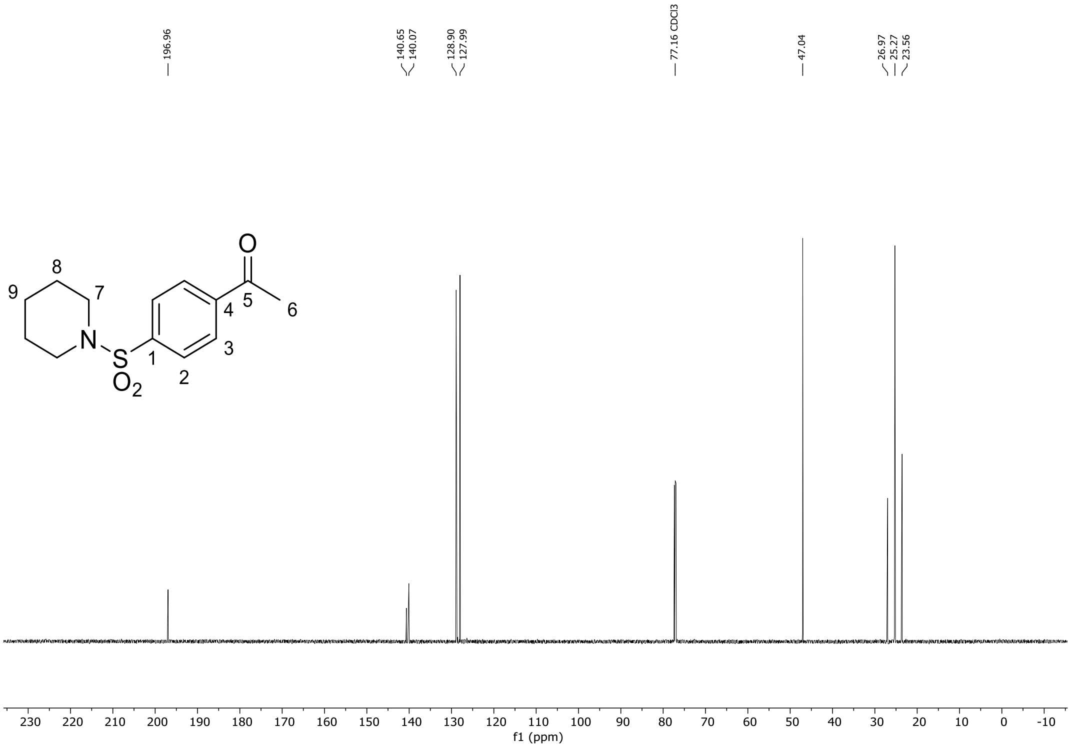

2-Bromo-1-fluoro-4-(prop-1-en-2-yl)benzene (S17)

^{1}H NMR (599 MHz, $CDCl_3$):

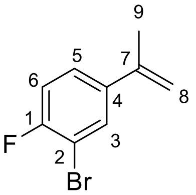

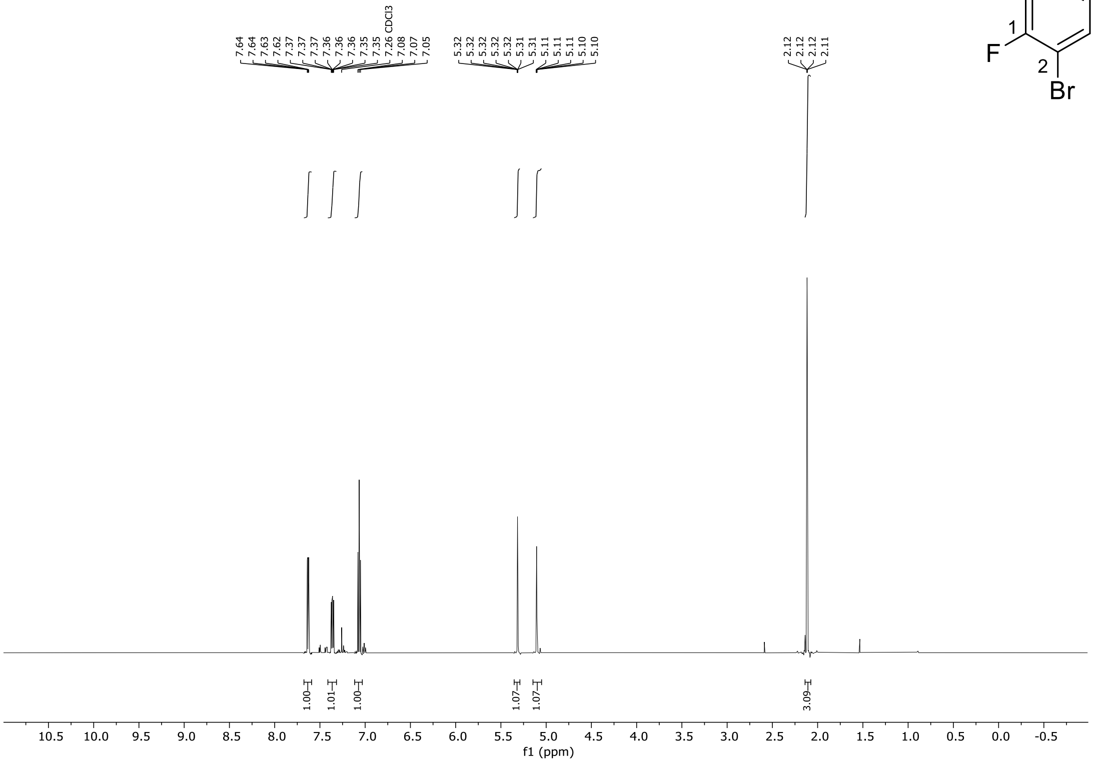

^{13}C{^{1}H} NMR (151 MHz, $CDCl_3$):

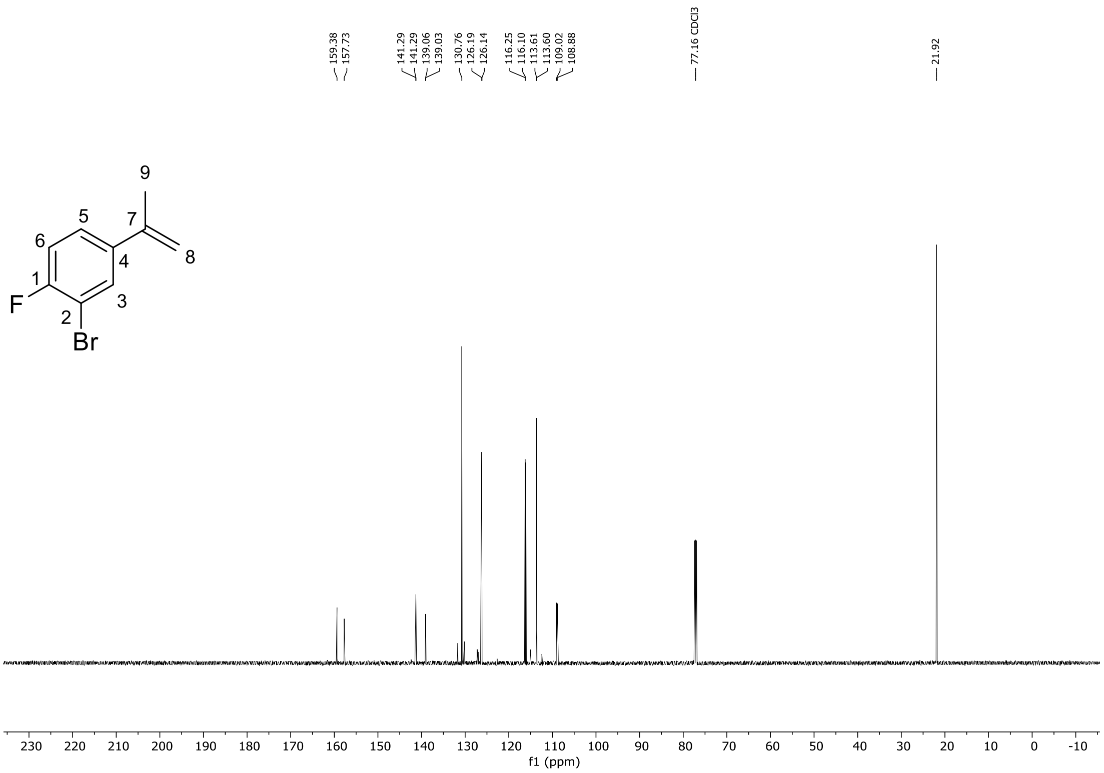

^{19}F NMR (564 MHz, $CDCl_3$):

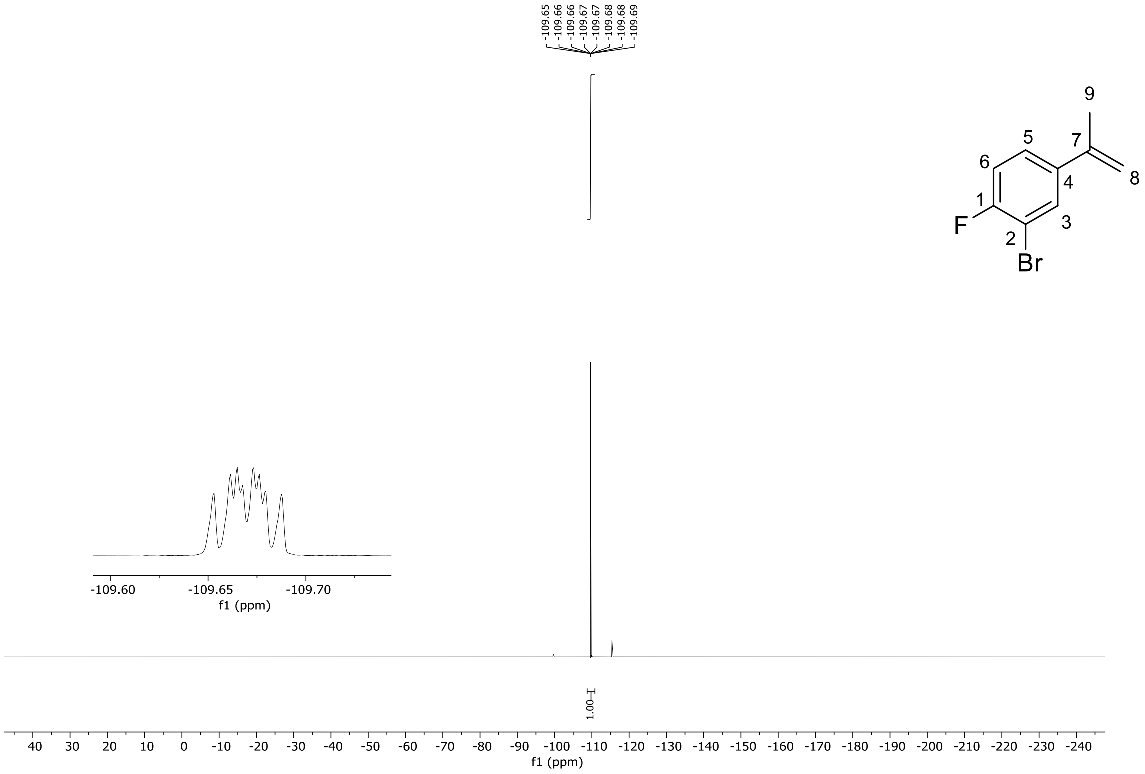

^{19}F{^{1}H} NMR (564 MHz, $CDCl_3$):

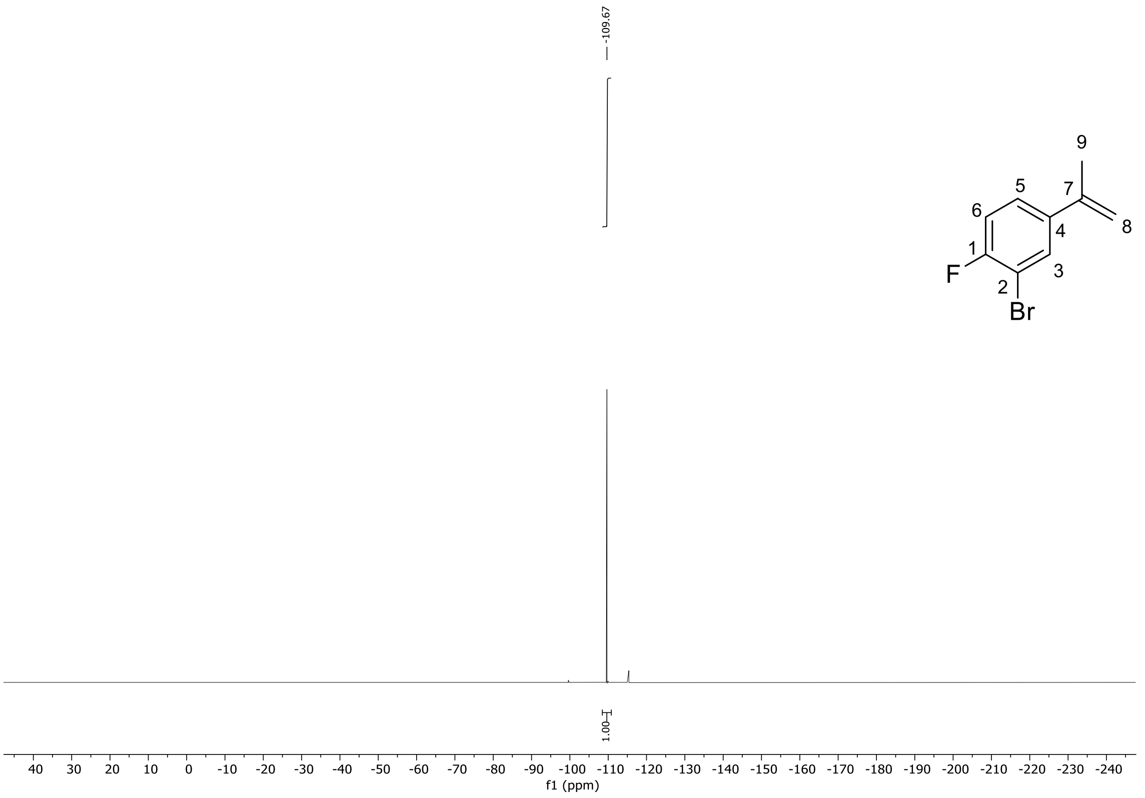

1-((4-(Prop-1-en-2-yl)phenyl)sulfonyl)piperidine (S18)

^{1}H NMR (500 MHz, $CDCl_3$):

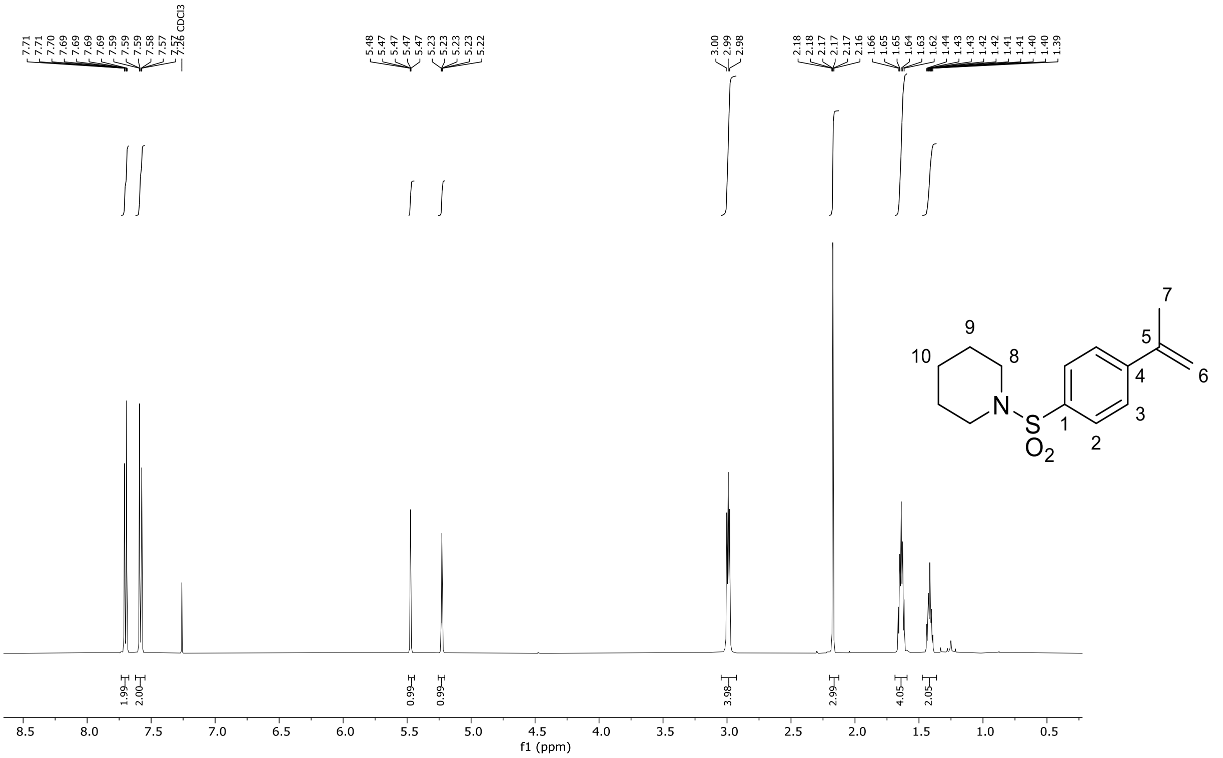

^{13}C NMR (126 MHz, $CDCl_3$):

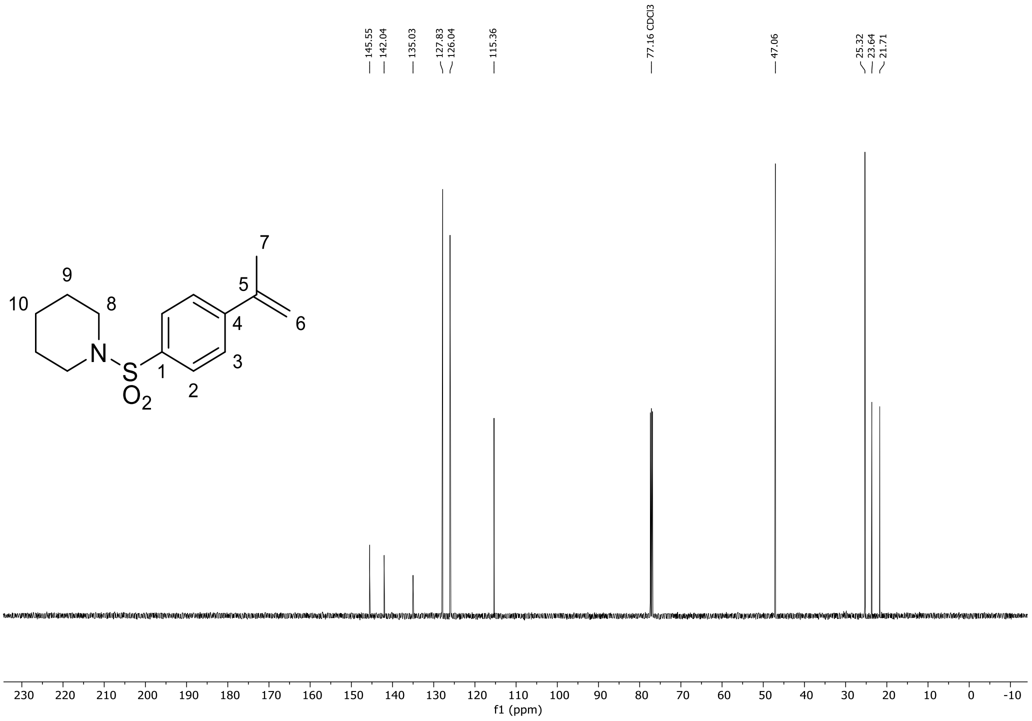

2-(4-(Prop-1-en-2-yl)phenyl)isoindoline-1,3-dione (S20)

^{1}H NMR (500 MHz, $CDCl_3$):

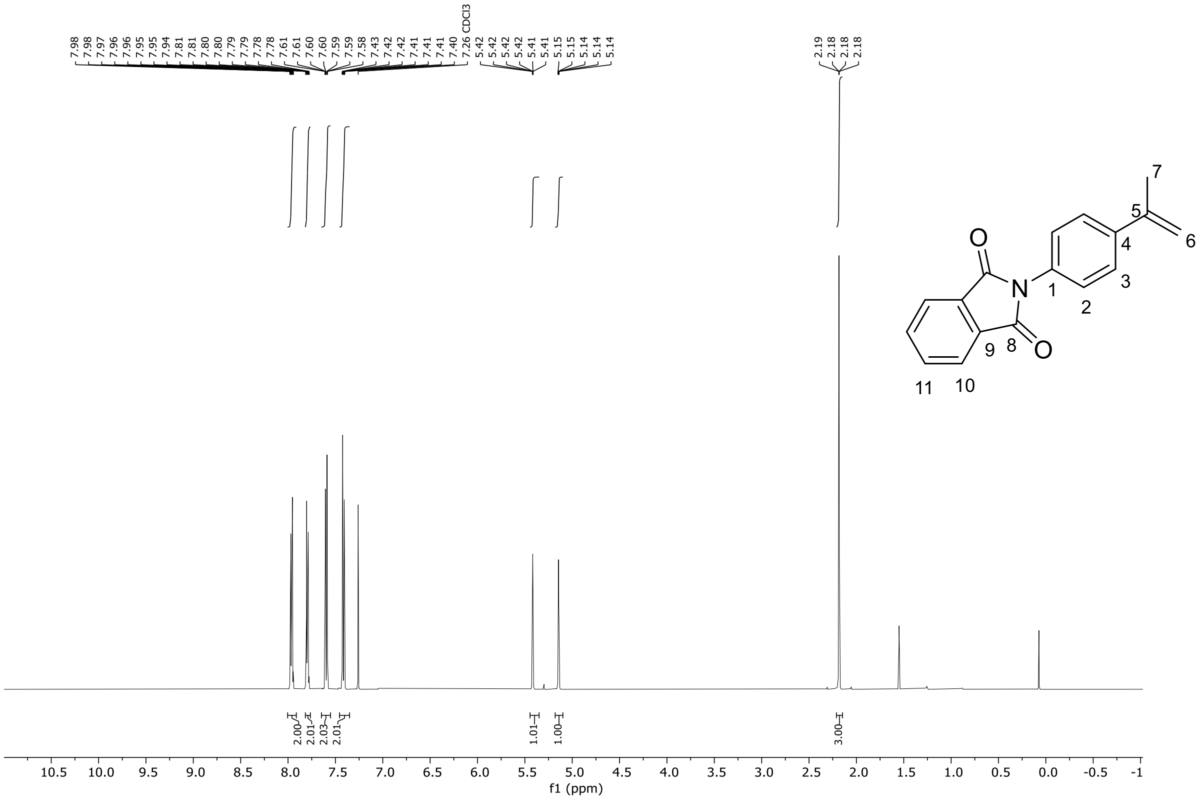

^{13}C NMR (126 MHz, $CDCl_3$):

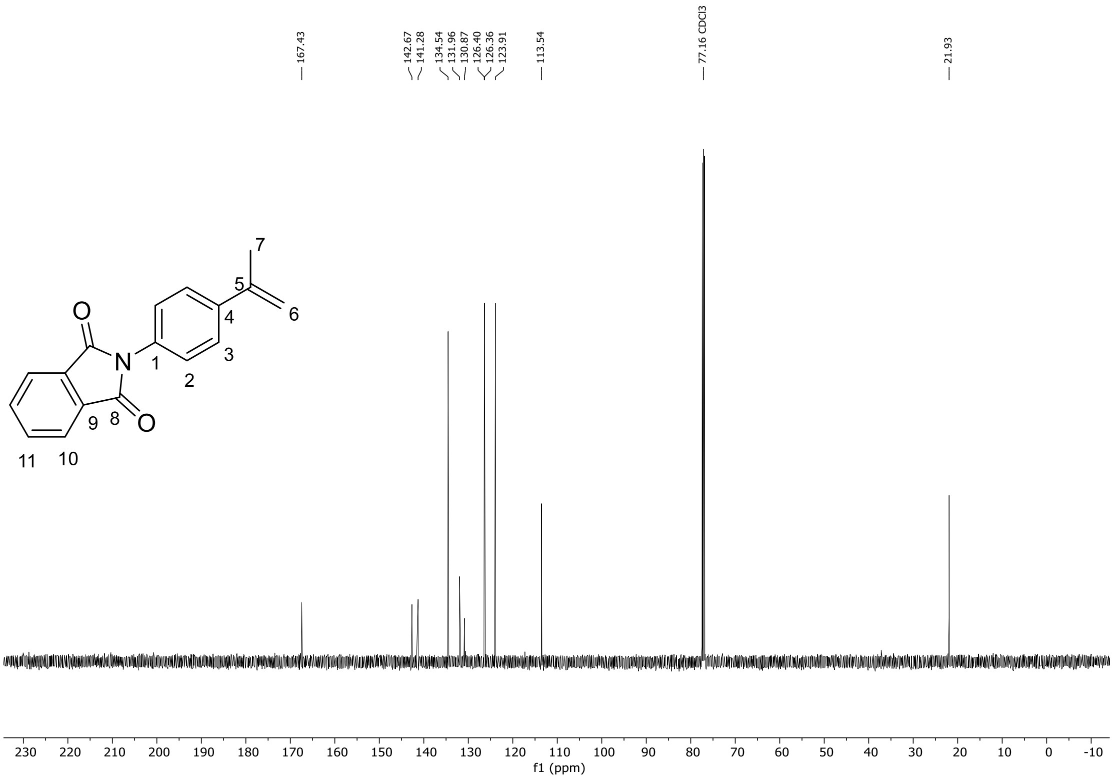

2,2,2-Trifluoro-1-(4-(prop-1-en-2-yl)phenyl)ethan-1-one (S21)

^{1}H NMR (599 MHz, $CDCl_3$):

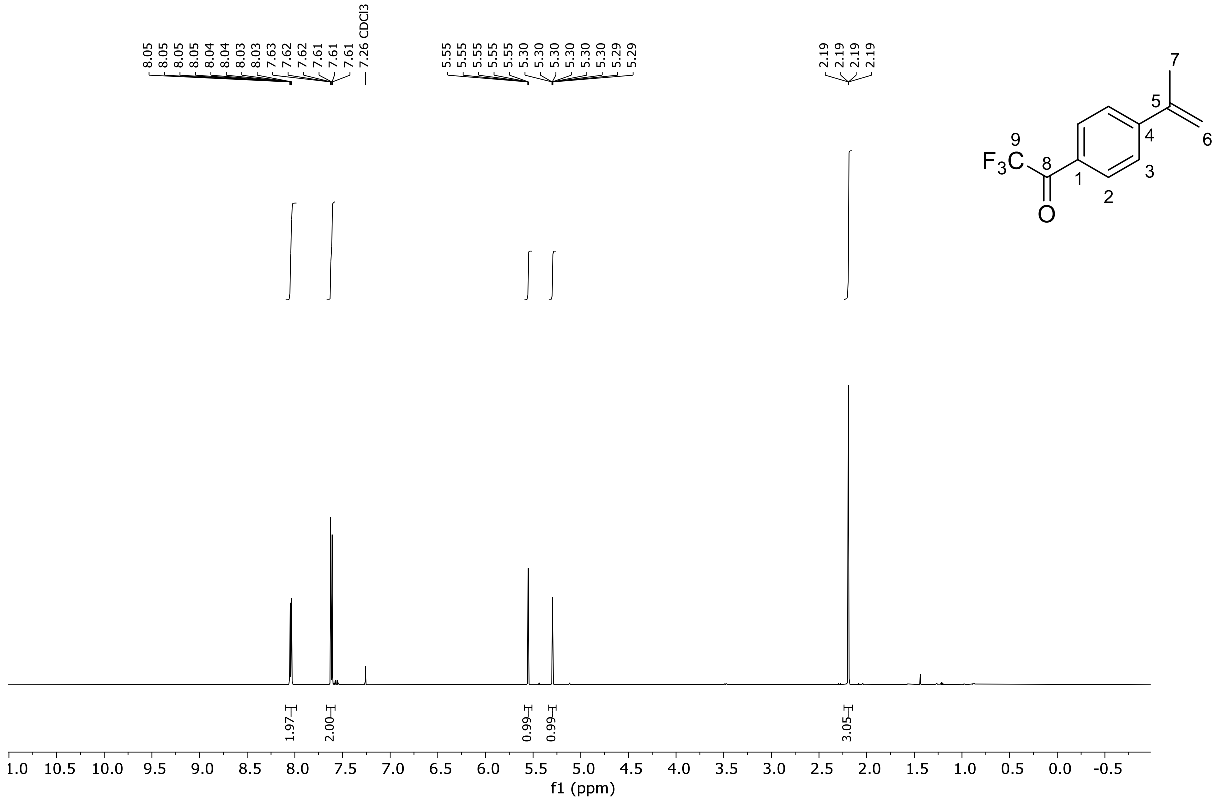

^{13}C NMR (151 MHz, $CDCl_3$):

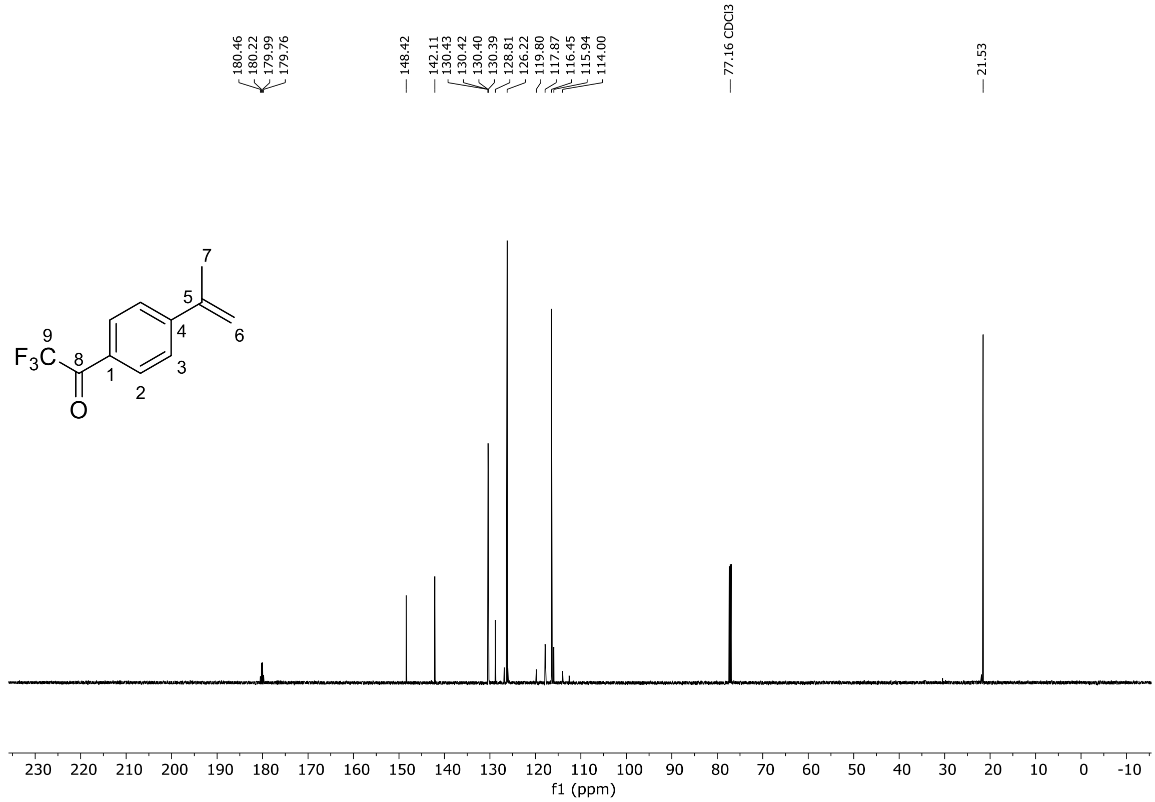

^{19}F NMR (376 MHz, $CDCl_3$):

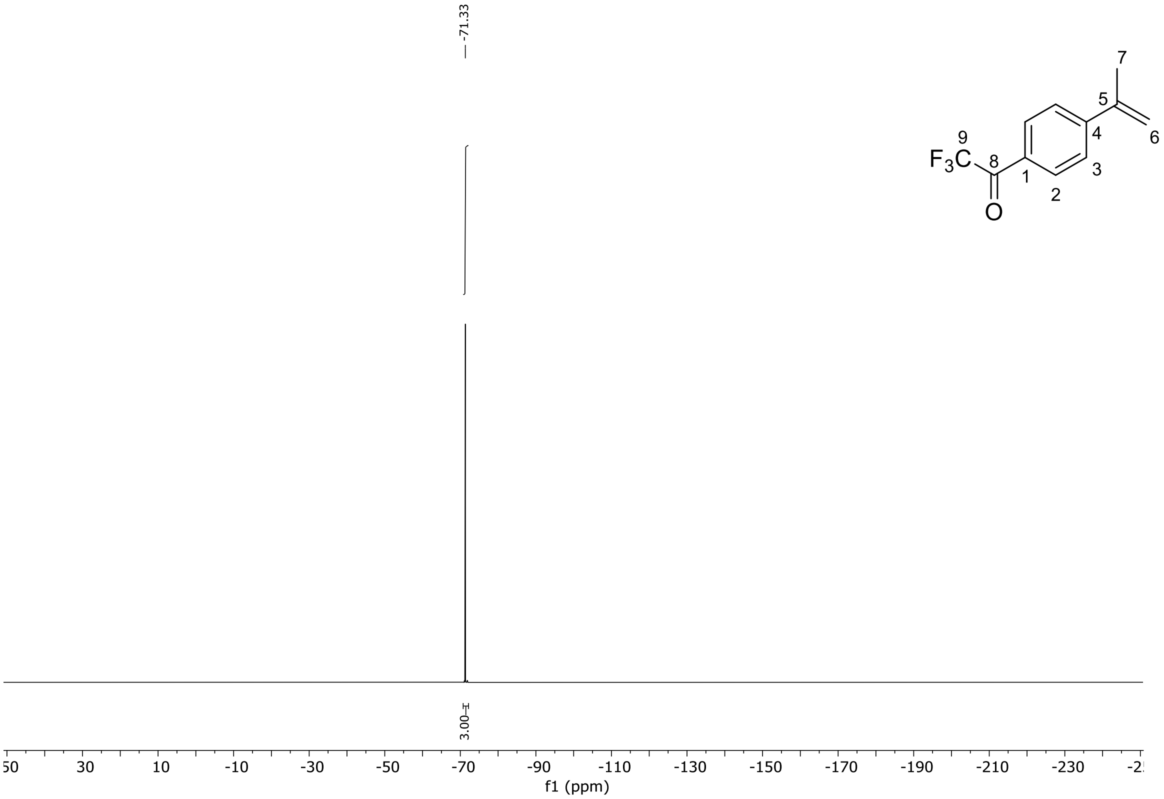

^{19}F{^{1}H} NMR (376 MHz, $CDCl_3$):

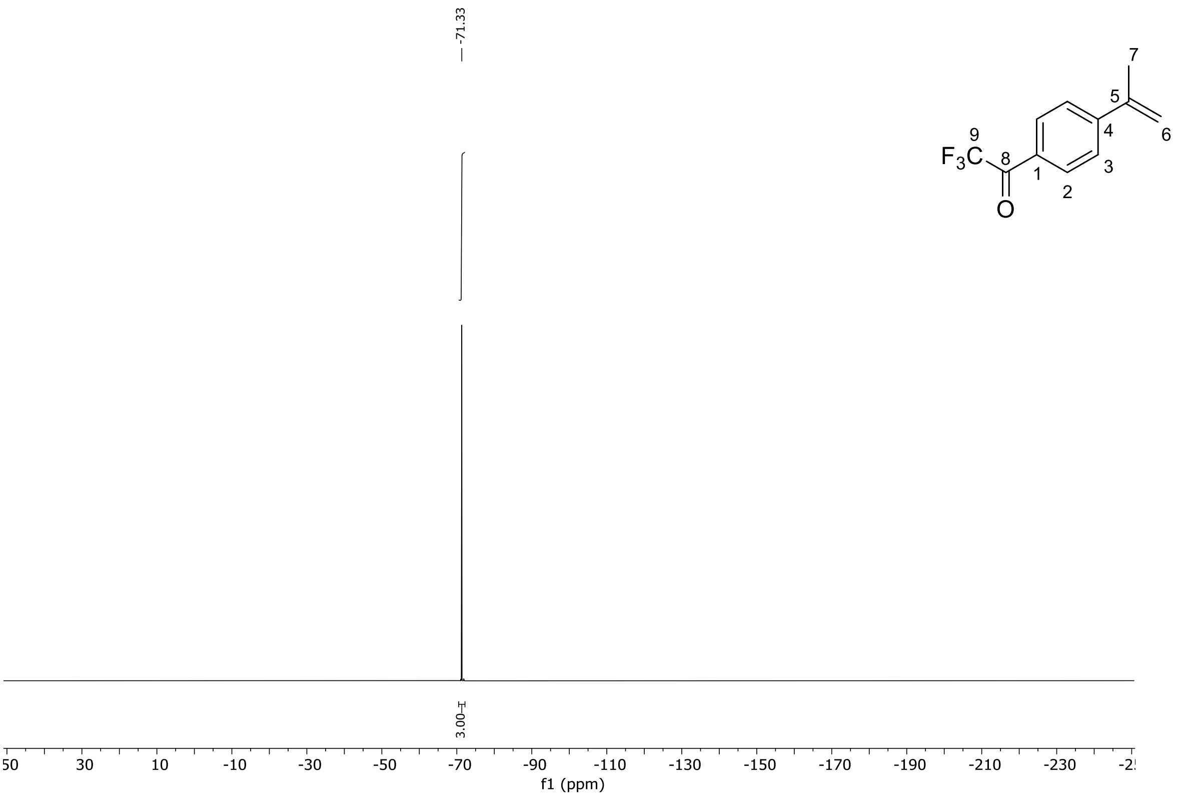

1-(Prop-1-en-2-yl)-4-(3,3,3-trifluoroprop-1-en-2-yl)benzene (S22)

¹H NMR (599 MHz, $CDCl_3$):

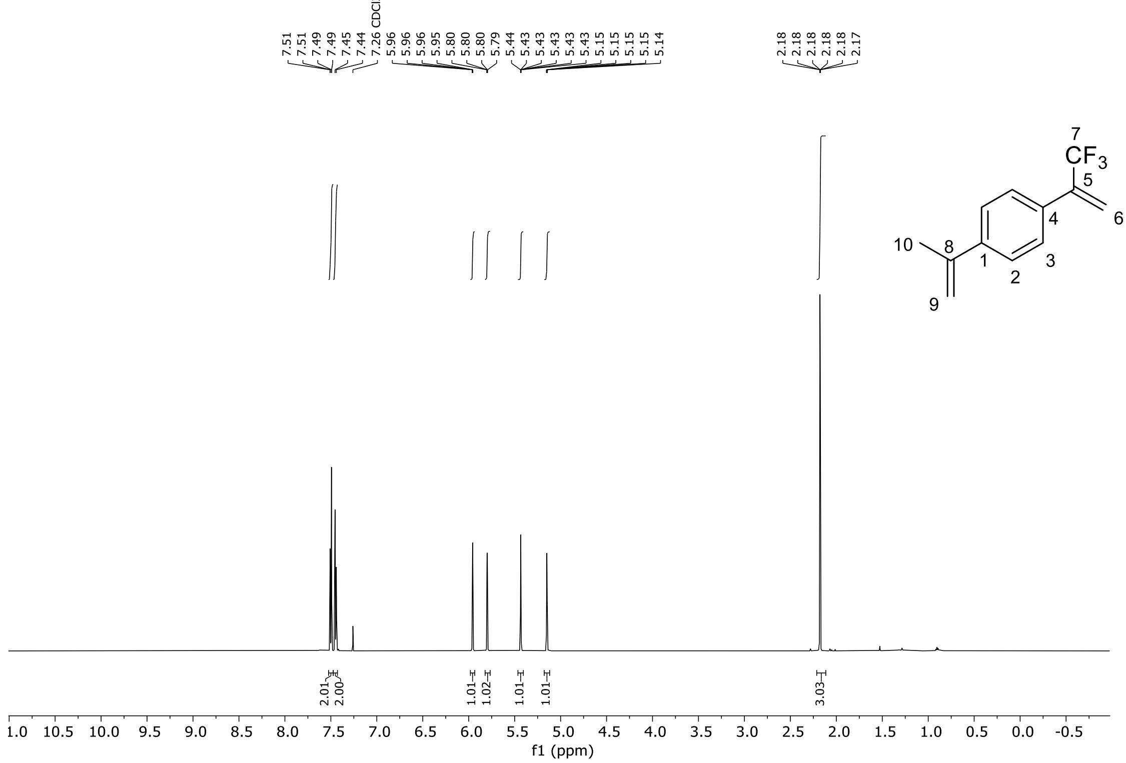

¹³C NMR (151 MHz, $CDCl_3$):

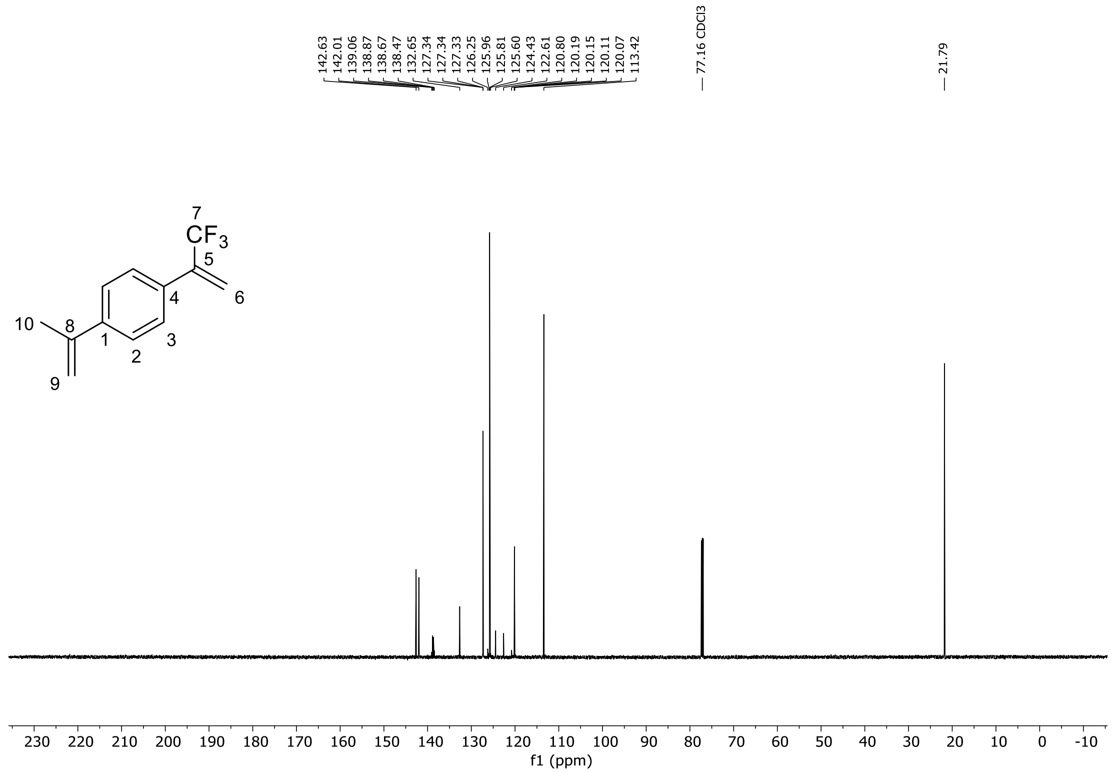

^{19}F NMR (376 MHz, $CDCl_3$):

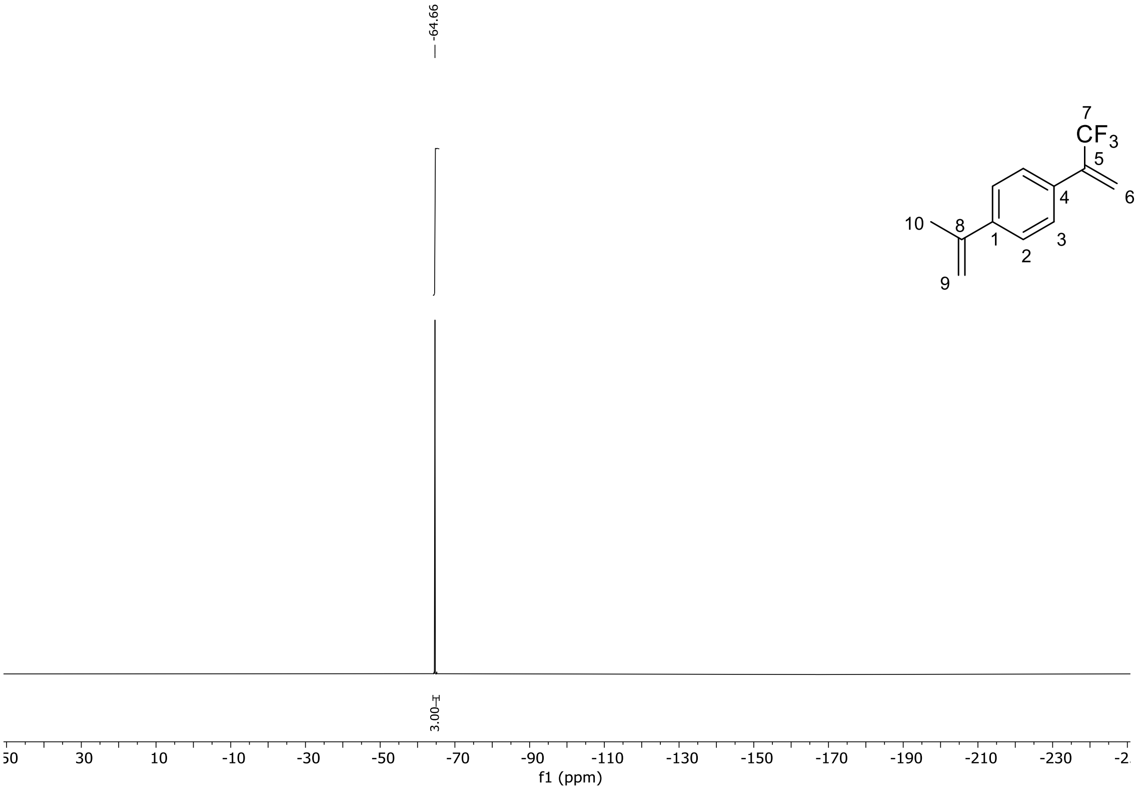

^{19}F{^{1}H} NMR (376 MHz, $CDCl_3$):

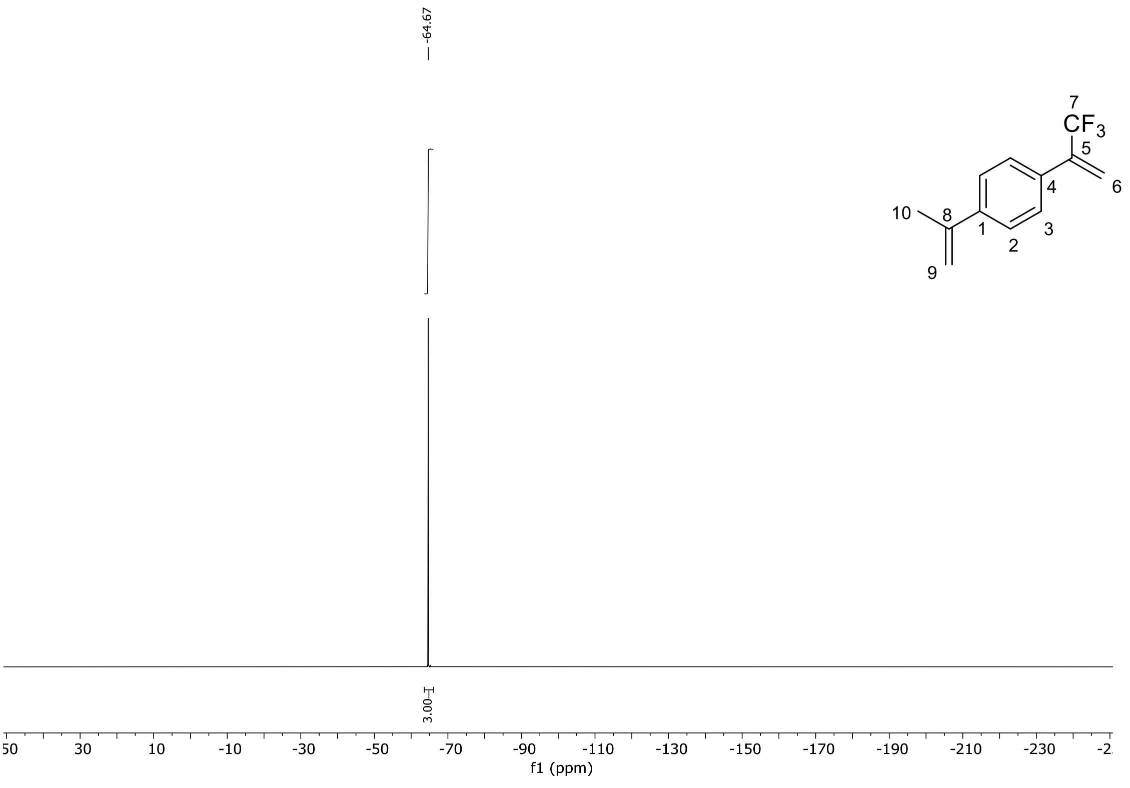

Methyl (*E*)-3-(4-(prop-1-en-2-yl)phenyl)acrylate (S23)

^{1}H NMR (500 MHz, $CDCl_3$):

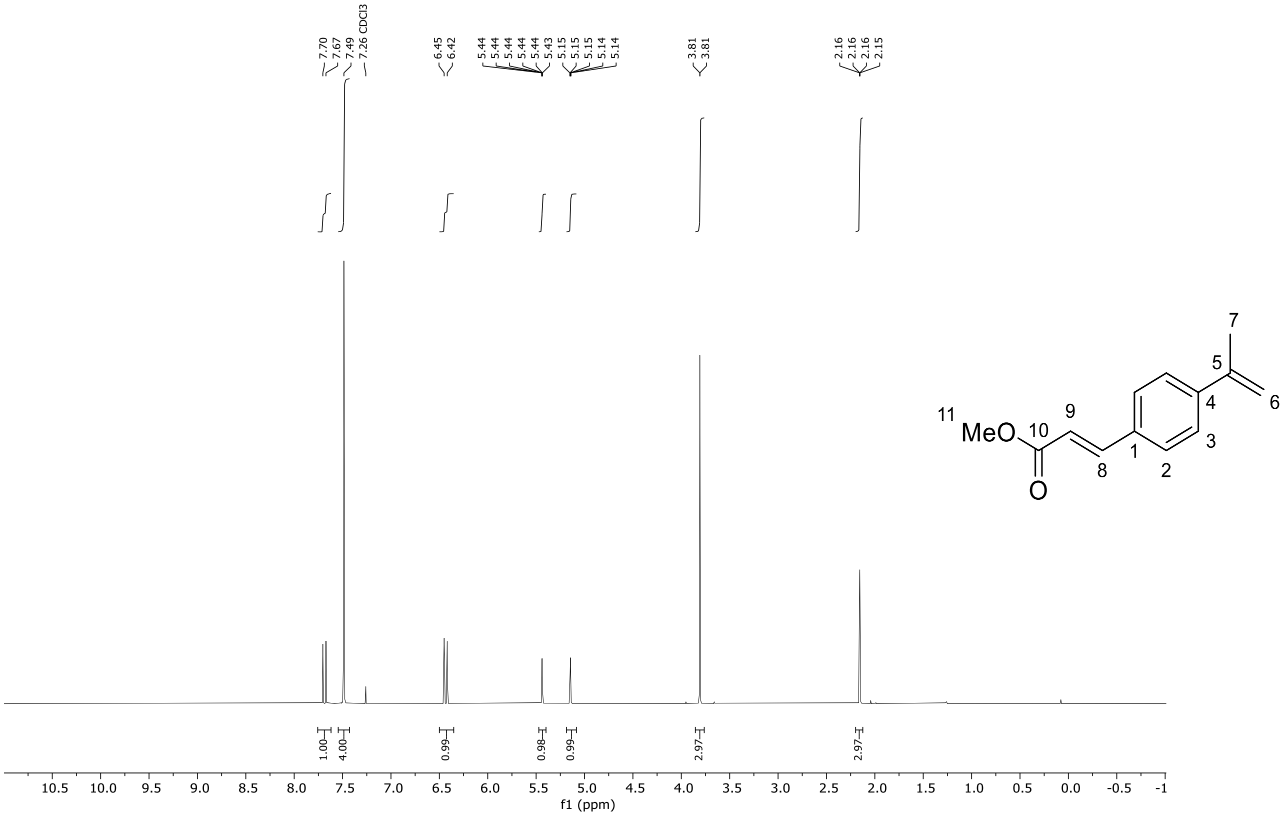

^{13}C NMR (126 MHz, $CDCl_3$):

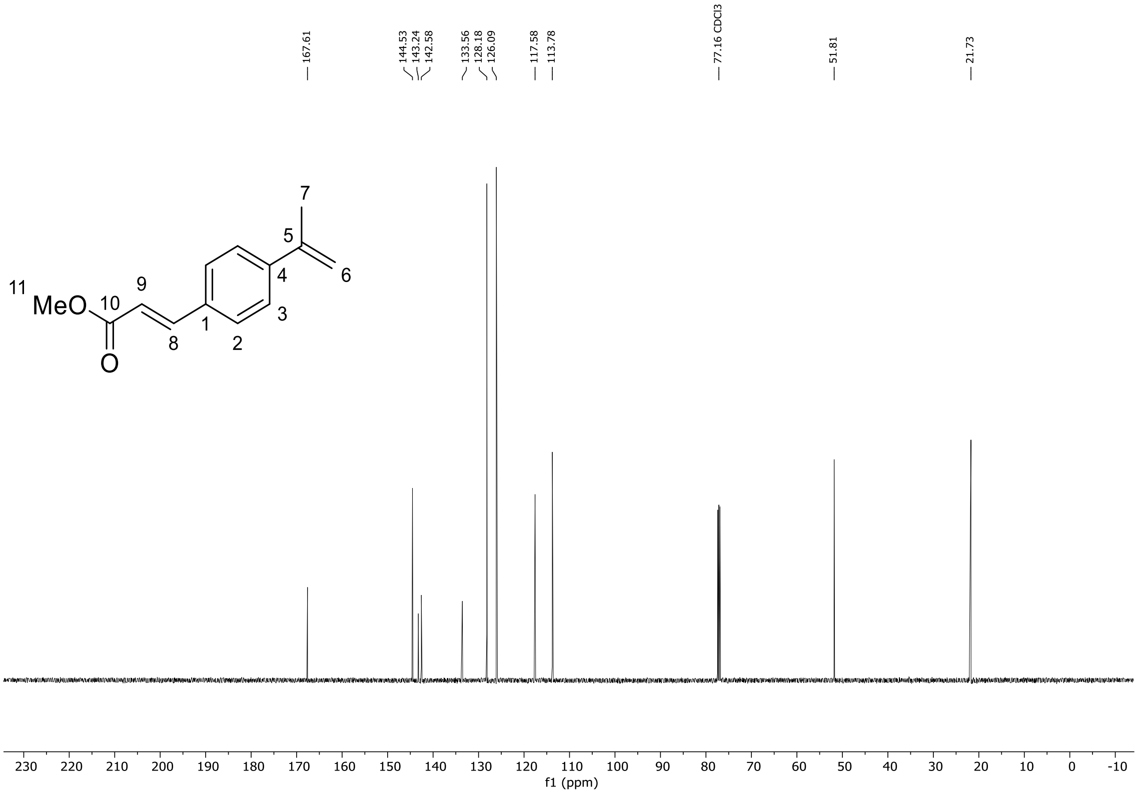

1-Bromo-2-(3-bromoprop-1-en-2-yl)benzene (1f)

^{1}H NMR (599 MHz, $CDCl_3$):

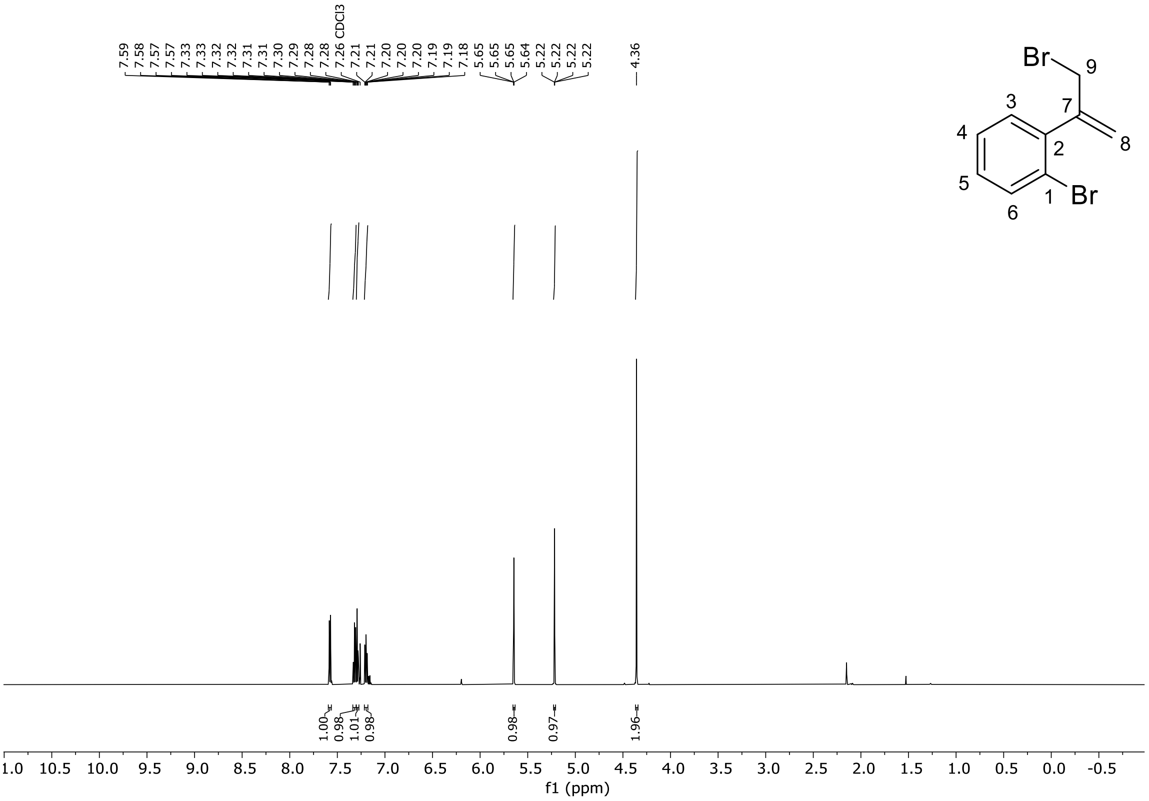

^{13}C NMR (151 MHz, $CDCl_3$):

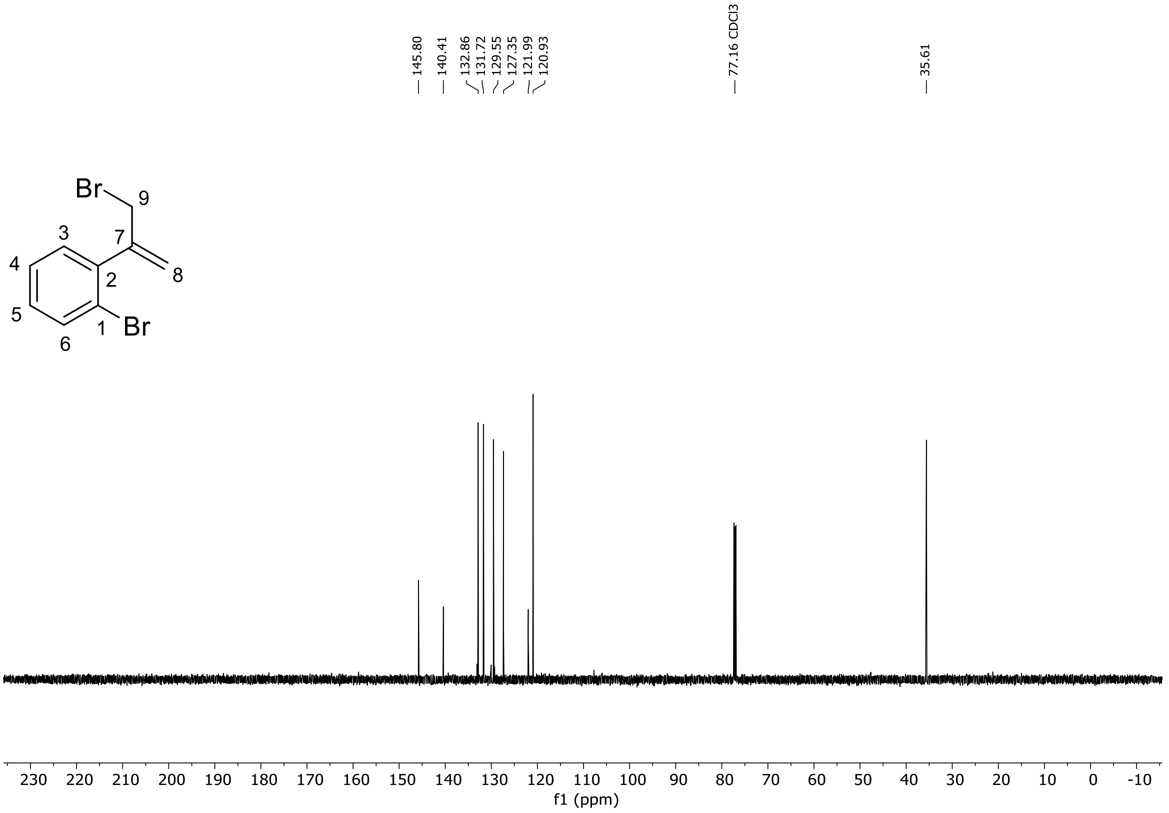

Methyl 4-(3-bromoprop-1-en-2-yl)benzoate (1i)

^{1}H NMR (599 MHz, $CDCl_3$):

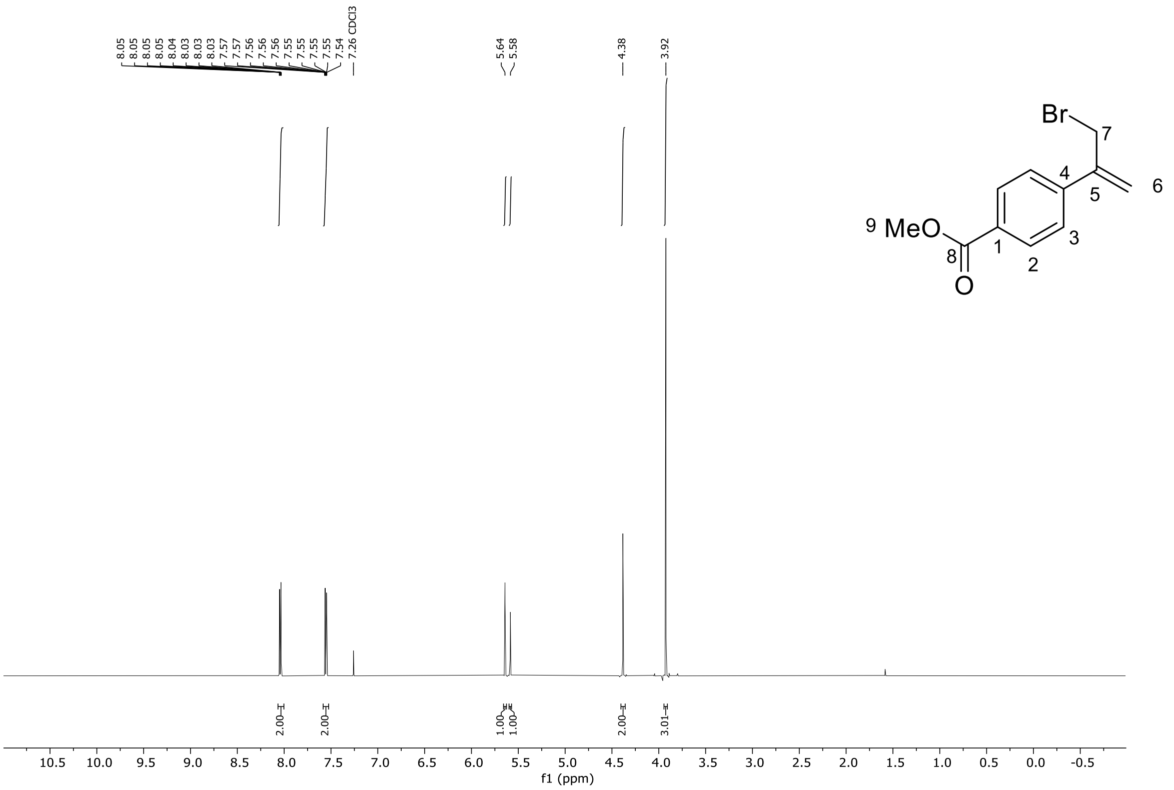

^{13}C NMR (151 MHz, $CDCl_3$):

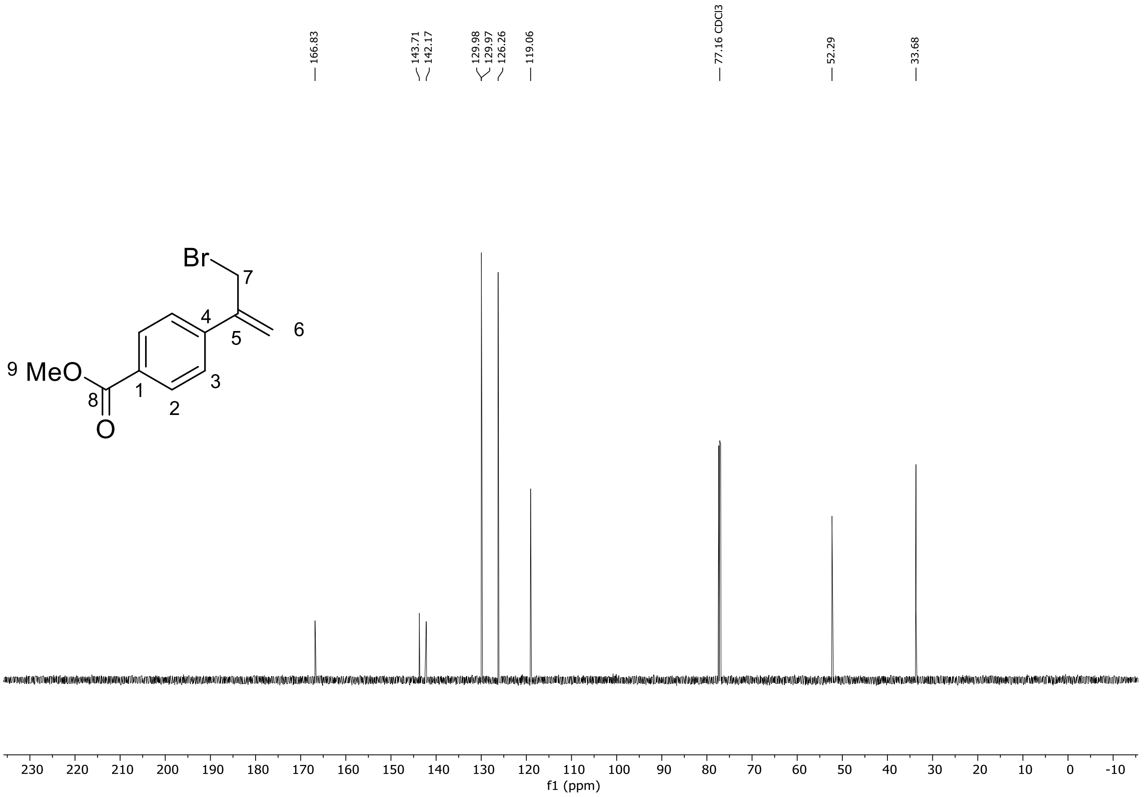

1-(3-Bromoprop-1-en-2-yl)-4-nitrobenzene (1j)

¹H NMR (500 MHz, $CDCl_3$):

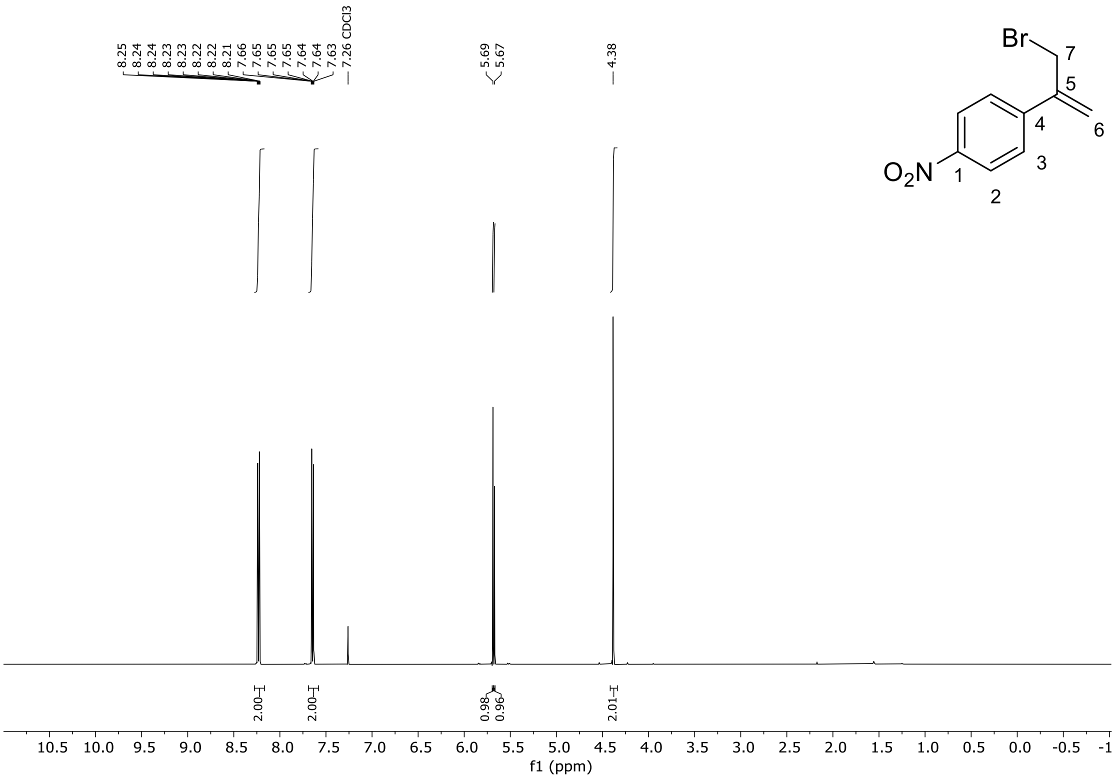

¹³C{¹H} NMR (126 MHz, $CDCl_3$):

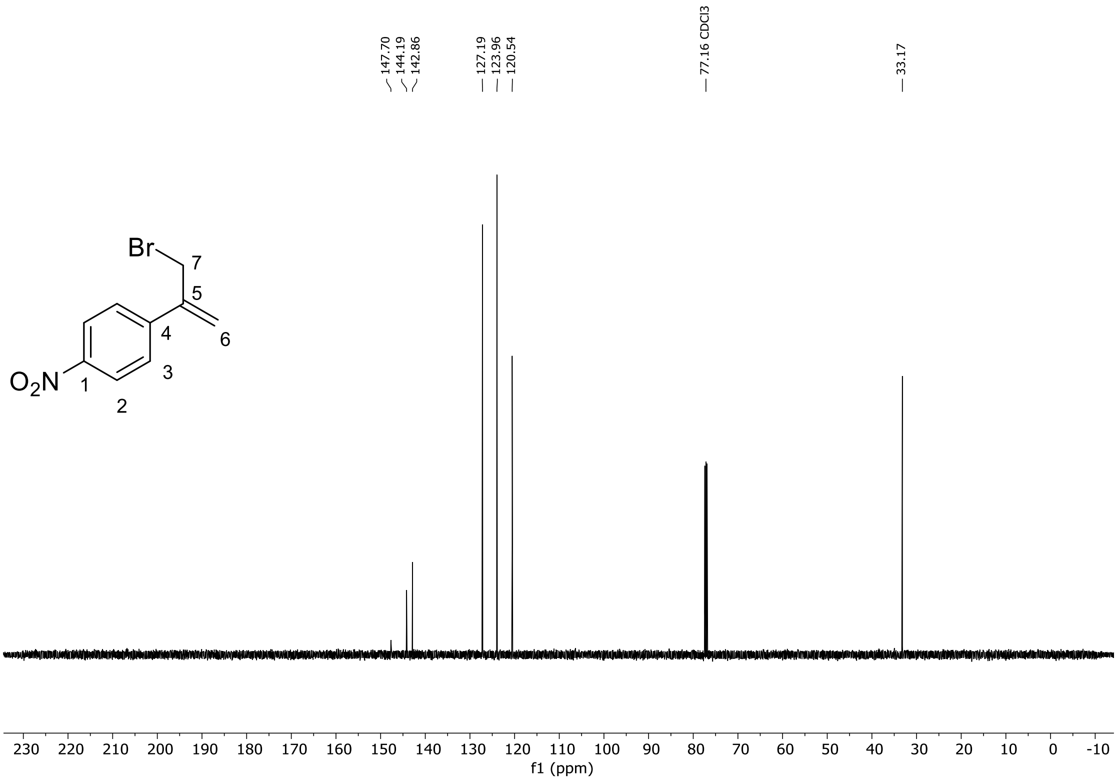

4-(3-Bromoprop-1-en-2-yl)phenyl 4-methylbenzenesulfonate (1k)

^{1}H NMR (500 MHz, $CDCl_3$):

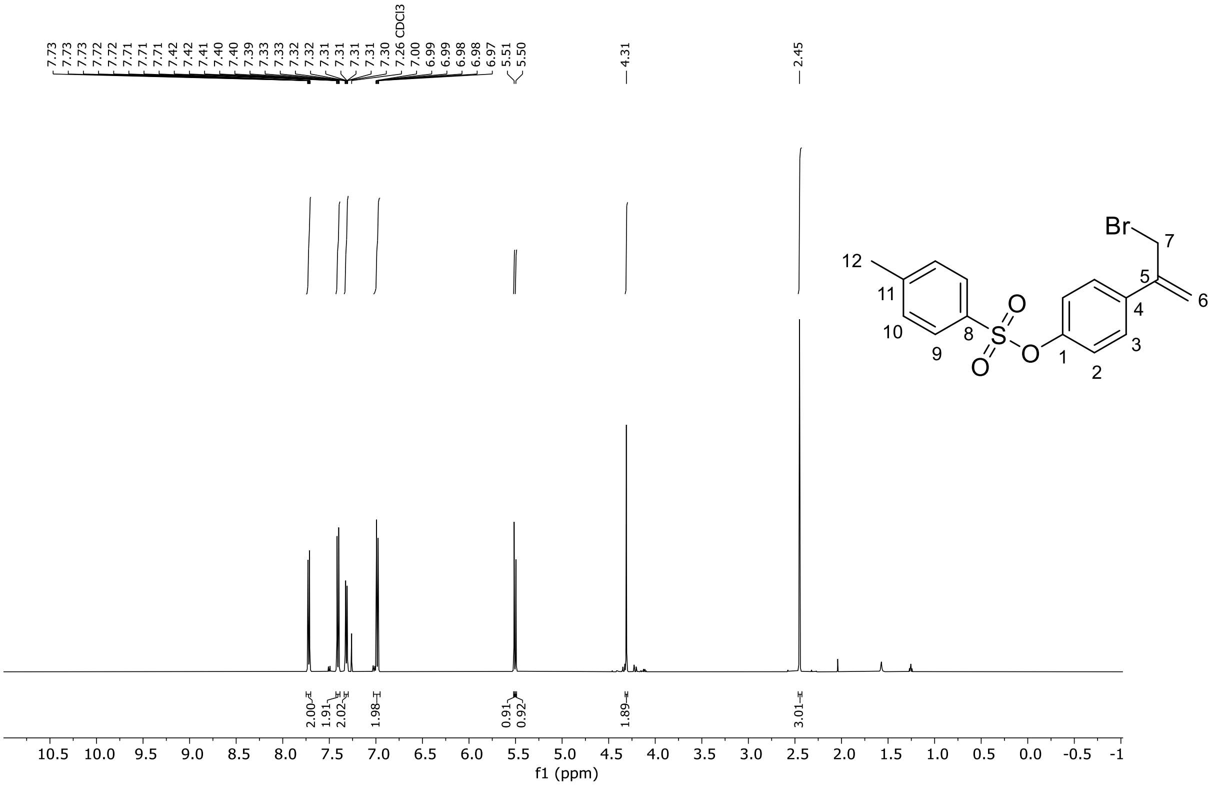

^{13}C{^{1}H} NMR (126 MHz, $CDCl_3$):

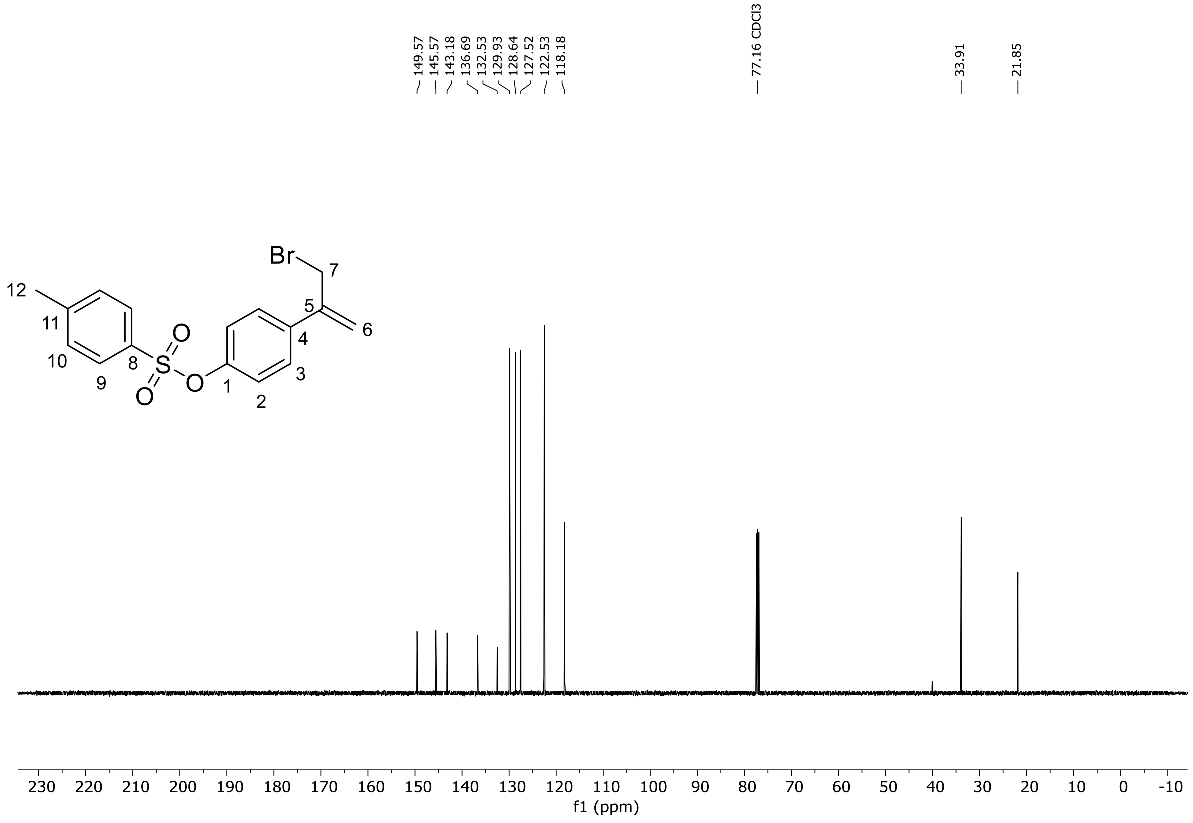

2-Bromo-4-(3-bromoprop-1-en-2-yl)-1-fluorobenzene (1l)

^{1}H NMR (500 MHz, $CDCl_3$):

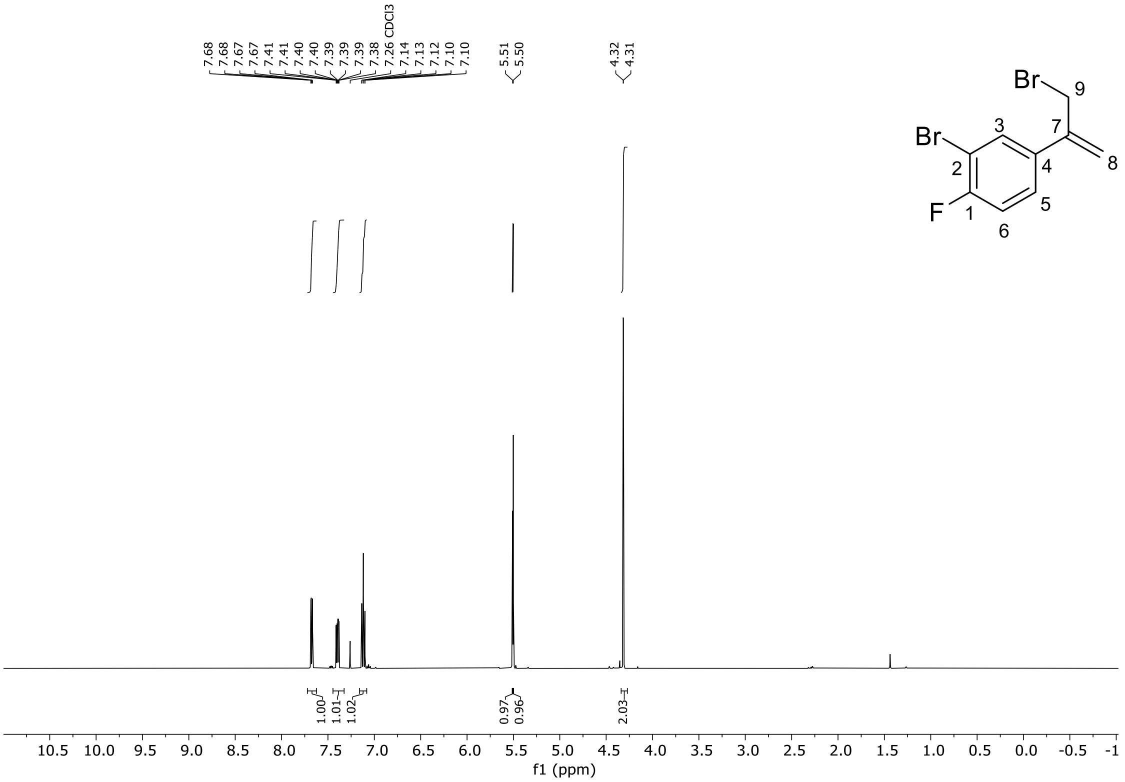

^{13}C{^{1}H} NMR (126 MHz, $CDCl_3$):

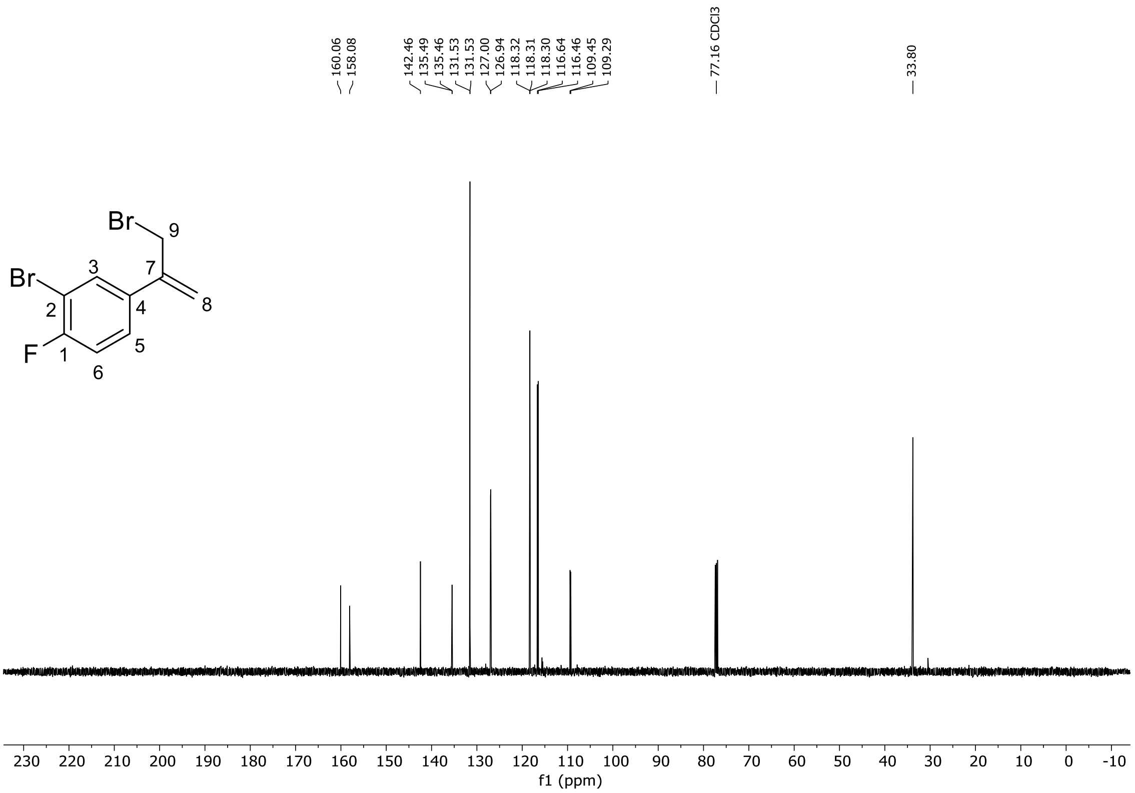

^{19}F NMR (376 MHz, $CDCl_3$):

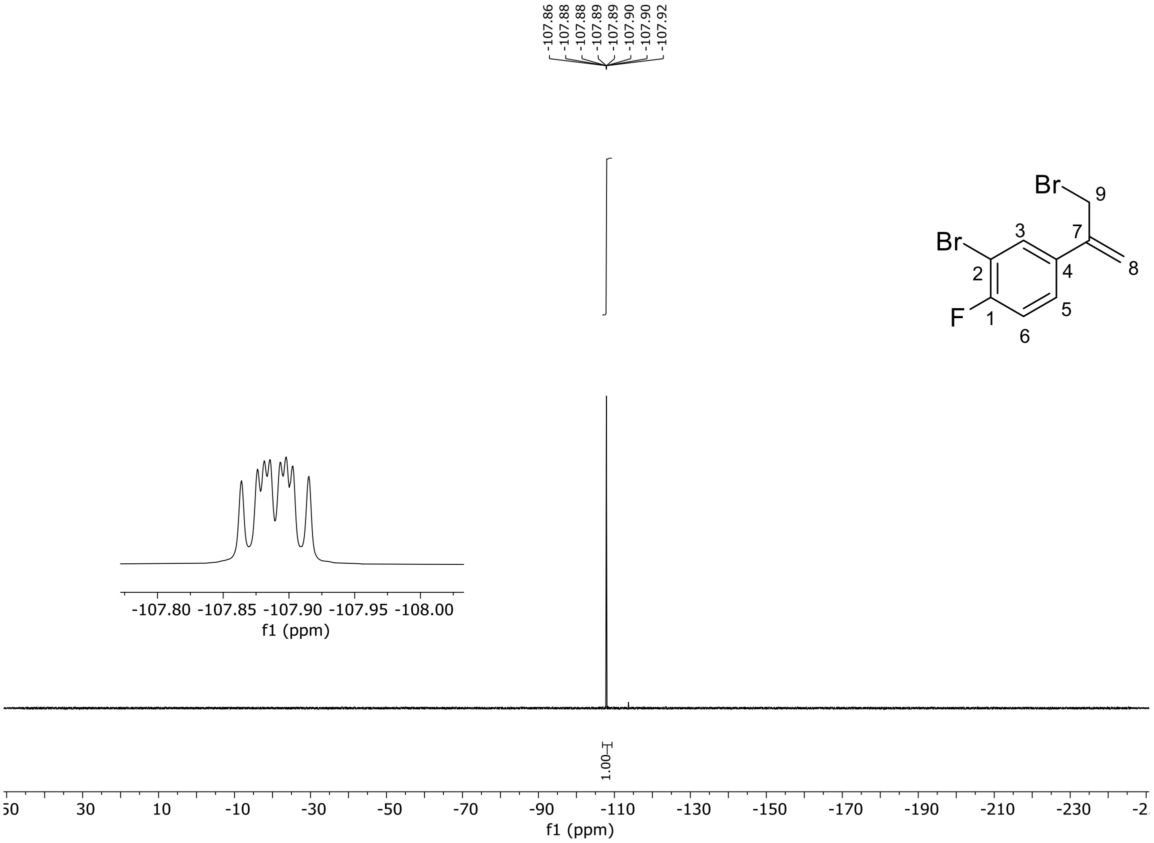

^{19}F{^{1}H} NMR (376 MHz, $CDCl_3$):

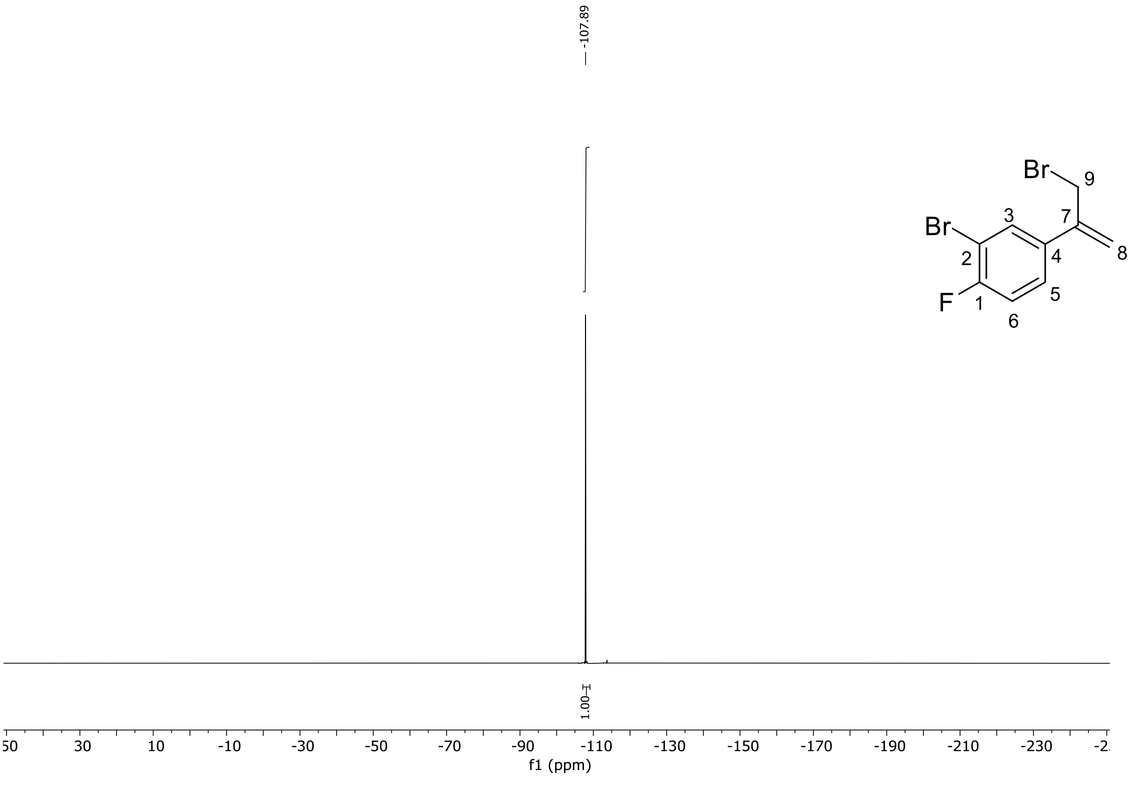

1-((4-(3-Bromoprop-1-en-2-yl)phenyl)sulfonyl)piperidine (1m)

^{1}H NMR (500 MHz, $CDCl_3$):

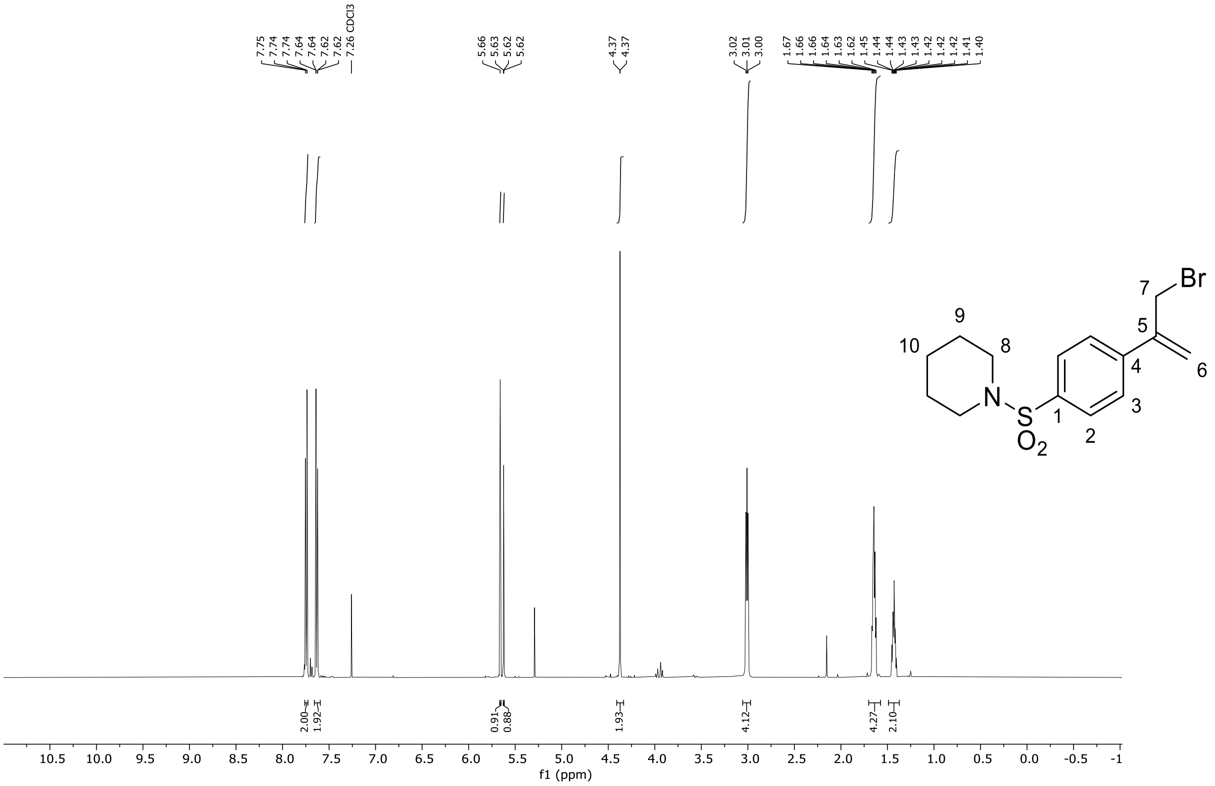

^{13}C{^{1}H} NMR (126 MHz, $CDCl_3$):

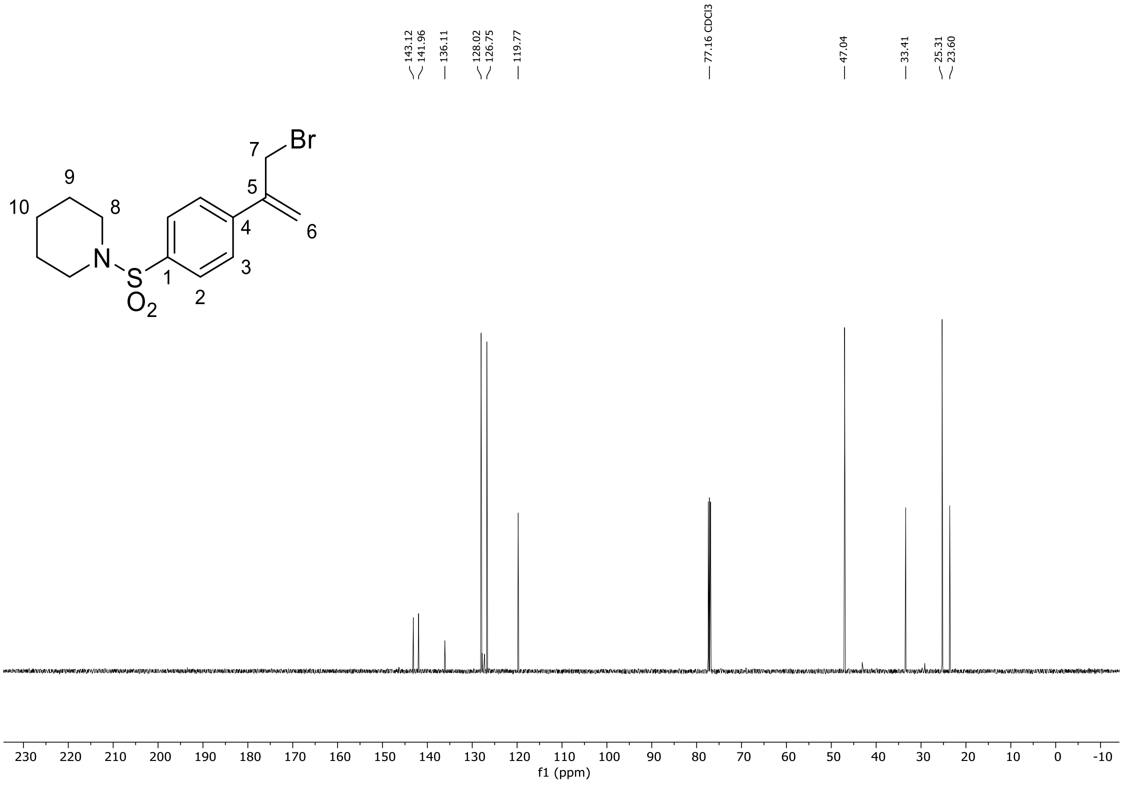

2-(4-(3-Bromoprop-1-en-2-yl)phenyl)isoindoline-1,3-dione (1n)

^{1}H NMR (599 MHz, $CDCl_3$):

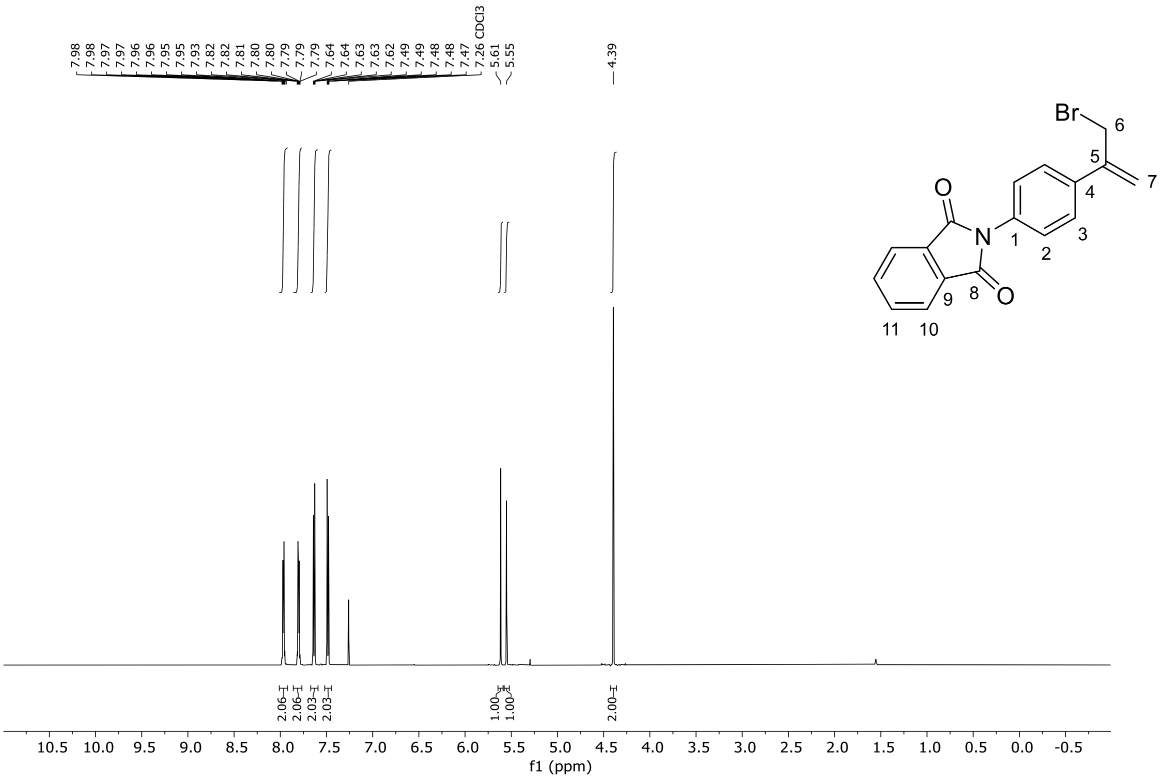

$^{13}C\{^1H\}$ NMR (151 MHz, $CDCl_3$):

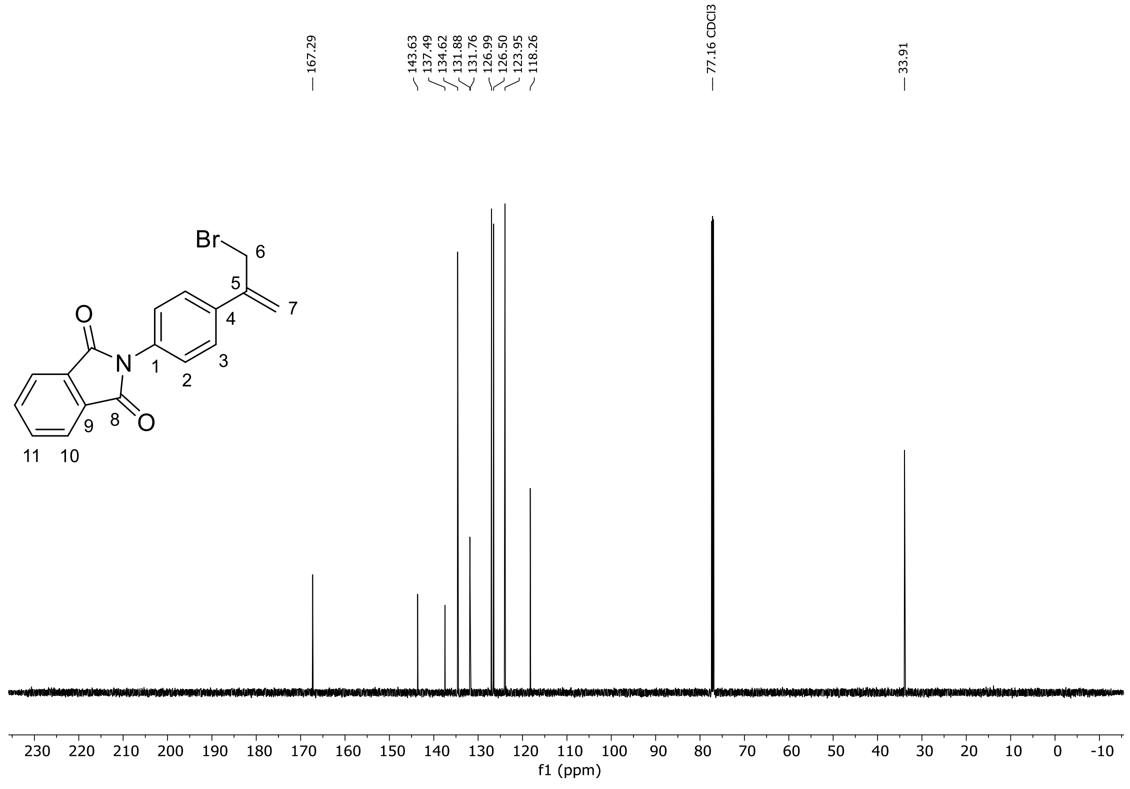

1-(3-Bromoprop-1-en-2-yl)-4-(3,3,3-trifluoroprop-1-en-2-yl)benzene (3)

^{1}H NMR (500 MHz, $CDCl_3$):

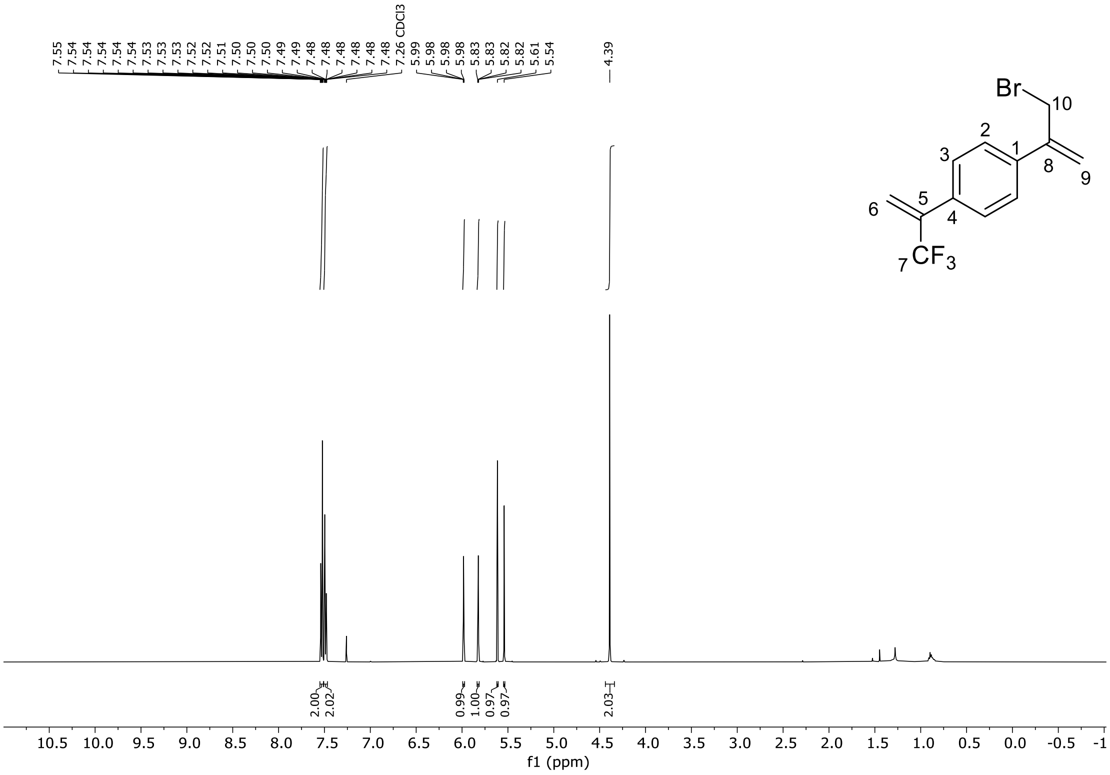

^{13}C{^{1}H} NMR (126 MHz, $CDCl_3$):

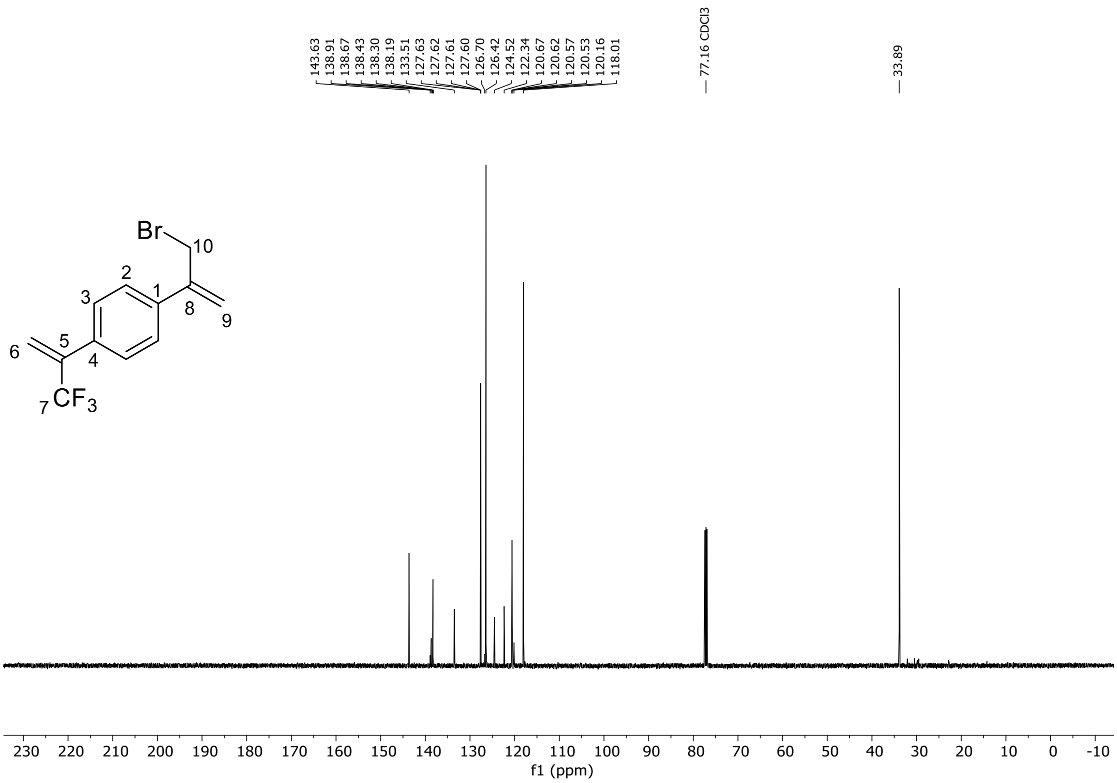

^{19}F NMR (376 MHz, $CDCl_3$):

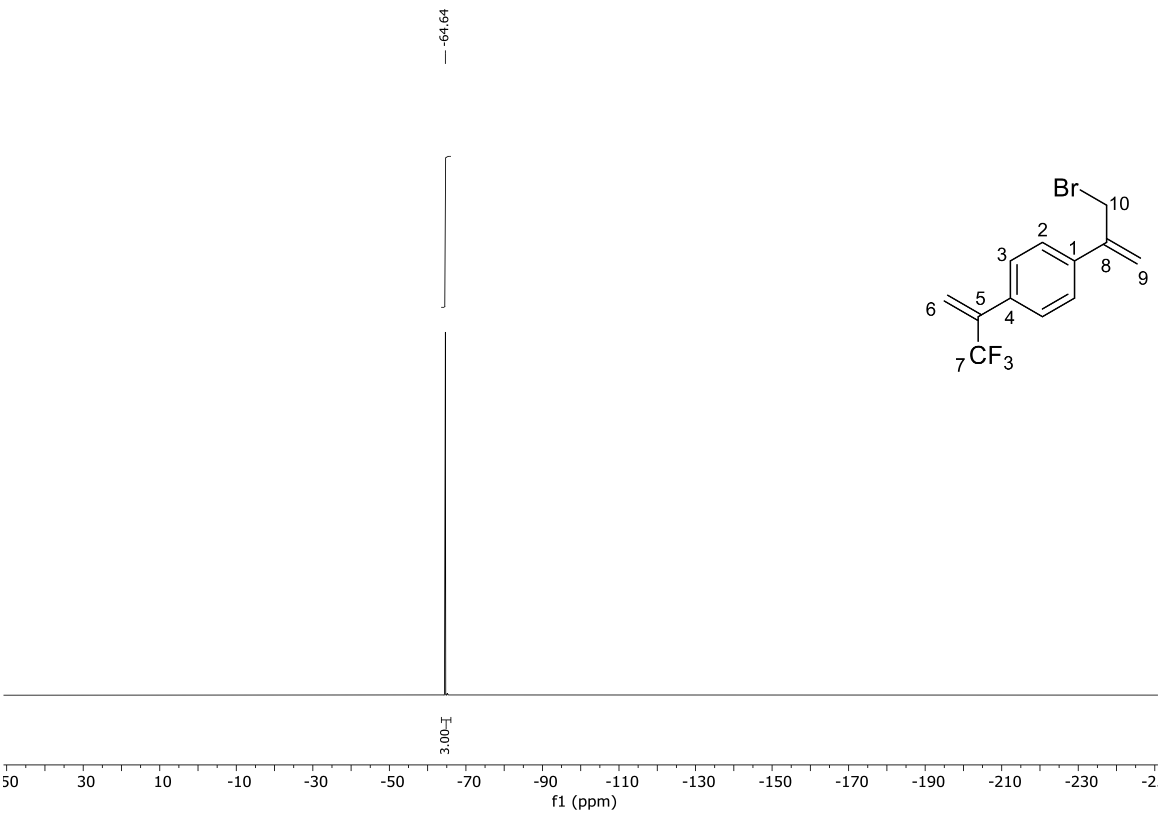

^{19}F{^{1}H} NMR (376 MHz, $CDCl_3$):

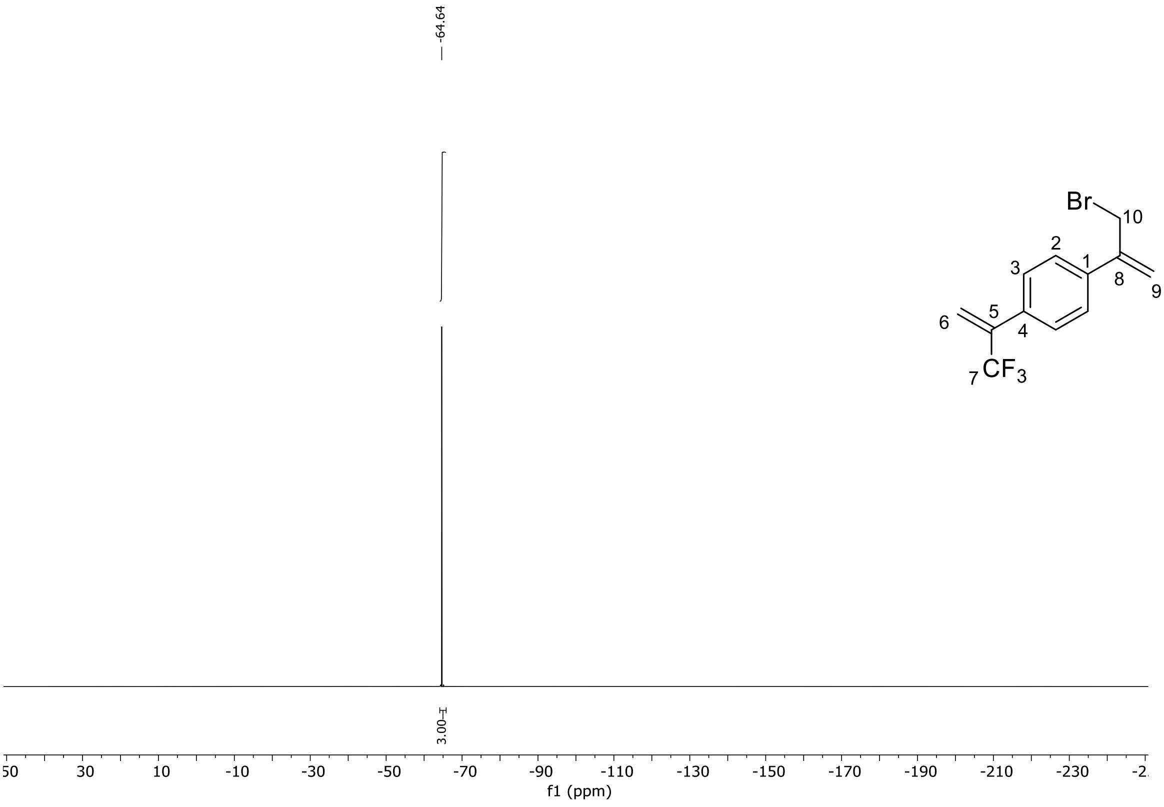

Methyl (*E*)-3-(4-(3-bromoprop-1-en-2-yl)phenyl)acrylate (6)

^{1}H NMR (500 MHz, $CDCl_3$):

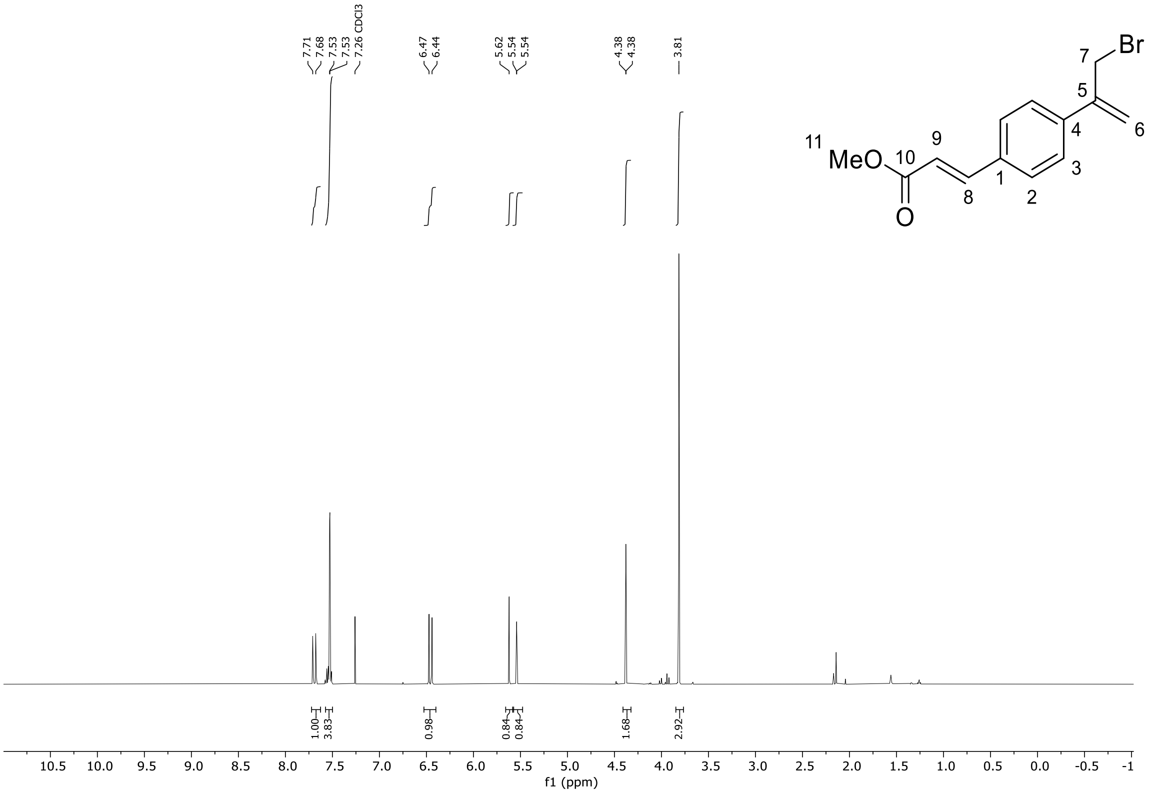

^{13}C{^{1}H} NMR (126 MHz, $CDCl_3$):

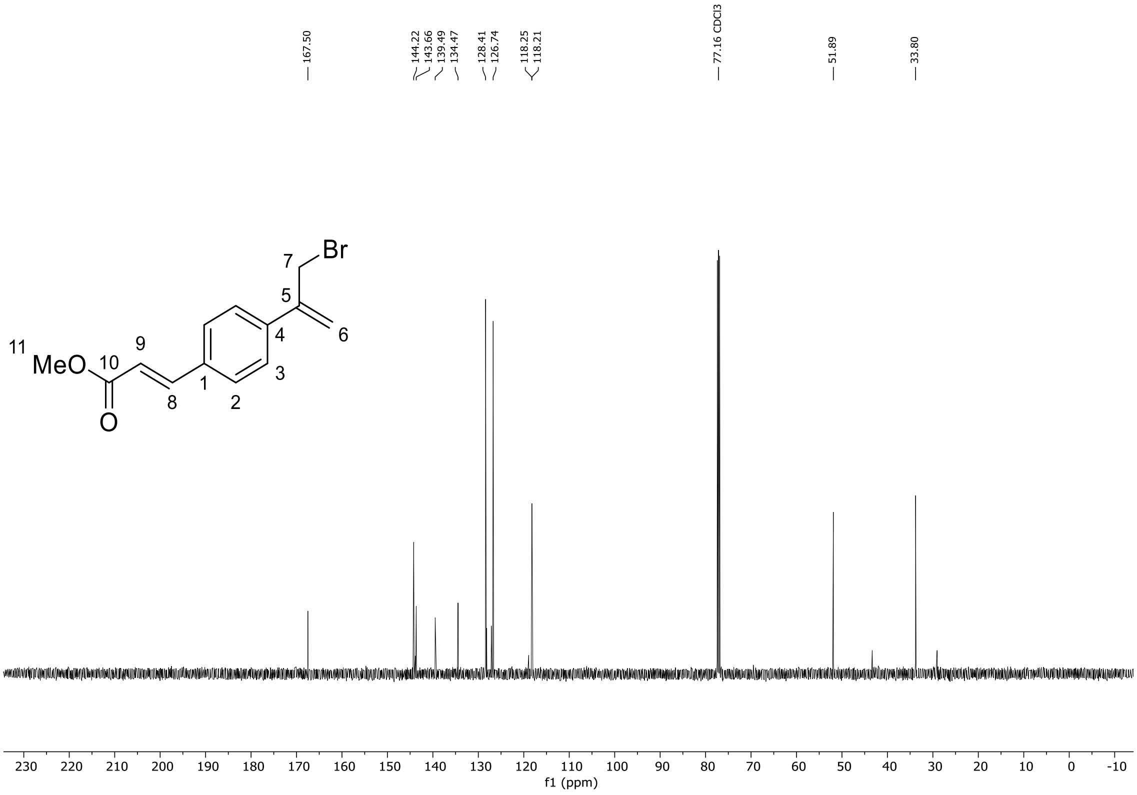

1,4-*Bis*(3-bromoprop-1-en-2-yl)benzene (9)

^{1}H NMR (500 MHz, $CDCl_3$):

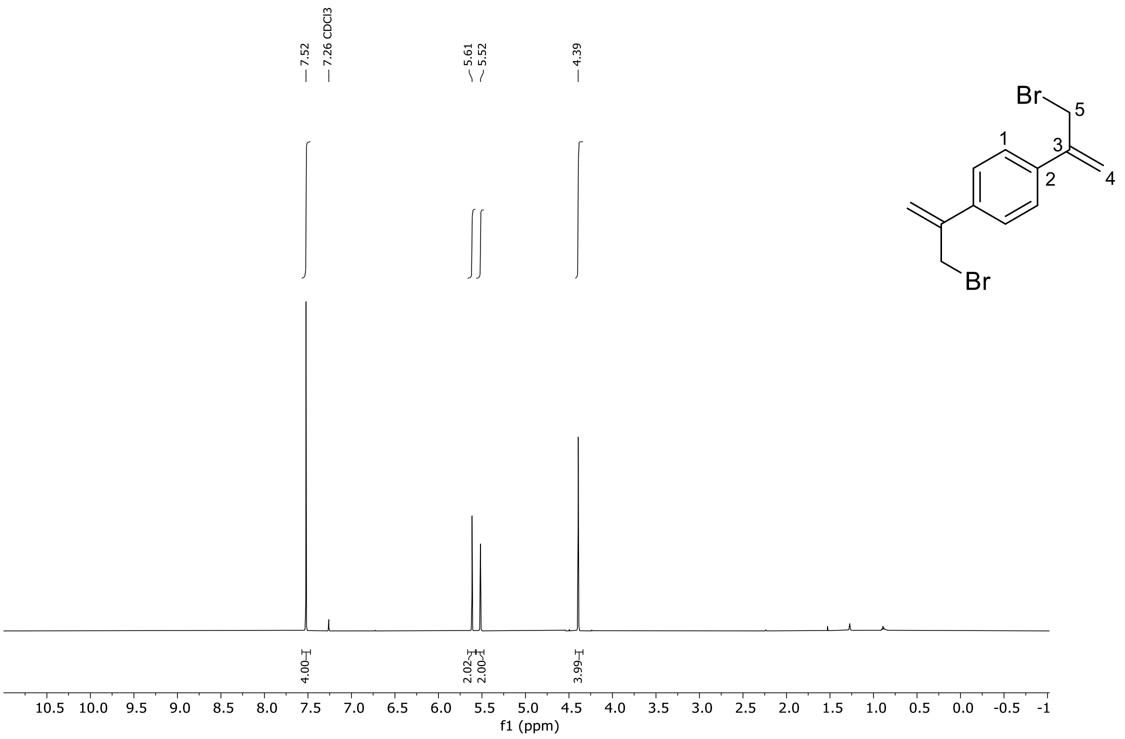

^{13}C{^{1}H} NMR (126 MHz, $CDCl_3$):

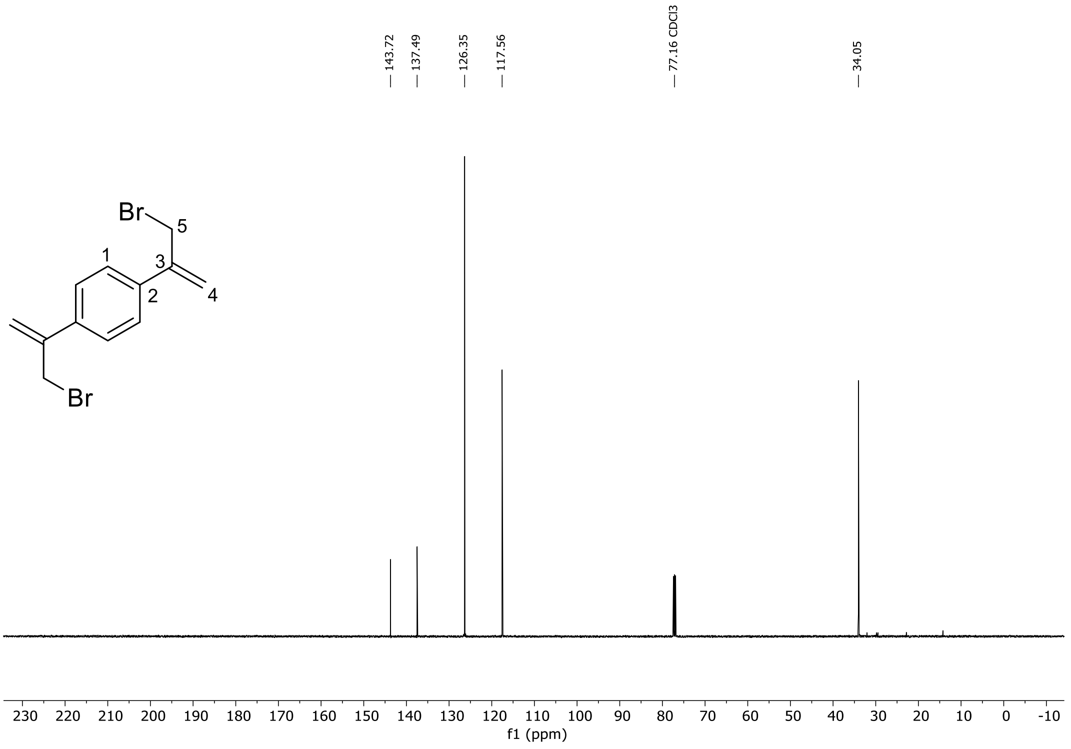

1,3-*Bis*(3-bromoprop-1-en-2-yl)benzene (11)

^{1}H NMR (500 MHz, $CDCl_3$):

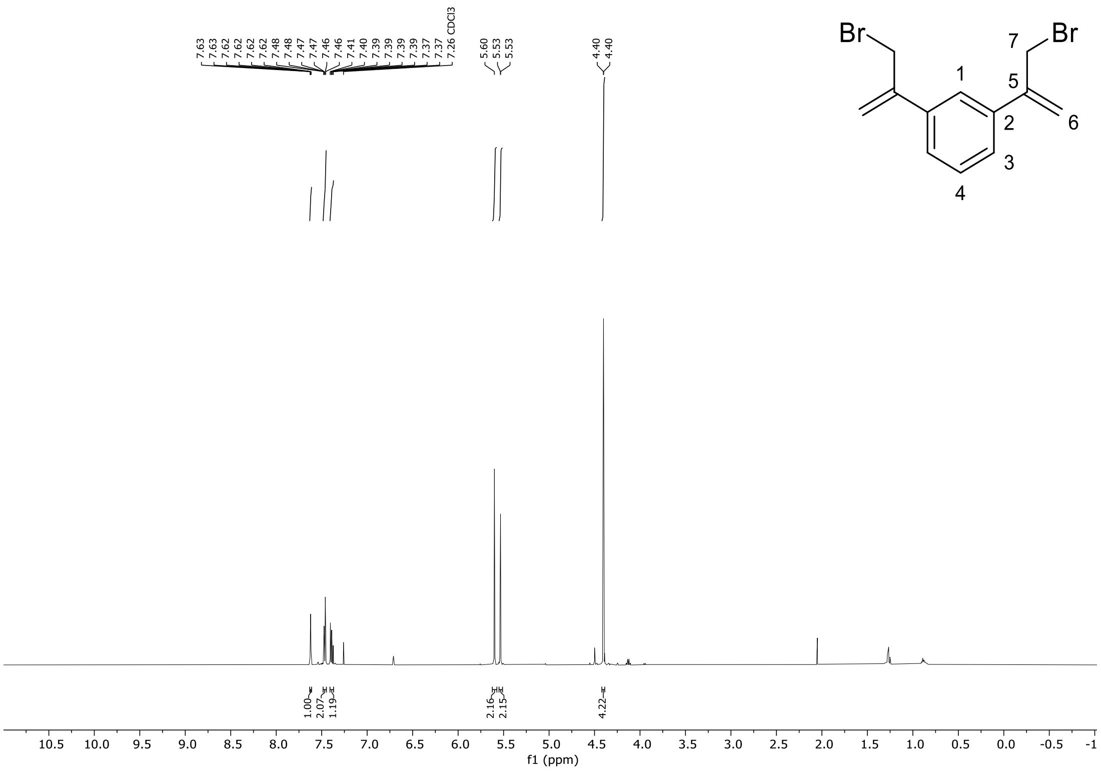

$^{13}C\{^1H\}$ NMR (126 MHz, $CDCl_3$):

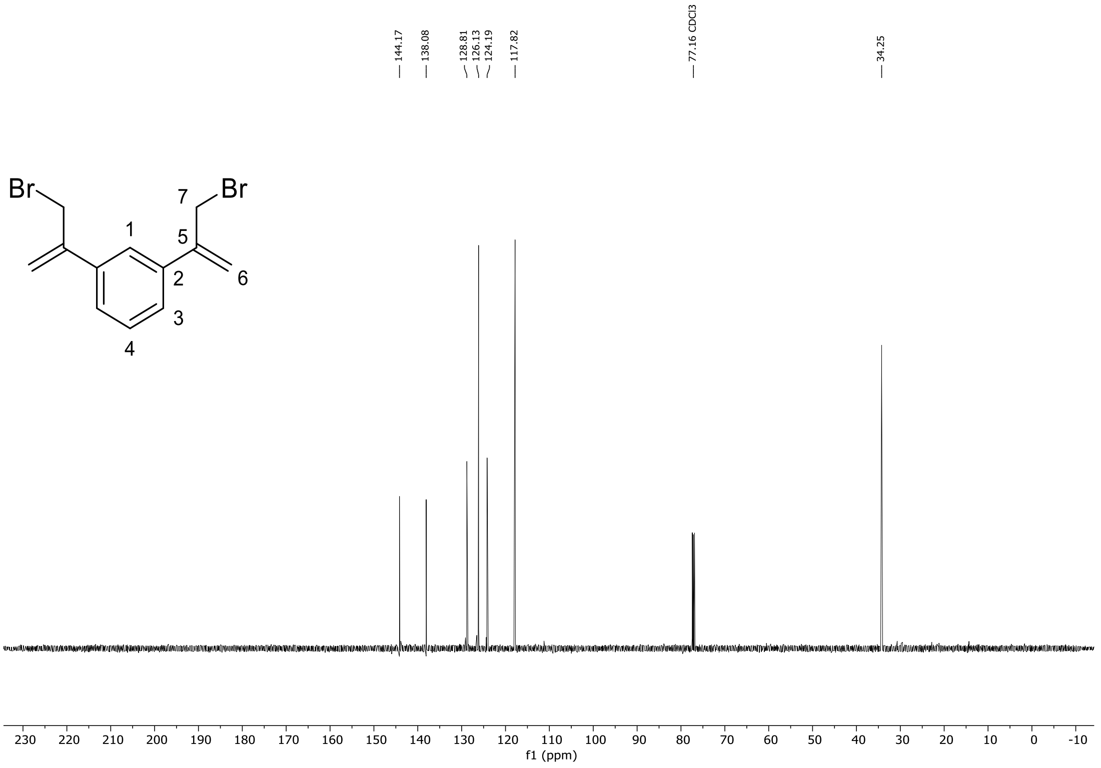

Ethyl (*E*)-4-bromo-3-phenylbut-2-enoate (13)

^{1}H NMR (500 MHz, $CDCl_3$):

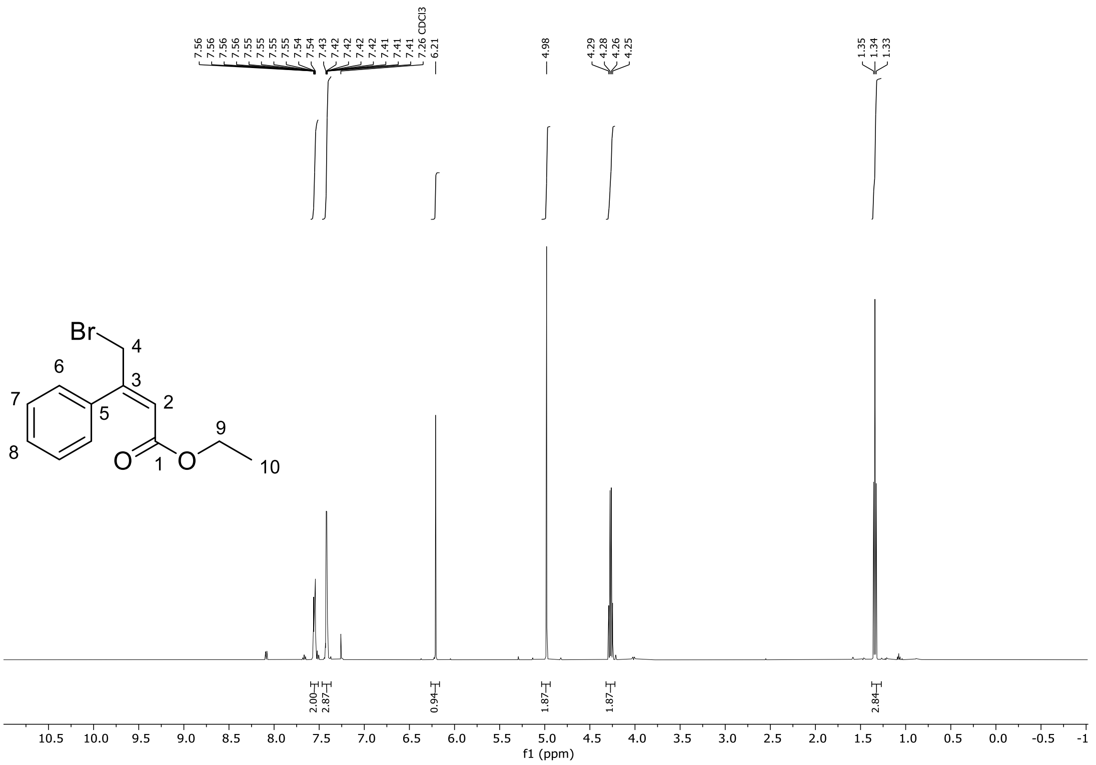

$^{13}C\{^1H\}$ NMR (126 MHz, $CDCl_3$):

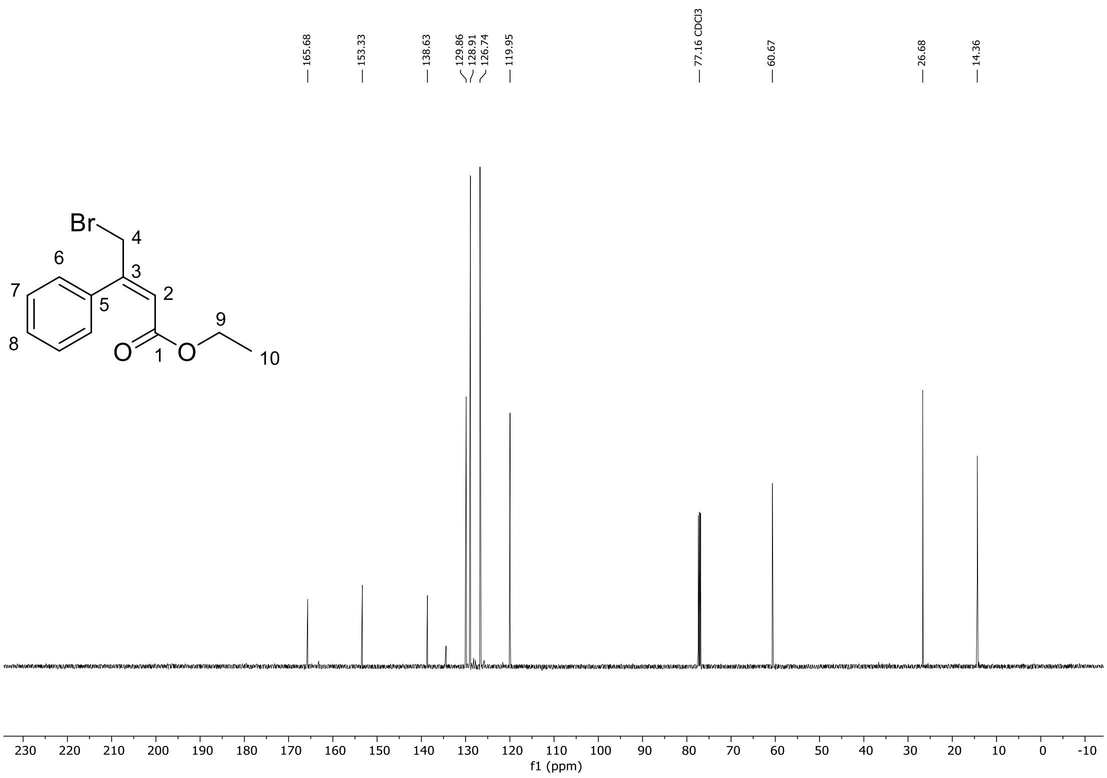

1-Bromo-4-(3-bromo-2,2-difluoropropyl)benzene (2a)

^{1}H NMR (500 MHz, $CDCl_3$):

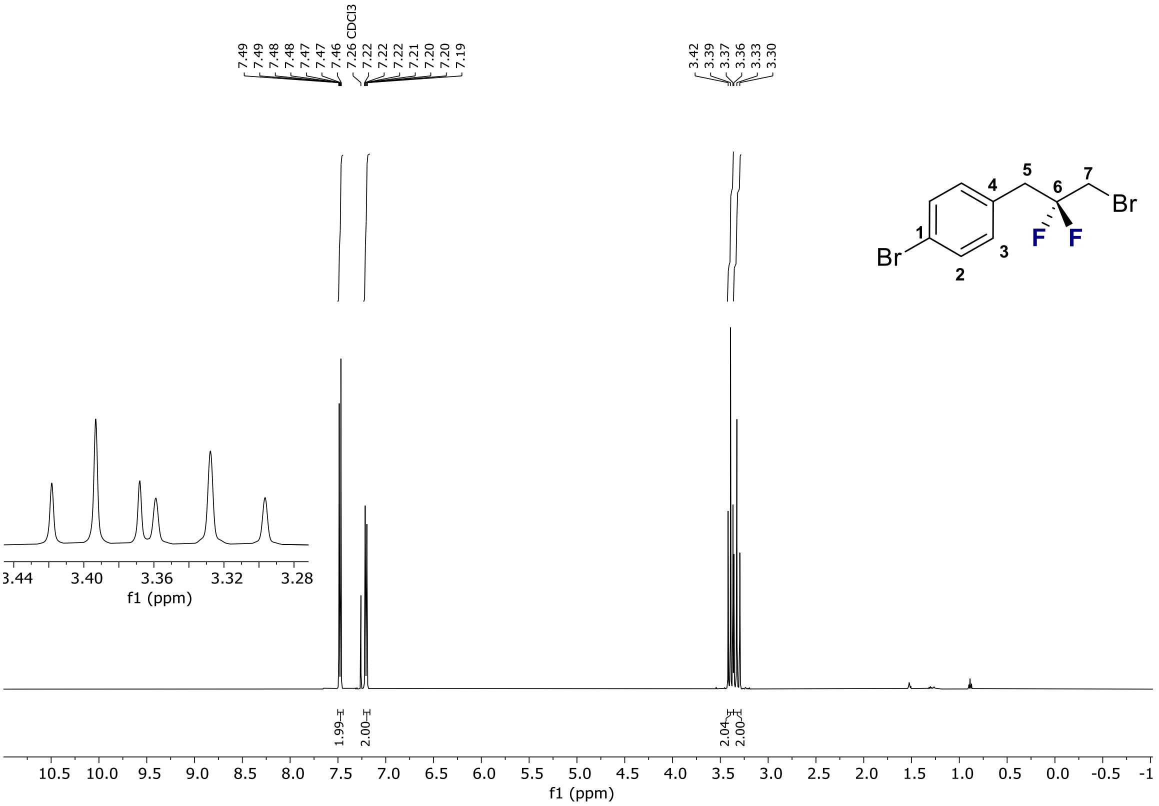

$^{13}C\{^1H\}$ NMR (126 MHz, $CDCl_3$):

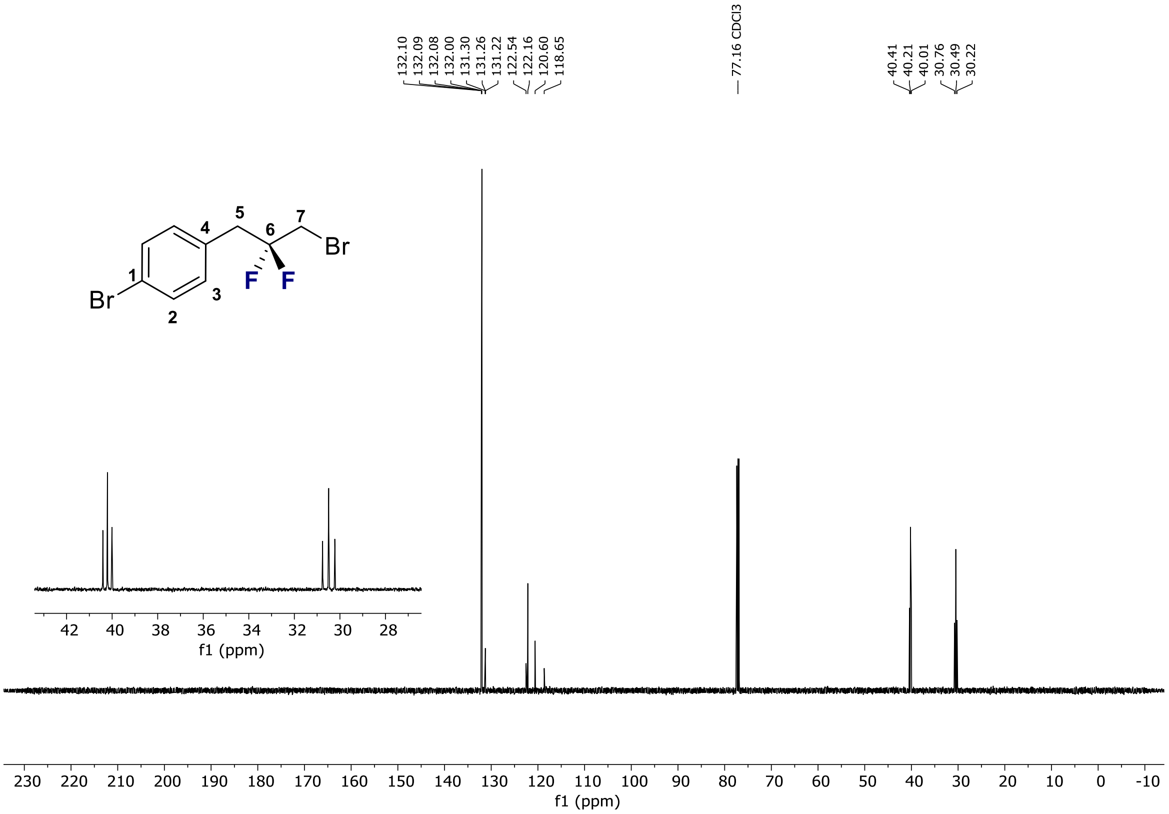

^{19}F NMR (376 MHz, $CDCl_3$):

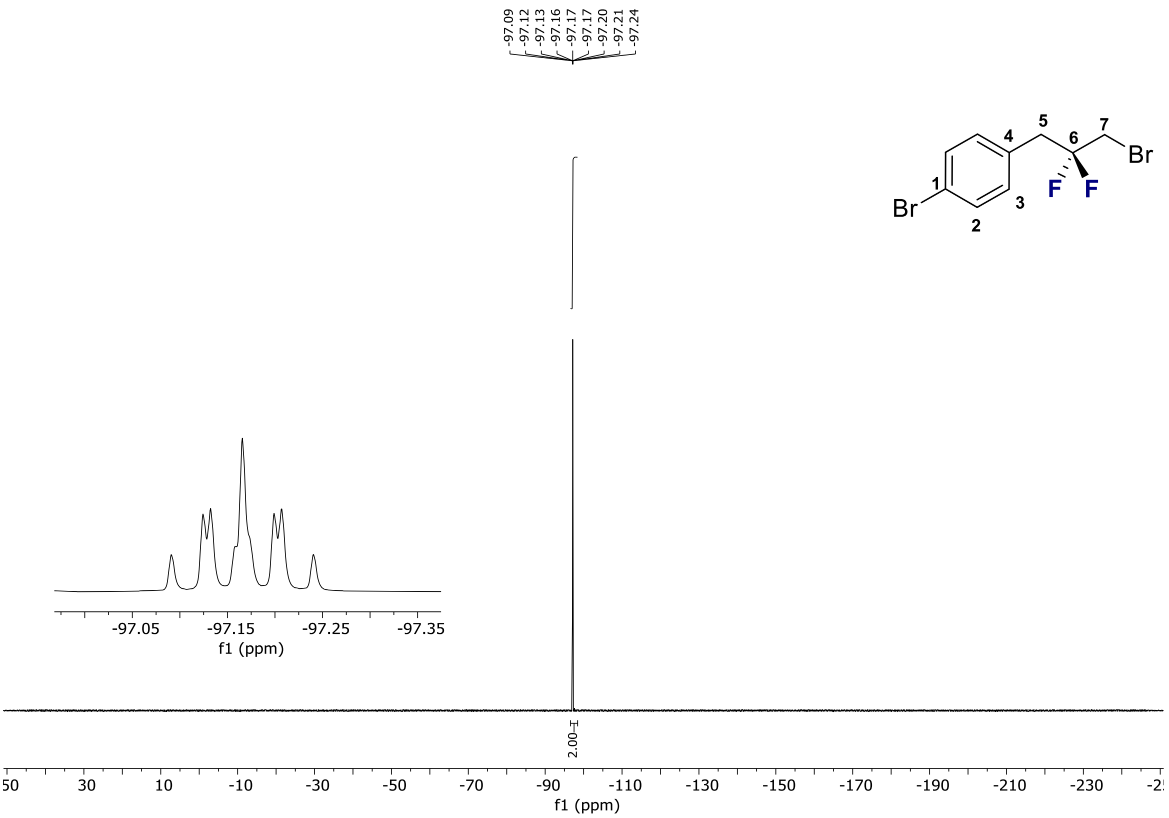

^{19}F{^{1}H} NMR (376 MHz, $CDCl_3$):

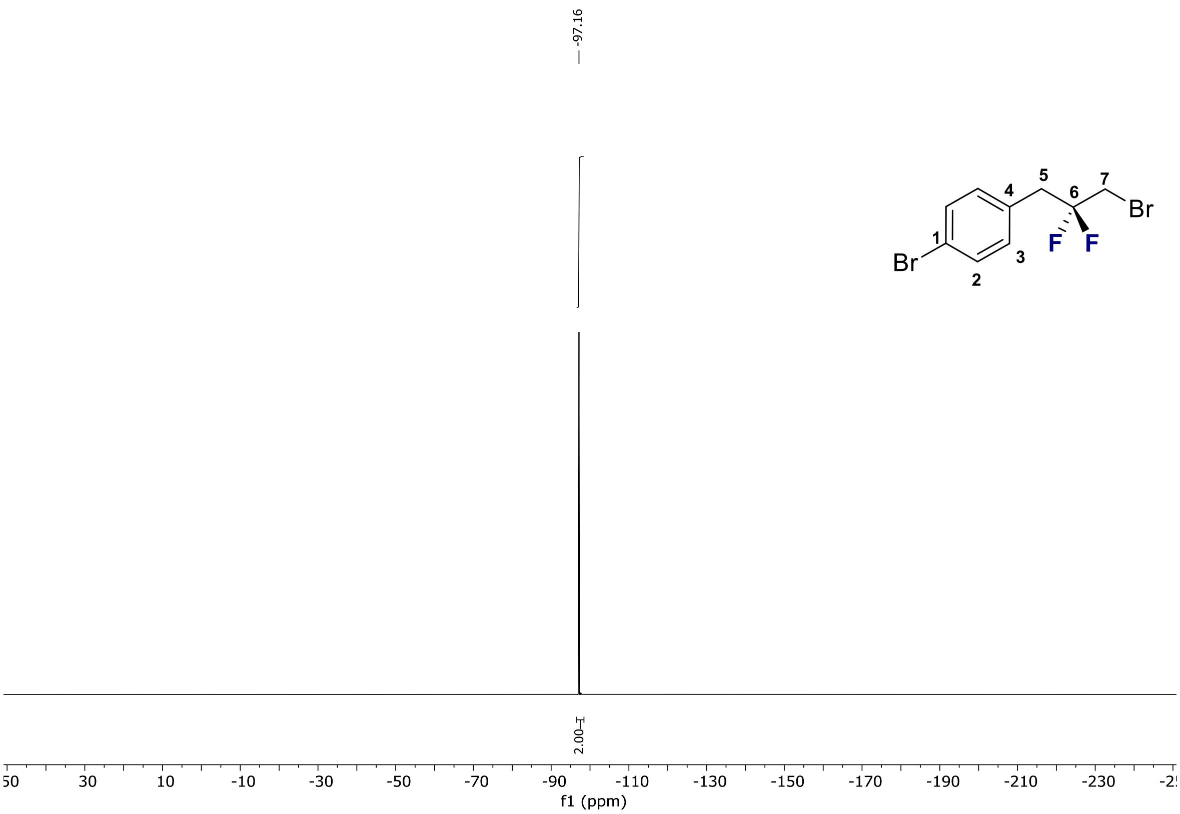

1-(3-Bromo-2,2-difluoropropyl)benzene (2b)

^{1}H NMR (500 MHz, $CDCl_3$):

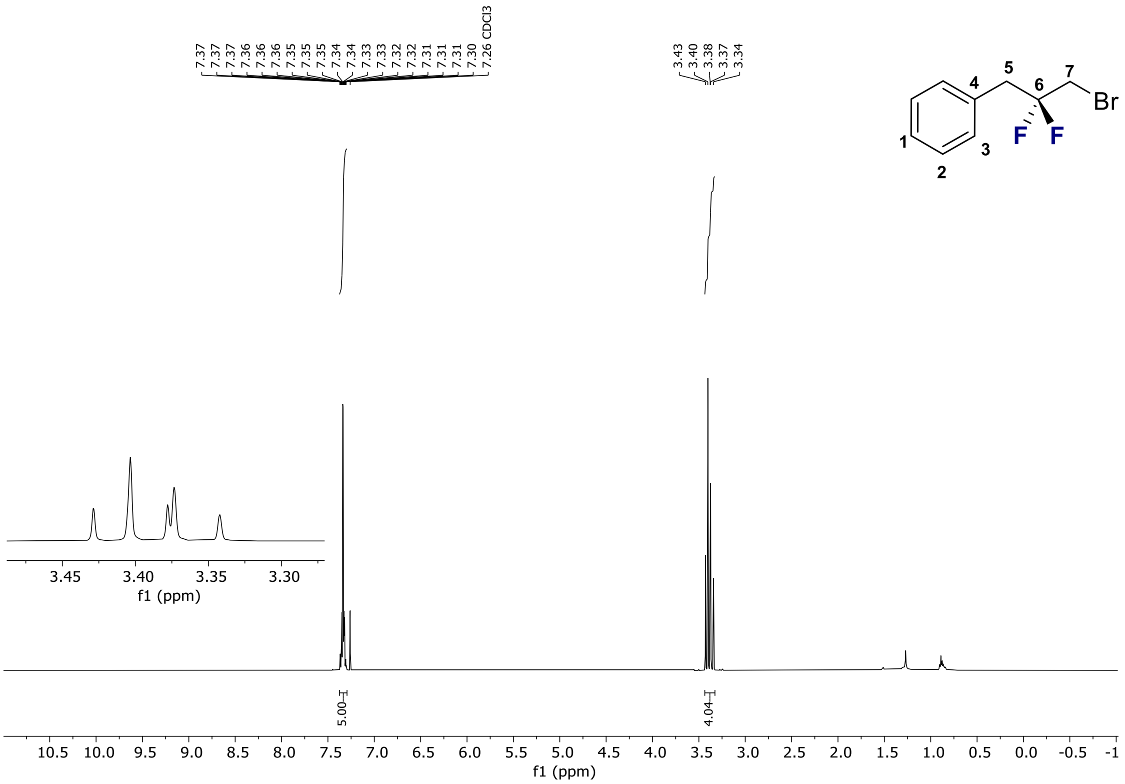

^{13}C{^{1}H} NMR (126 MHz, $CDCl_3$):

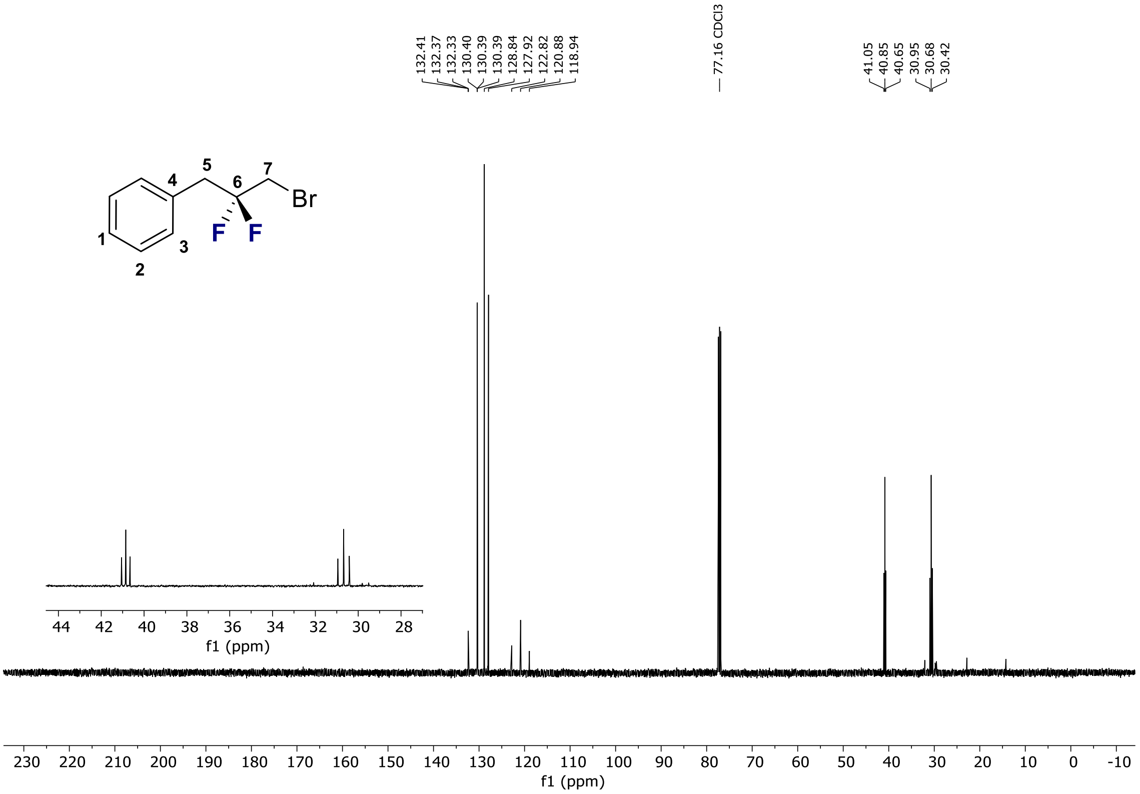

^{19}F NMR (376 MHz, $CDCl_3$):

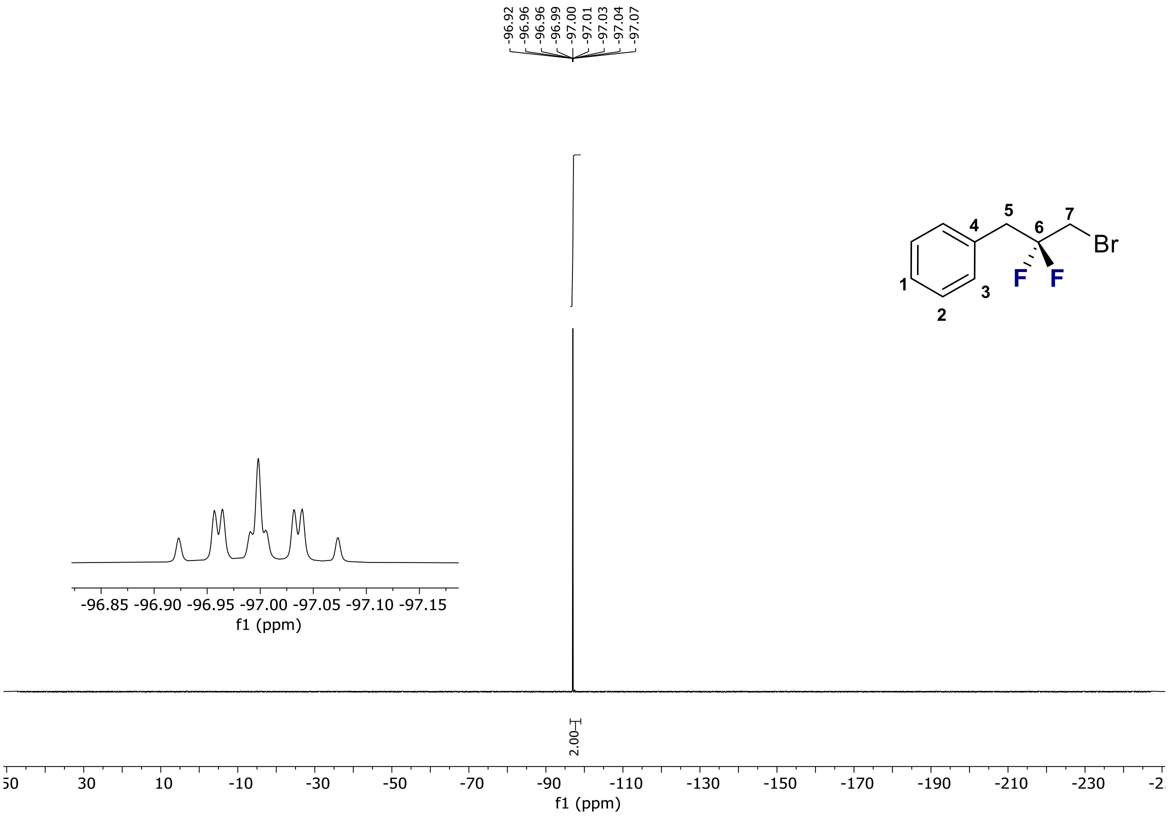

^{19}F{^{1}H} NMR (376 MHz, $CDCl_3$):

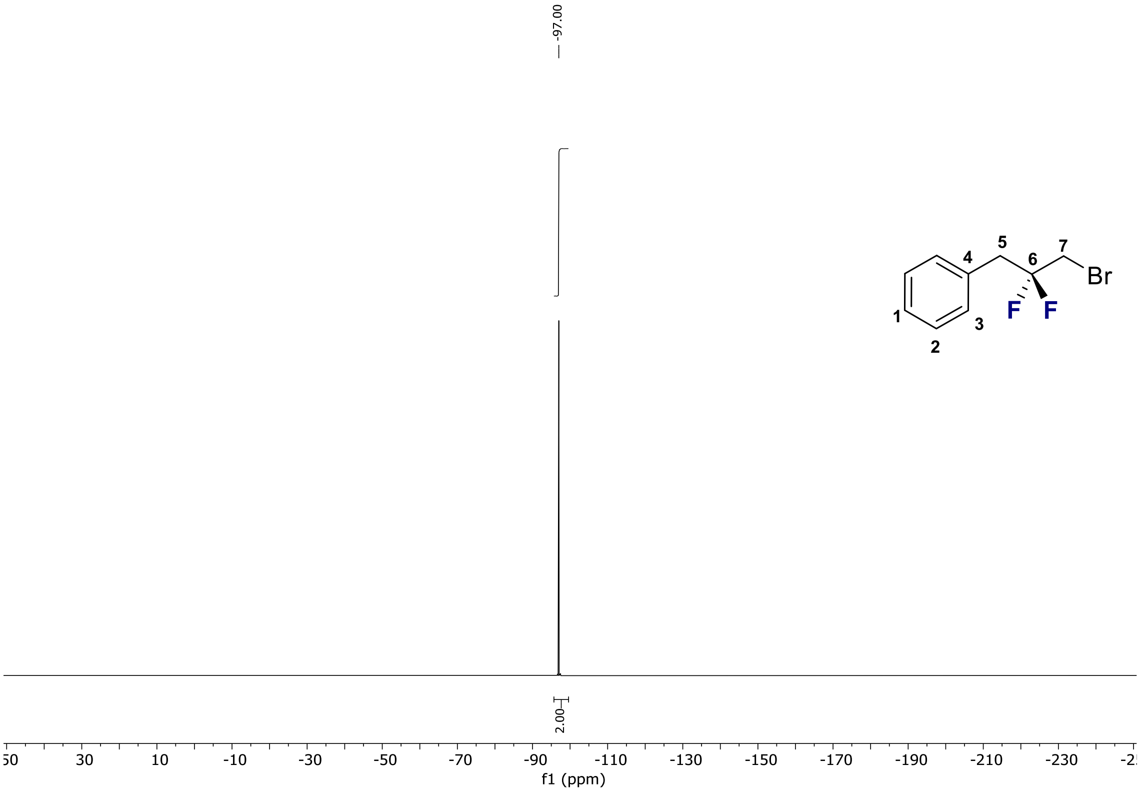

1-(3-Bromo-2,2-difluoropropyl)-4-fluorobenzene (2c)

^{1}H NMR (500 MHz, $CDCl_3$):

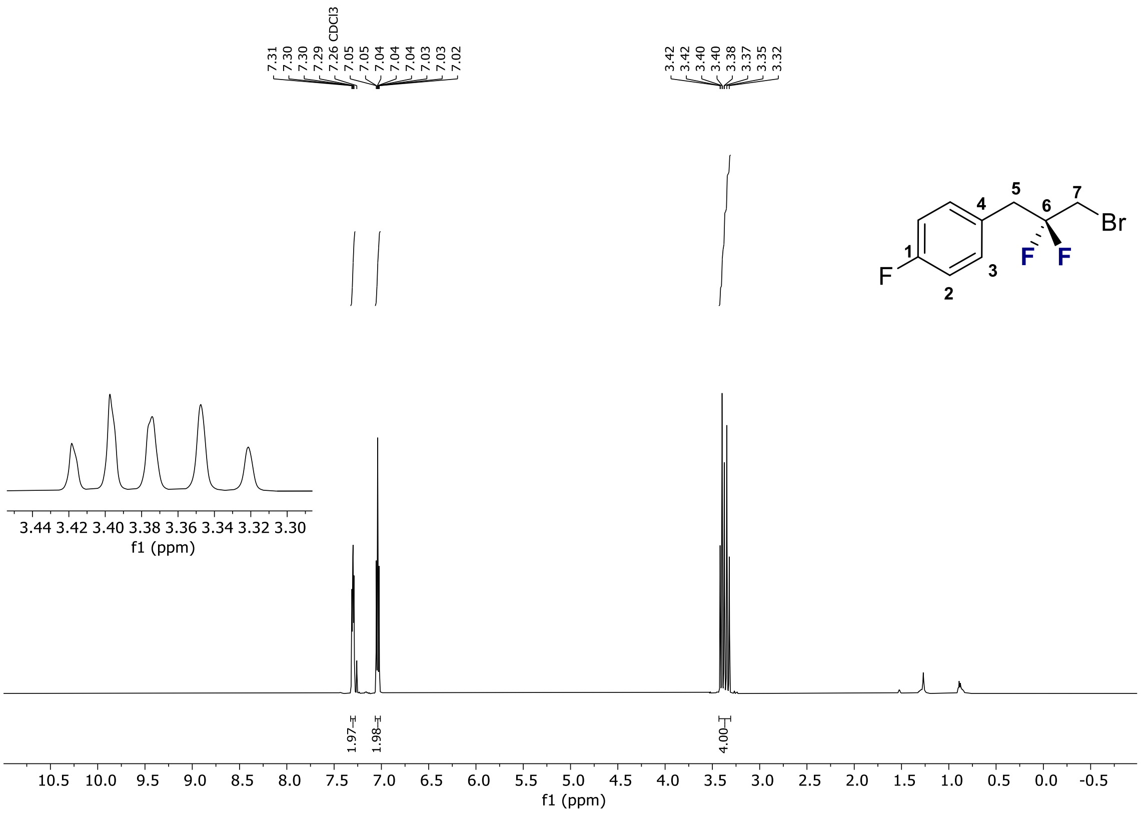

^{13}C{^{1}H} NMR (126 MHz, $CDCl_3$):

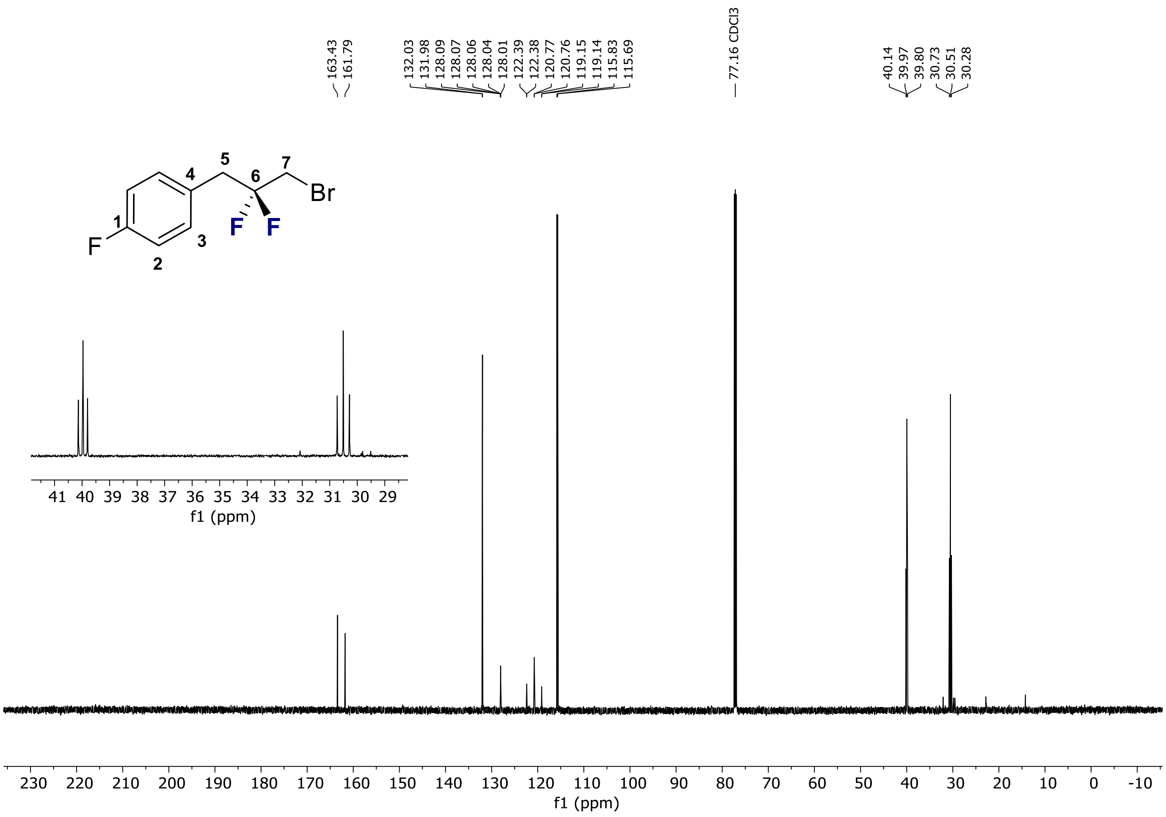

^{19}F NMR (376 MHz, $CDCl_3$):

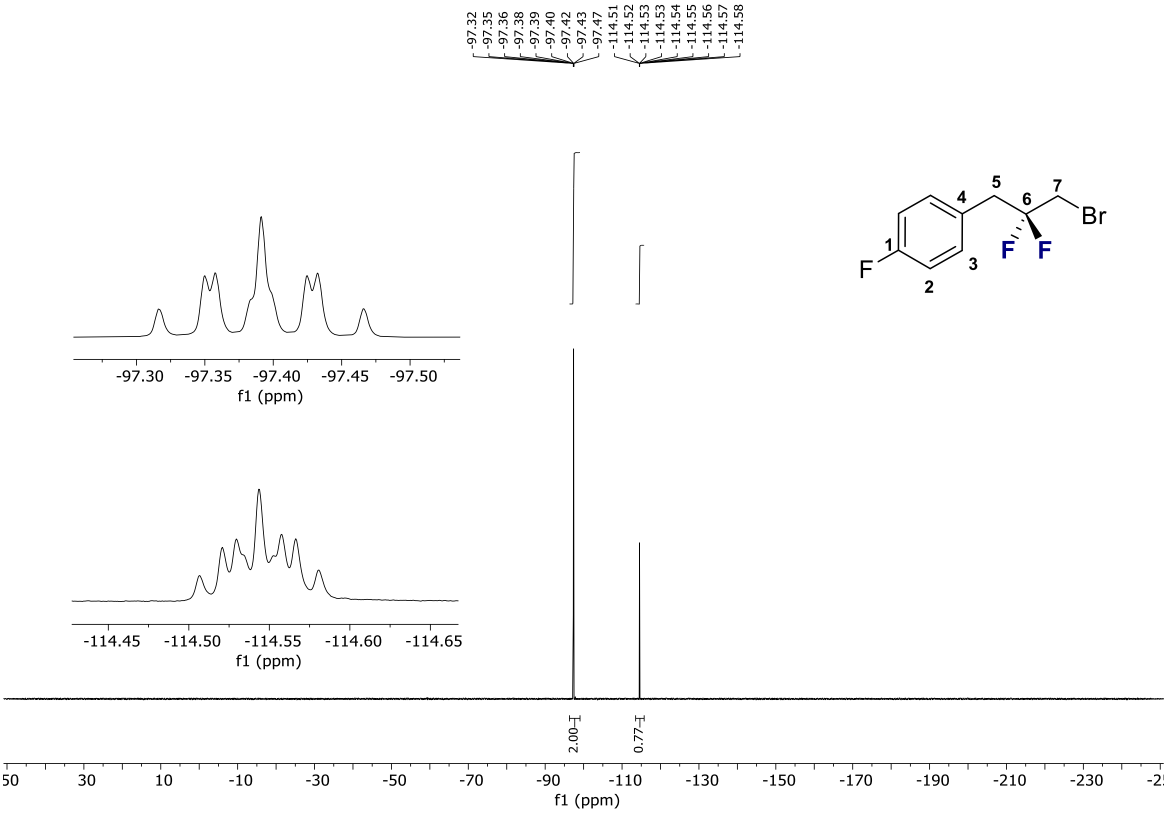

^{19}F{^{1}H} NMR (376 MHz, $CDCl_3$):

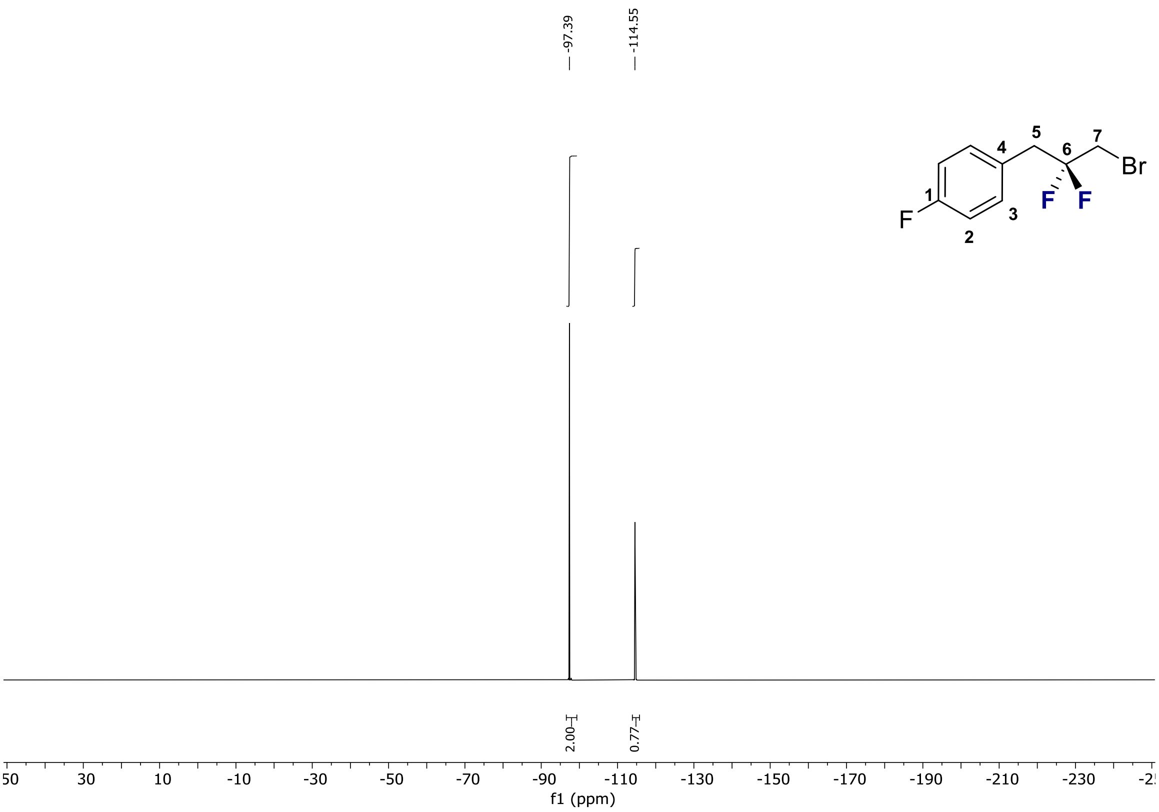

1-(3-Bromo-2,2-difluoropropyl)-4-chlorobenzene (2d)

^{1}H NMR (500 MHz, $CDCl_3$):

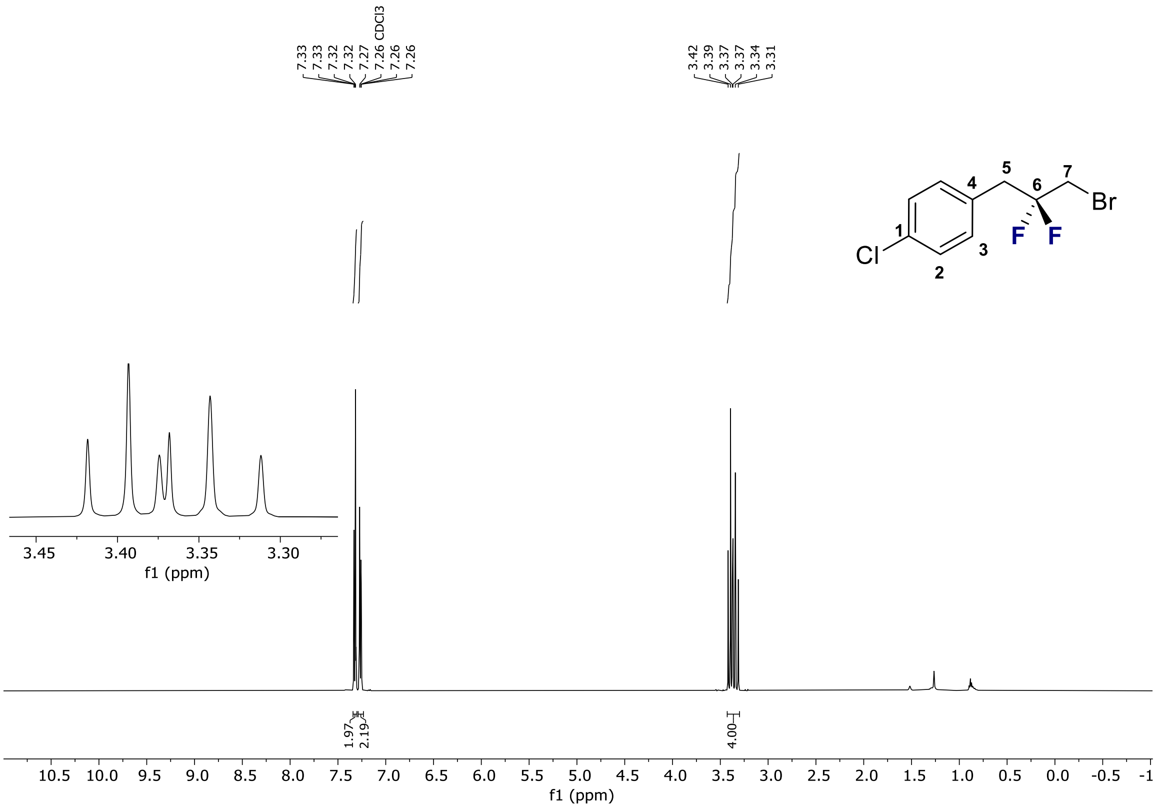

^{13}C{^{1}H} NMR (126 MHz, $CDCl_3$):

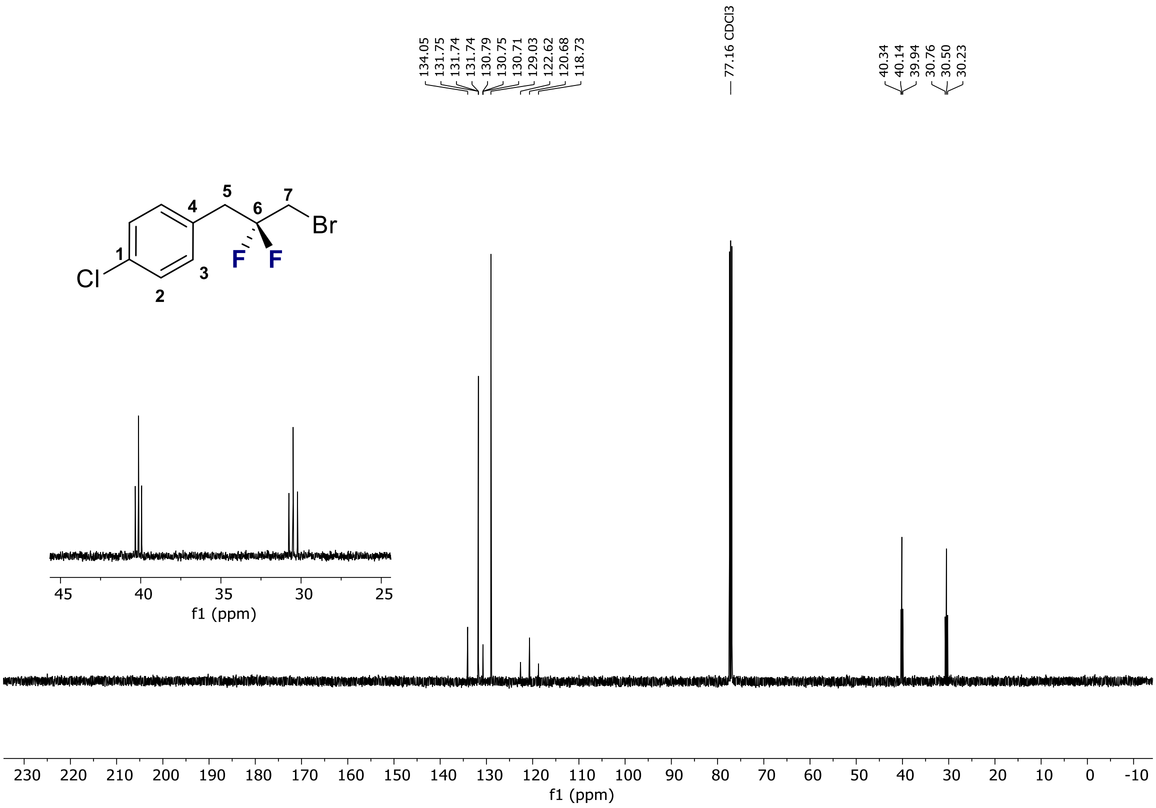

^{19}**F NMR** (376 MHz, $CDCl_3$):

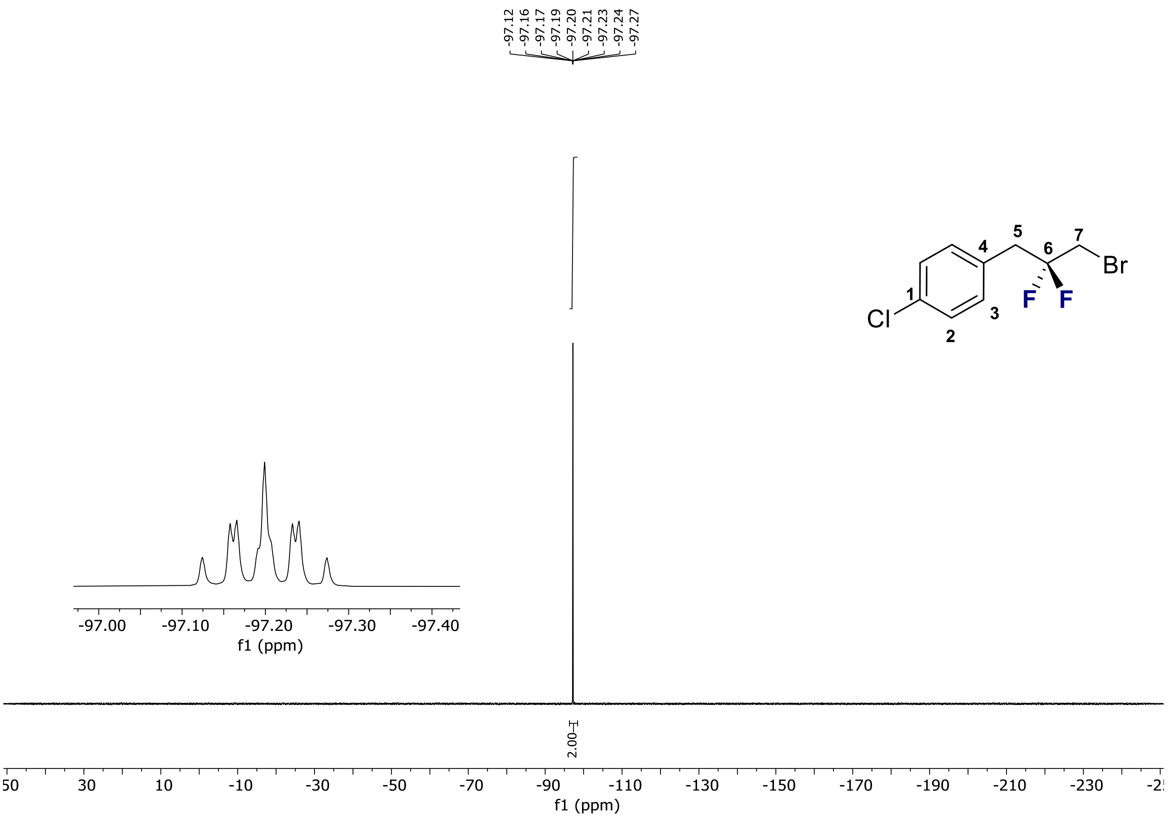

^{19}**F{^{1}H} NMR** (376 MHz, $CDCl_3$):

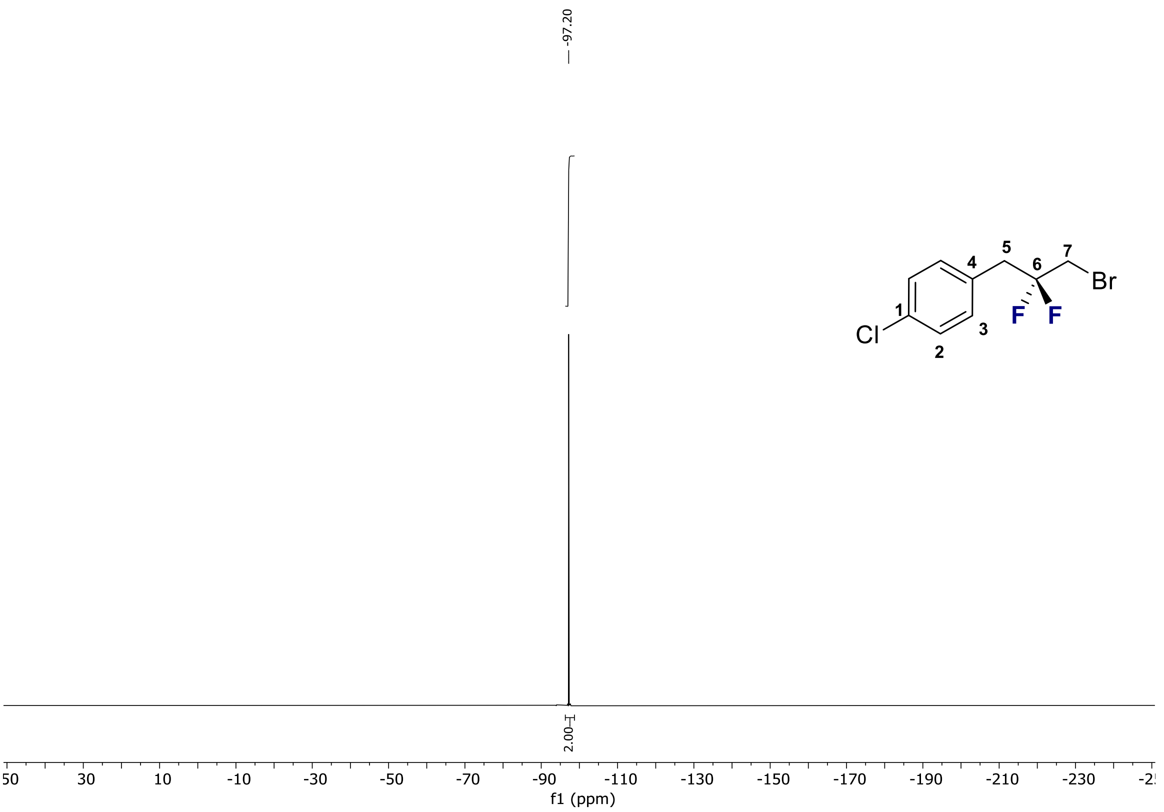

1-Bromo-3-(3-bromo-2,2-difluoropropyl)benzene (2e)

^{1}H NMR (500 MHz, $CDCl_3$):

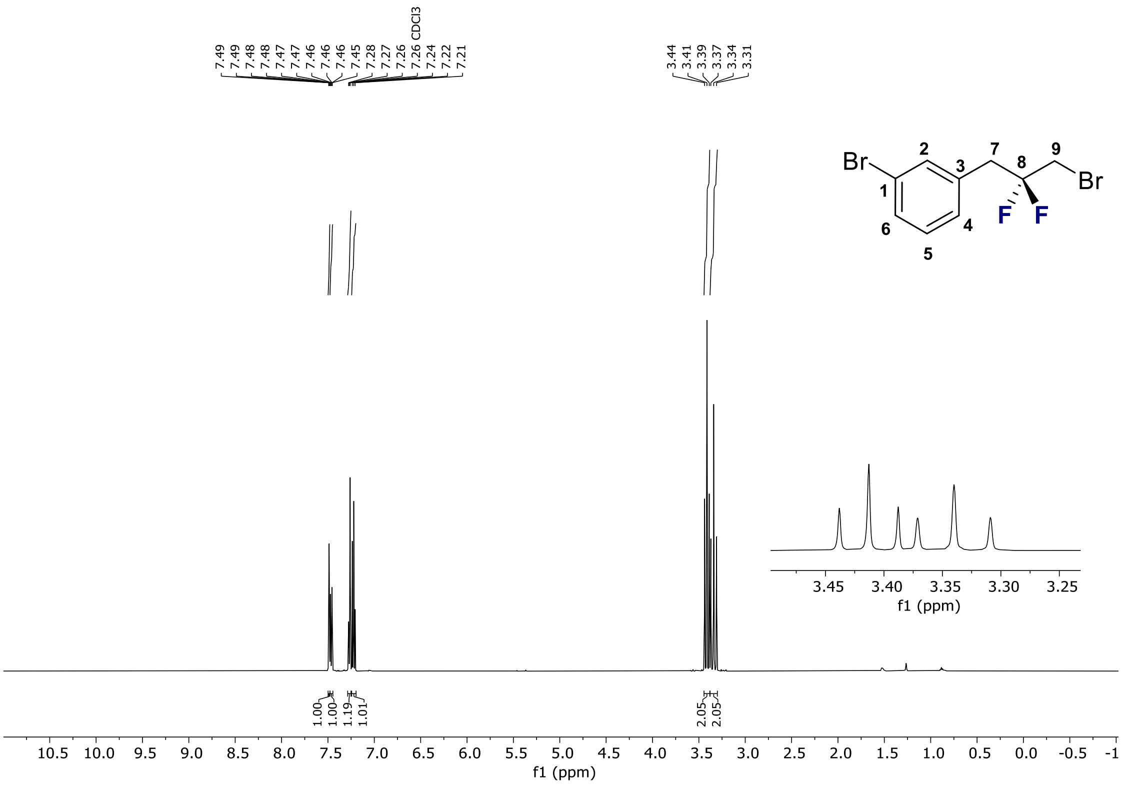

^{13}C NMR (126 MHz, $CDCl_3$):

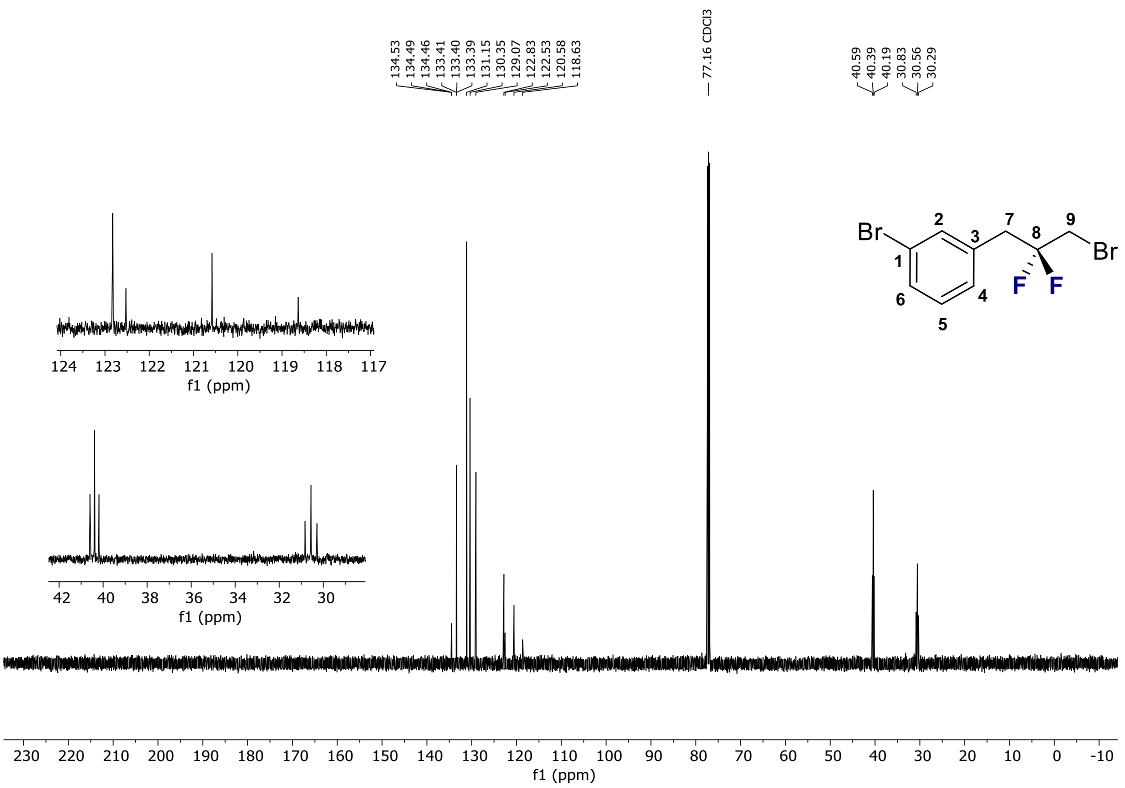

^{19}F NMR (376 MHz, $CDCl_3$):

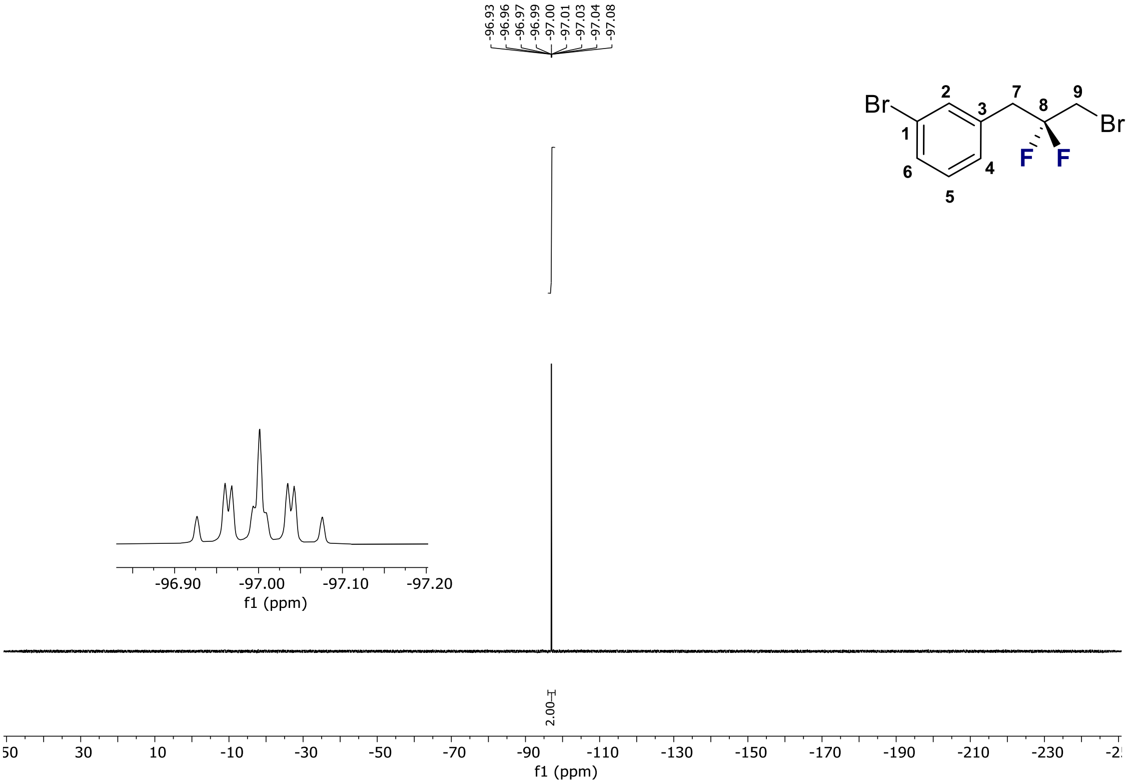

^{19}F{^{1}H} NMR (376 MHz, $CDCl_3$):

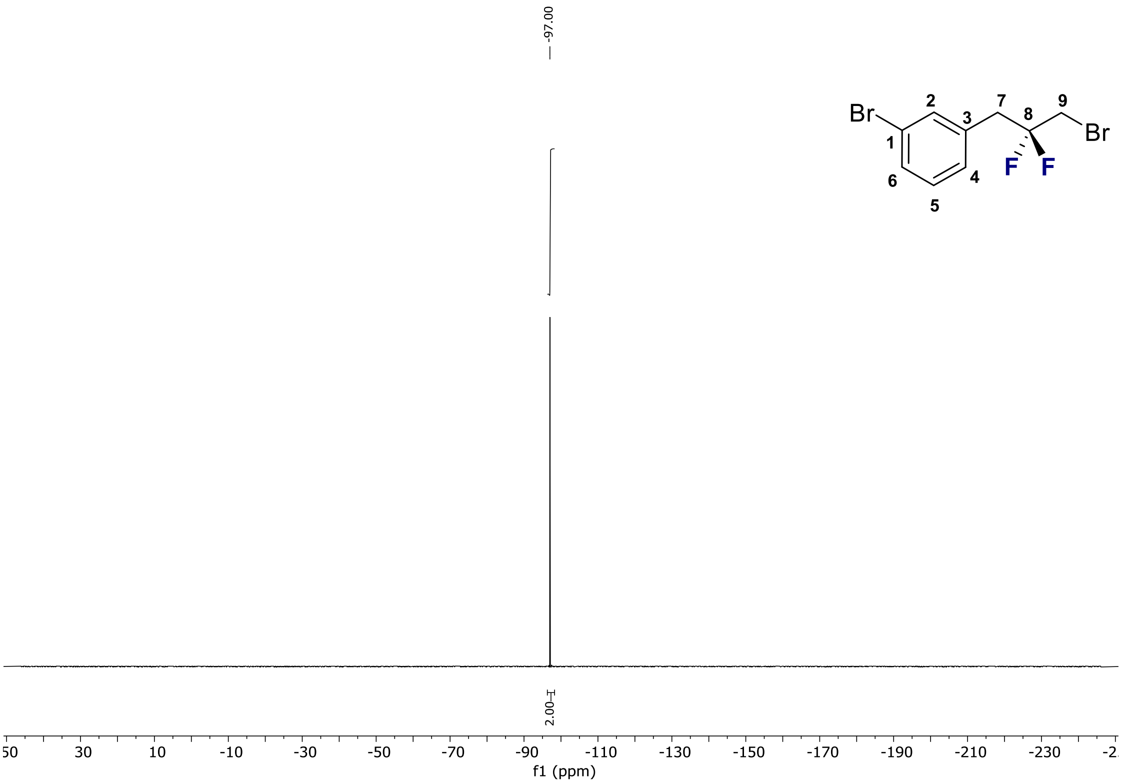

1-Bromo-2-(3-bromo-2,2-difluoropropyl)benzene (2f)

^{1}H NMR (500 MHz, $CDCl_3$):

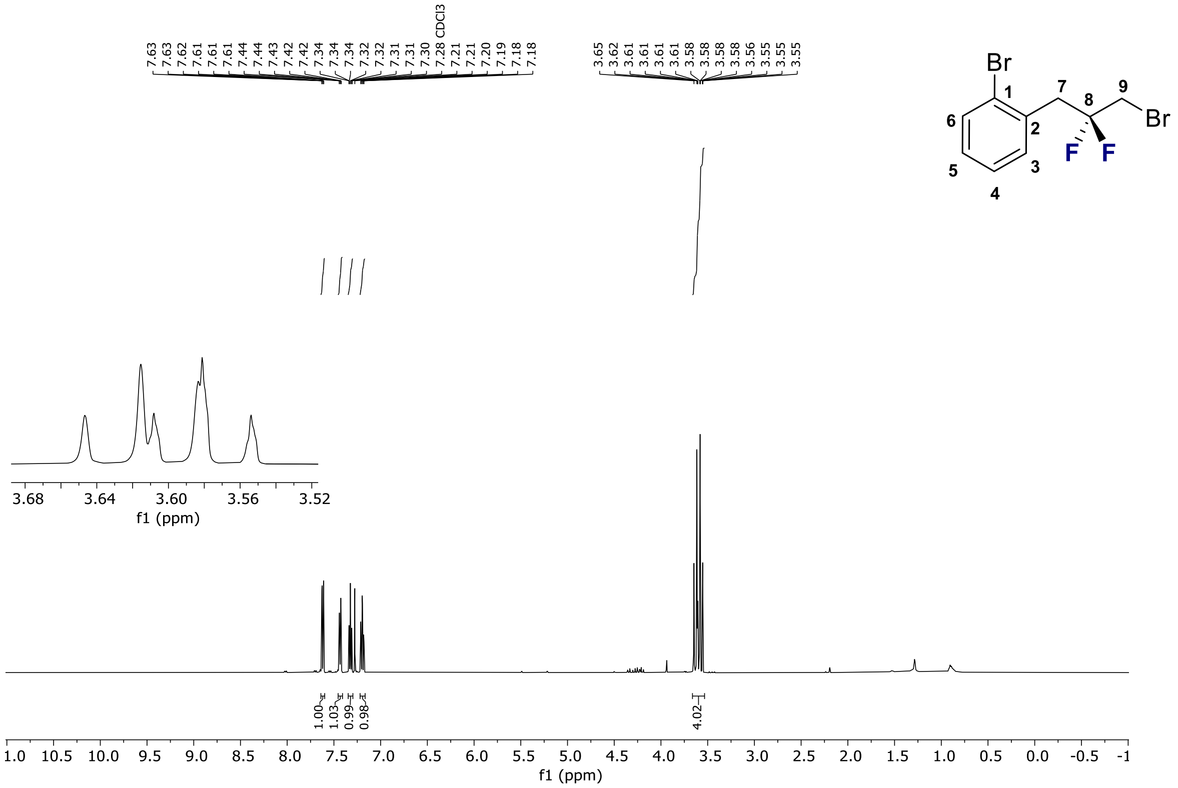

^{13}C NMR (126 MHz, $CDCl_3$):

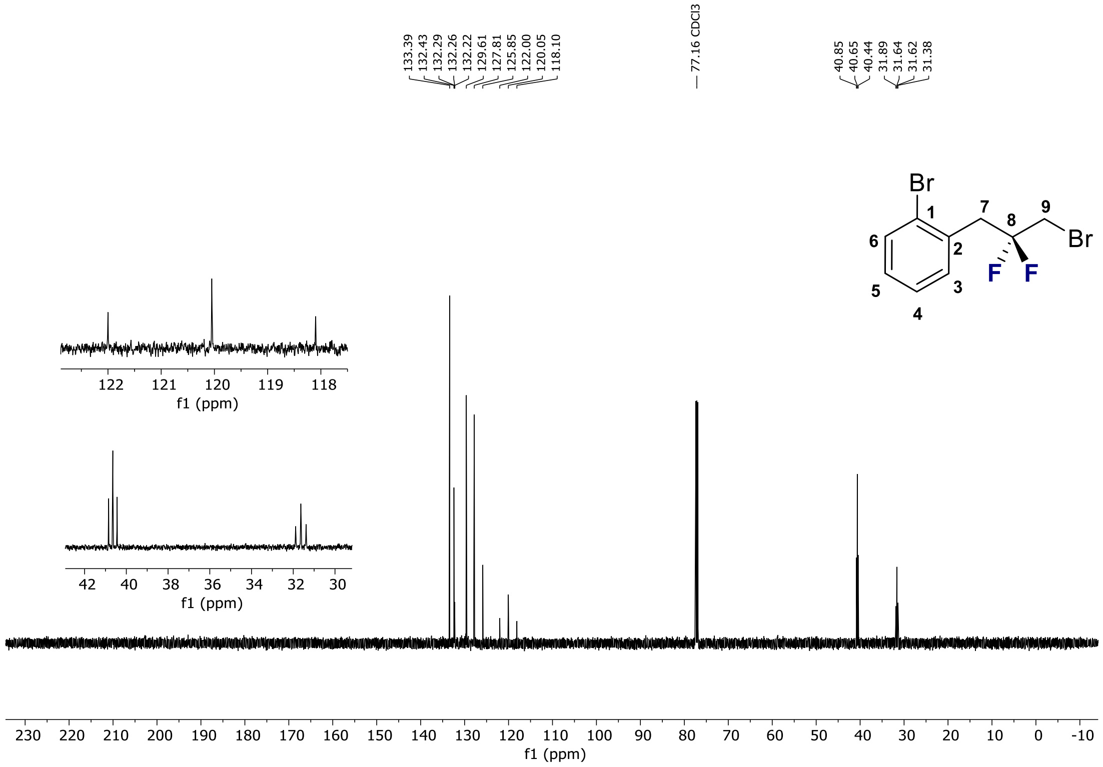

^{19}F NMR (376 MHz, $CDCl_3$):

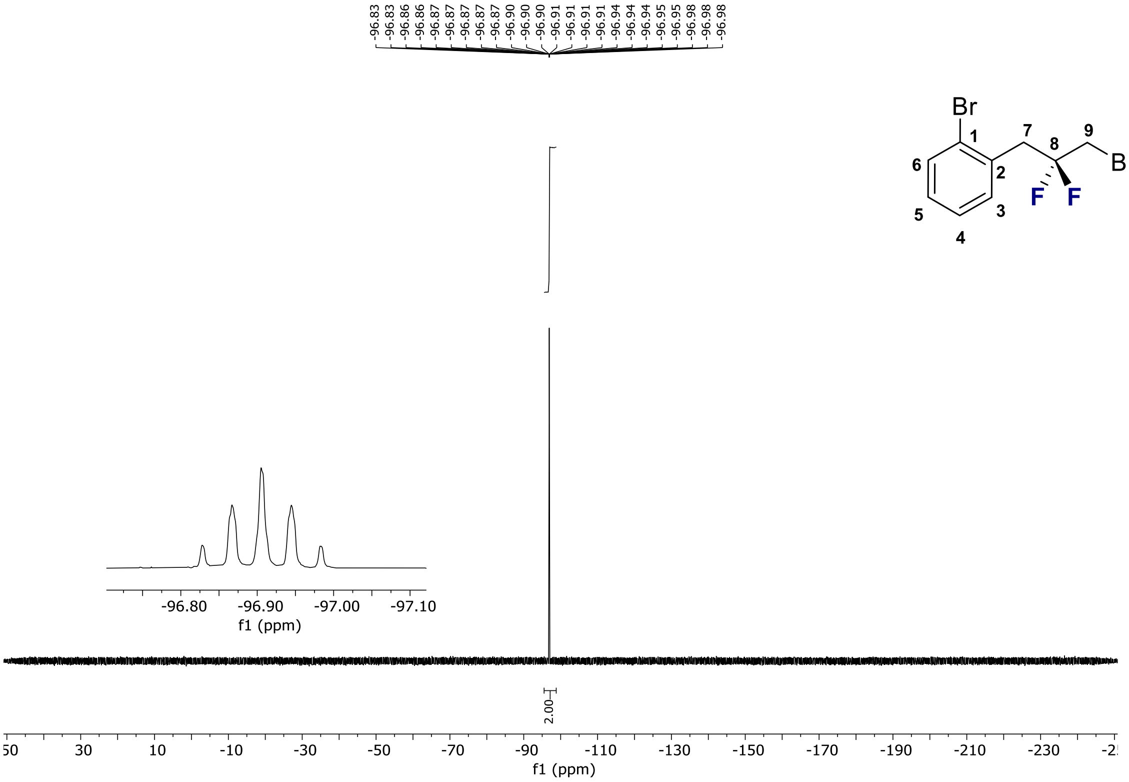

^{19}F{^{1}H} NMR (376 MHz, $CDCl_3$):

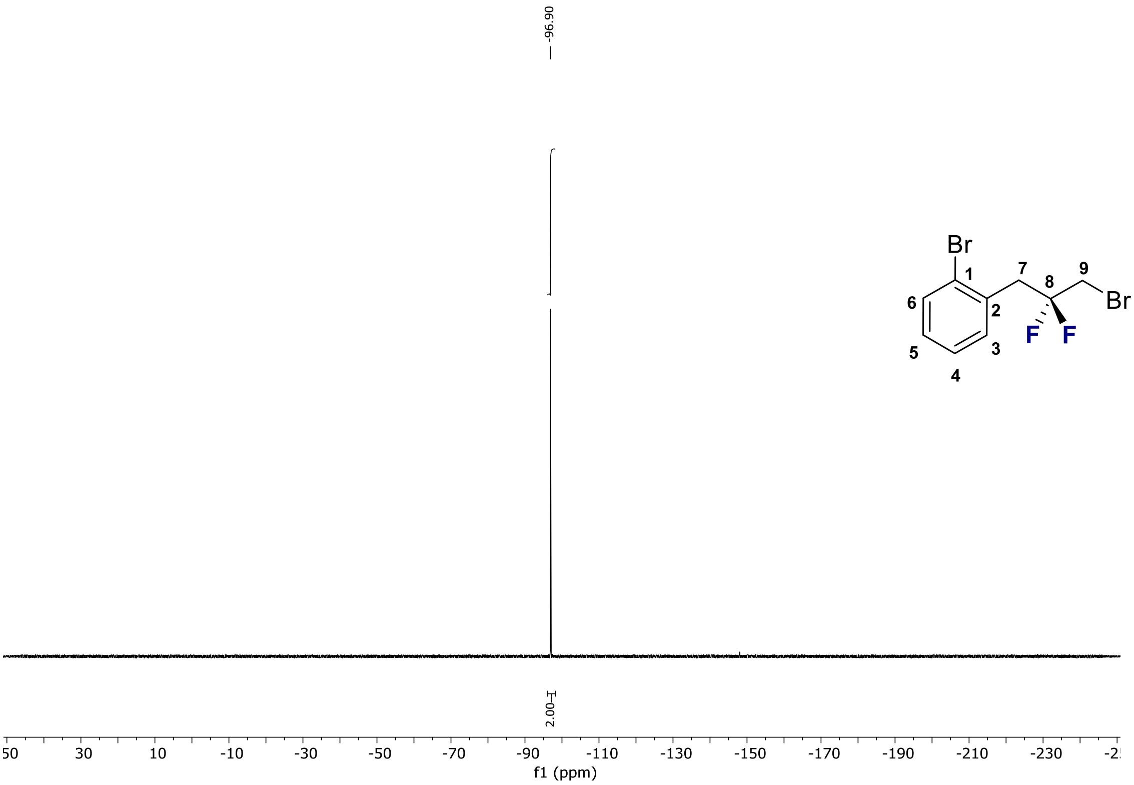

1-(3-Bromo-2,2-difluoropropyl)-4-(*tert*-butyl)benzene (2g)

^{1}H NMR (500 MHz, $CDCl_3$):

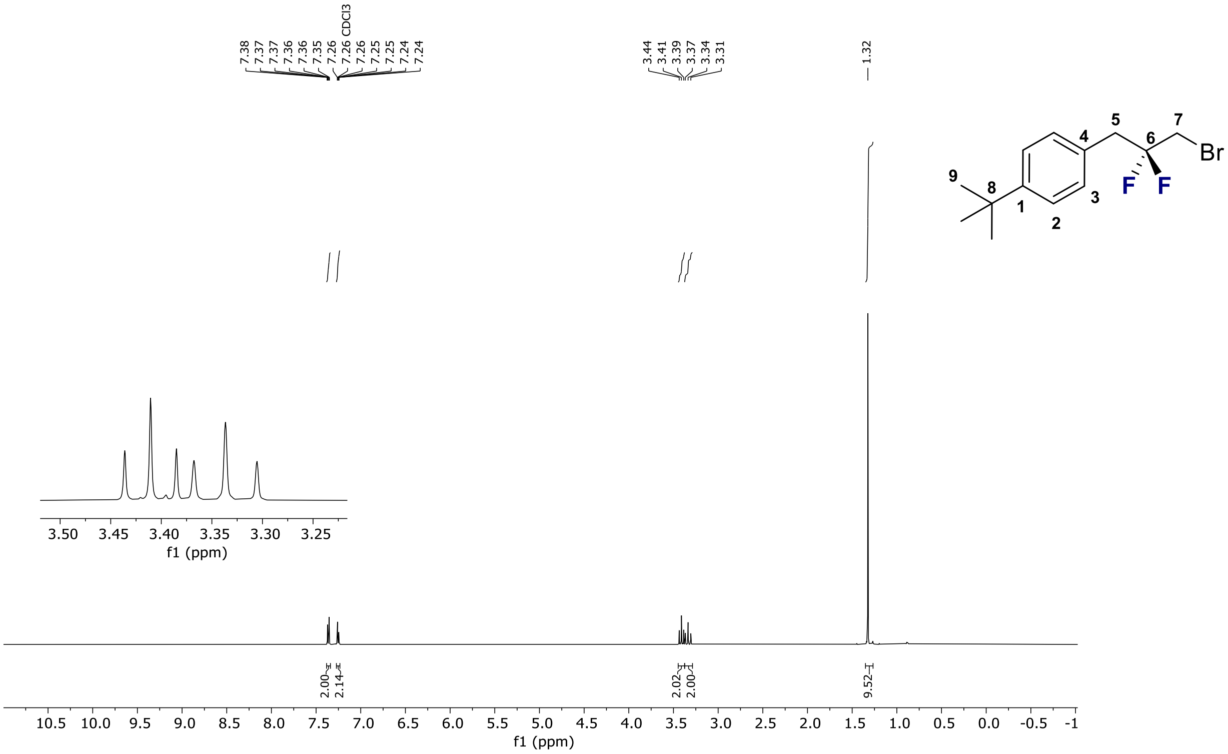

^{13}C{^{1}H} NMR (126 MHz, $CDCl_3$):

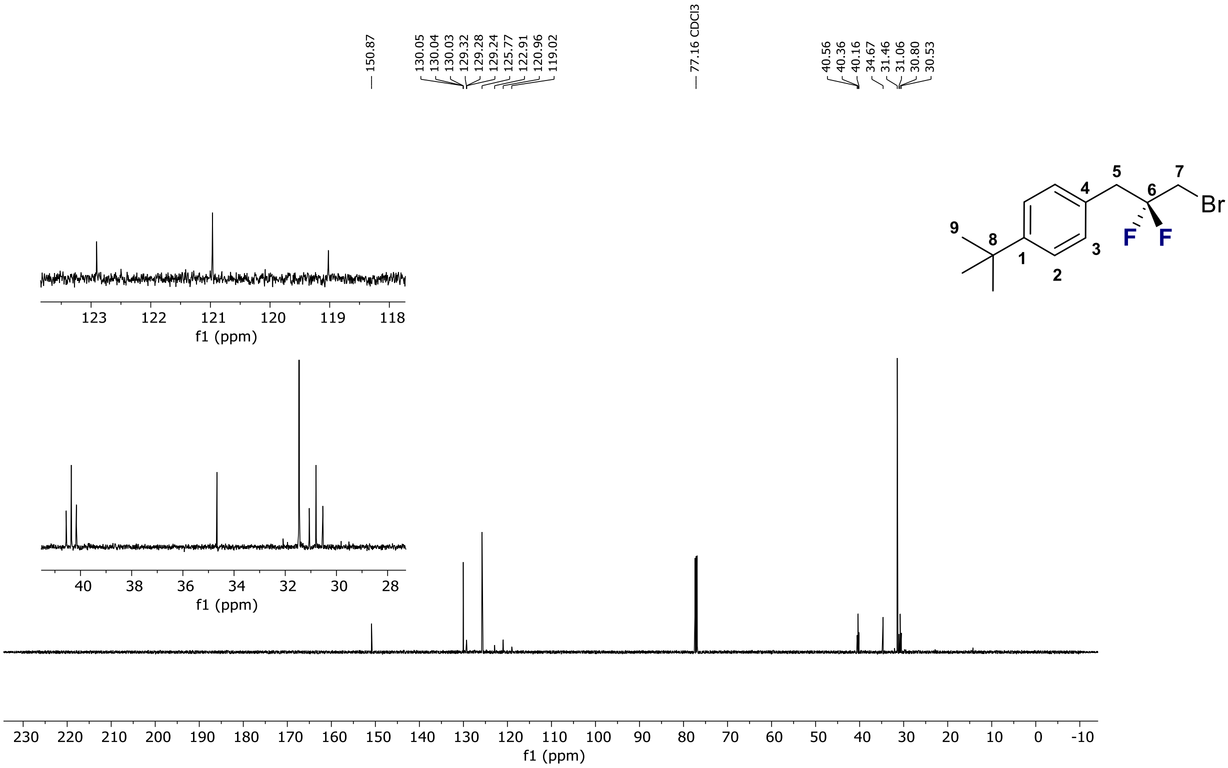

^{19}F NMR (376 MHz, $CDCl_3$):

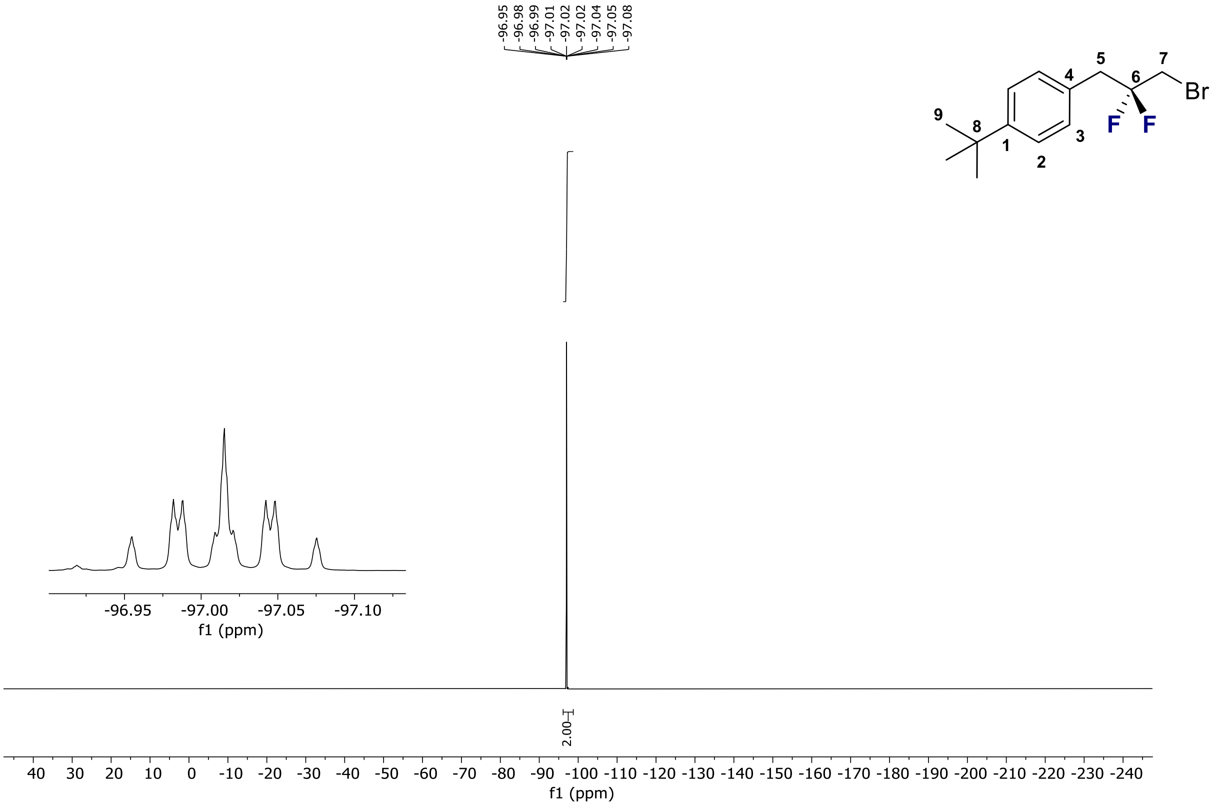

^{19}F{^{1}H} NMR (376 MHz, $CDCl_3$):

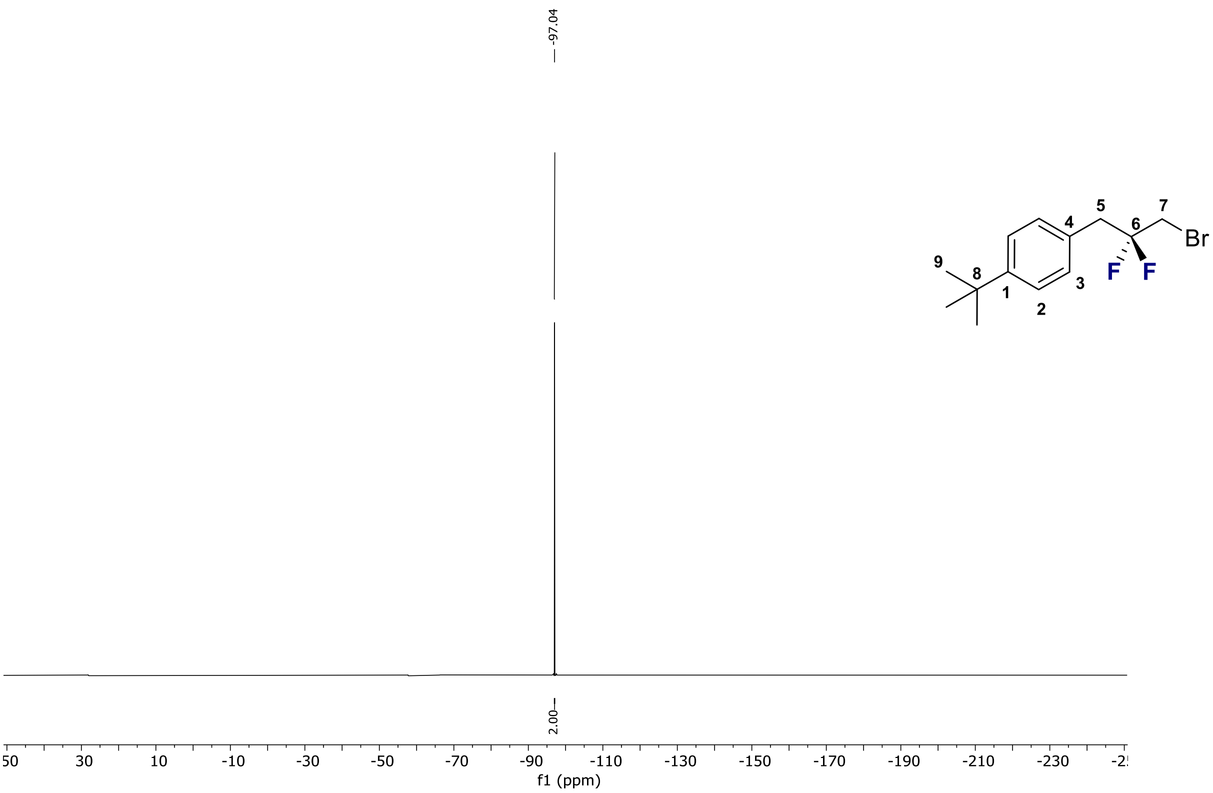

1-(3-Bromo-2,2-difluoropropyl)-4-(trifluoromethyl)benzene (2h)

^{1}H NMR (599 MHz, $CDCl_3$):

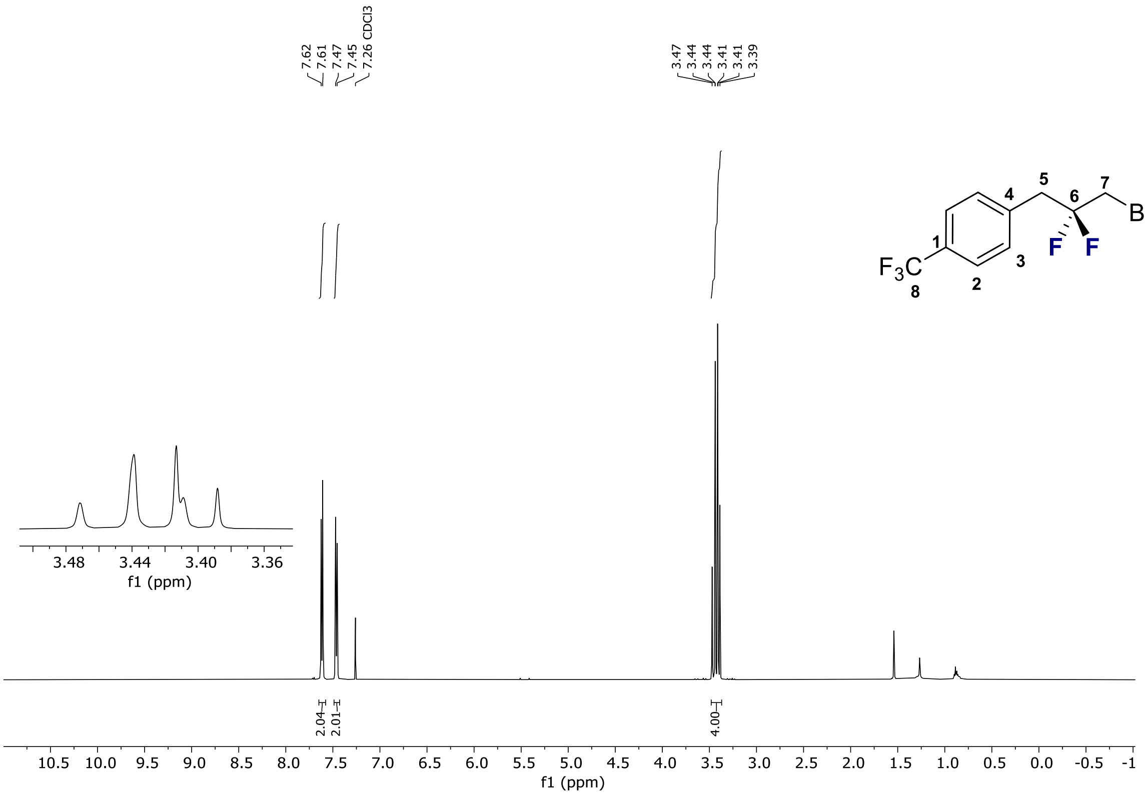

^{13}C{^{1}H} NMR (151 MHz, $CDCl_3$):

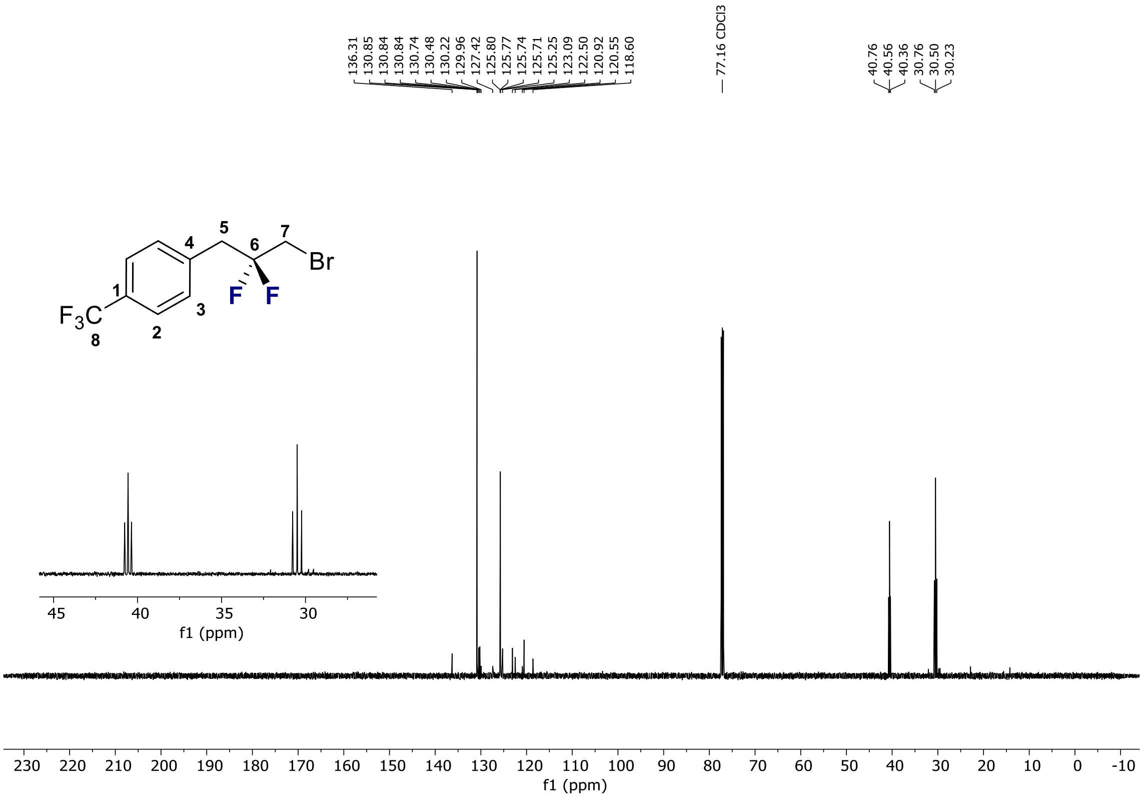

^{19}**F NMR** (376 MHz, $CDCl_3$):

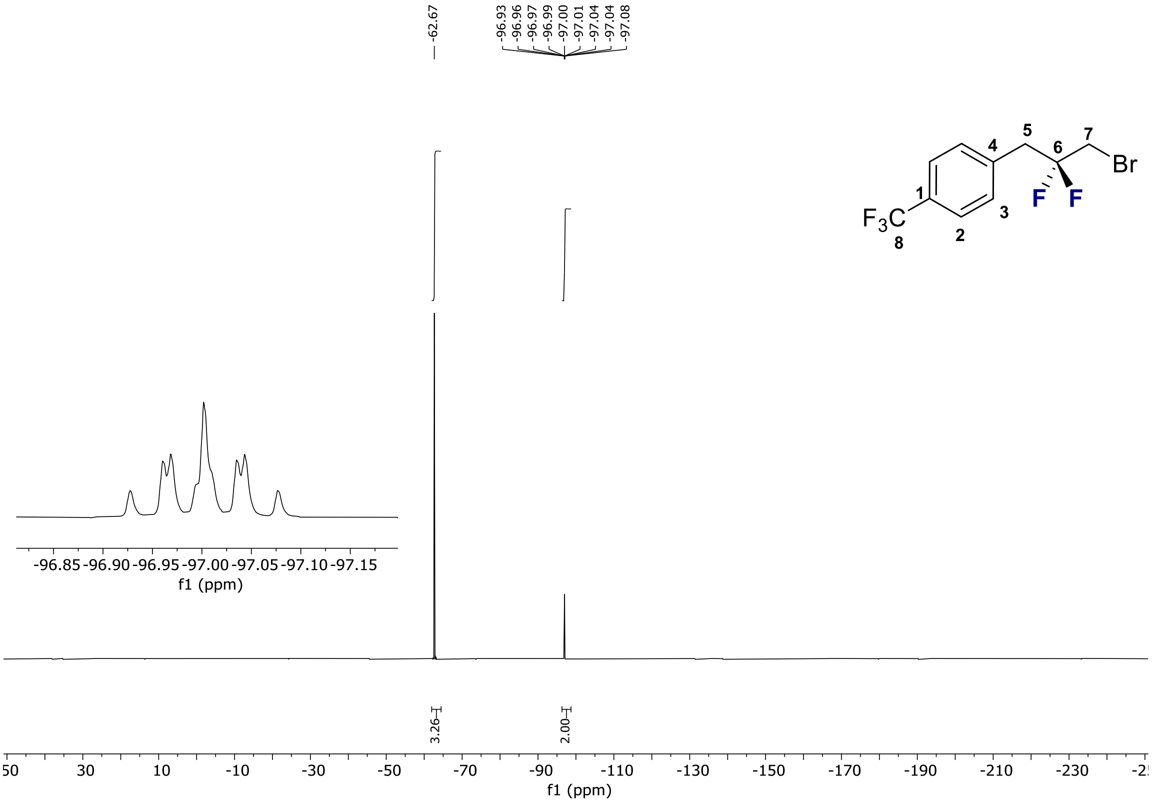

^{19}**F{^{1}H} NMR** (376 MHz, $CDCl_3$):

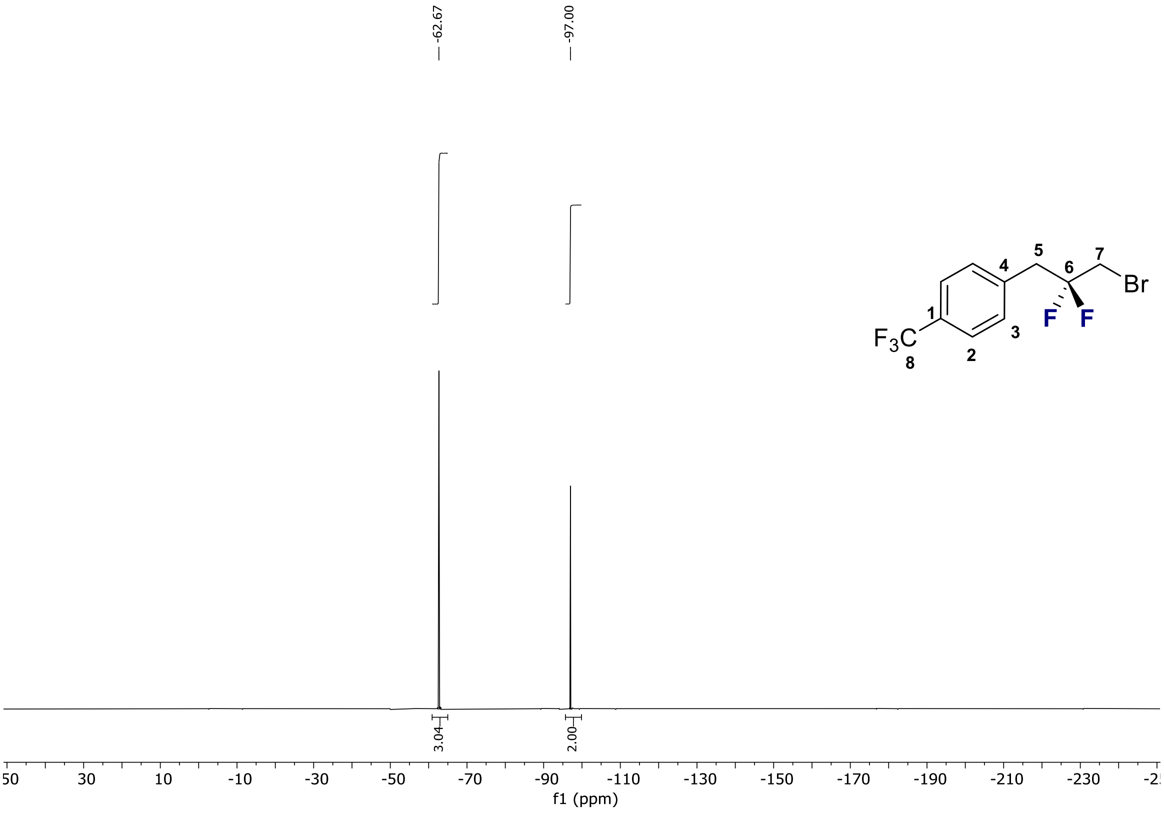

Methyl 4-(3-bromo-2,2-difluoropropyl)benzoate (2i)

^{1}H NMR (500 MHz, $CDCl_3$):

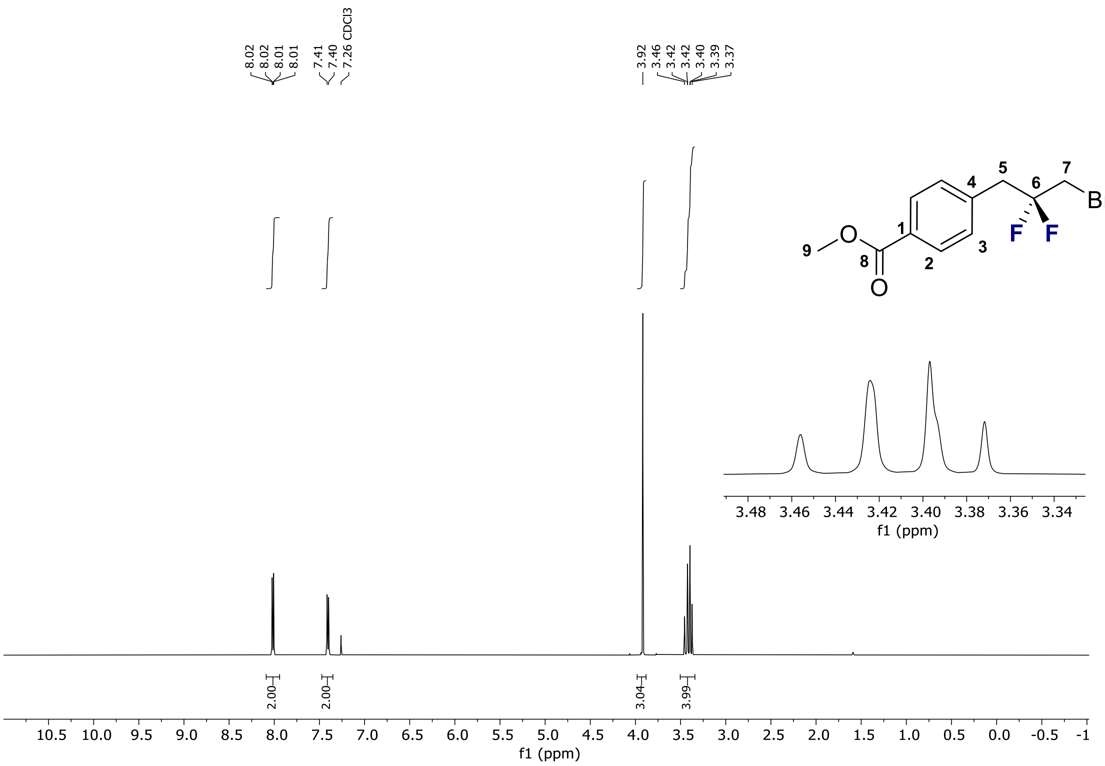

$^{13}C\{^1H\}$ NMR (126 MHz, $CDCl_3$):

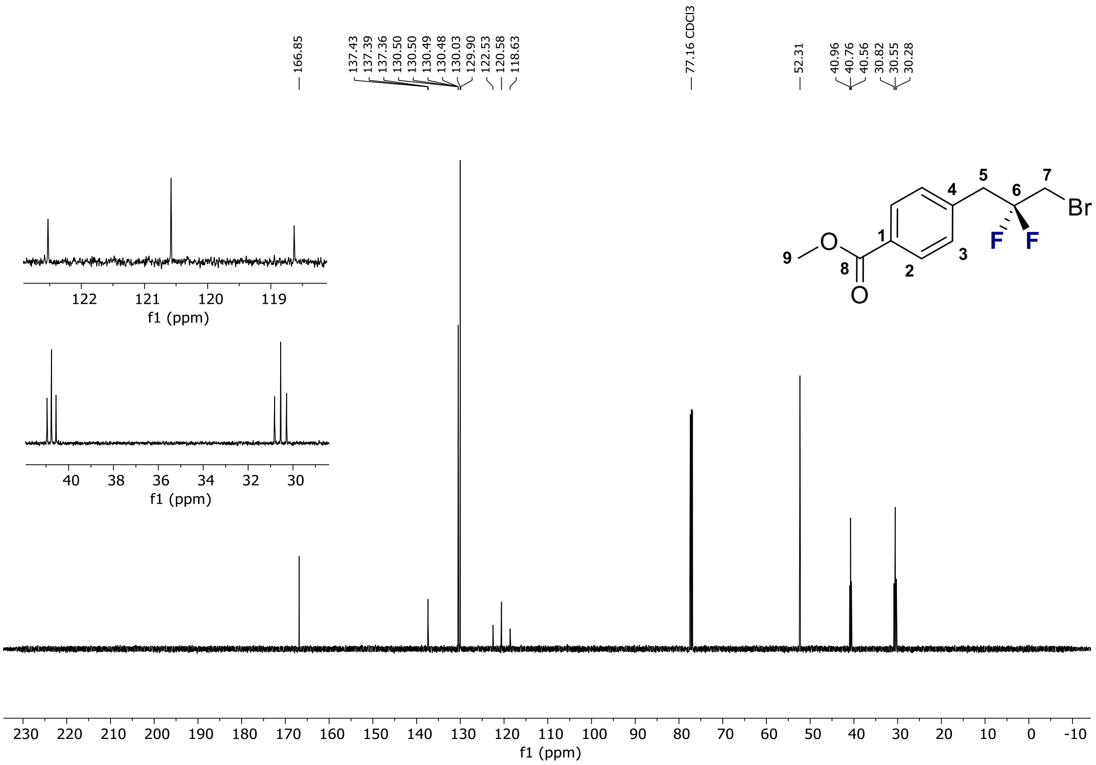

^{19}F NMR (376 MHz, $CDCl_3$):

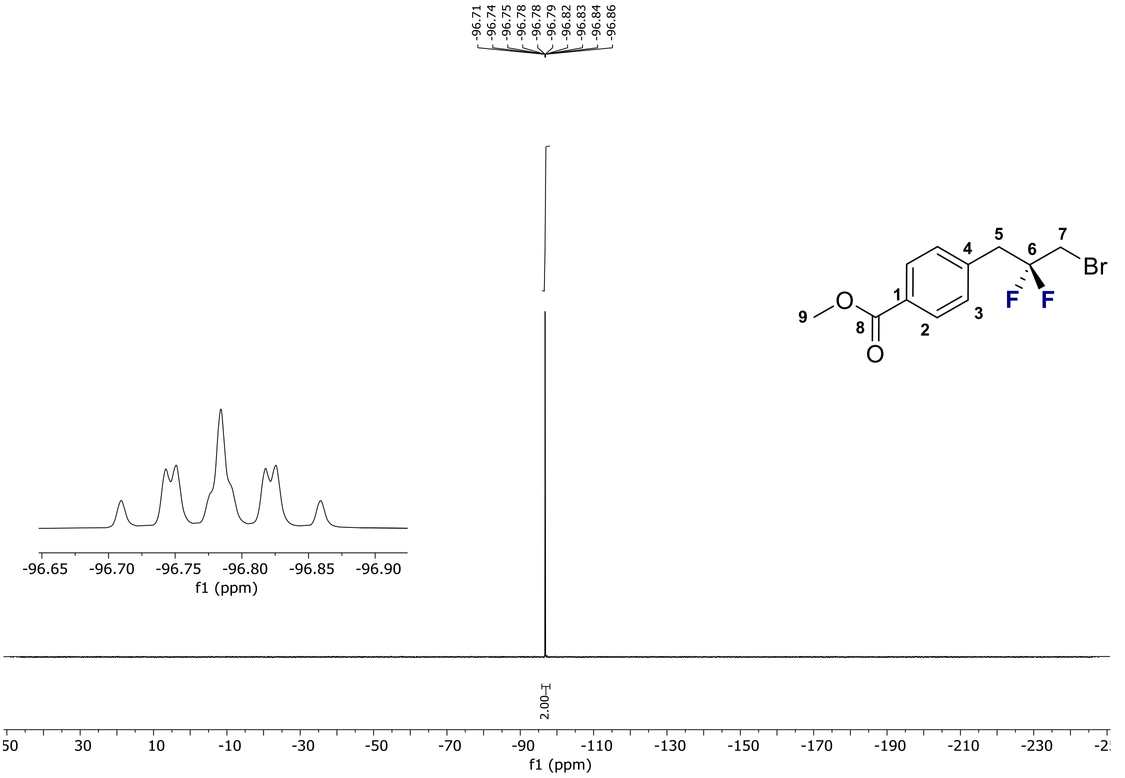

^{19}F{^{1}H} NMR (376 MHz, $CDCl_3$):

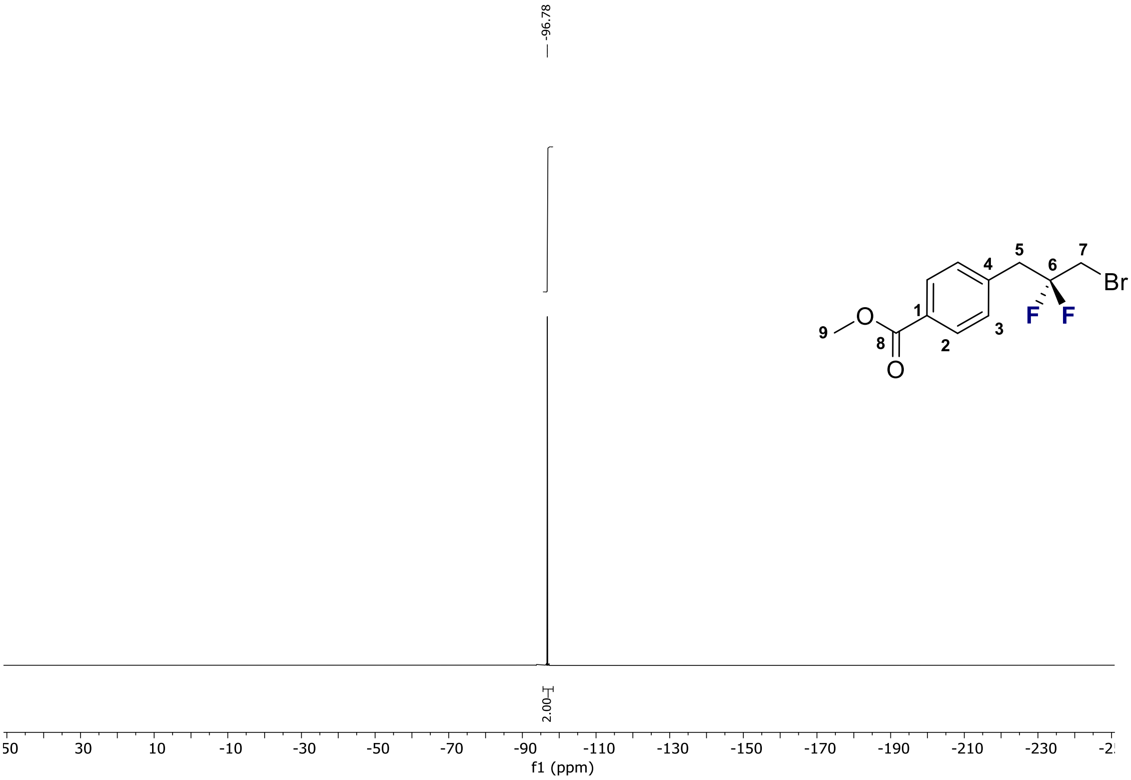

1-(3-Bromo-2,2-difluoropropyl)-4-nitrobenzene (2j)

^{1}H NMR (500 MHz, $CDCl_3$):

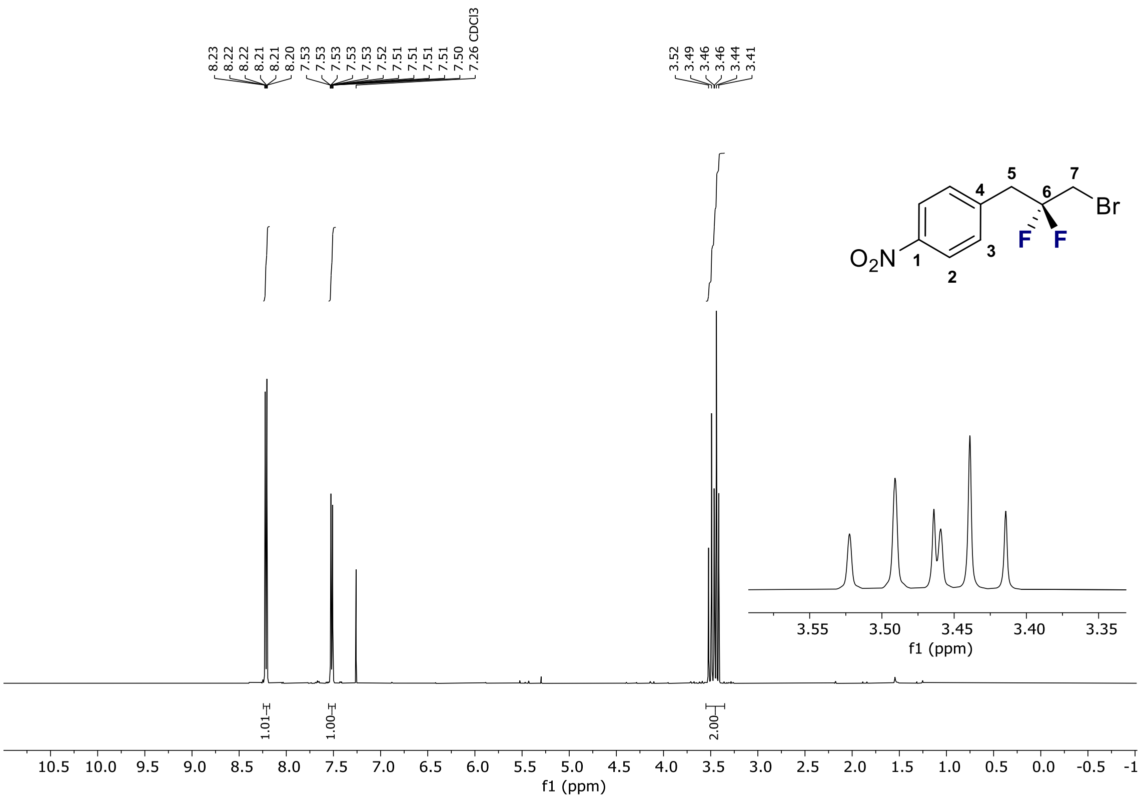

$^{13}C\{^1H\}$ NMR (126 MHz, $CDCl_3$):

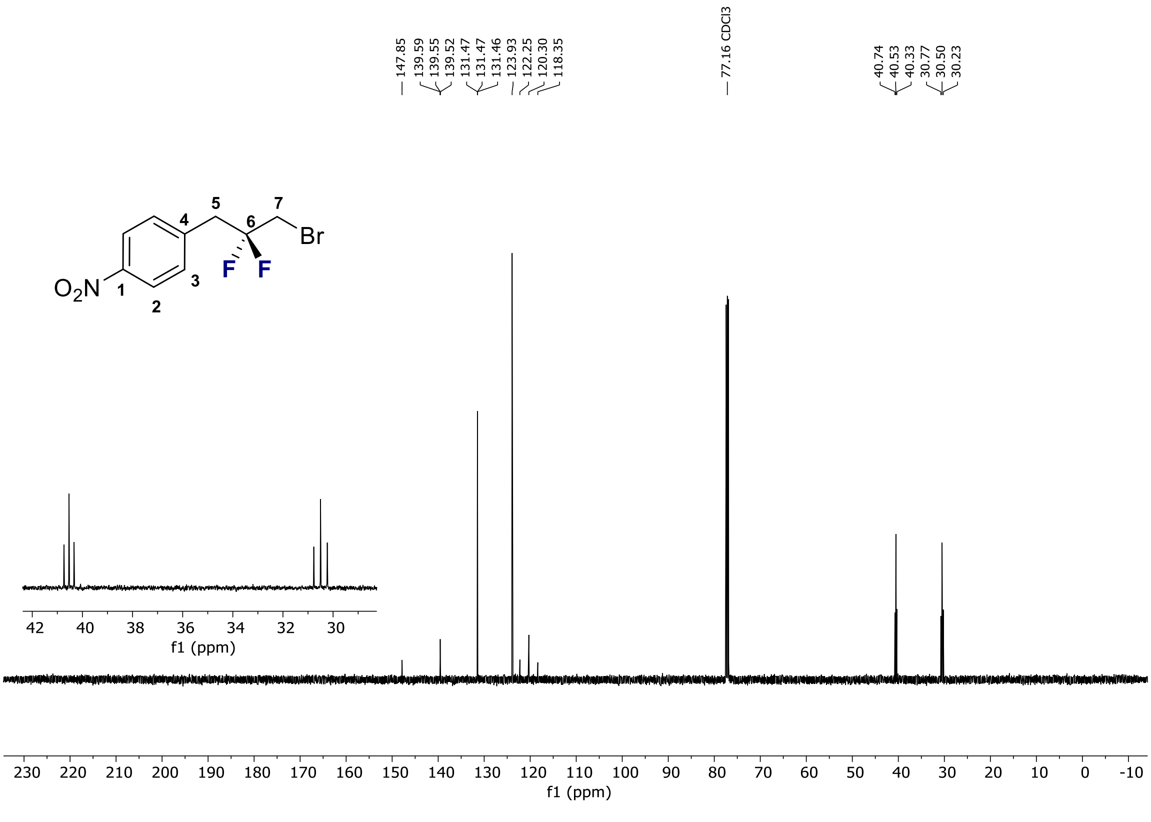

^{19}F NMR (376 MHz, $CDCl_3$):

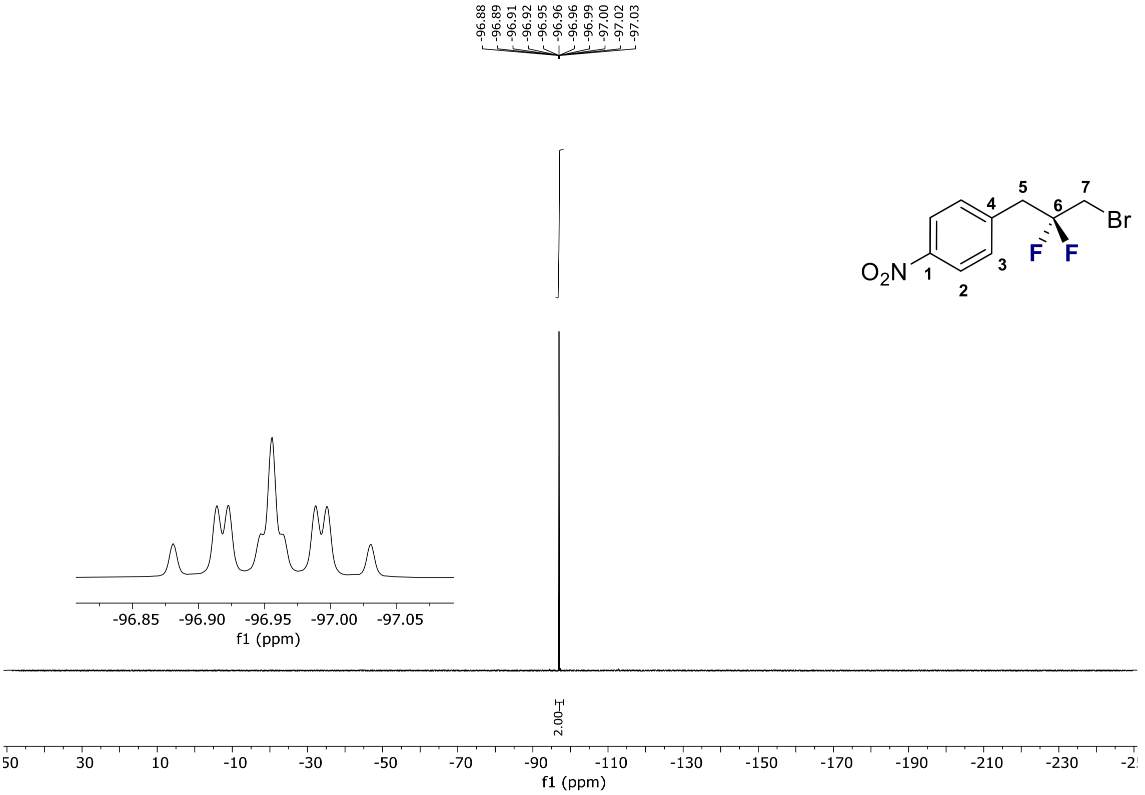

^{19}F{^{1}H} NMR (376 MHz, $CDCl_3$):

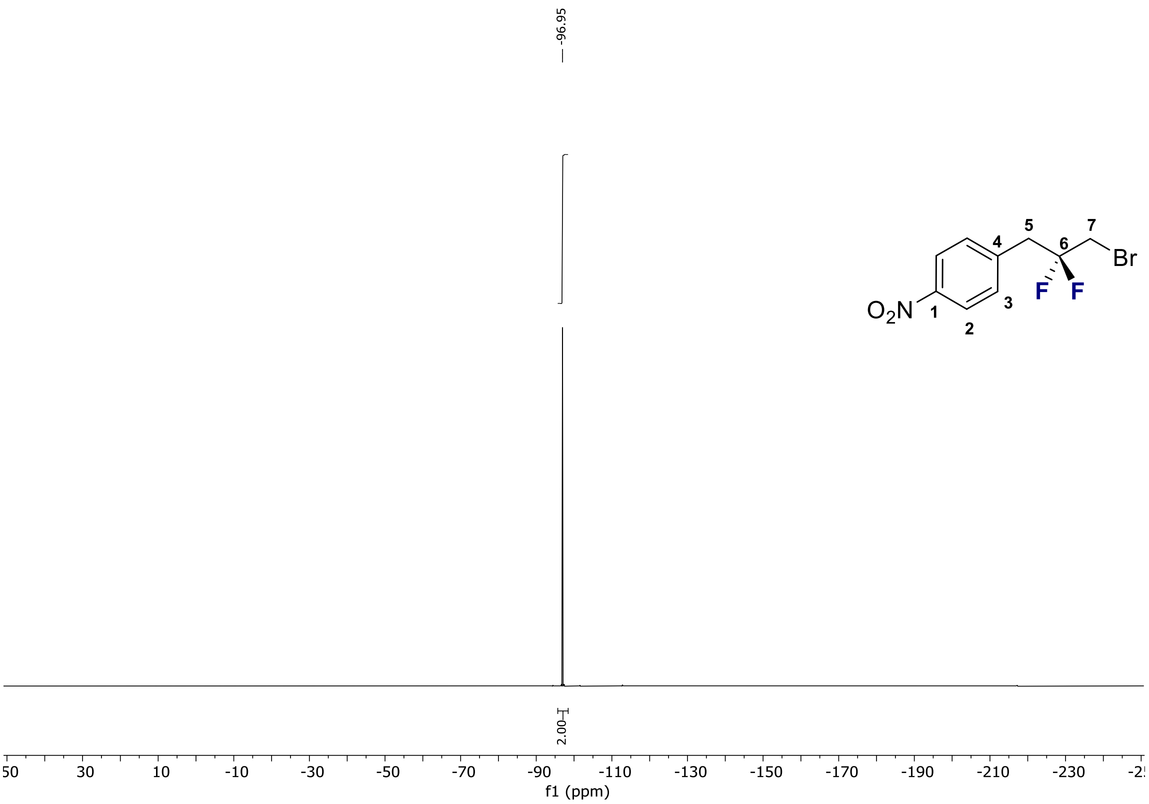

4-(3-Bromo-2,2-difluoropropyl)phenyl 4-methylbenzenesulfonate (2k)

^{1}H NMR (599 MHz, $CDCl_3$):

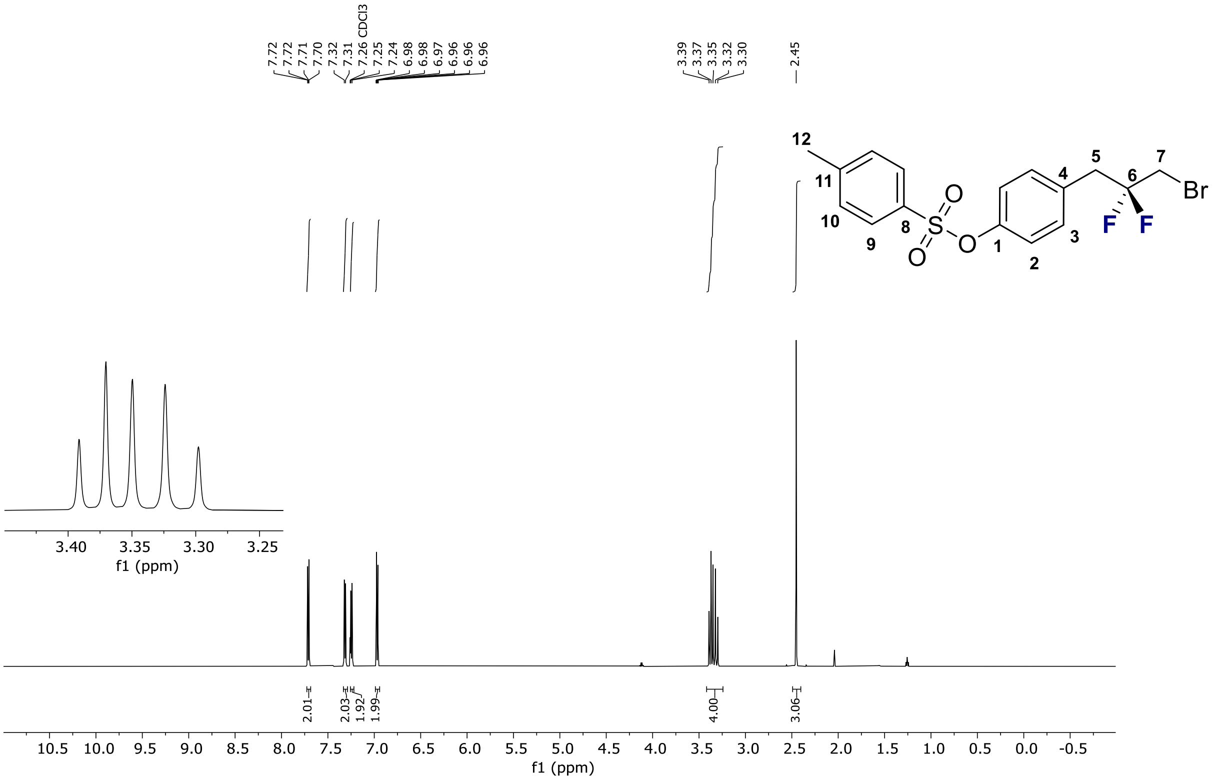

^{13}C{^{1}H} NMR (151 MHz, $CDCl_3$):

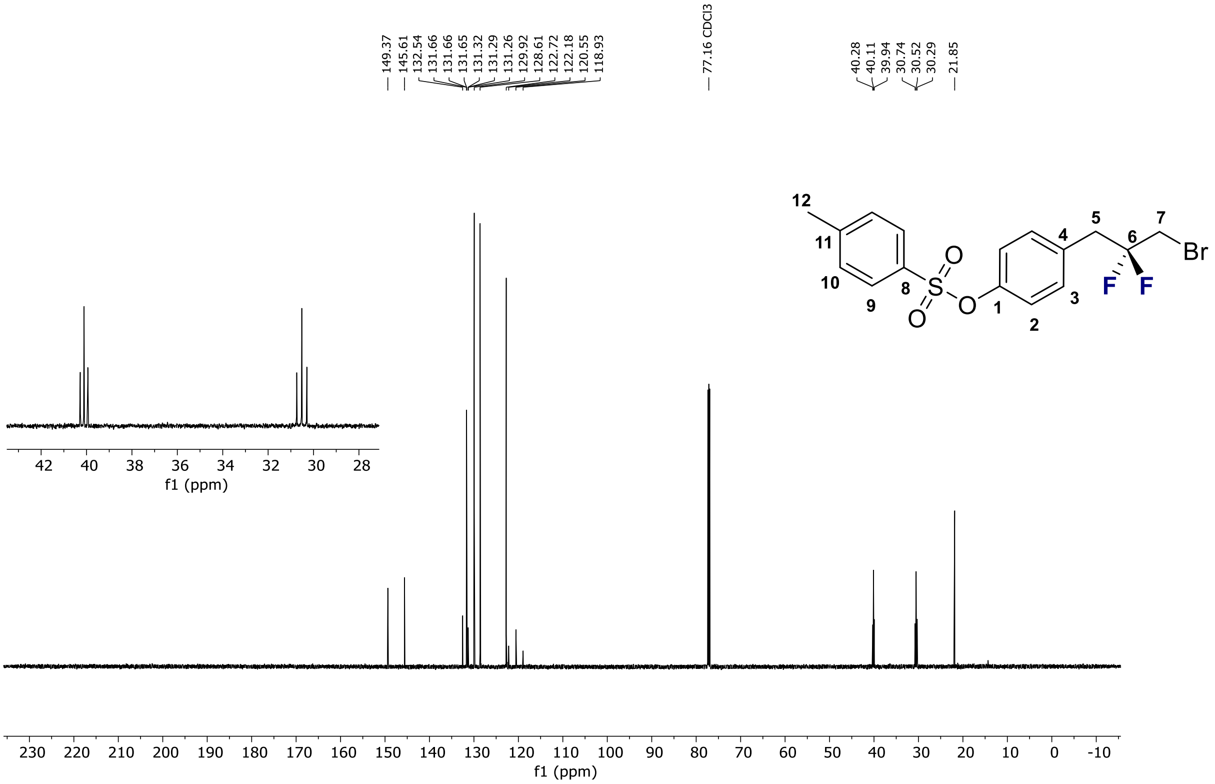

^{19}F NMR (376 MHz, $CDCl_3$):

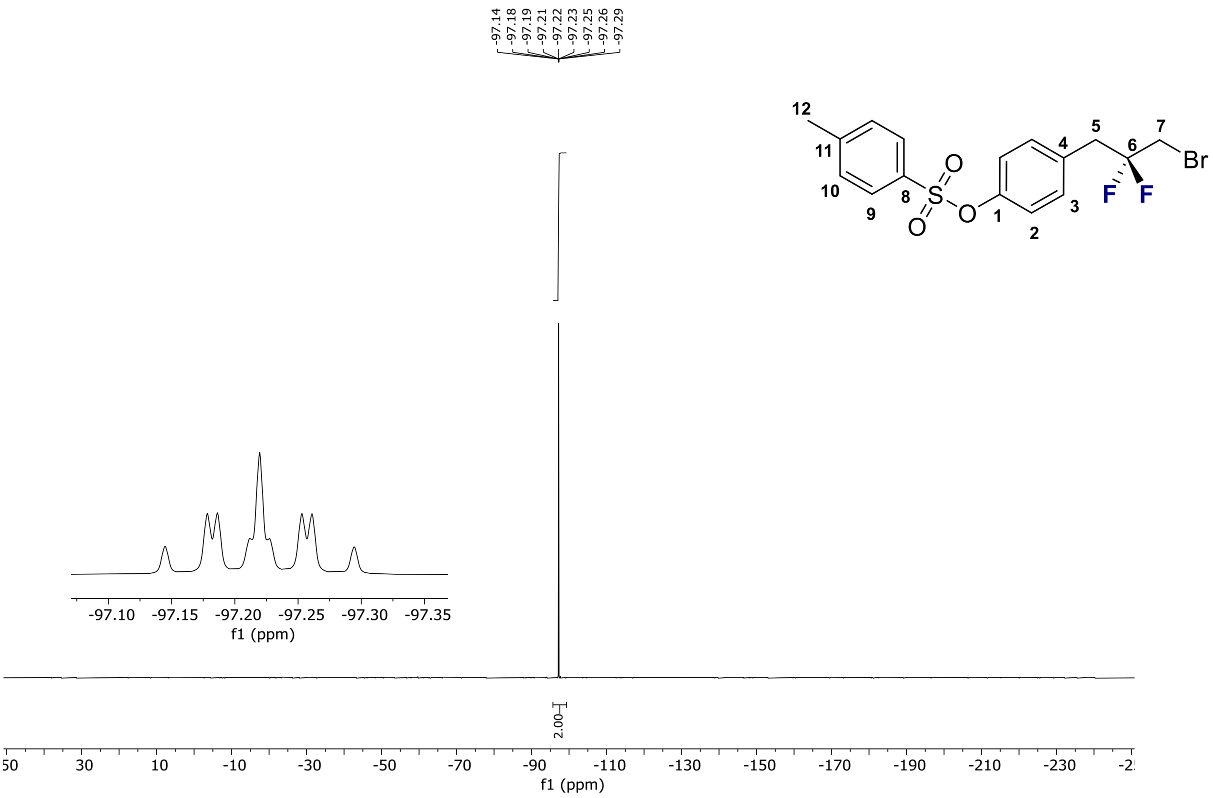

^{19}F{^{1}H} NMR (376 MHz, $CDCl_3$):

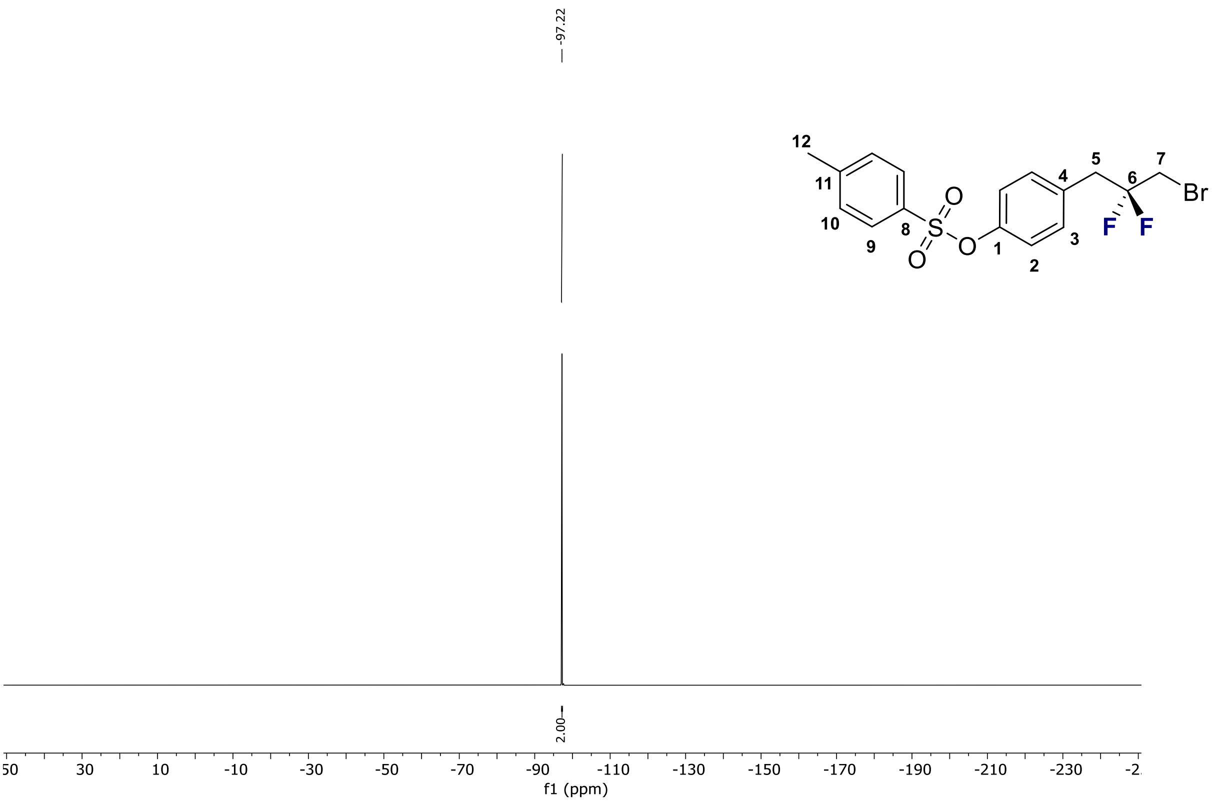

2-Bromo-4-(3-bromo-2,2-difluoropropyl)-1-fluorobenzene (2l)

^{1}H NMR (500 MHz, $CDCl_3$):

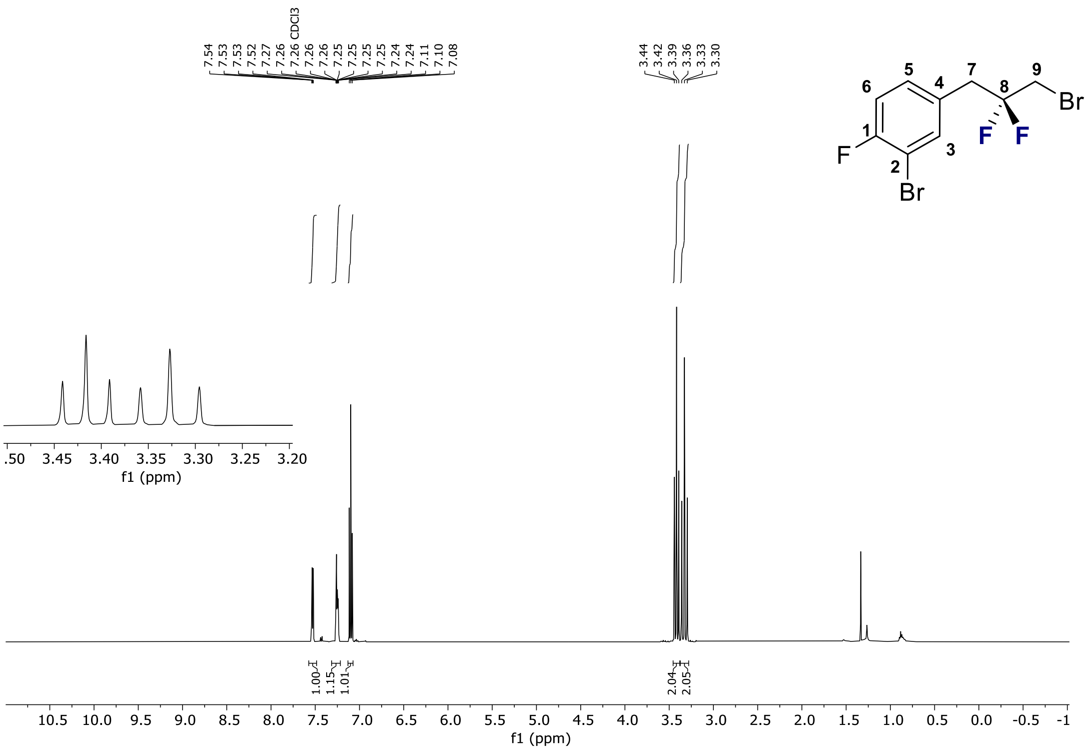

$^{13}C\{^1H\}$ NMR (126 MHz, $CDCl_3$):

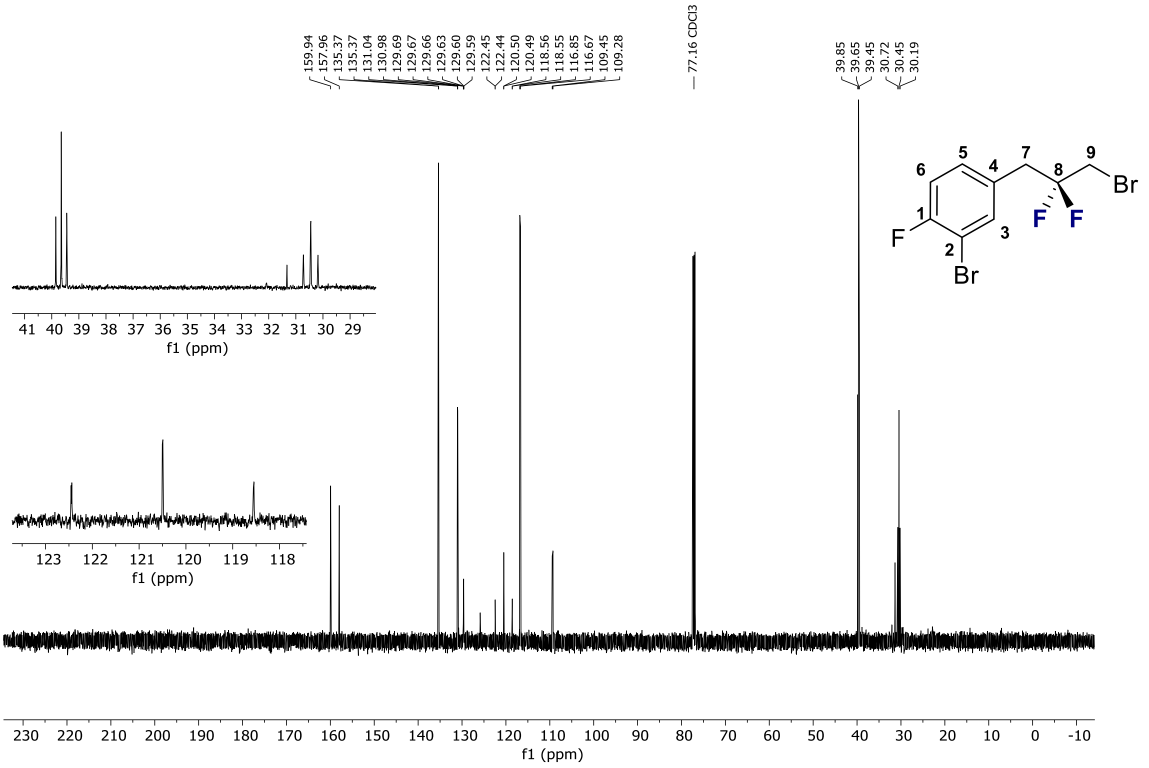

^{19}F NMR (376 MHz, $CDCl_3$):

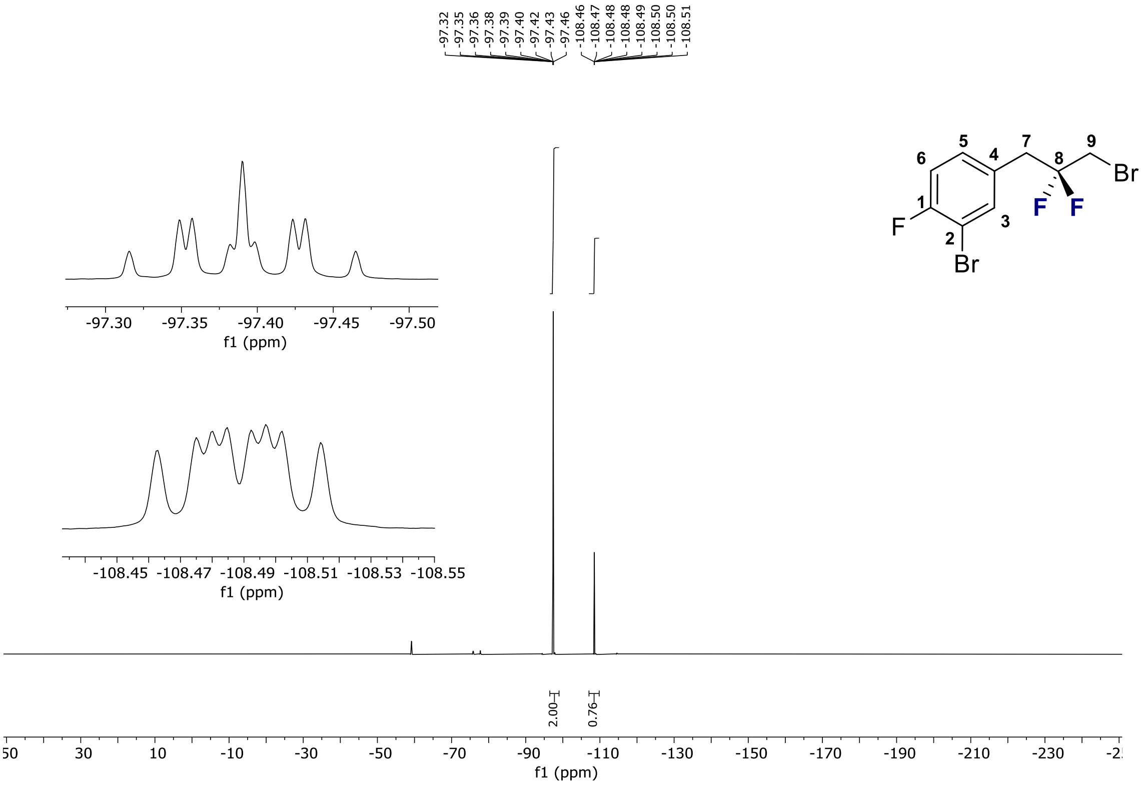

^{19}F{^{1}H} NMR (376 MHz, $CDCl_3$):

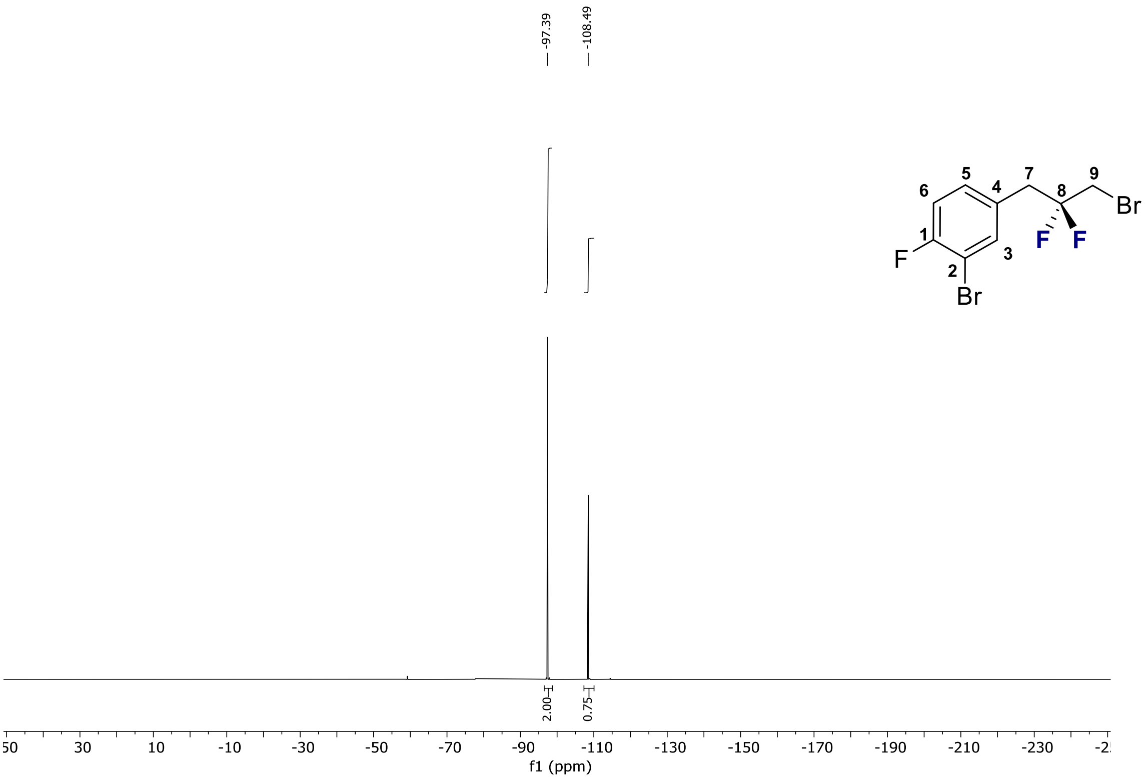

1-((4-(3-Bromo-2,2-difluoropropyl)phenyl)sulfonyl)piperidine (2m)

^{1}H NMR (500 MHz, $CDCl_3$):

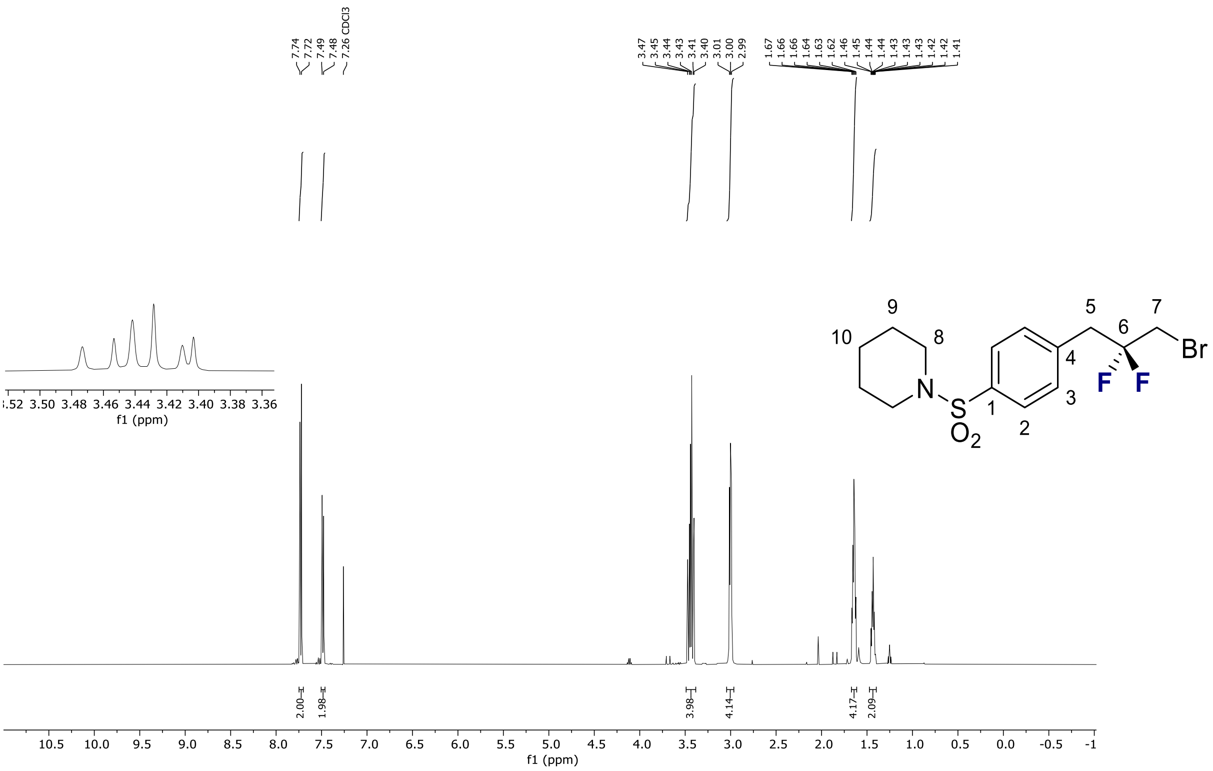

$^{13}C\{^1H\}$ NMR (126 MHz, $CDCl_3$):

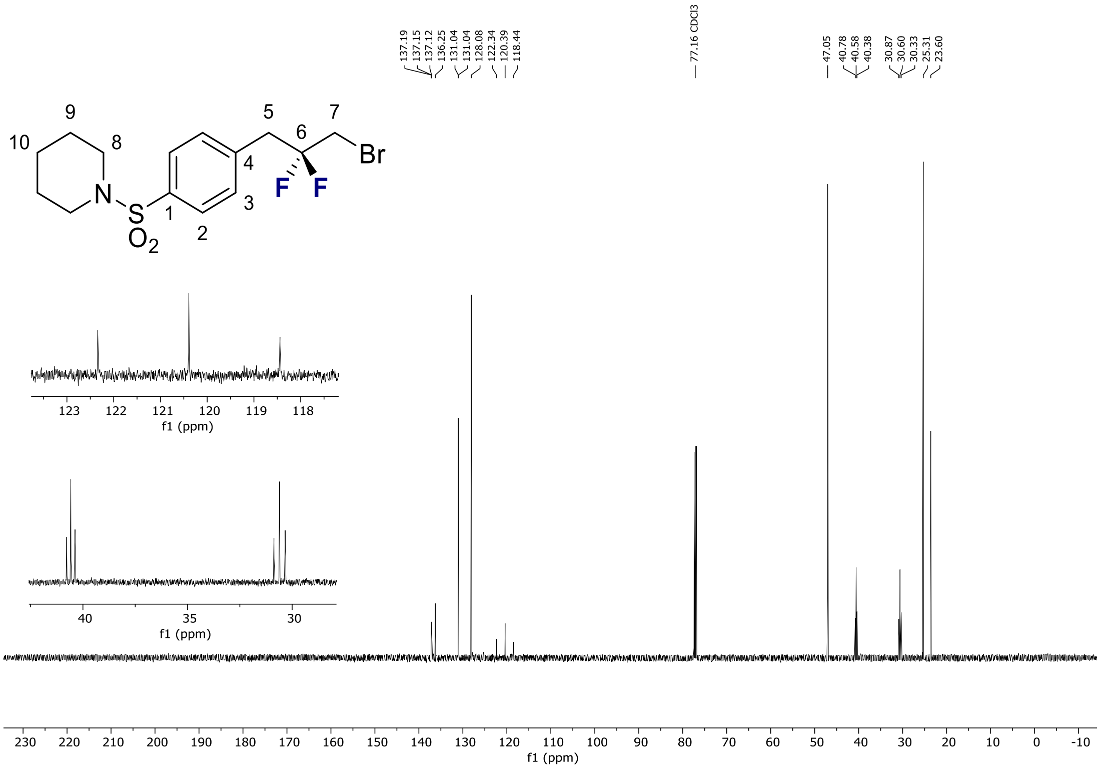

^{19}F NMR (376 MHz, $CDCl_3$):

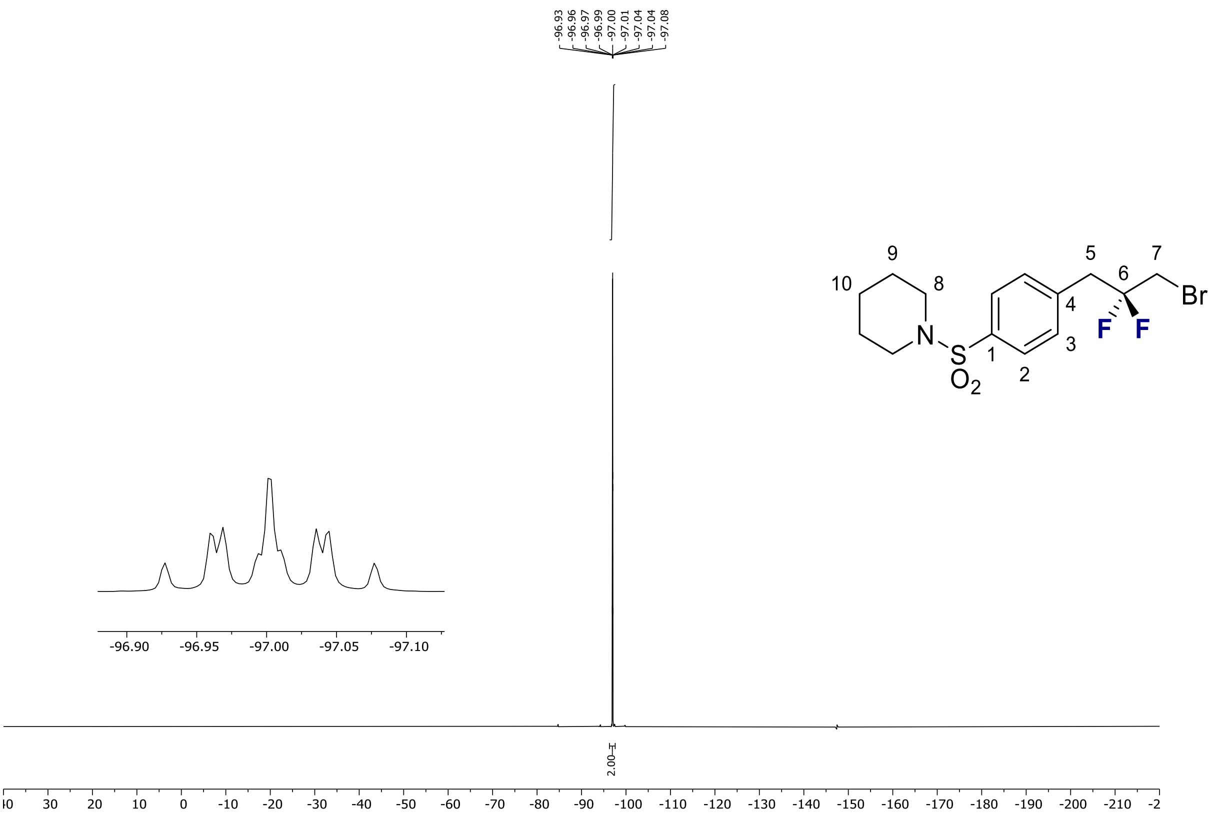

^{19}F{^{1}H} NMR (376 MHz, $CDCl_3$):

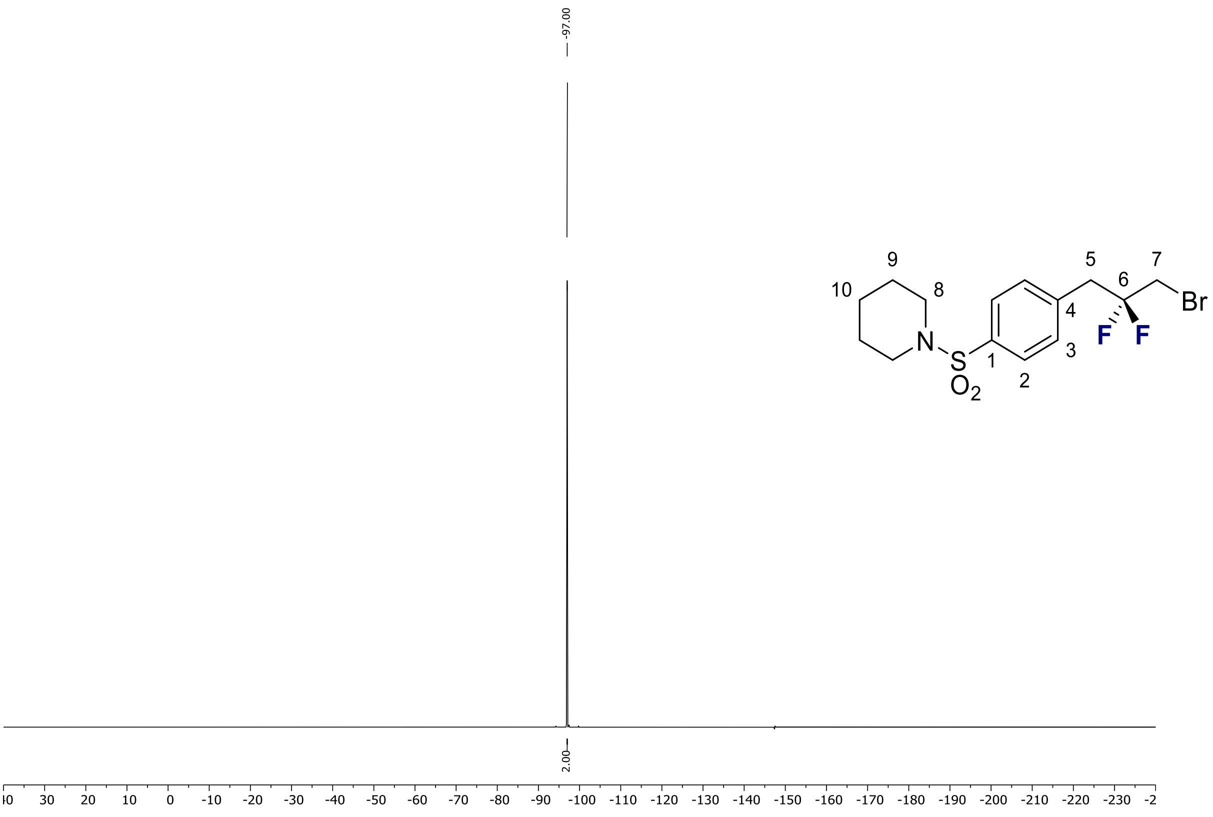

2-(4-(3-Bromo-2,2-difluoropropyl)phenyl)-1H-indene-1,3(2H)-dione (2n)

^{1}H NMR (500 MHz, $CDCl_3$):

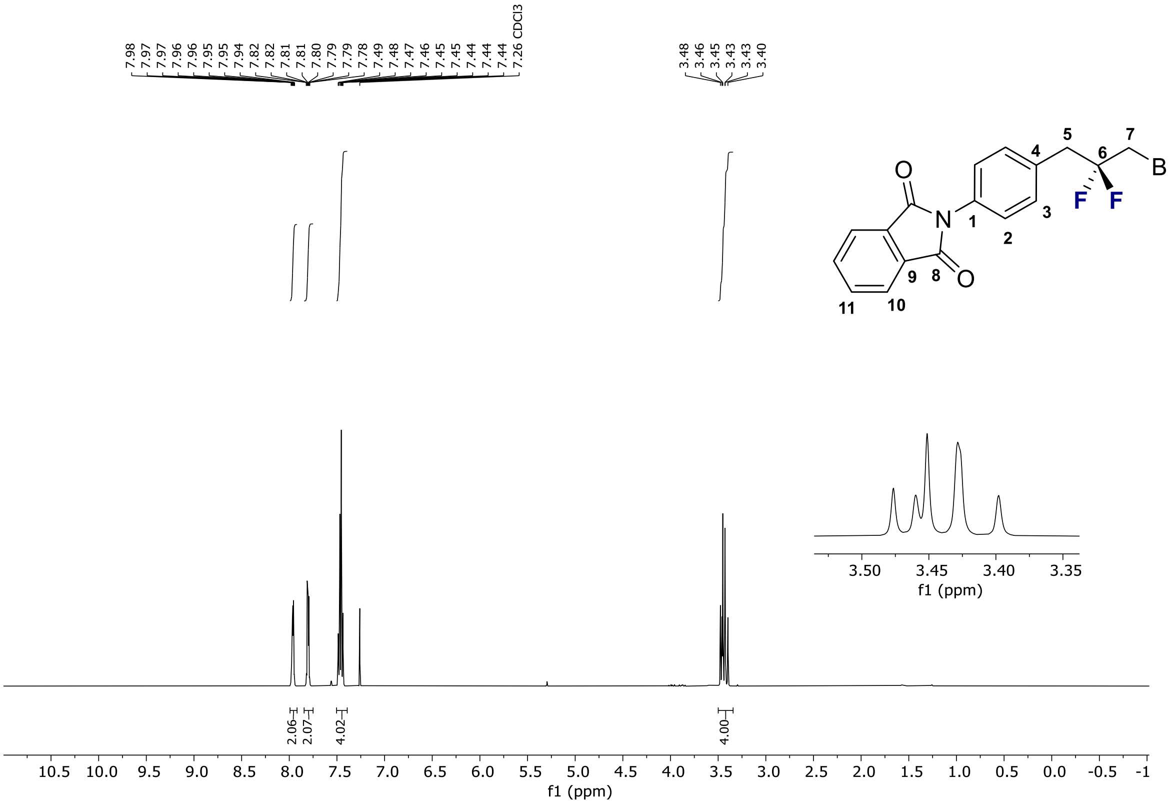

$^{13}C\{^{1}H\}$ NMR (126 MHz, $CDCl_3$):

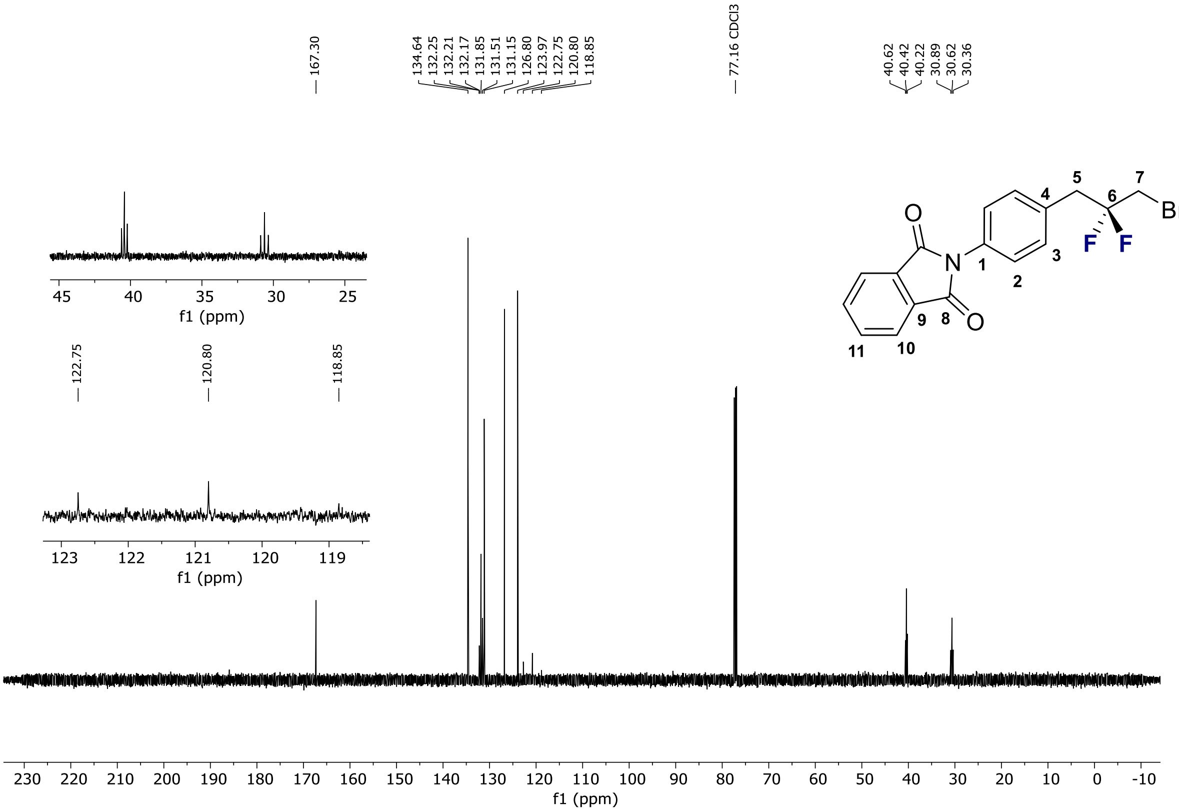

^{19}F NMR (376 MHz, $CDCl_3$):

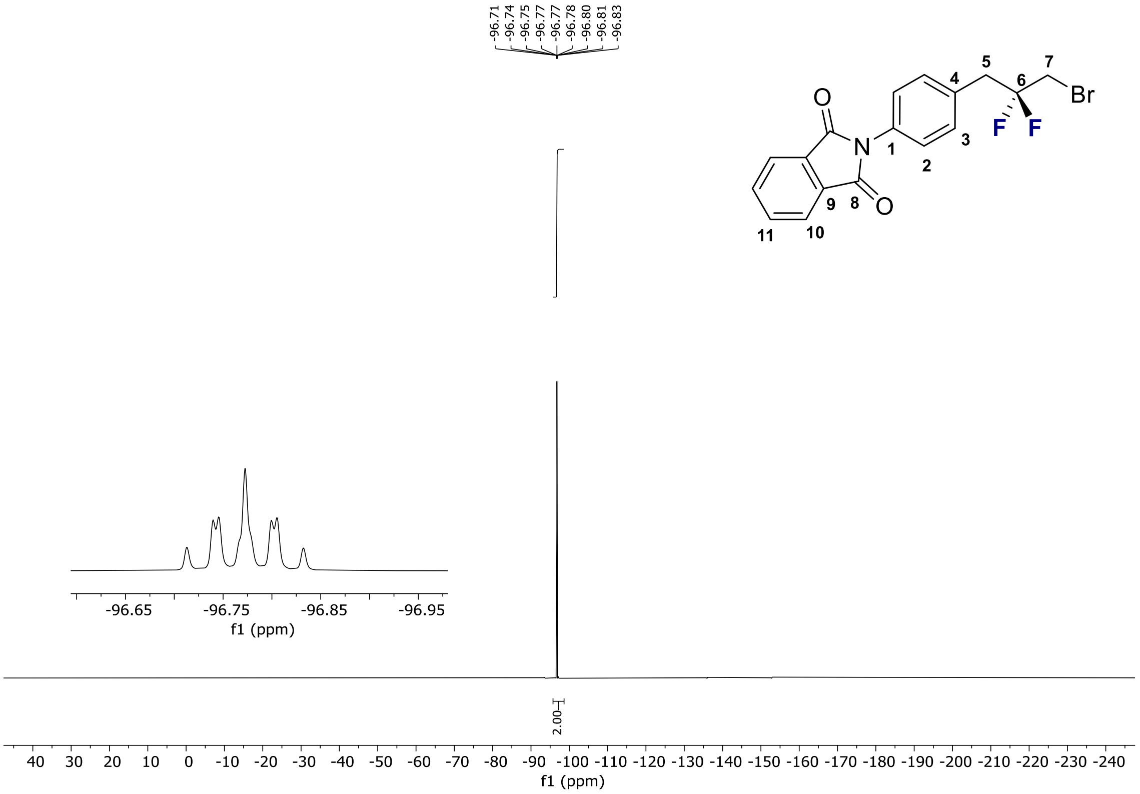

^{19}F{^{1}H} NMR (376 MHz, $CDCl_3$):

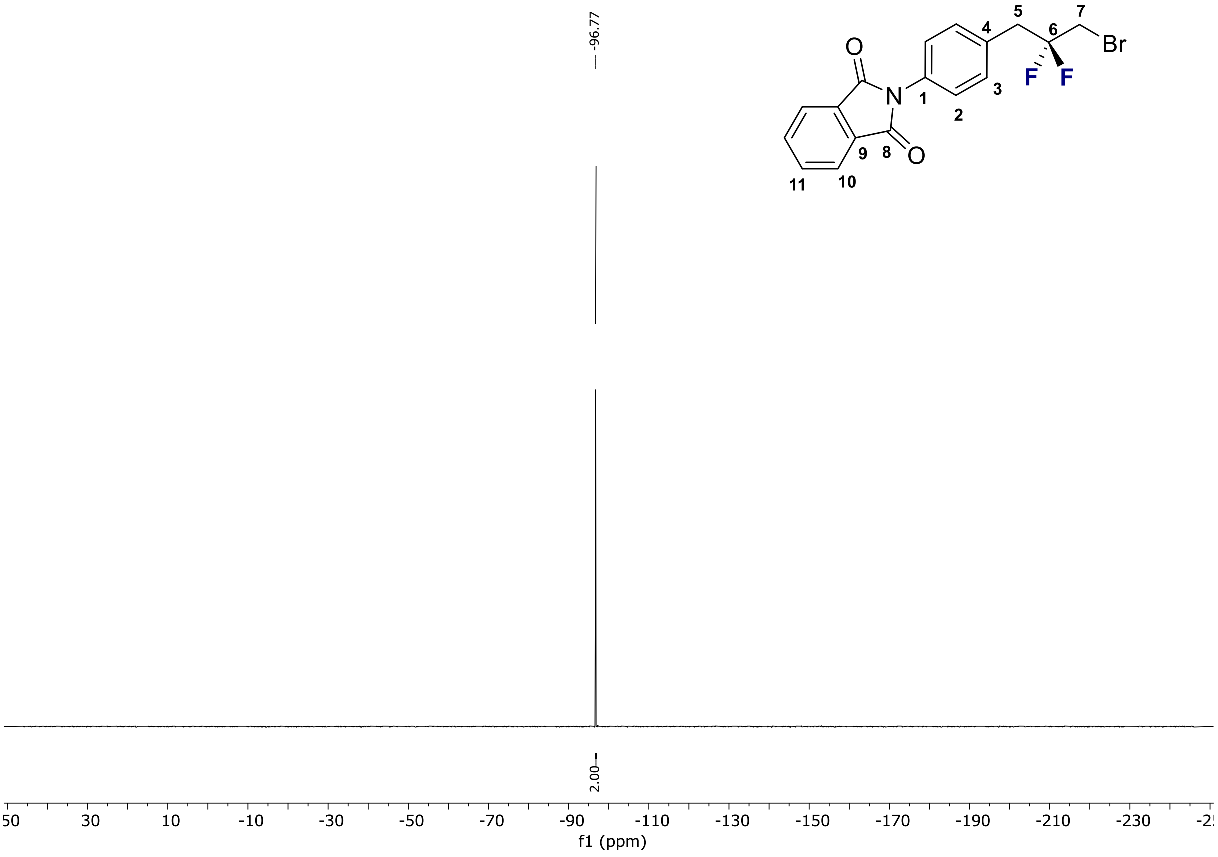

1-(3-Bromo-2,2-difluoropropyl)-4-(3,3,3-trifluoroprop-1-en-2-yl)benzene (4)

^{1}H NMR (599 MHz, $CDCl_3$):

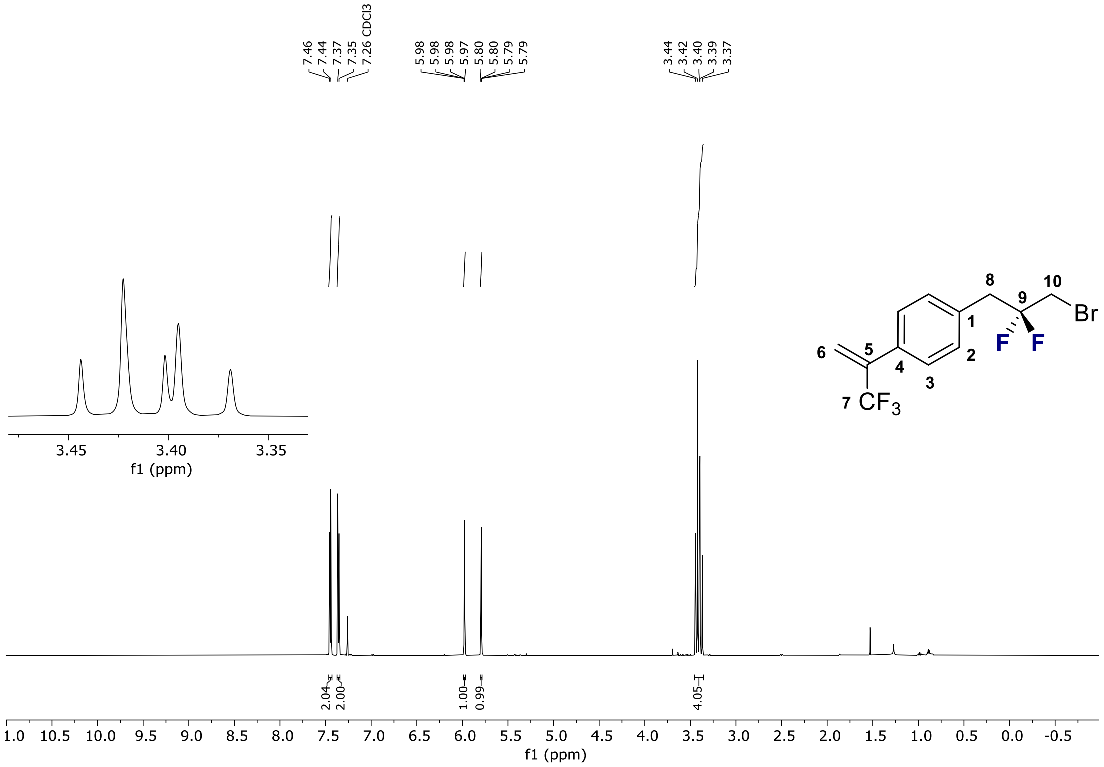

^{13}C{^{1}H} NMR (151 MHz, $CDCl_3$):

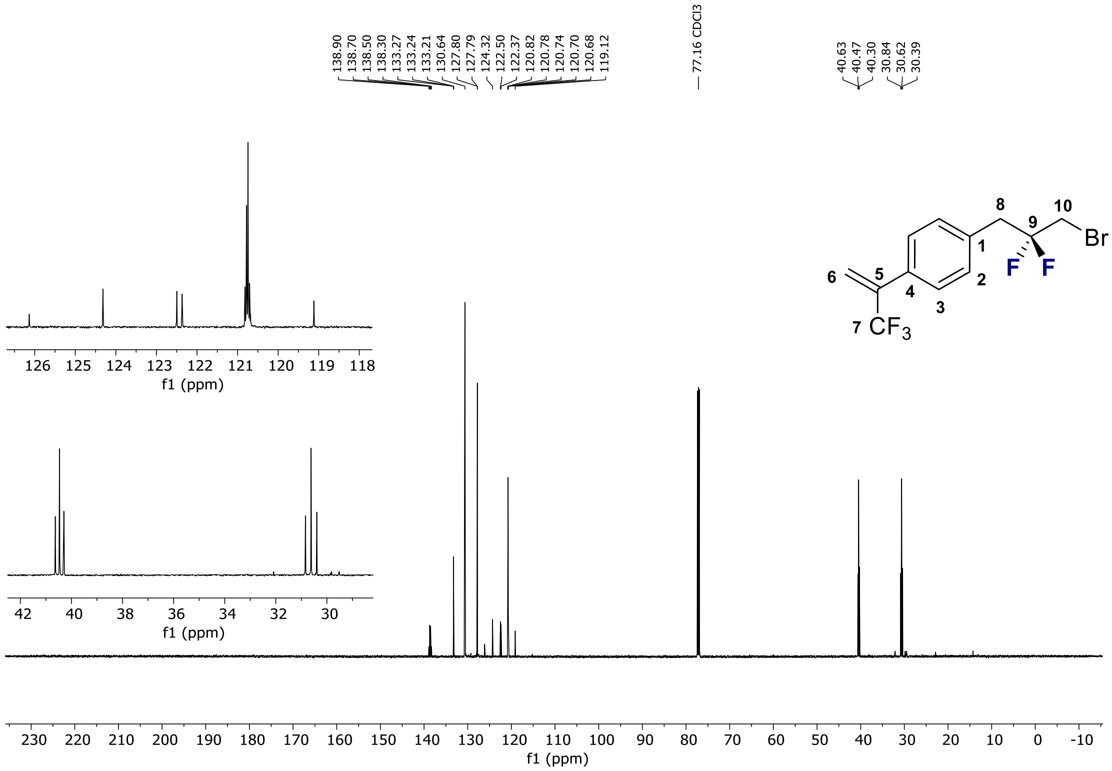

^{19}F NMR (376 MHz, $CDCl_3$):

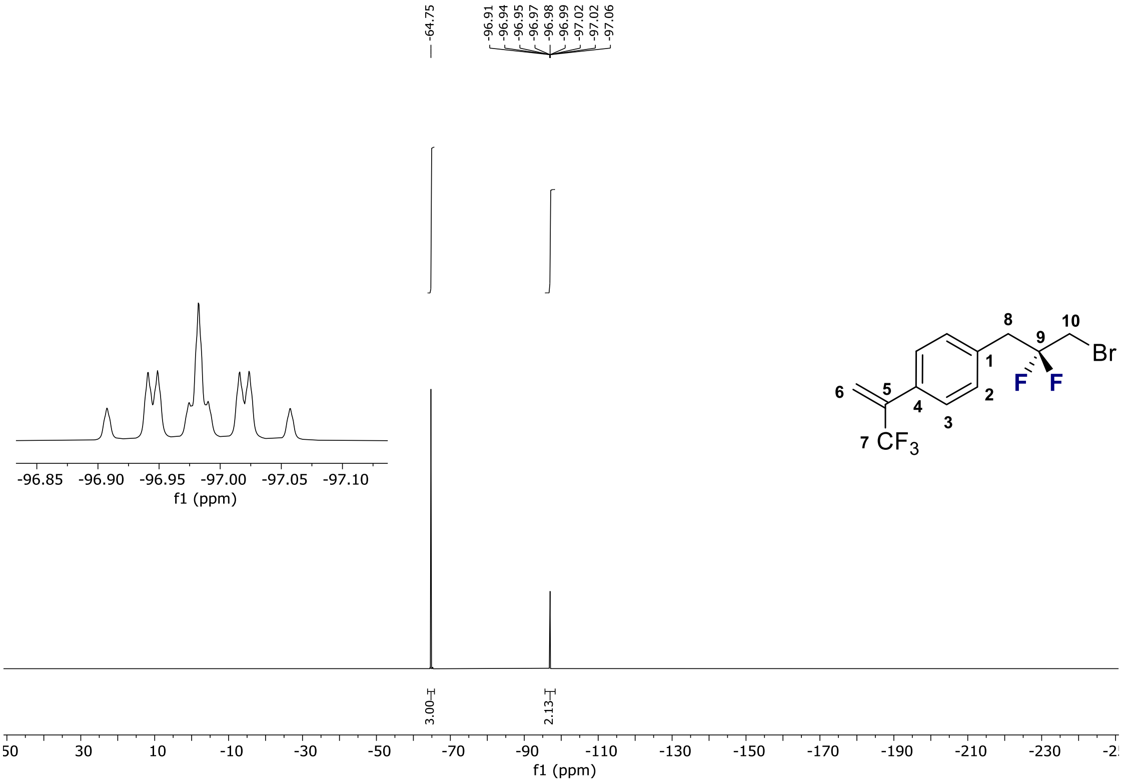

^{19}F{^{1}H} NMR (376 MHz, $CDCl_3$):

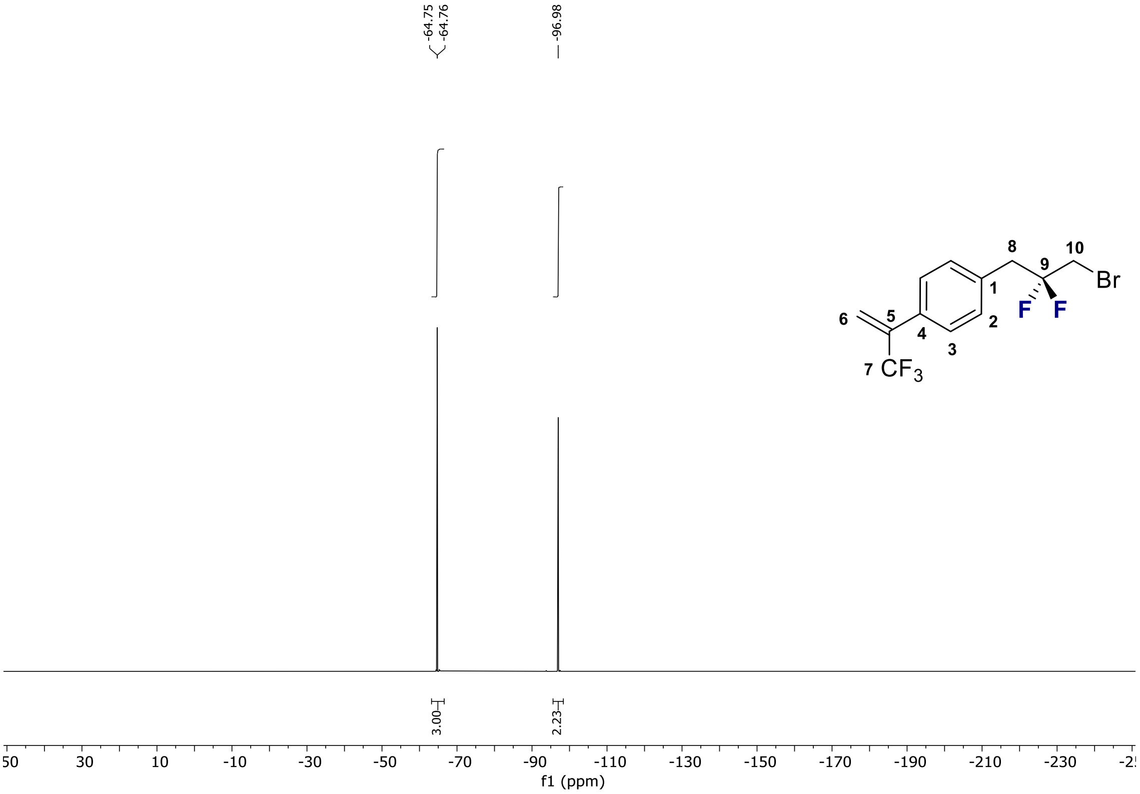

1-(3-Bromo-2,2-difluoropropyl)-4-(1,1,1,2,3-pentafluoropropan-2-yl)benzene (5)

^{1}H NMR (599 MHz, $CDCl_3$):

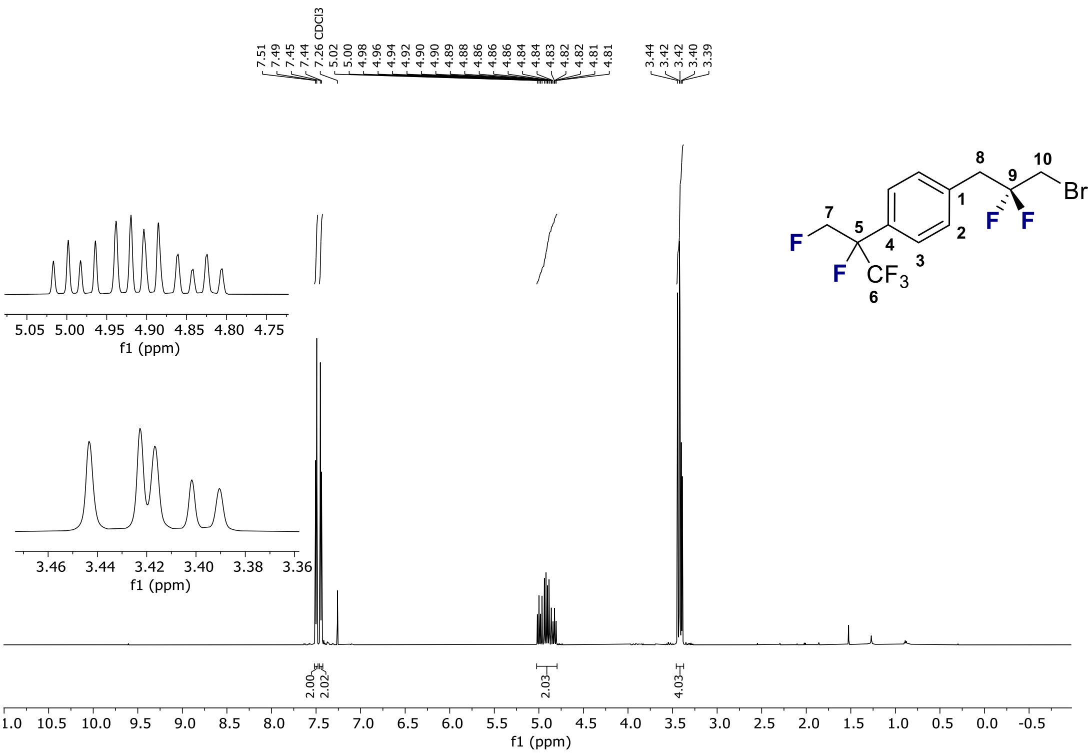

^{13}C{^{1}H} NMR (151 MHz, $CDCl_3$):

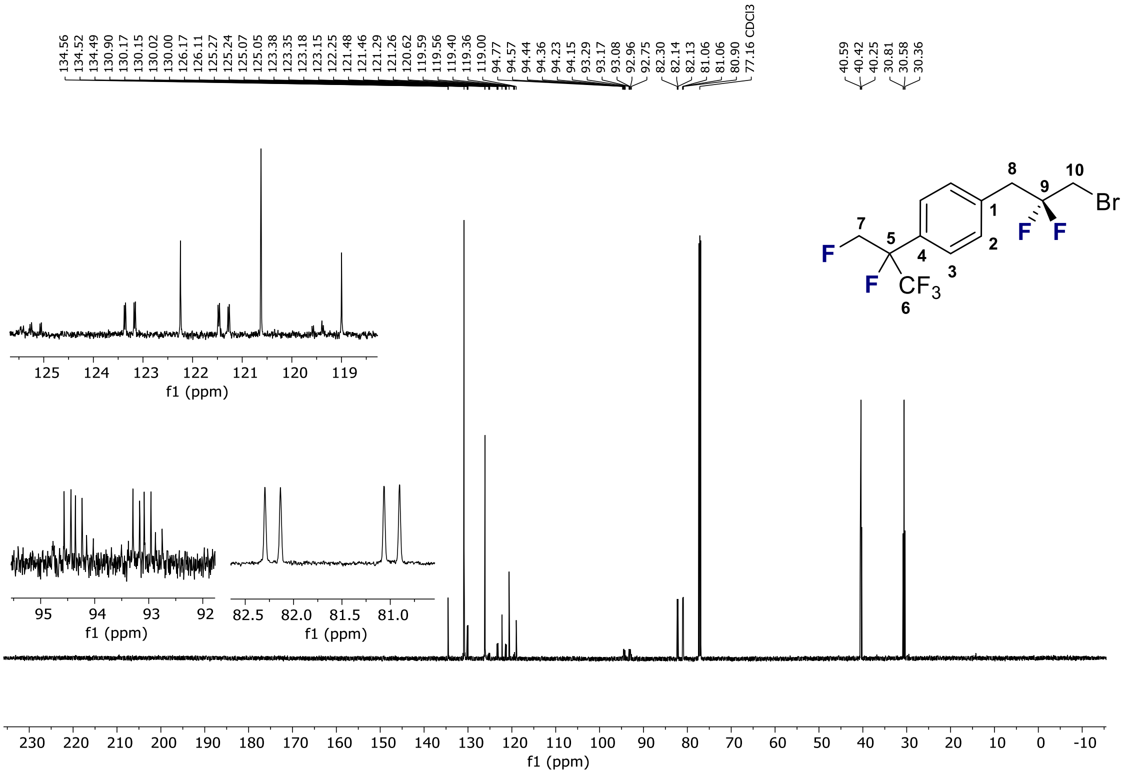

^{19}F NMR (376 MHz, $CDCl_3$):

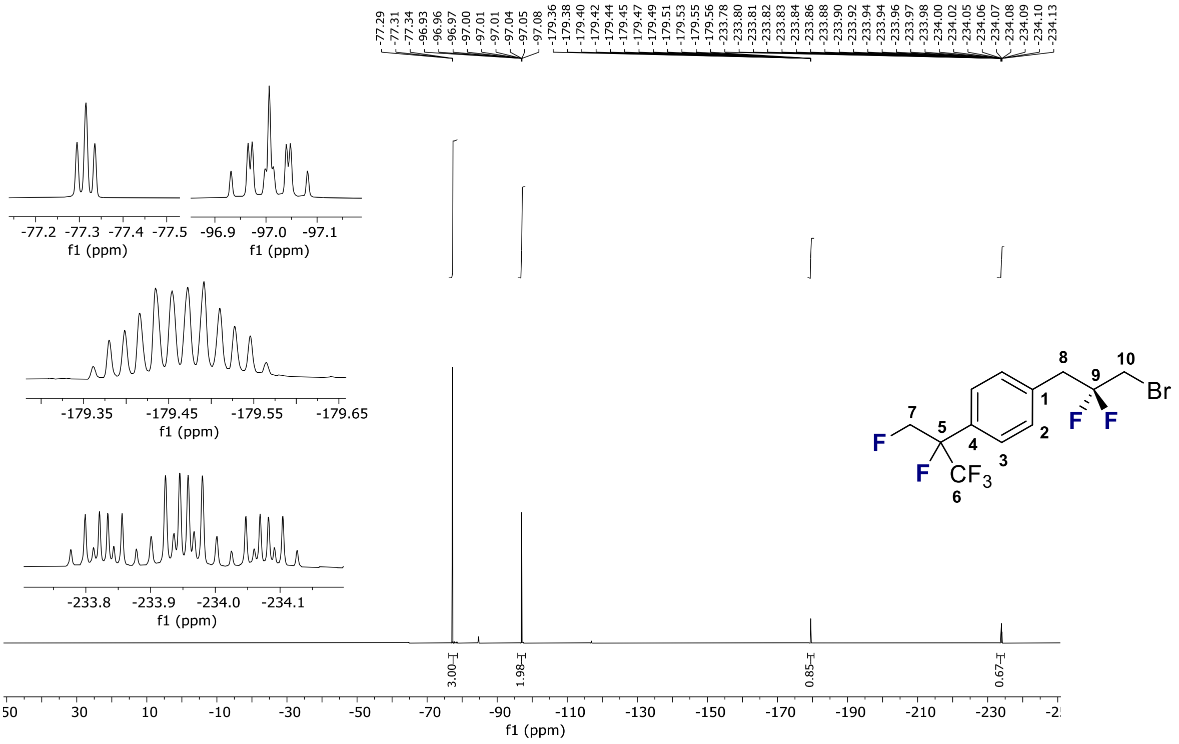

^{19}F{^{1}H} NMR (376 MHz, $CDCl_3$):

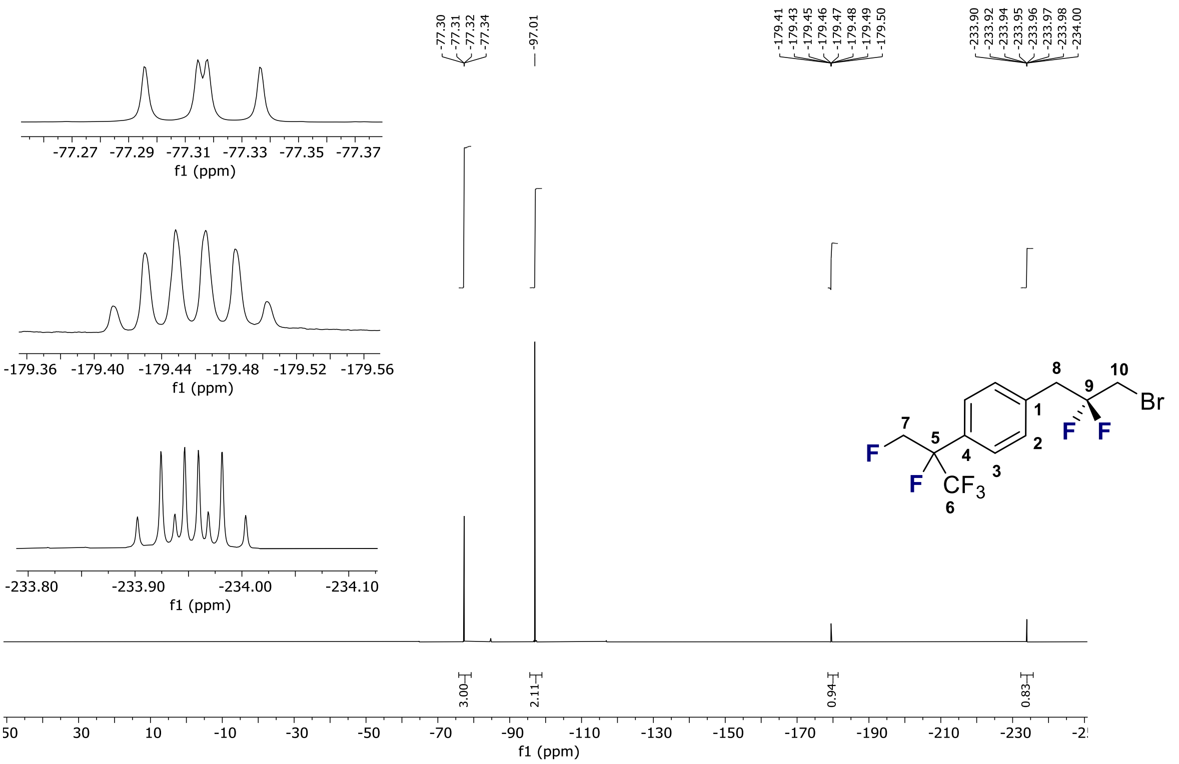

1-(3-Bromo-2,2-difluoropropyl)-4-(2,2,3,3,3-pentafluoropropyl)benzene *gem*-(5)

^{1}H NMR (599 MHz, $CDCl_3$):

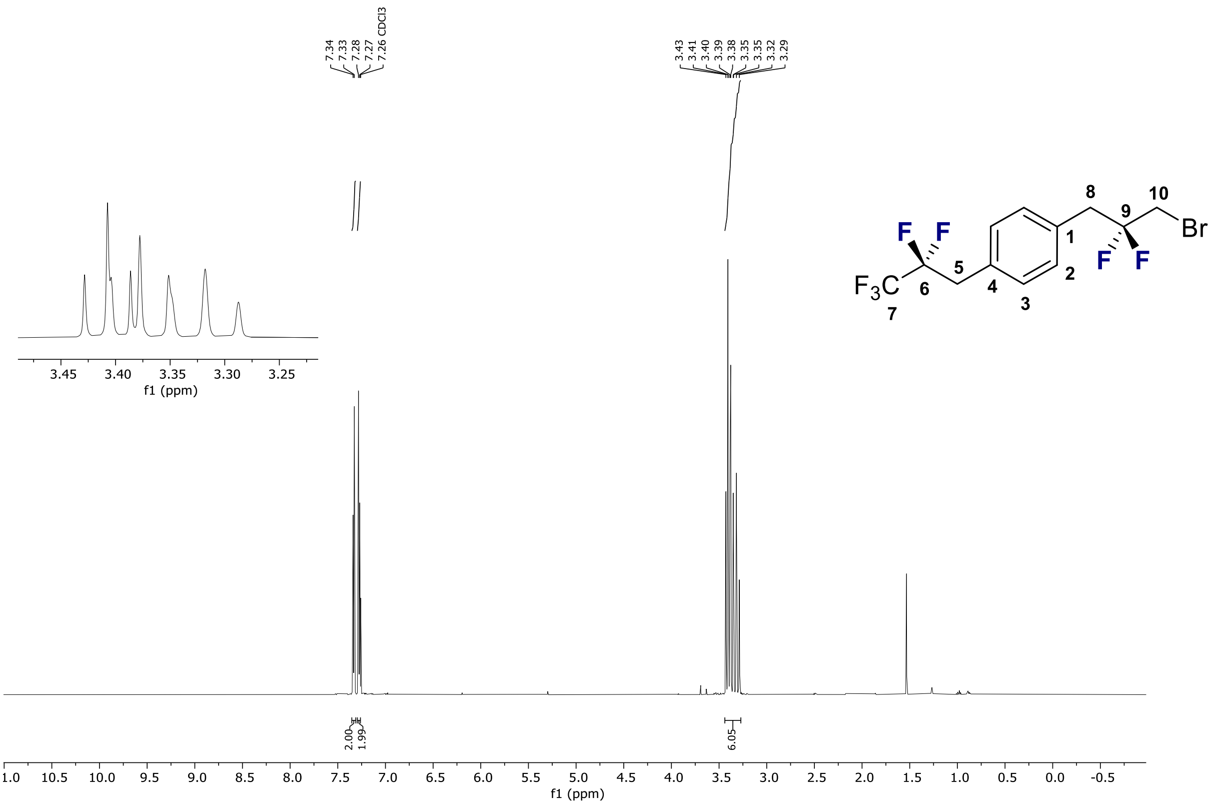

$^{13}C\{^1H\}$ NMR (151 MHz, $CDCl_3$):

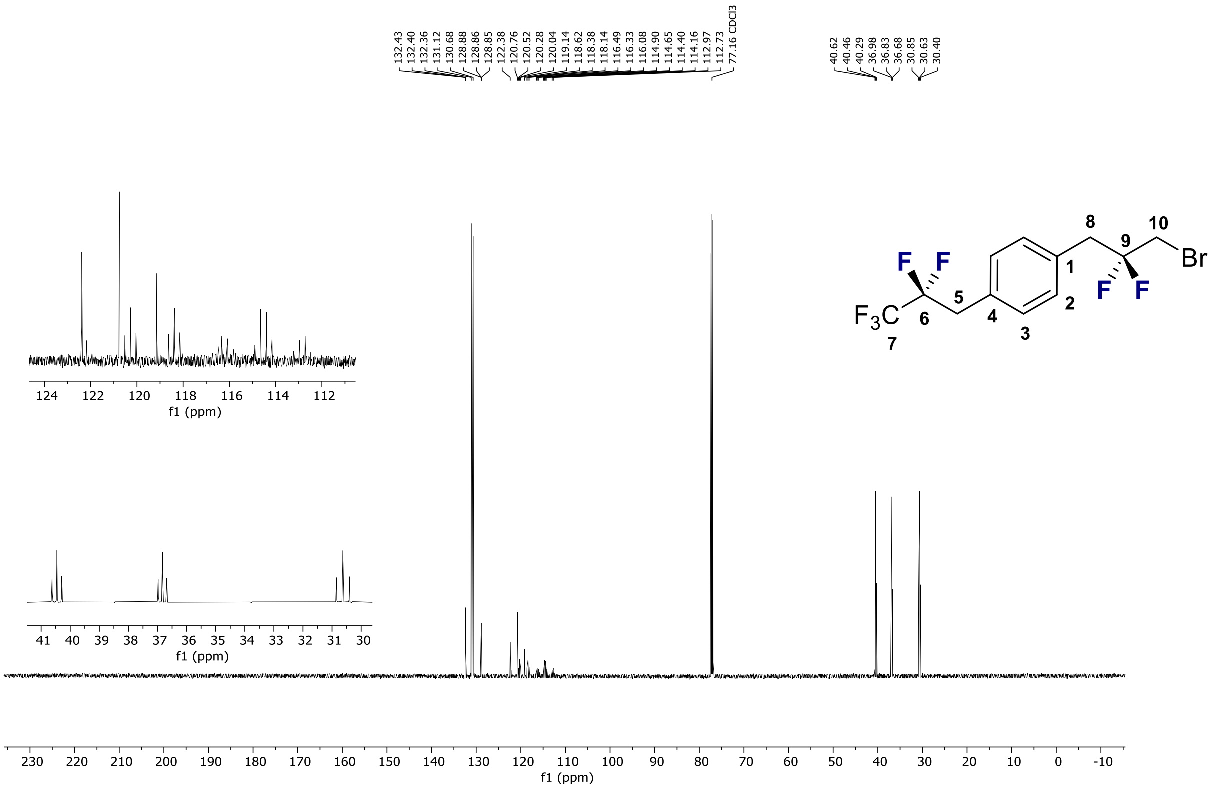

^{19}F NMR (376 MHz, $CDCl_3$):

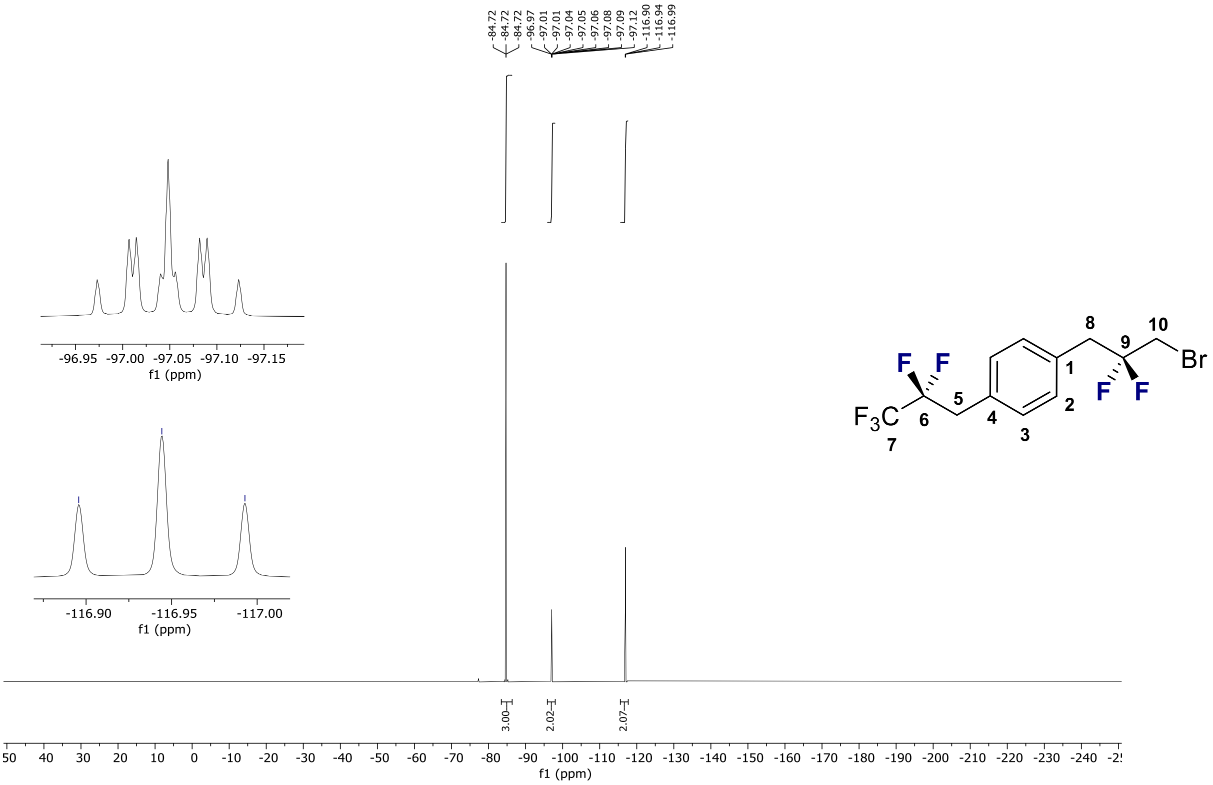

^{19}F{^{1}H} NMR (376 MHz, $CDCl_3$):

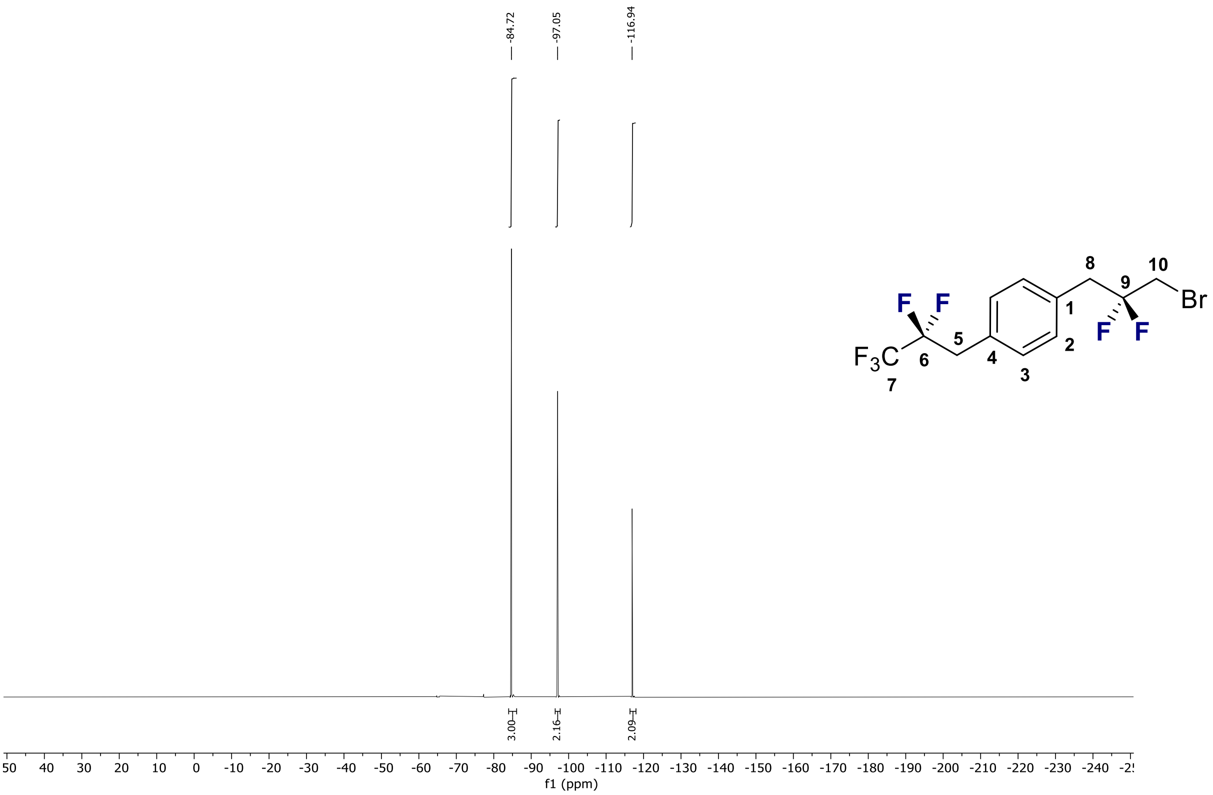

Methyl (*E*)-3-(4-(3-bromo-2,2-difluoropropyl)phenyl)acrylate (7)

^{1}H NMR (599 MHz, $CDCl_3$):

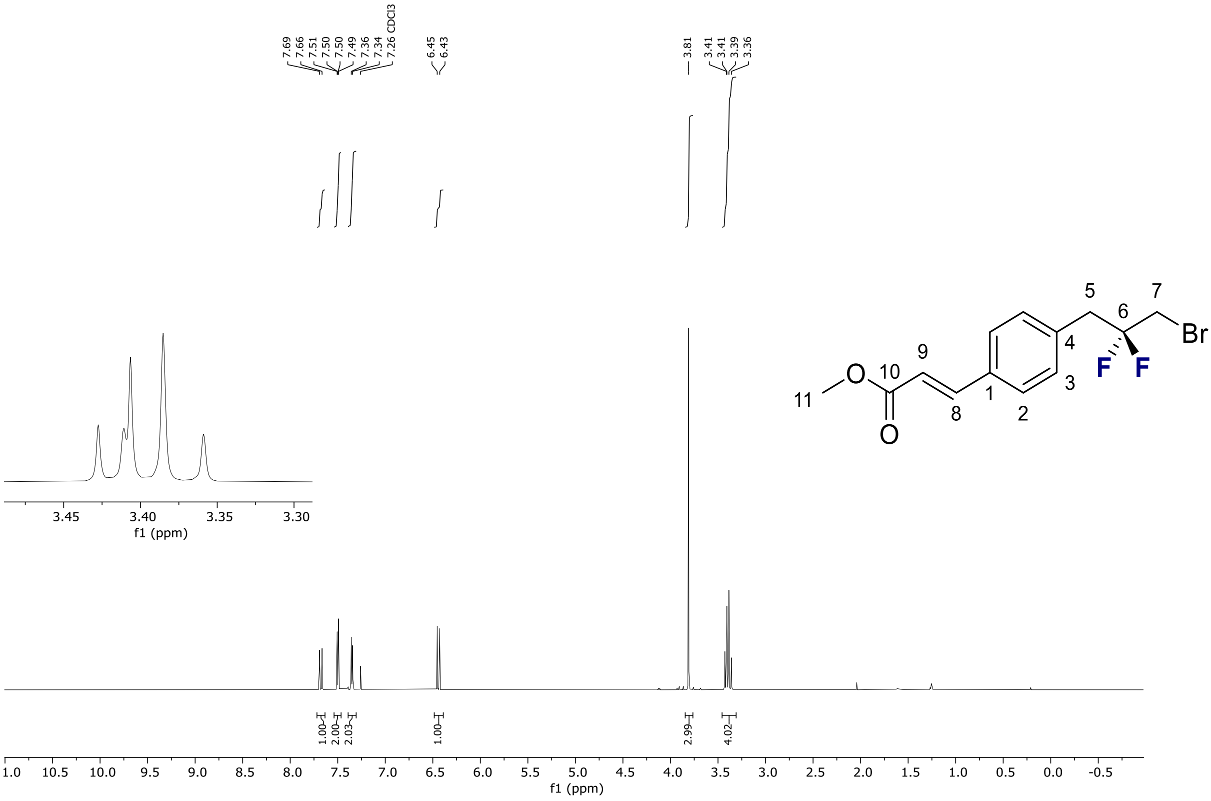

$^{13}C\{^{1}H\}$ NMR (151 MHz, $CDCl_3$):

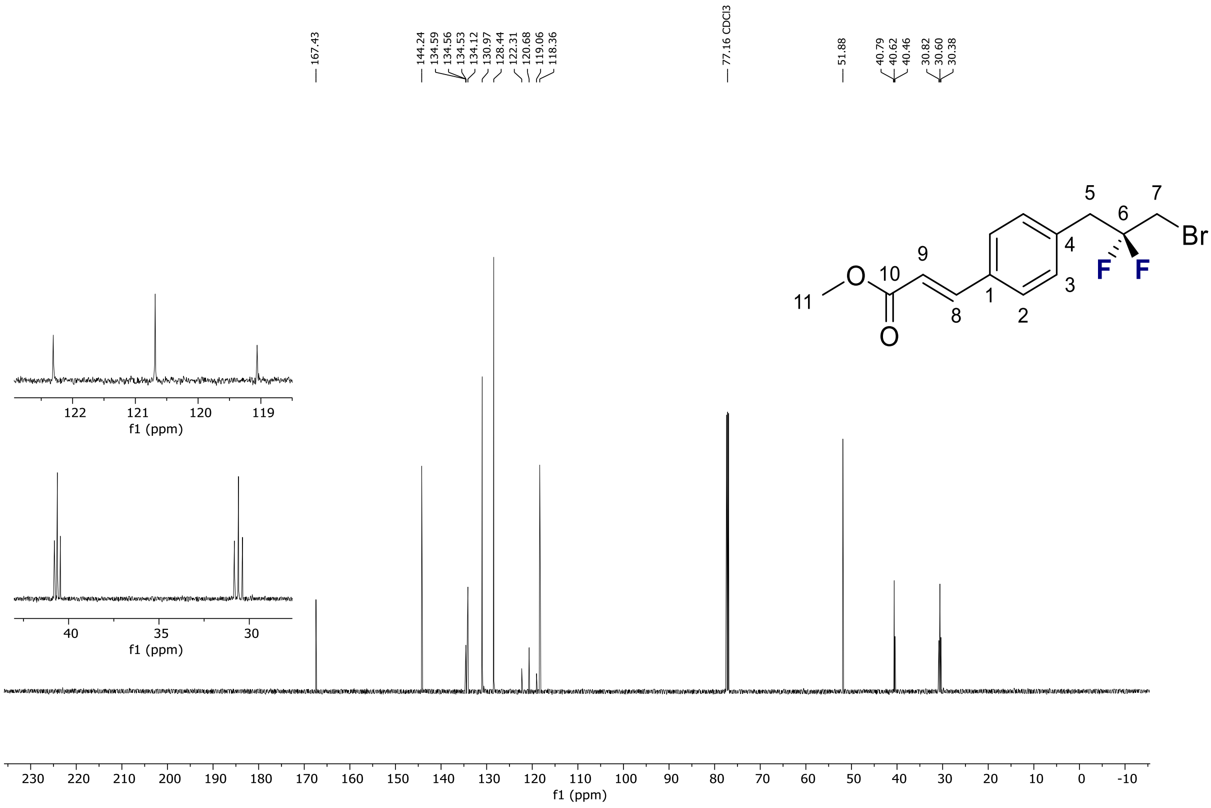

Methyl 2-(4-(3-bromo-2,2-difluoropropyl)phenyl)-3,3-difluoropropanoate (8)

^{1}H NMR (500 MHz, $CDCl_3$):

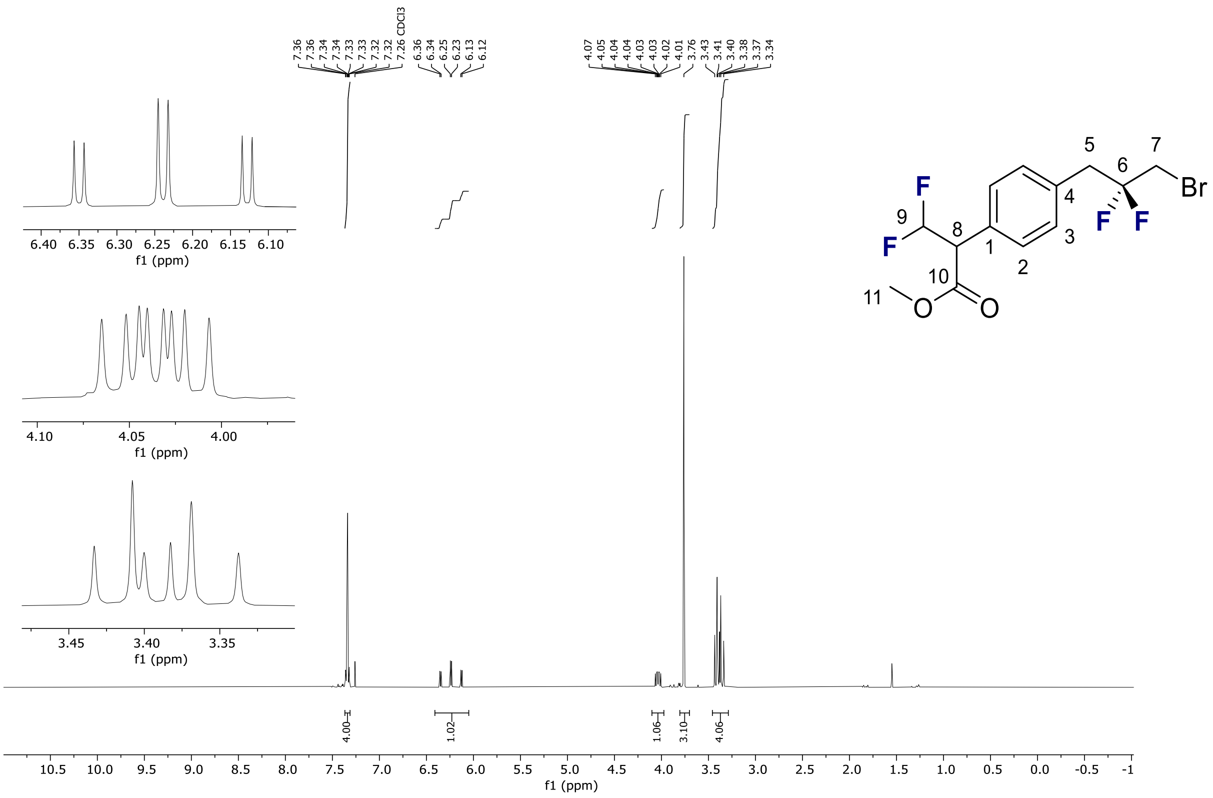

^{13}C{^{1}H} NMR (126 MHz, $CDCl_3$):

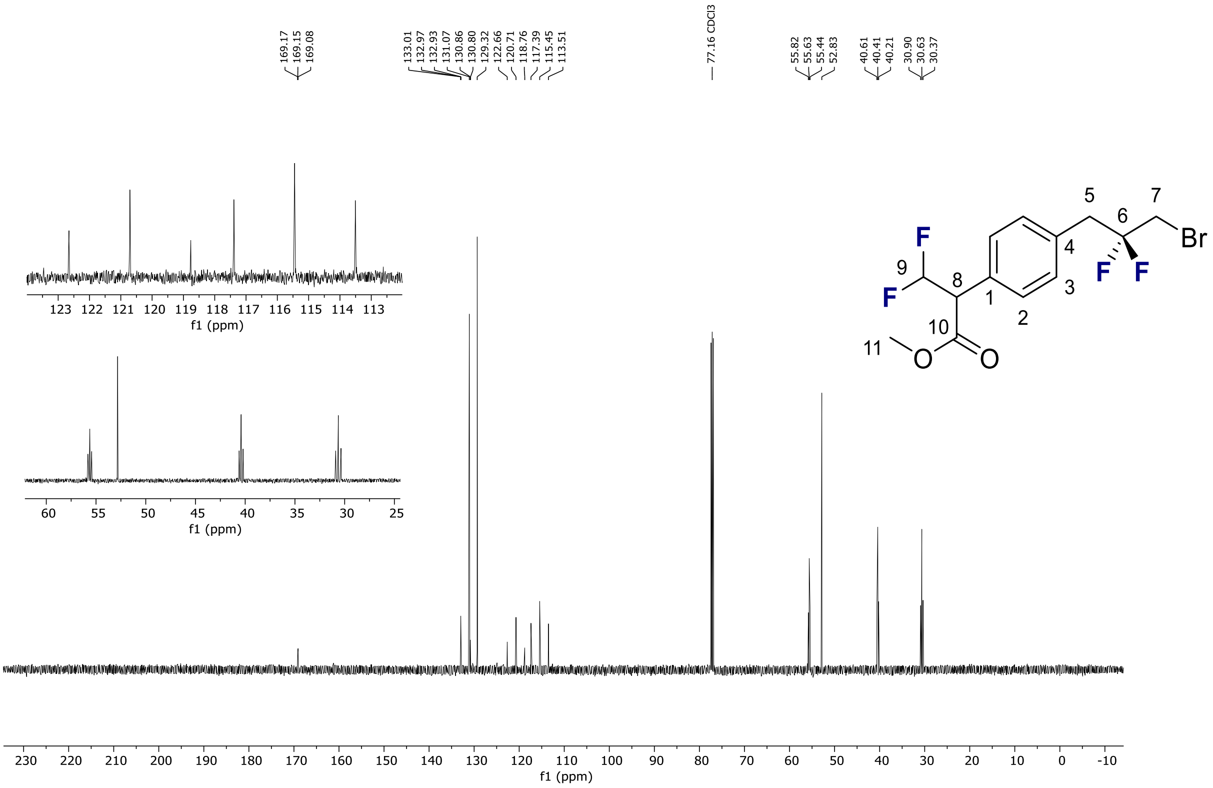

19F NMR (470 MHz, $CDCl_3$):

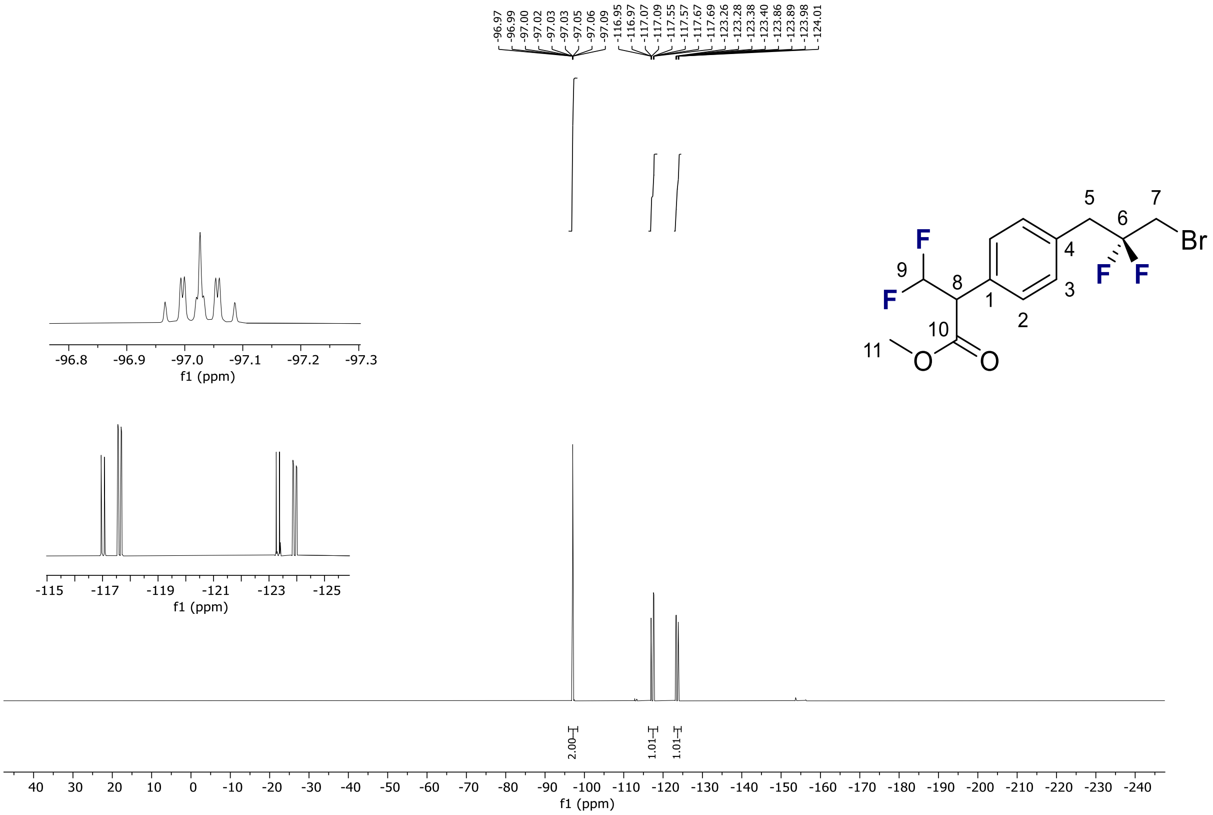

19F{1H} NMR (470 MHz, $CDCl_3$):

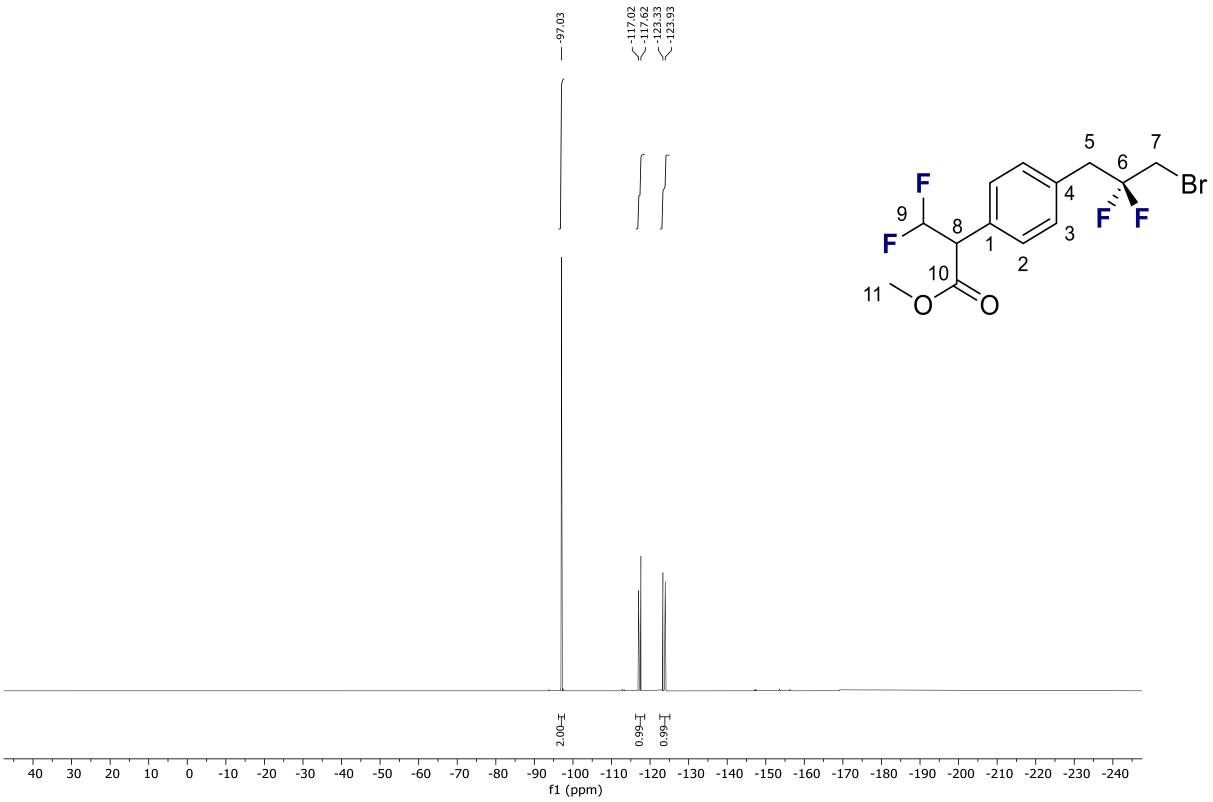

1,4-*Bis*(3-bromo-2,2-difluoropropyl)benzene (10)

^{1}H NMR (599 MHz, $CDCl_3$):

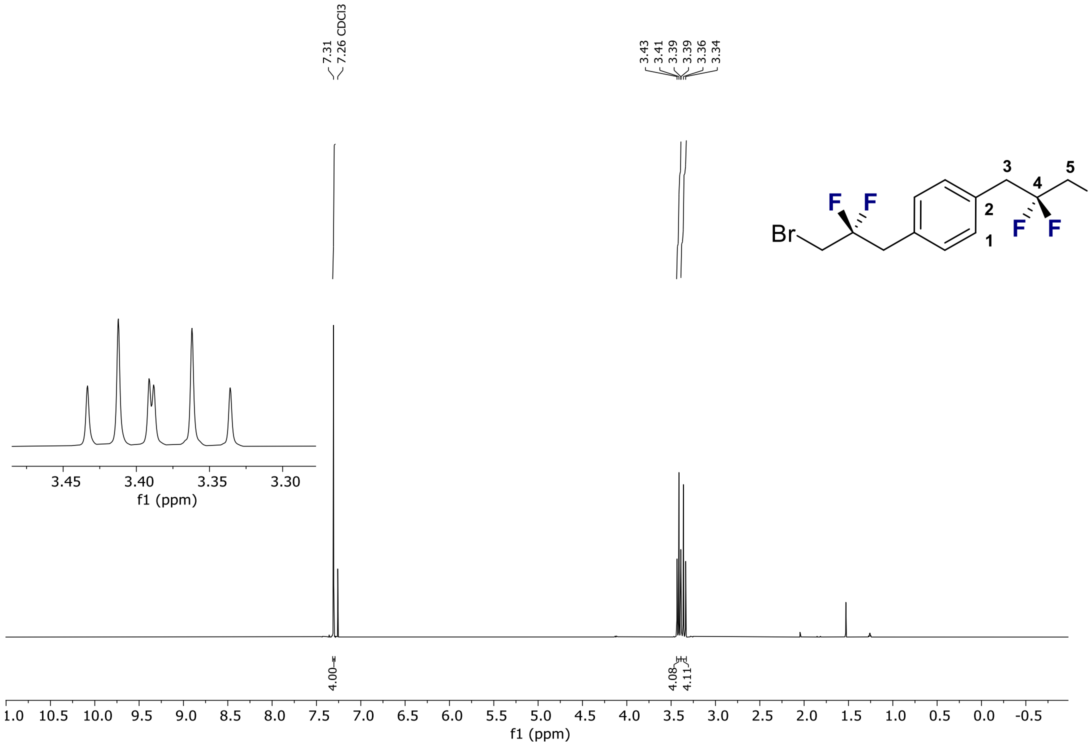

^{13}C{^{1}H} NMR (151 MHz, $CDCl_3$):

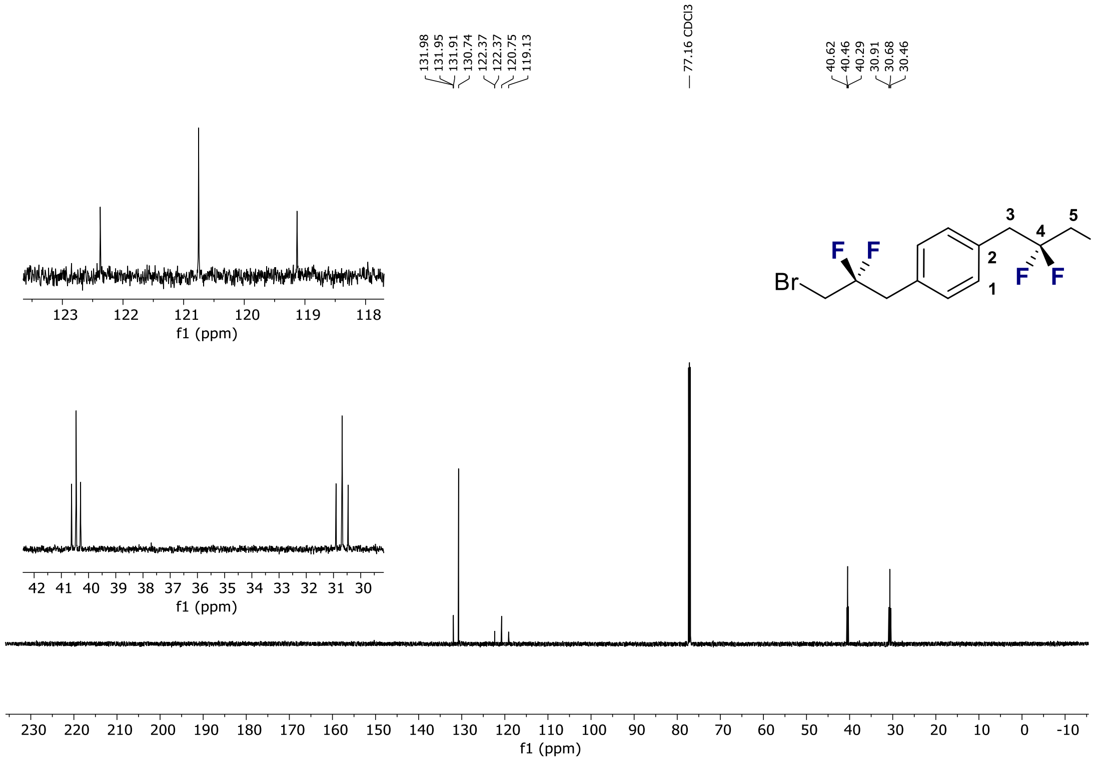

^{19}F NMR (376 MHz, $CDCl_3$):

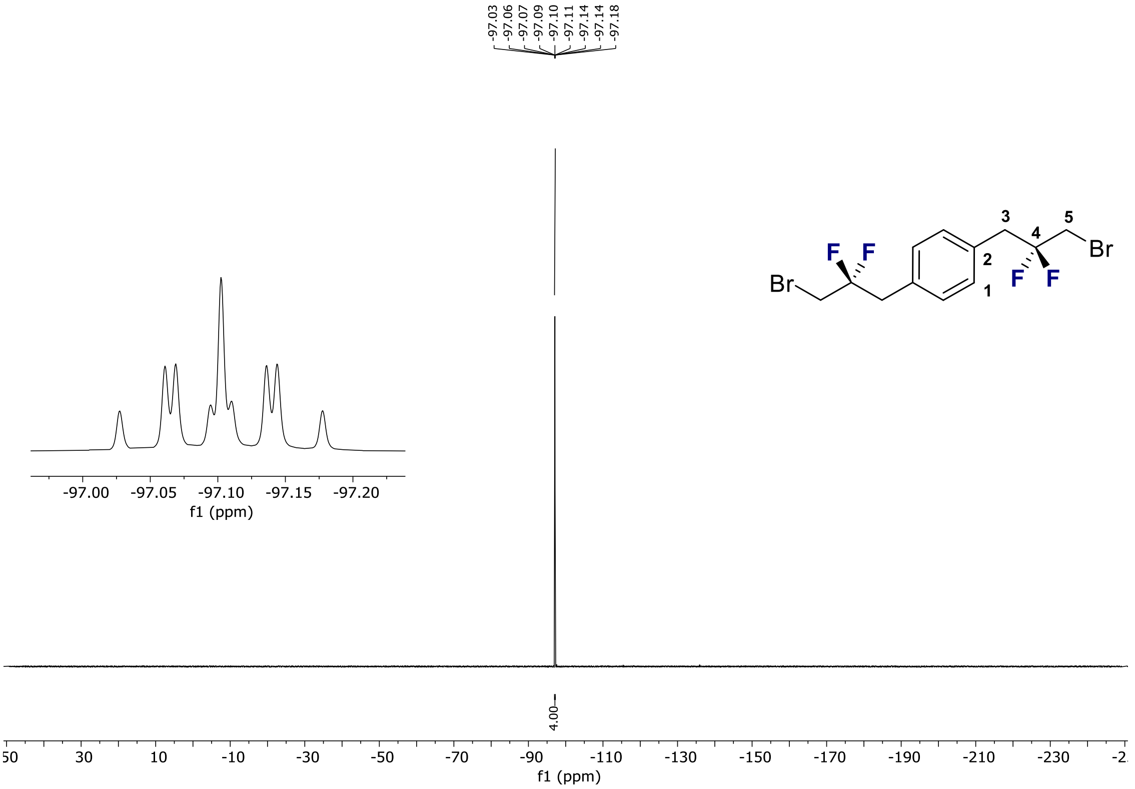

^{19}F{^{1}H} NMR (376 MHz, $CDCl_3$):

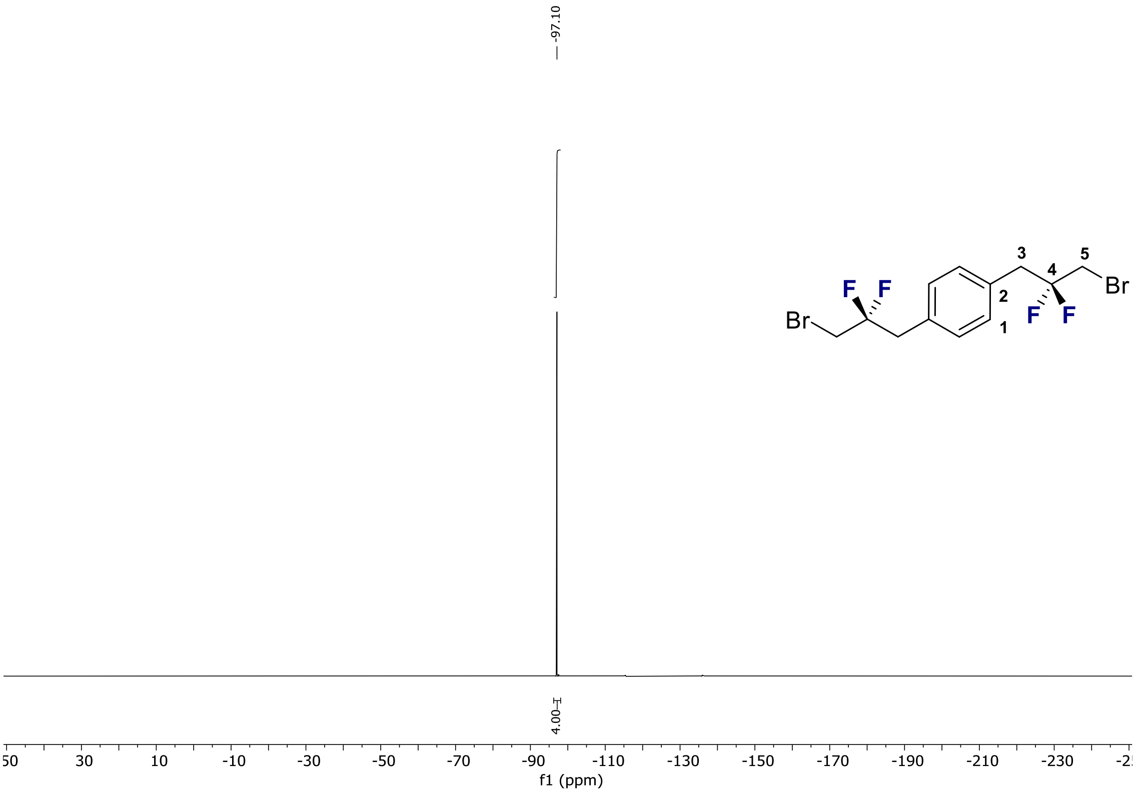

1,3-*Bis*(3-bromo-2,2-difluoropropyl)benzene (12)

^{1}H NMR (599 MHz, $CDCl_3$):

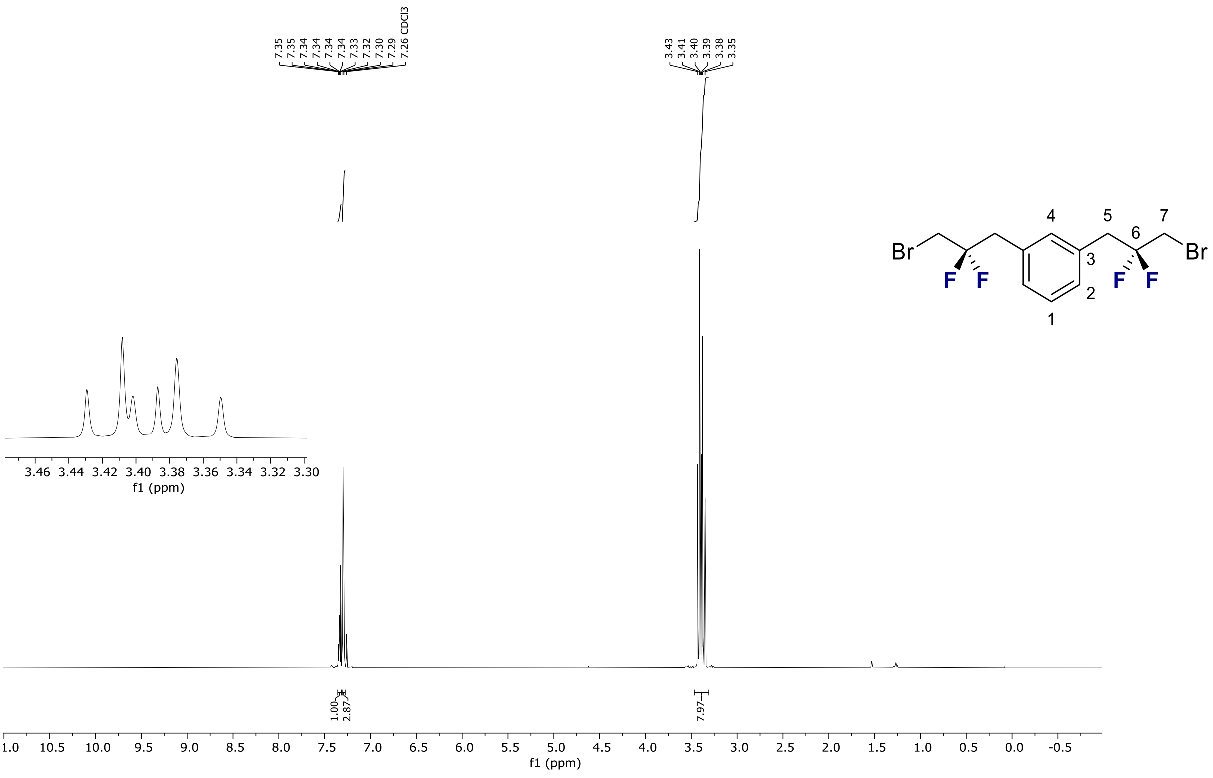

$^{13}C\{^{1}H\}$ NMR (151 MHz, $CDCl_3$):

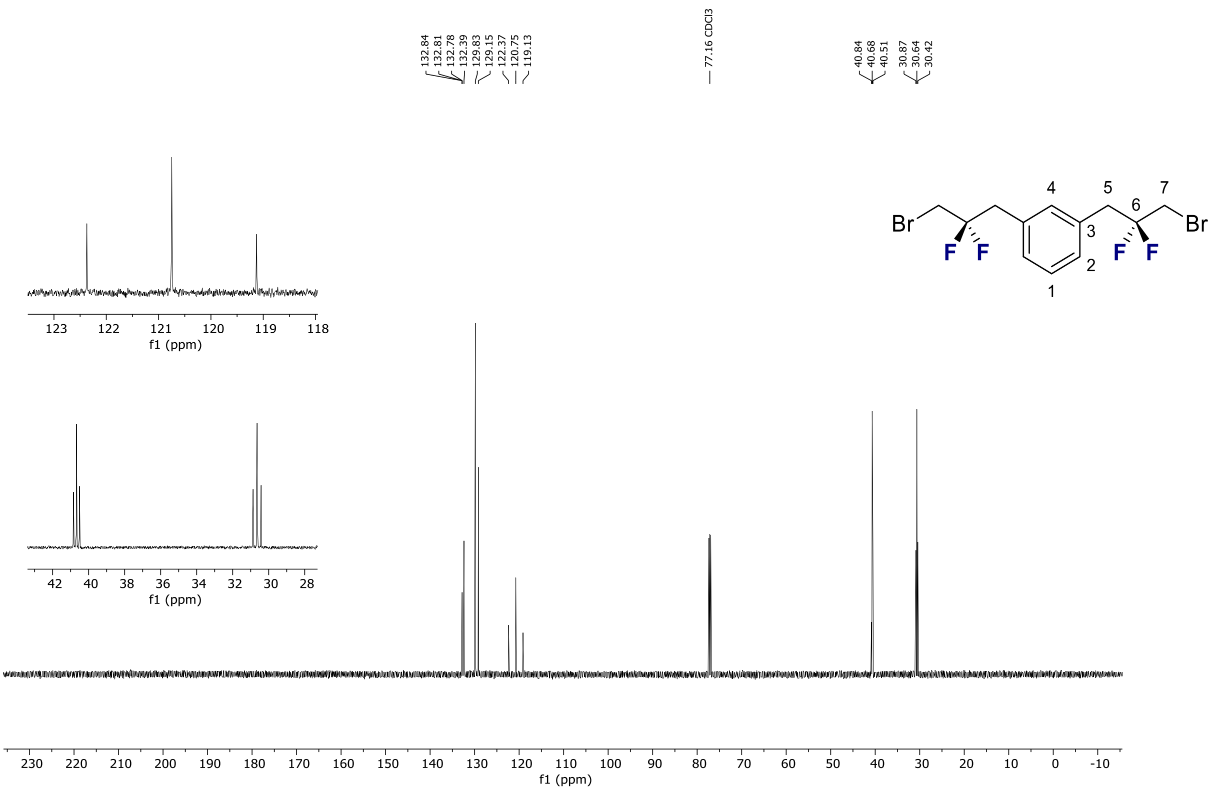

^{19}F NMR (564 MHz, $CDCl_3$):

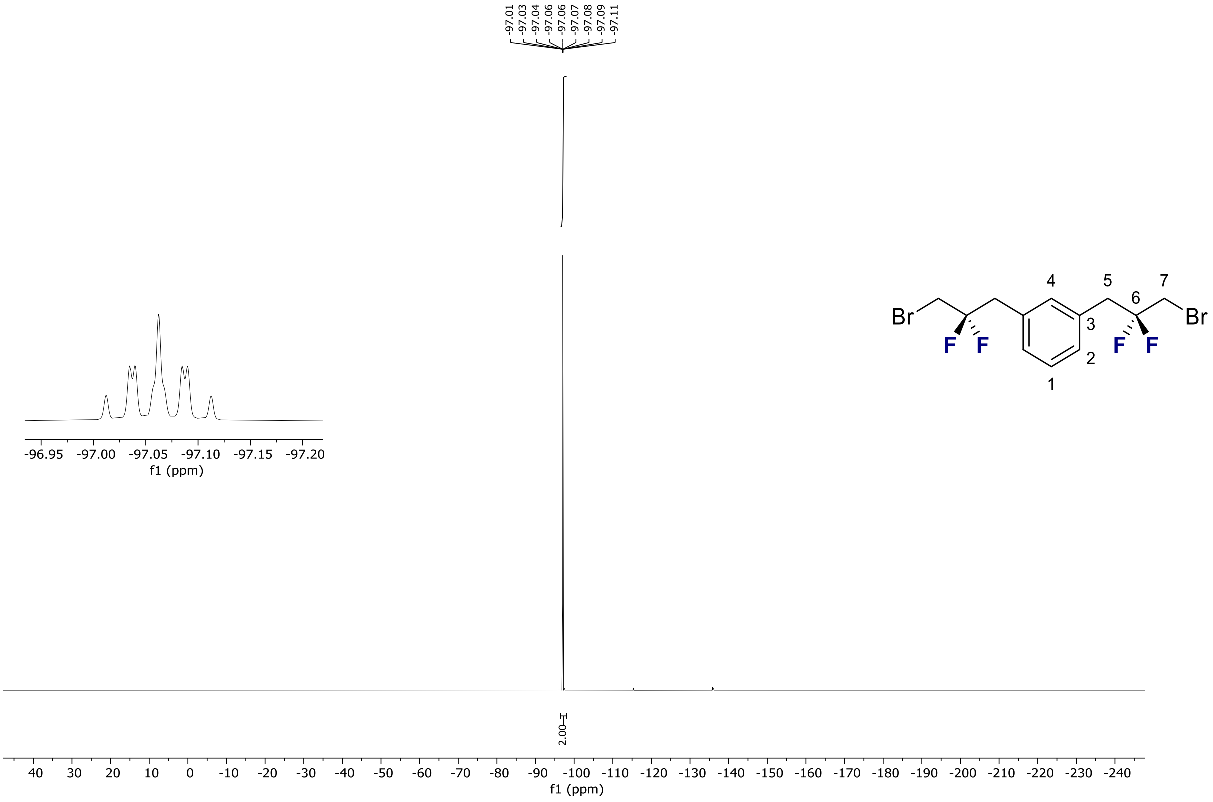

^{19}F{^{1}H} NMR (564 MHz, $CDCl_3$):

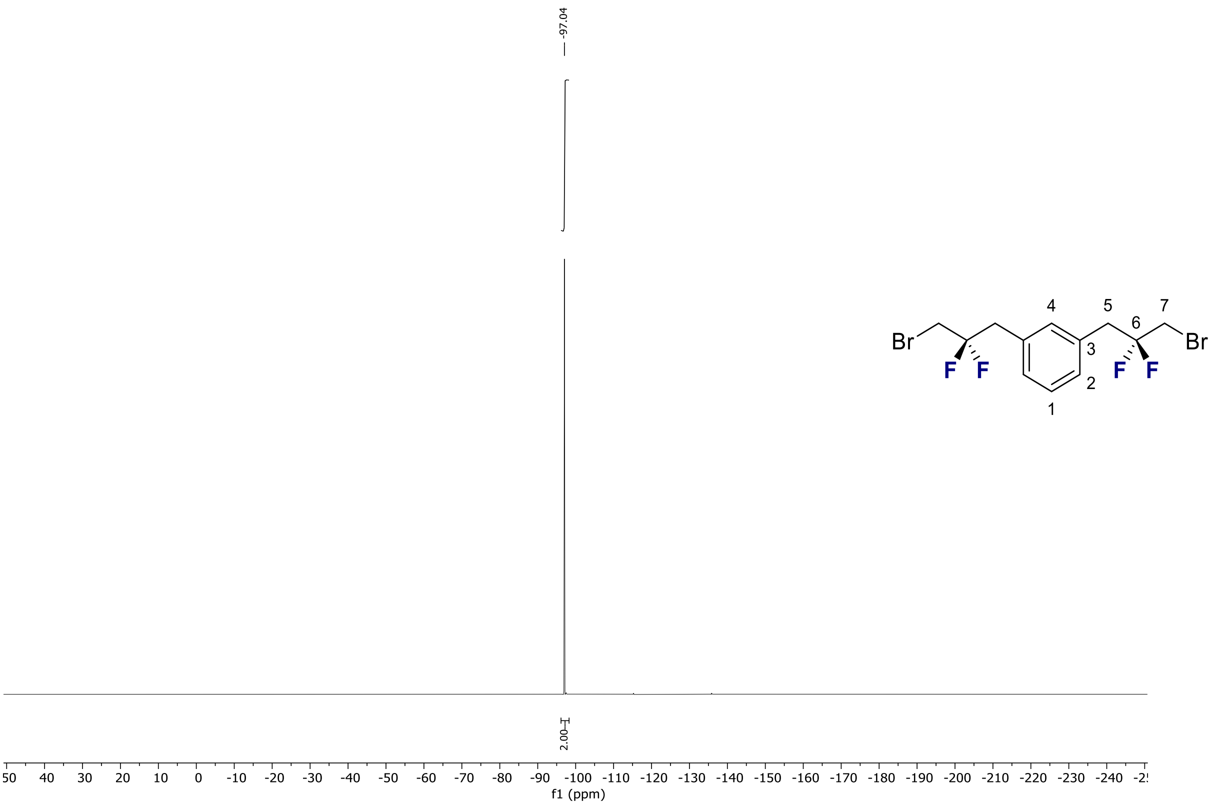

Ethyl 4-bromo-3,3-difluoro-2-phenylbutanoate (15)

^{1}H NMR (599 MHz, $CDCl_3$):

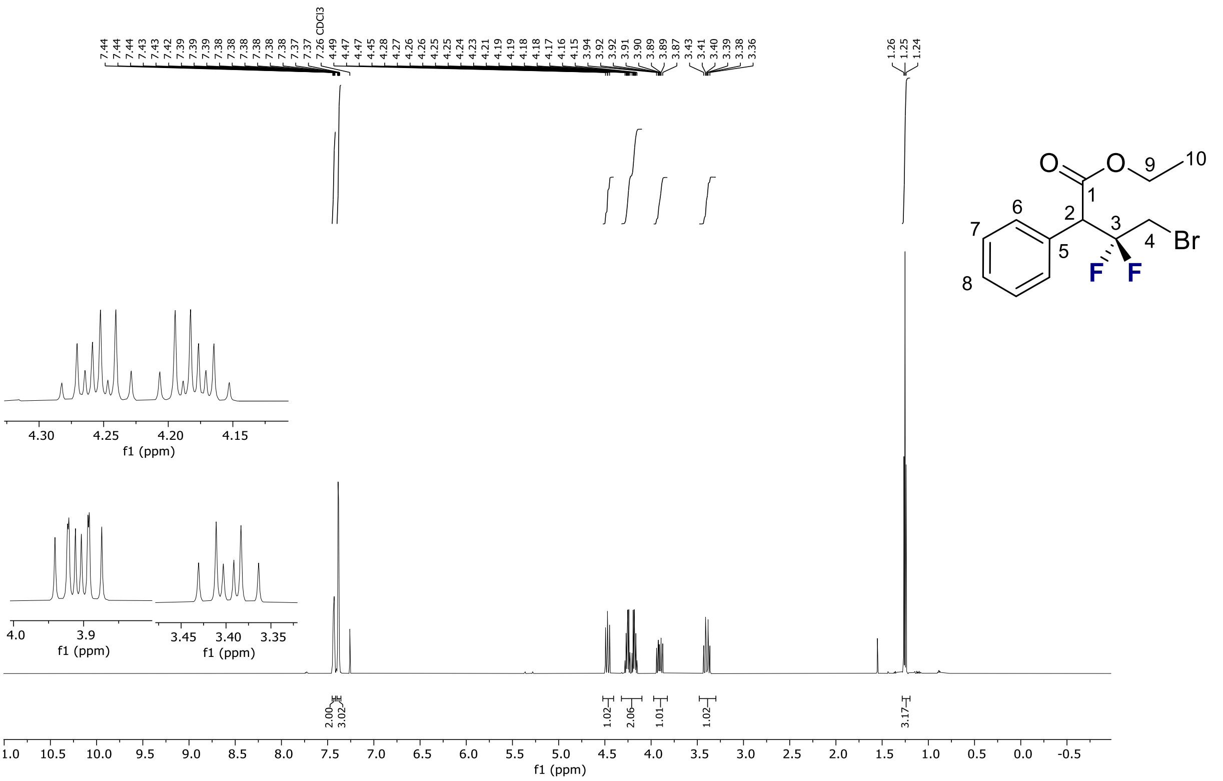

^{13}C NMR (151 MHz, $CDCl_3$):

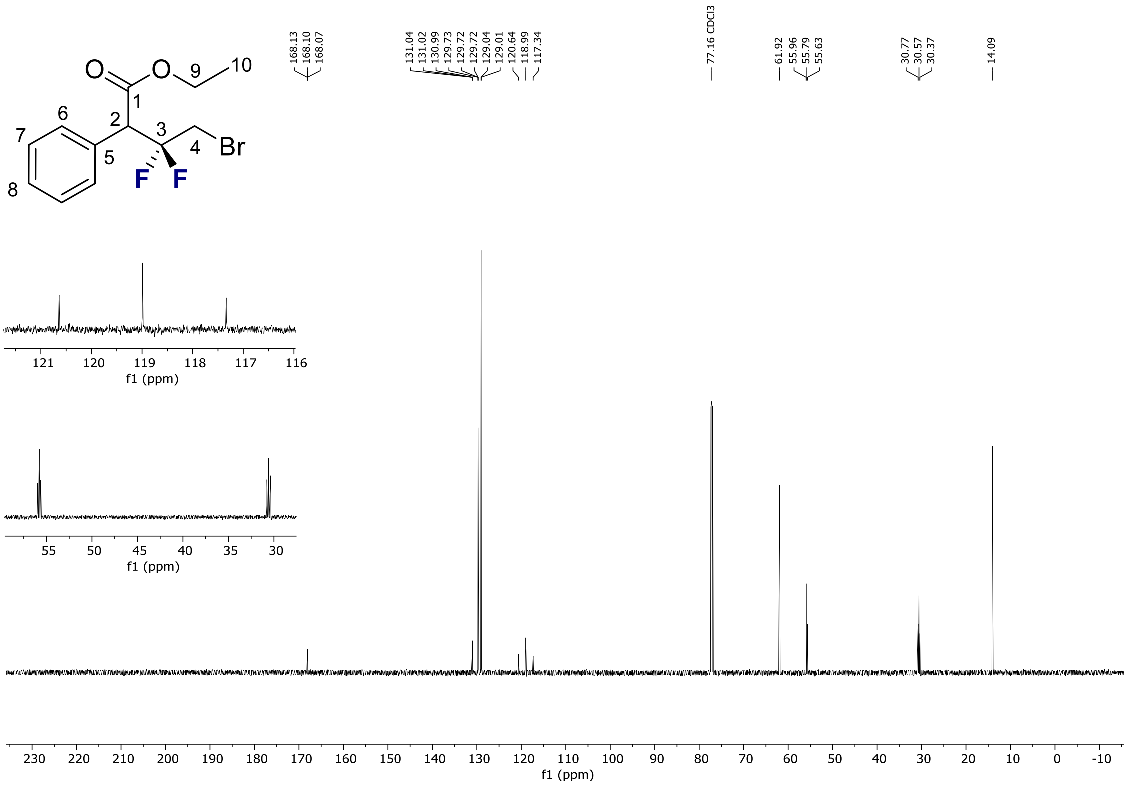

^{19}F NMR (376 MHz, $CDCl_3$):

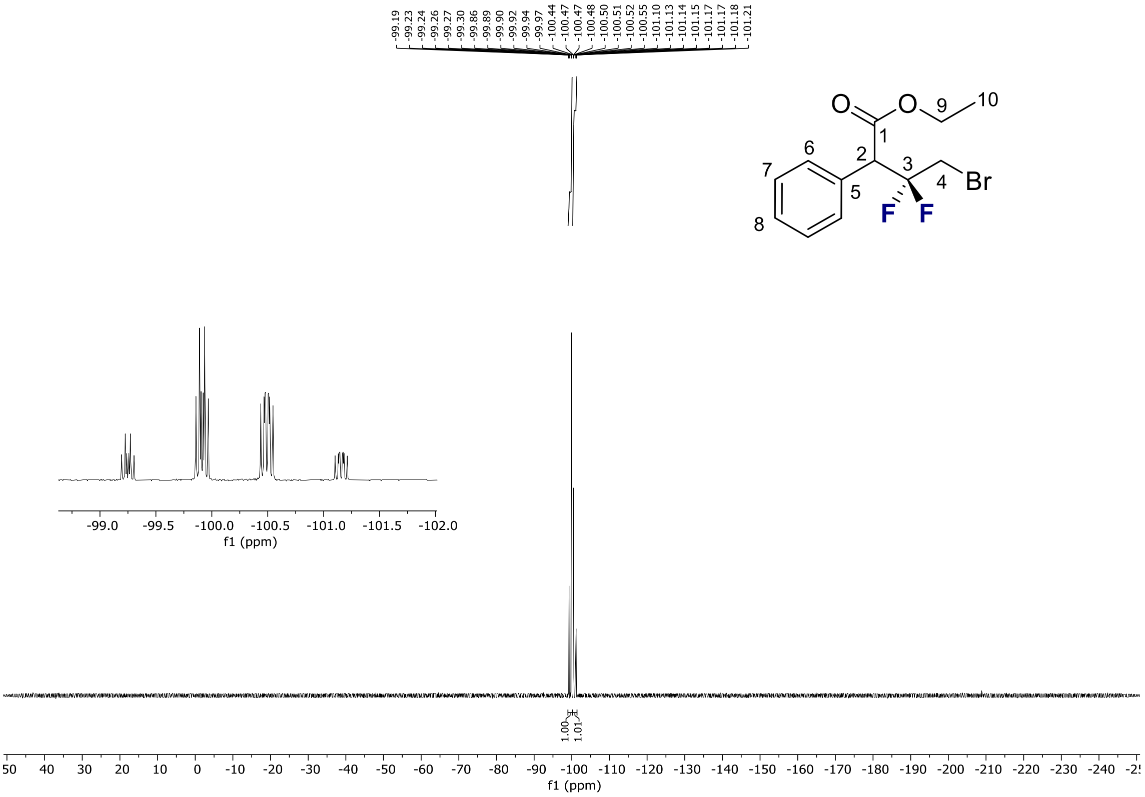

^{19}F{^{1}H} NMR (376 MHz, $CDCl_3$):

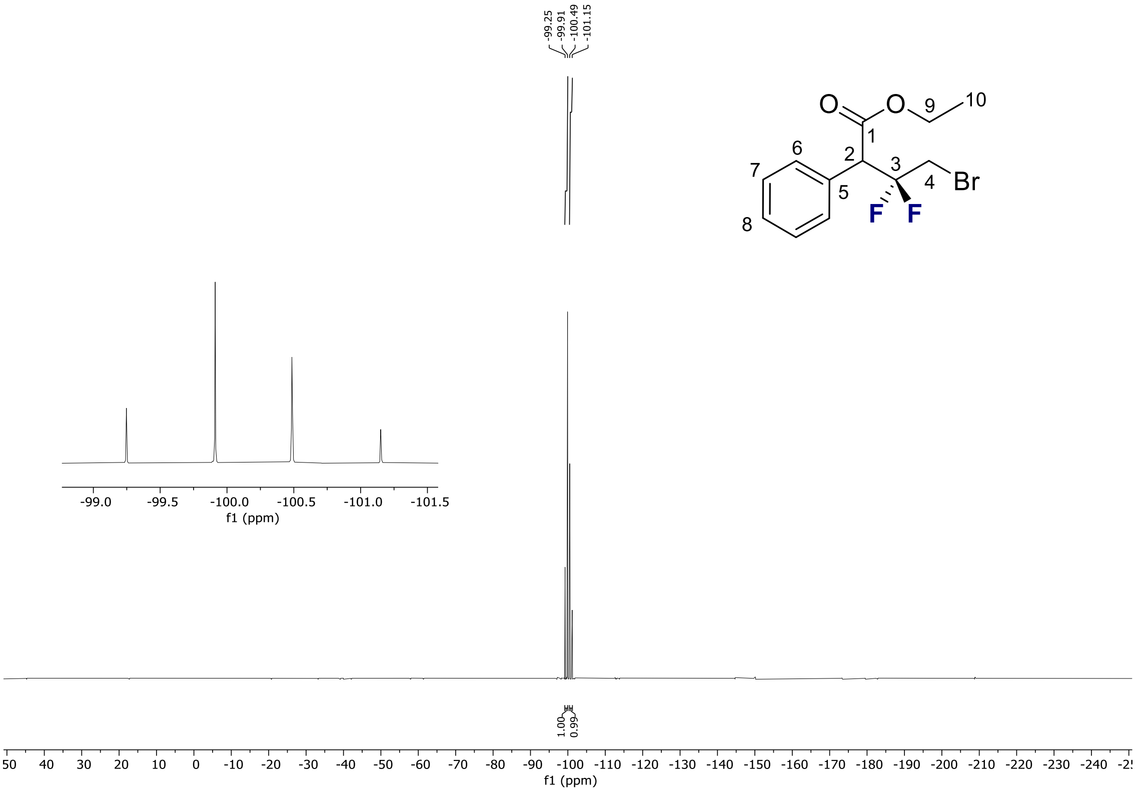

Ethyl 4-chloro-3,3-difluoro-2-phenylbutanoate (16)

^{1}H NMR (500 MHz, $CDCl_3$):

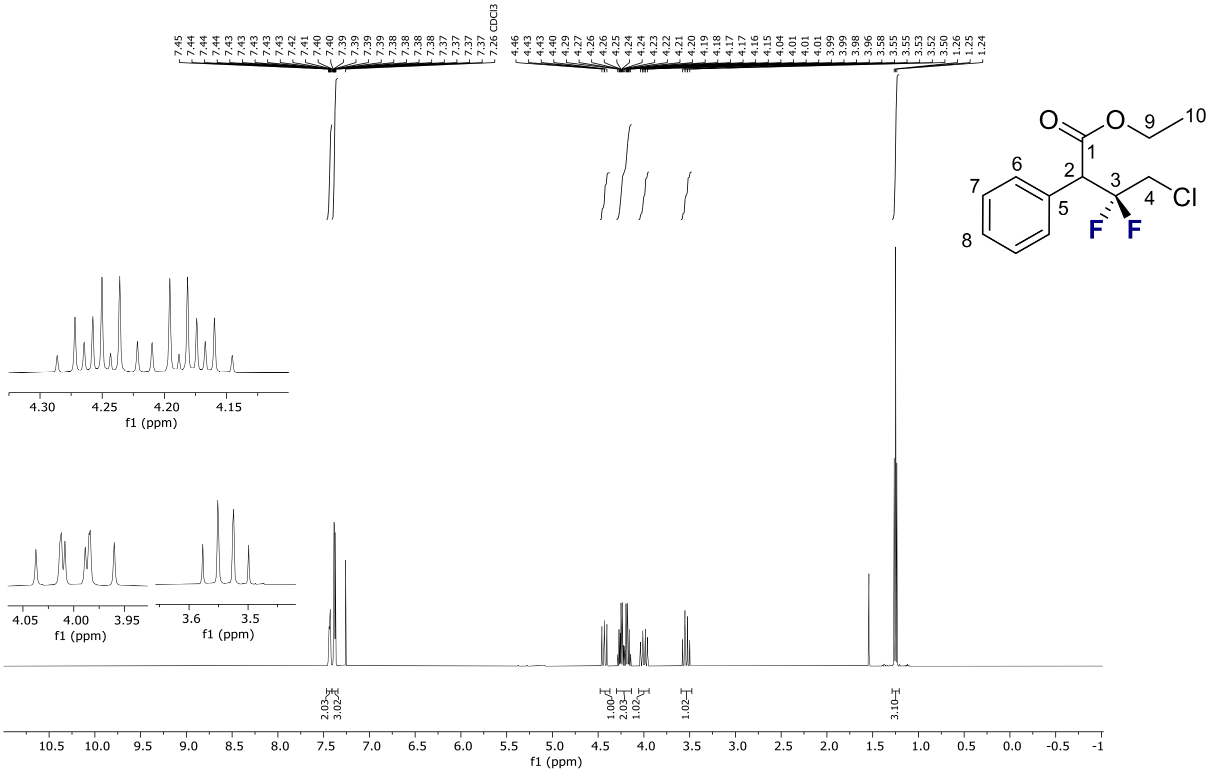

^{13}C NMR (126 MHz, $CDCl_3$):

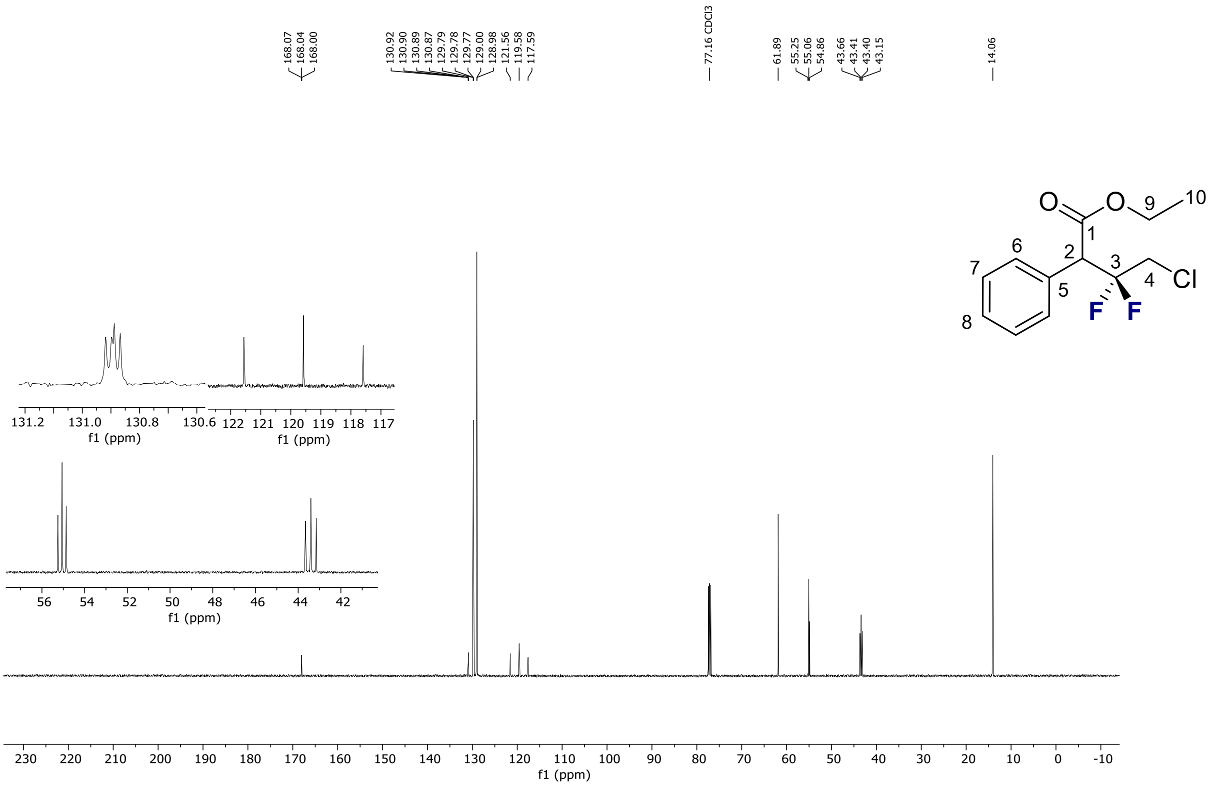

^{19}F NMR (376 MHz, $CDCl_3$):

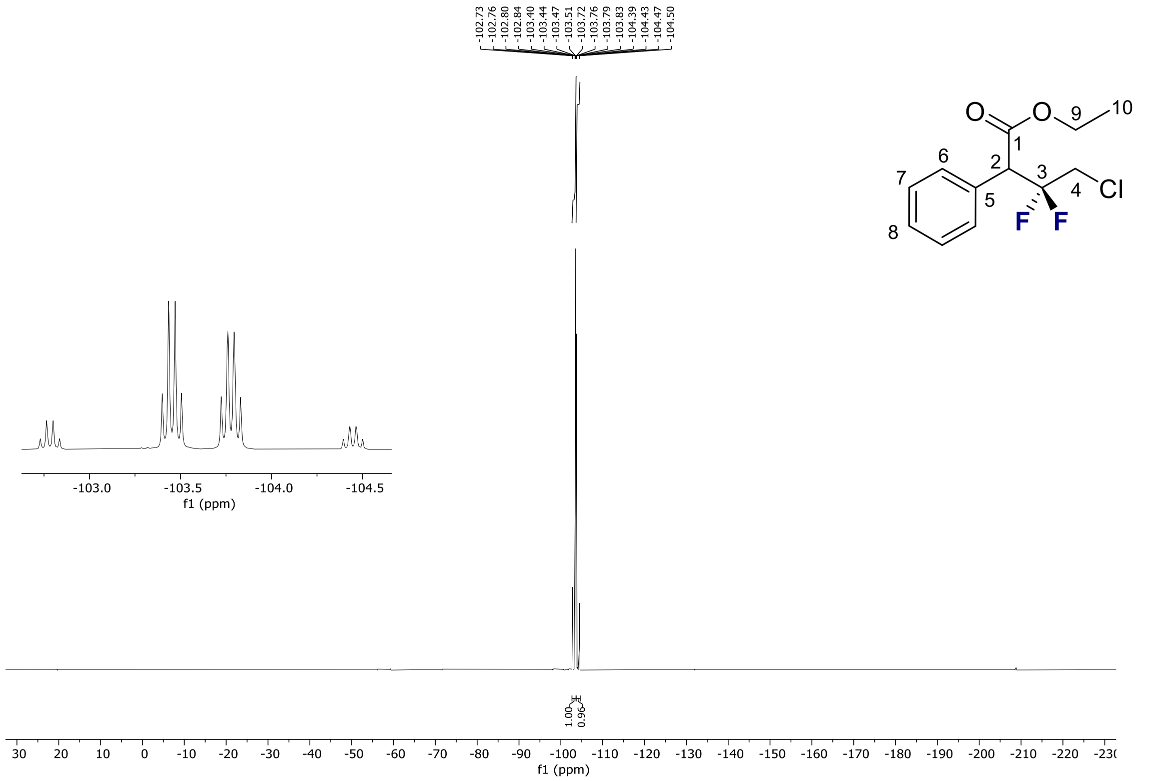

^{19}F{^{1}H} NMR (376 MHz, $CDCl_3$):

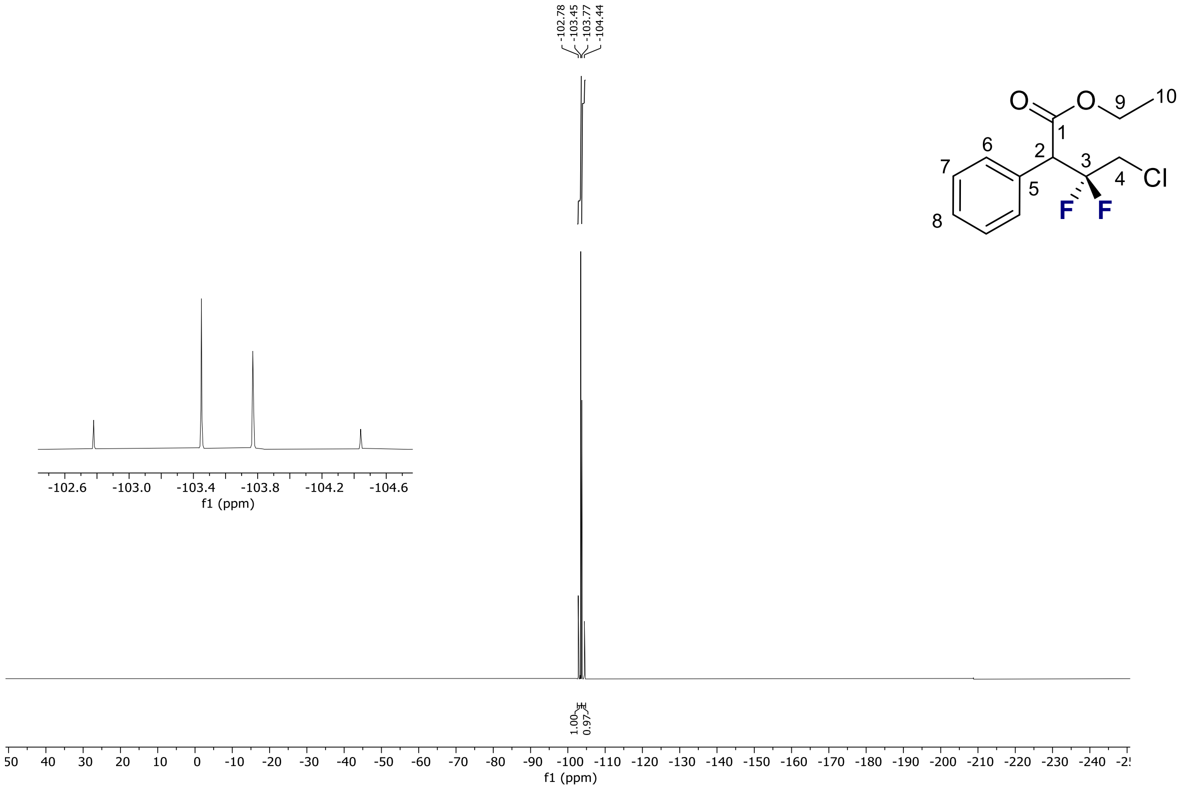

4-(3-(4-Bromophenyl)-2,2-difluoropropoxy)benzonitrile (17)

¹H NMR (500 MHz, $CDCl_3$):

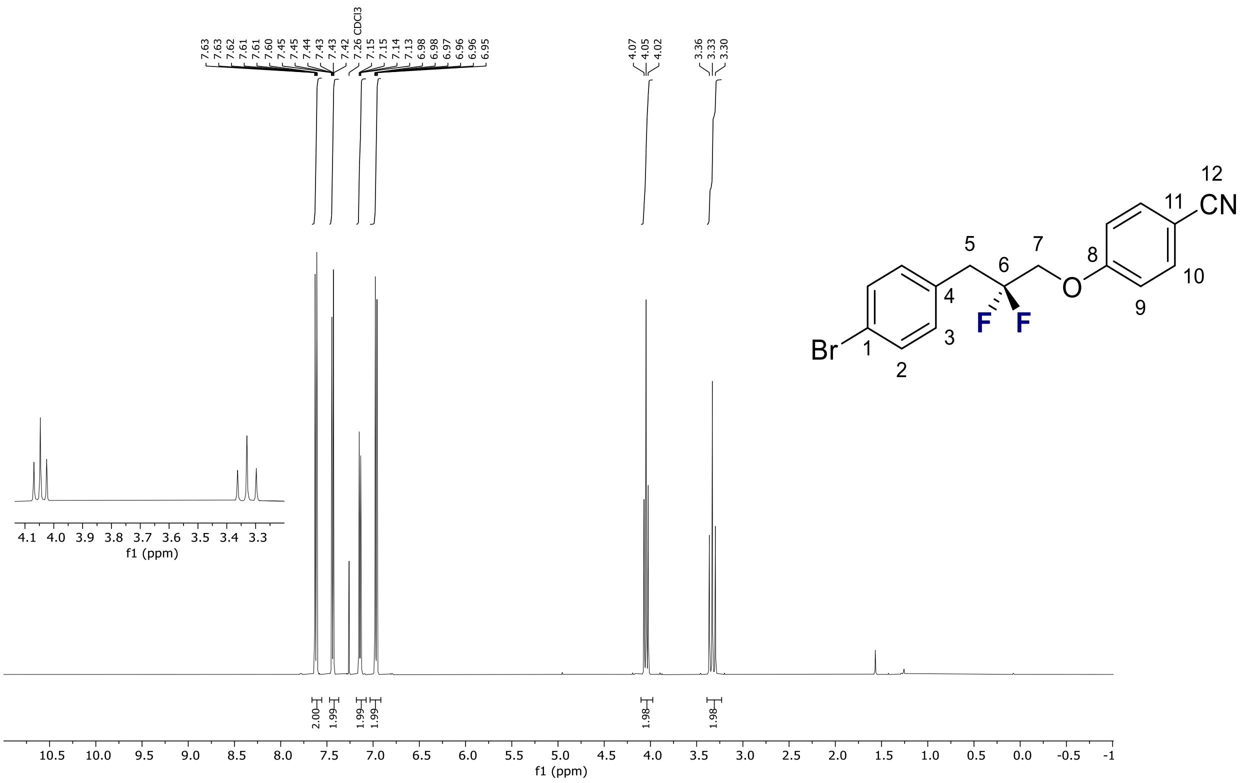

^{13}C{^{1}H} NMR (126 MHz, $CDCl_3$)

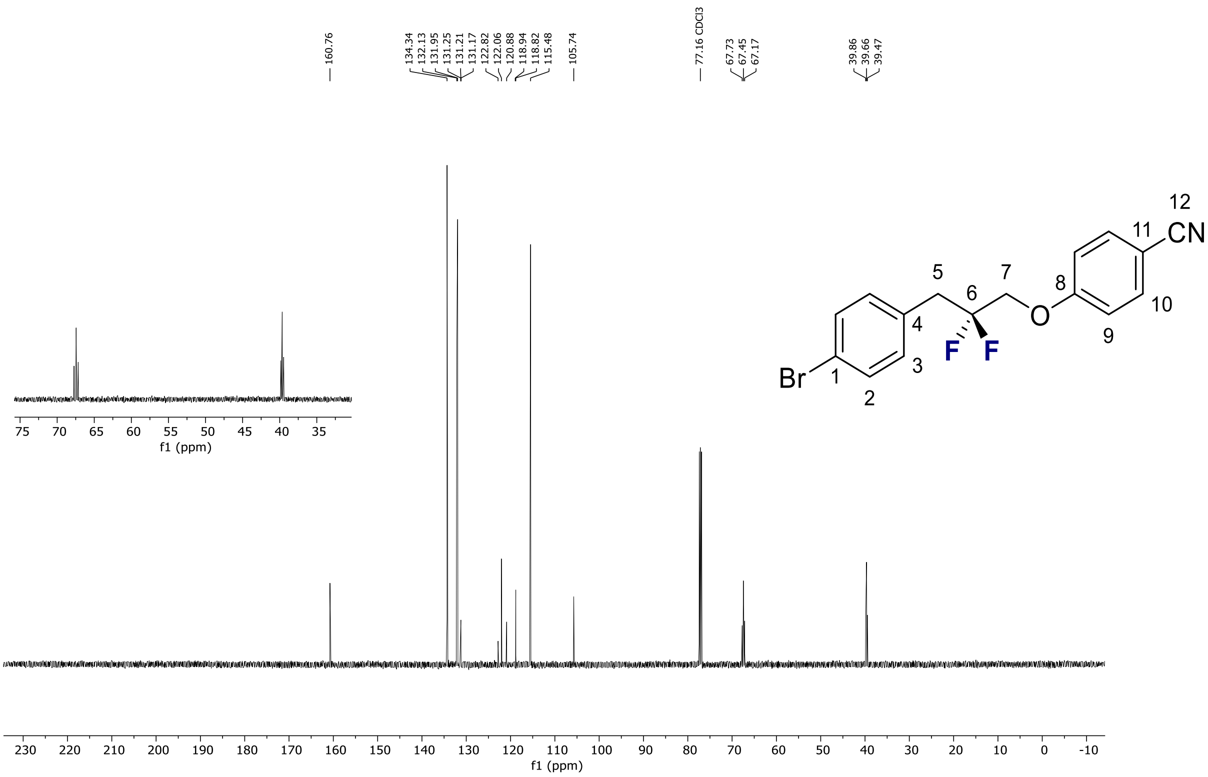

^{19}F NMR (470 MHz, $CDCl_3$):

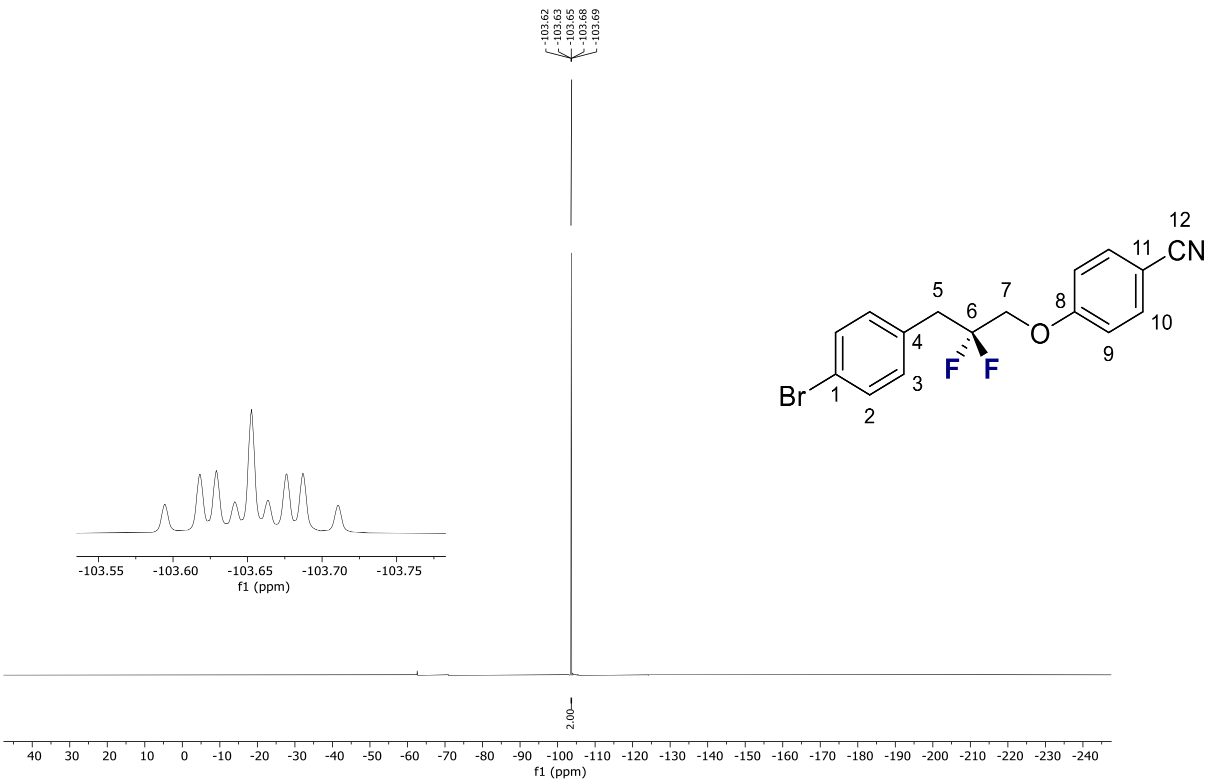

^{19}F{^{1}H} NMR (377 MHz, $CDCl_3$):

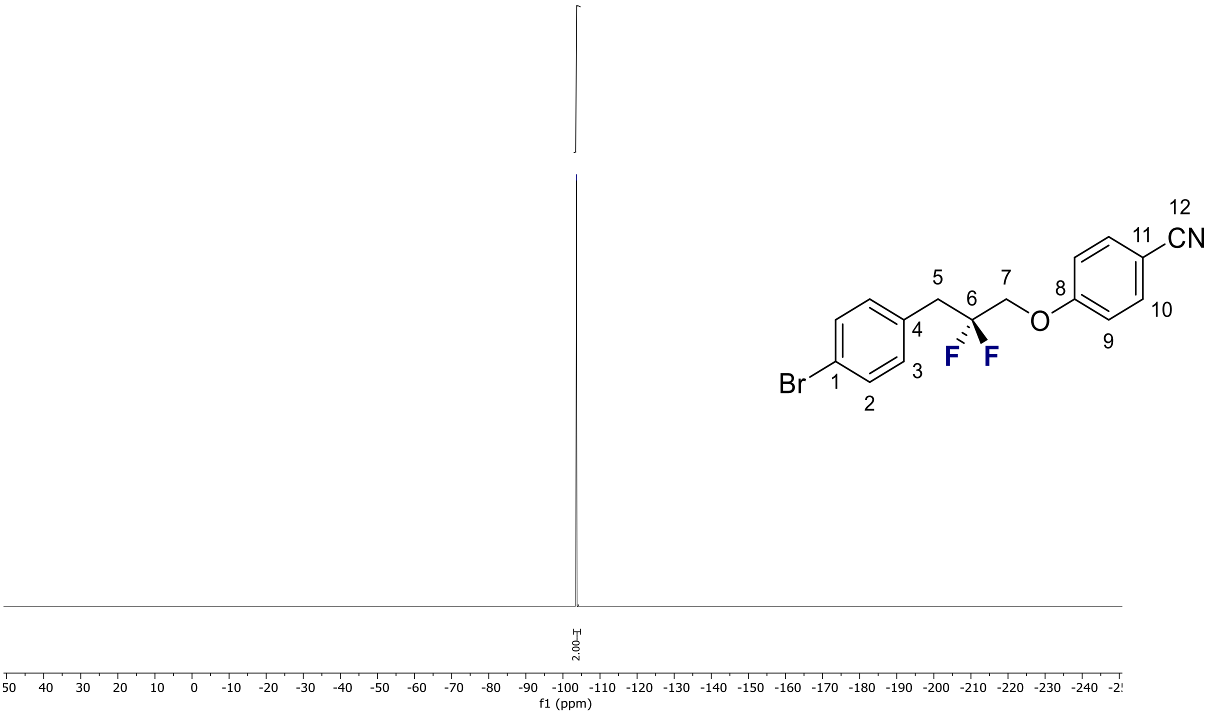

((4-(3-Bromo-2,2-difluoropropyl)phenyl)ethynyl)trimethylsilane (18)

^{1}H NMR (599 MHz, $CDCl_3$):

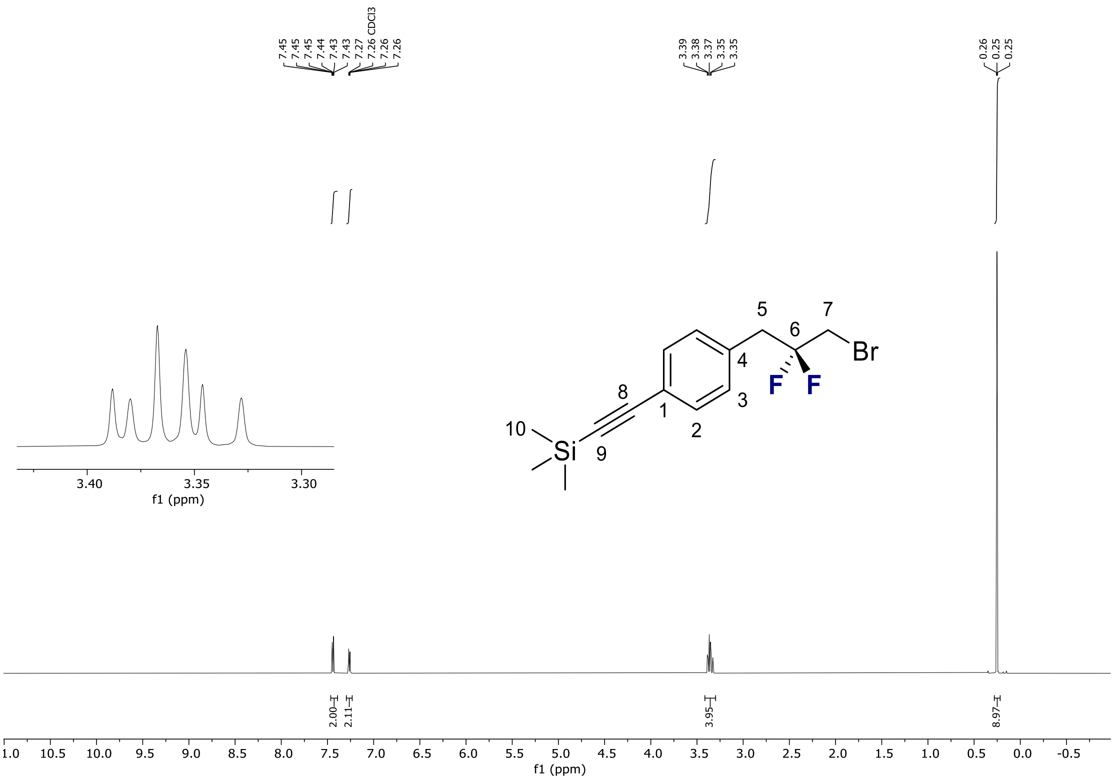

^{13}C{^{1}H} NMR (151 MHz, $CDCl_3$):

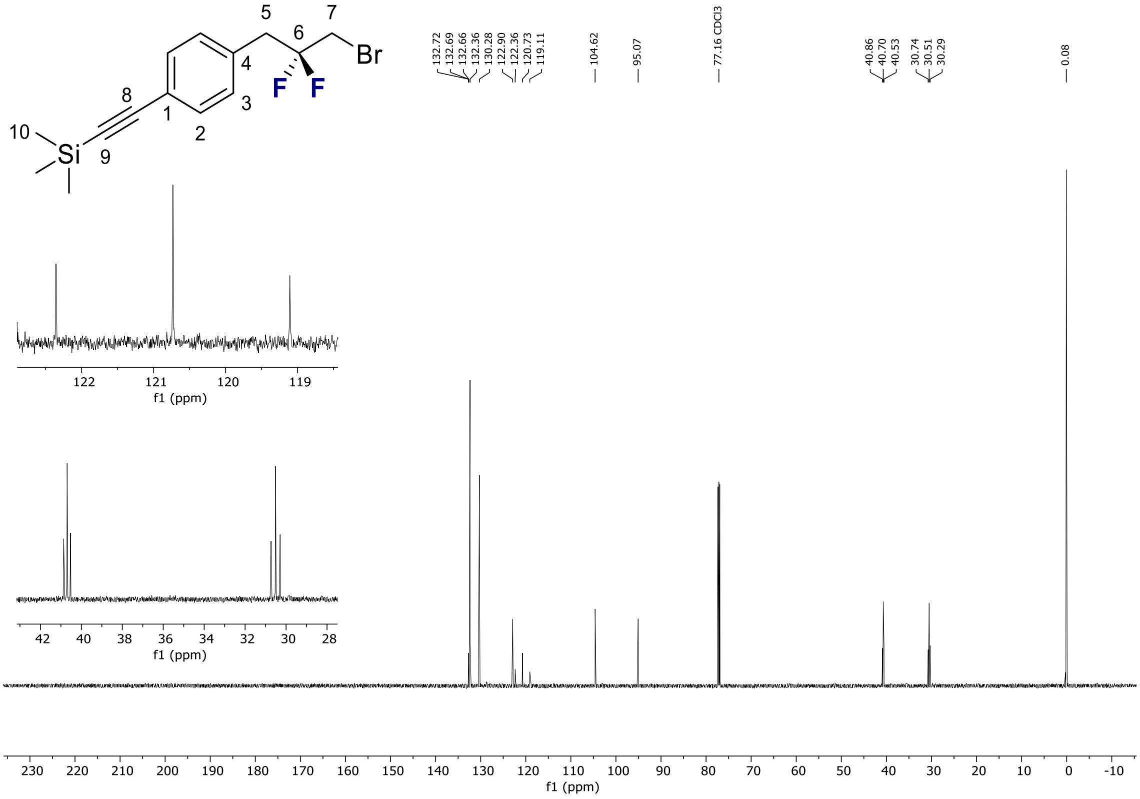

^{19}F NMR (564 MHz, $CDCl_3$):

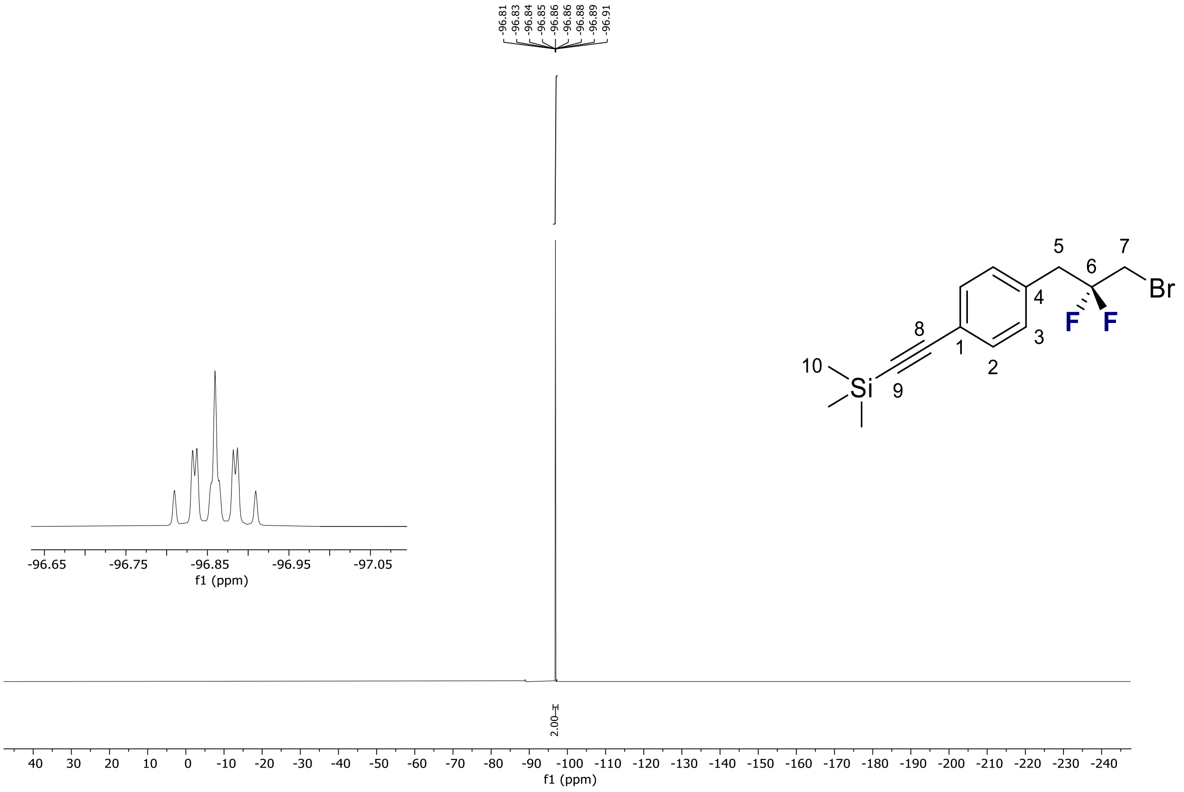

^{19}F{^{1}H} NMR (564 MHz, $CDCl_3$):

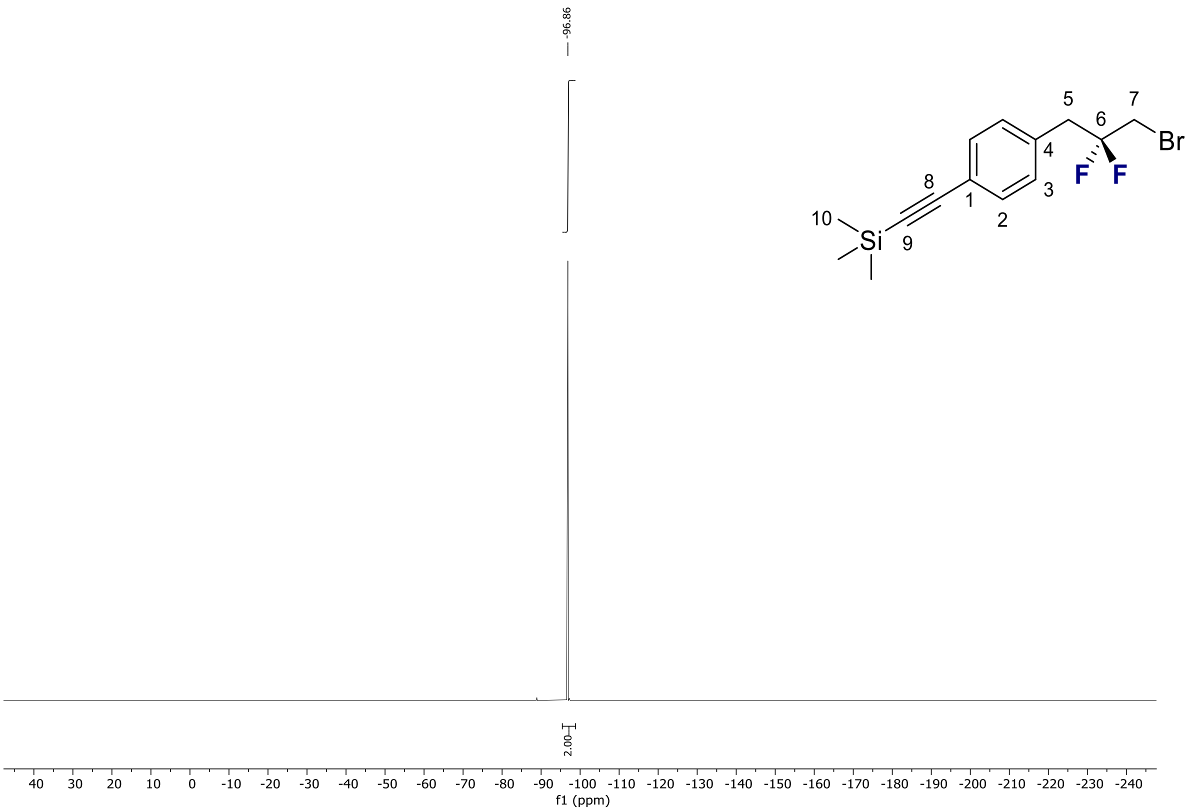

2,2-Difluoro-3-(4-(trifluoromethyl)phenyl)propan-1-aminium chloride (20)

^{1}H NMR (599 MHz, DMSO-d6):

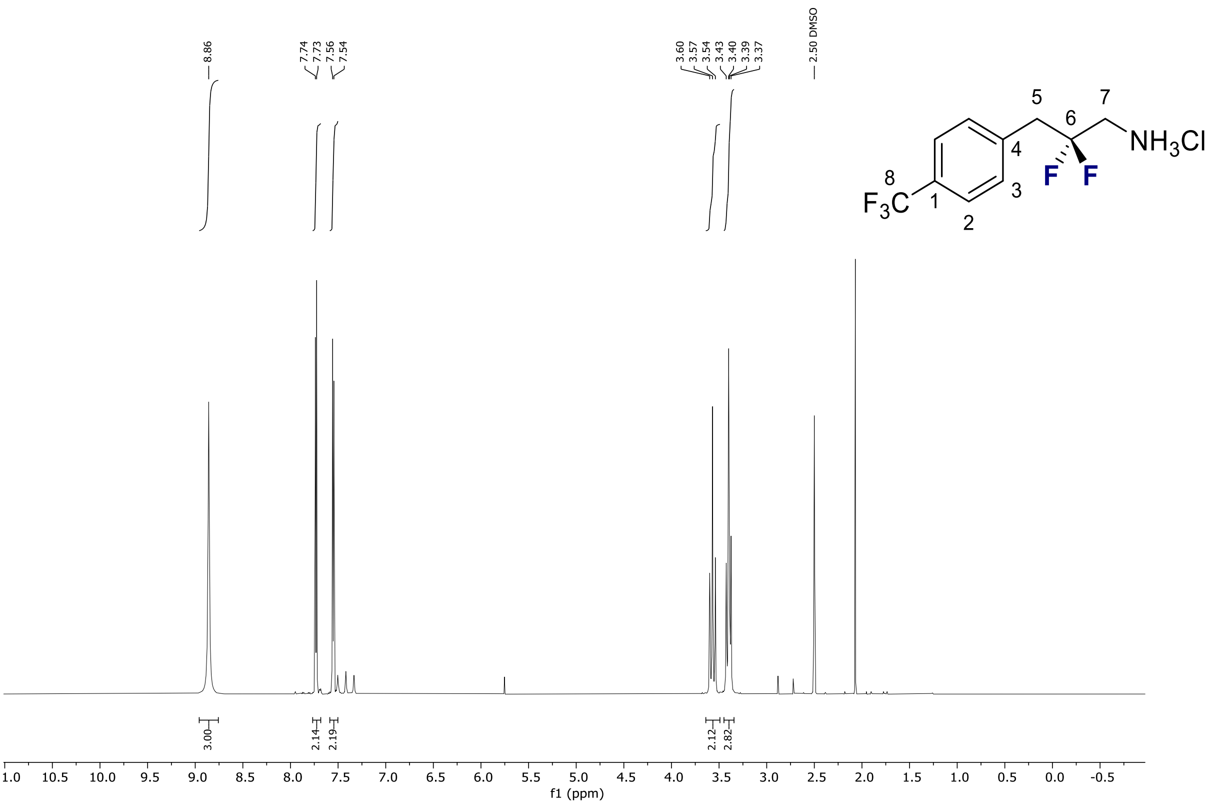

13C{1H} NMR (151 MHz, DMSO-d6):

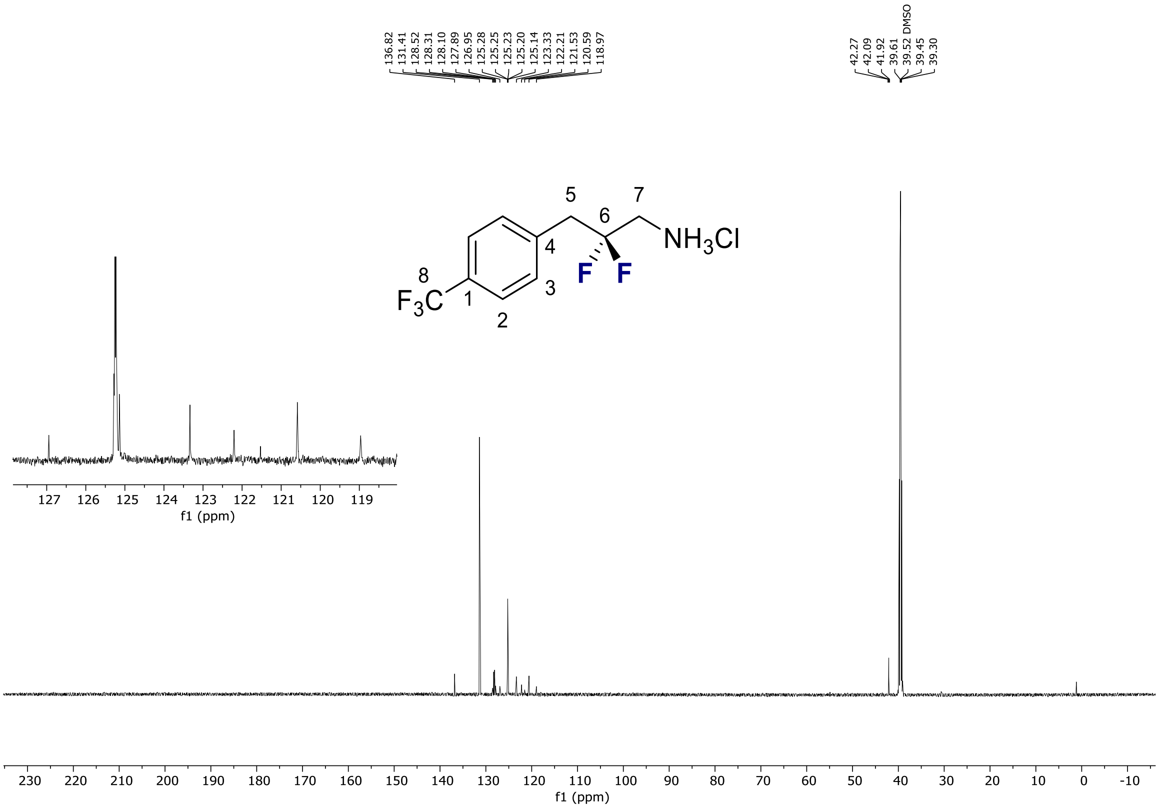

^{19}F NMR (564 MHz, DMSO-d6):

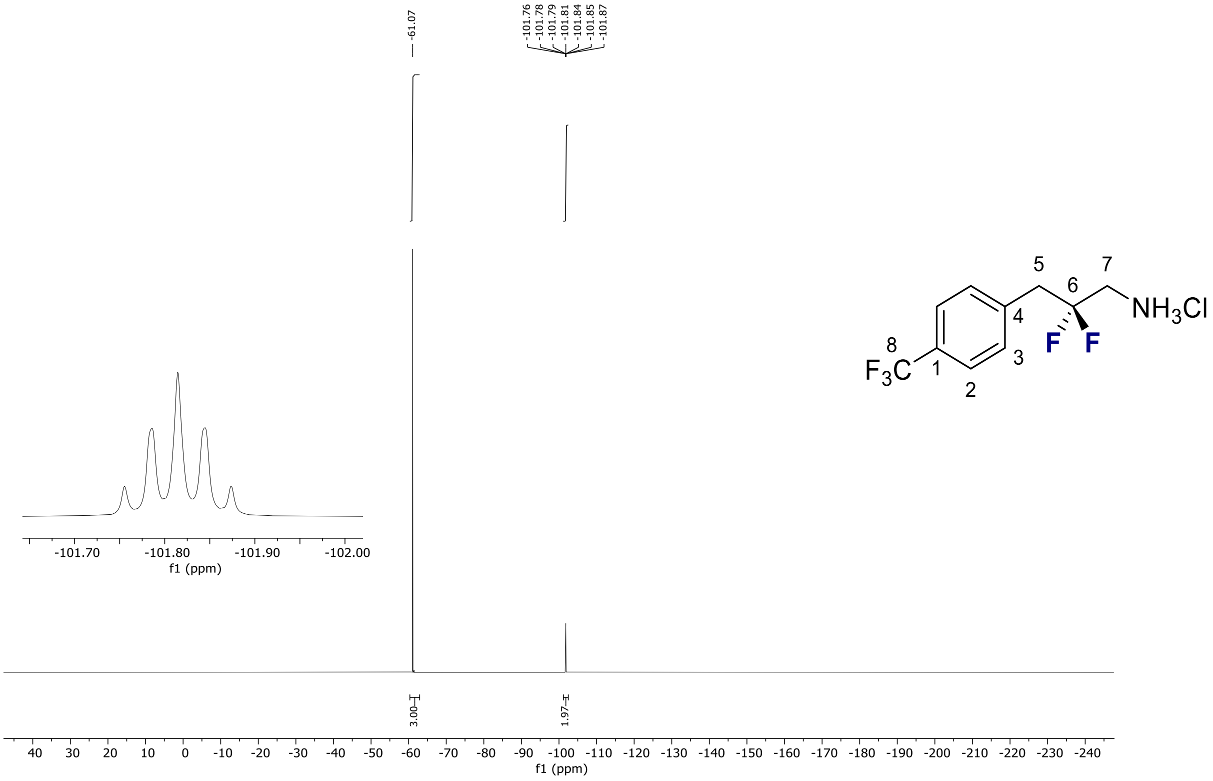

^{19}F{^{1}H} NMR (564 MHz, DMSO-d6):

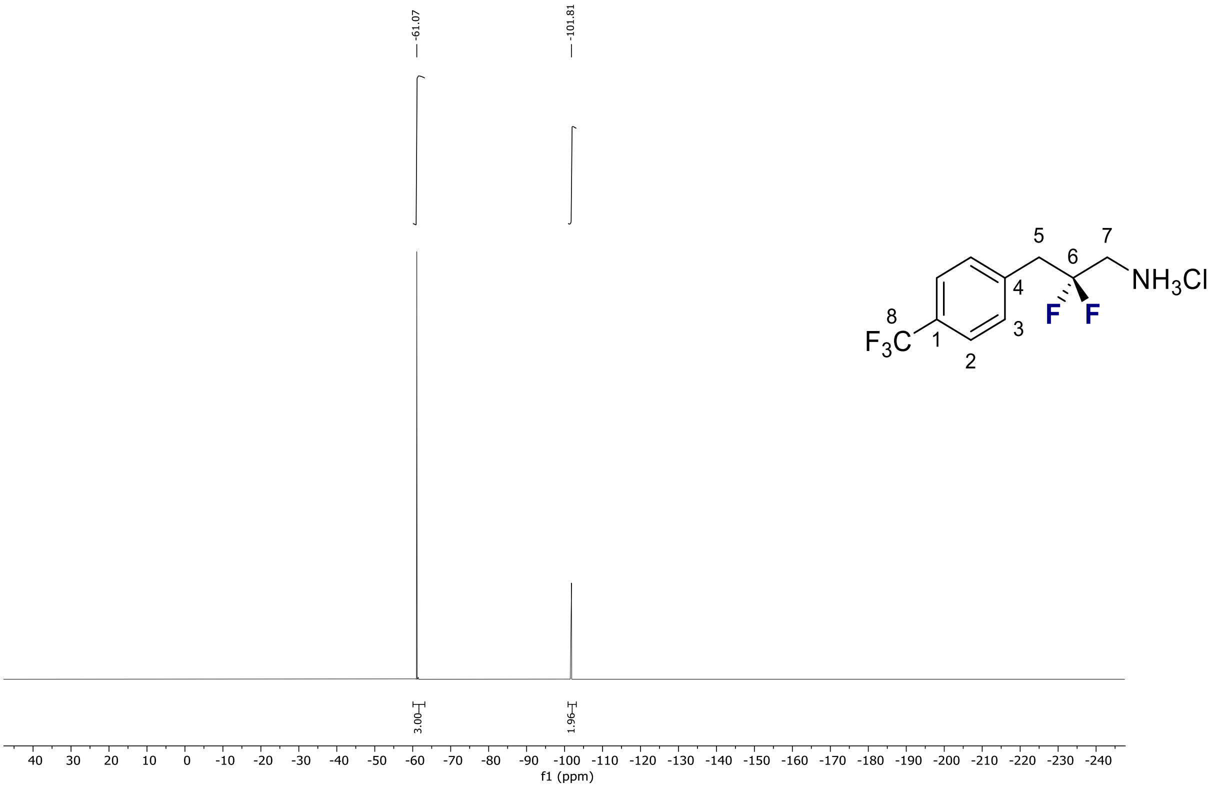

1,3-*Bis*(2,2-difluoro-3-(4-(trifluoromethyl)phenyl)propyl)urea (20-2)

^{1}H NMR (599 MHz, DMSO-d6):

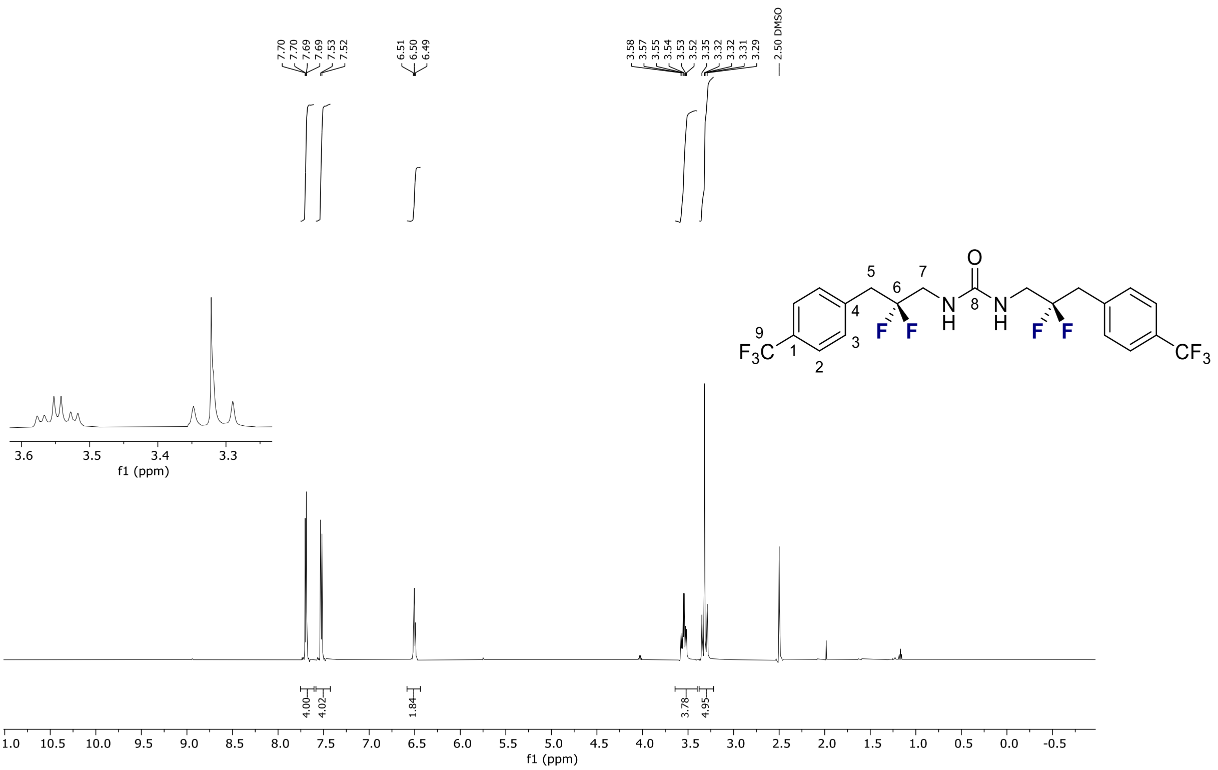

^{13}C NMR (151 MHz, DMSO-d6):

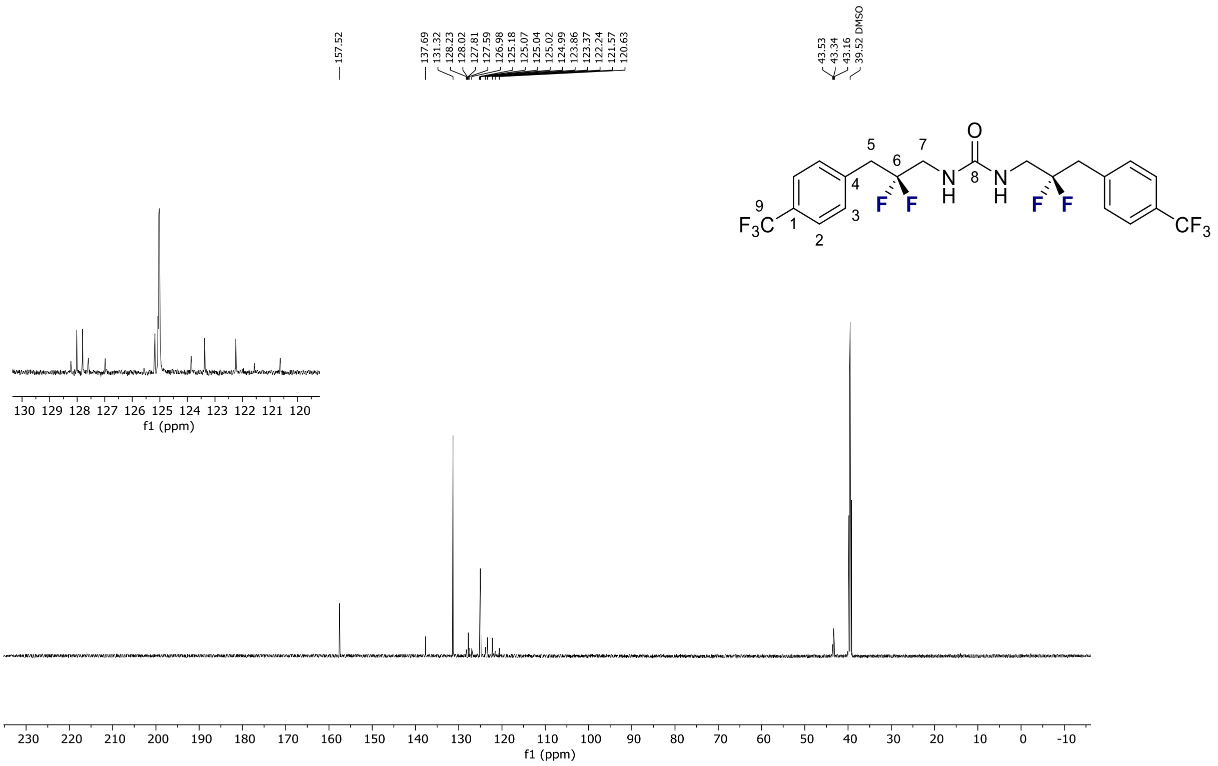

^{19}F NMR (564 MHz, DMSO-*d*6)

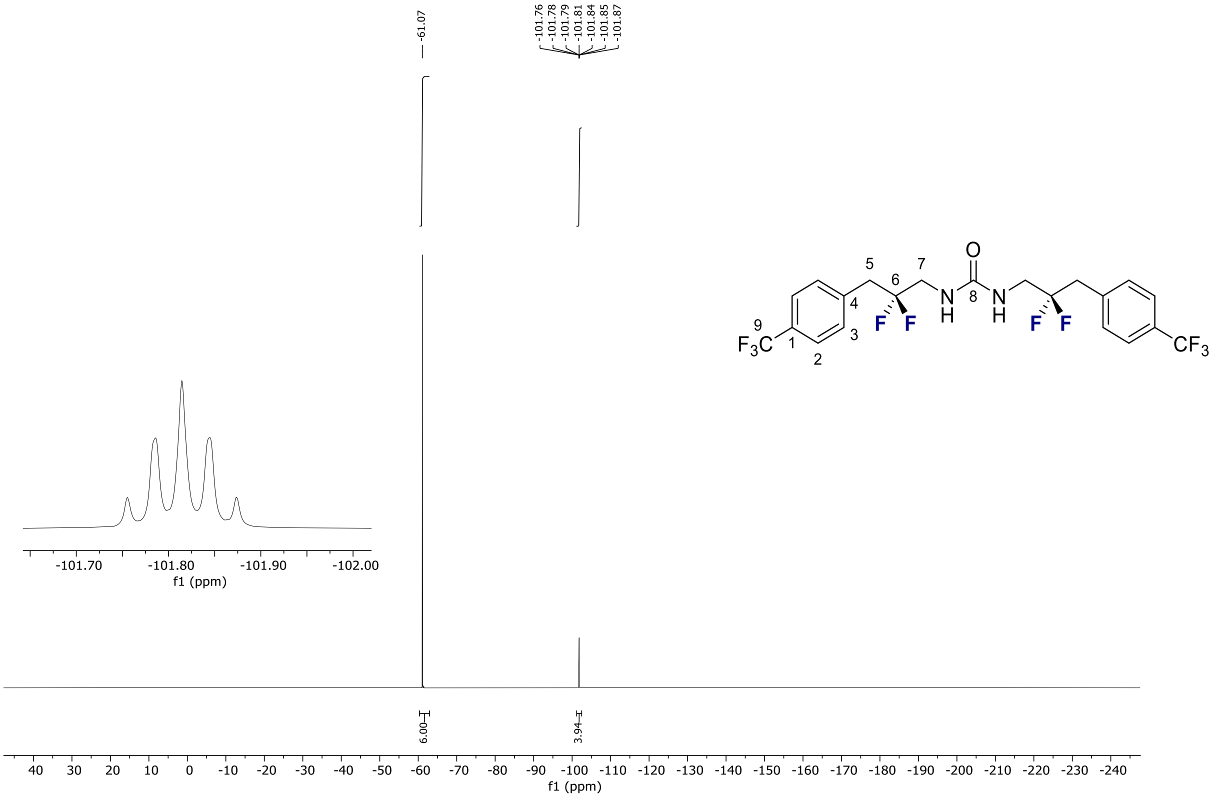

^{19}F{^{1}H} NMR (377 MHz, DMSO-*d*6)

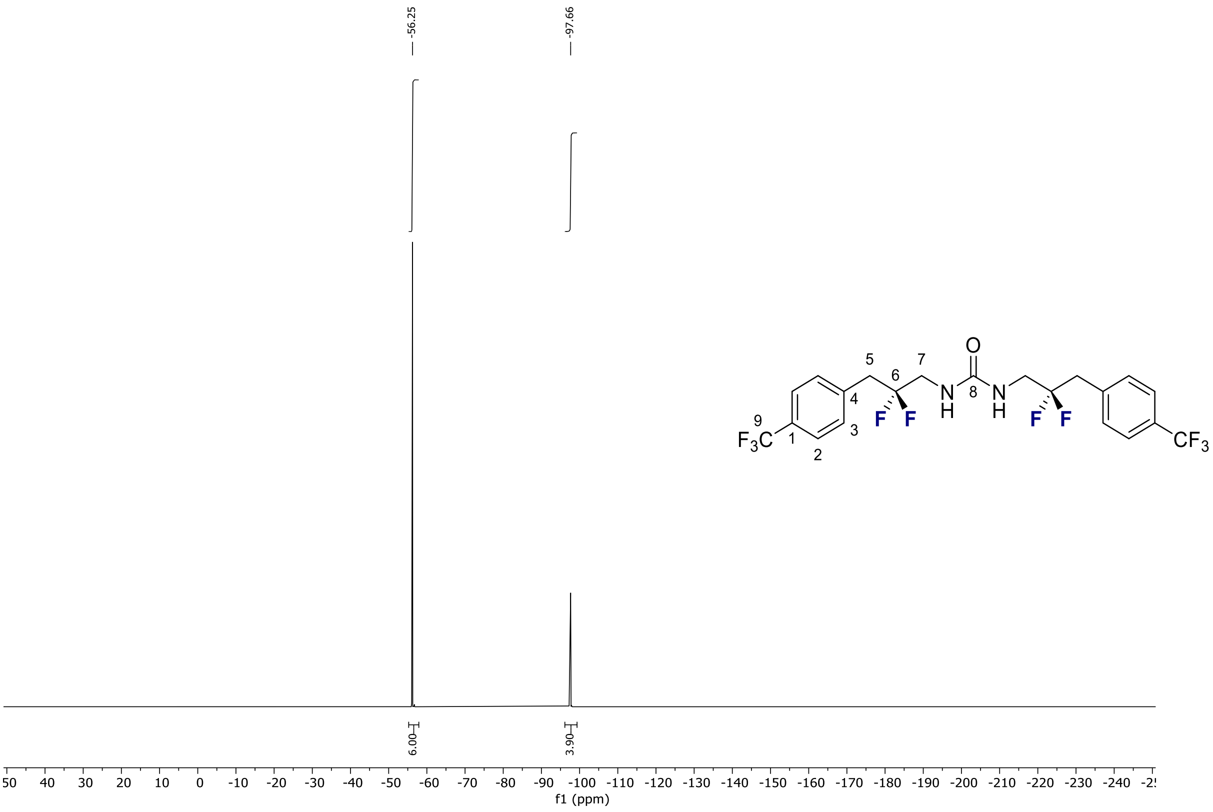

1,3-*Bis*(2,2-difluoro-3-(4-(trifluoromethyl)phenyl)propyl)pyrimidine-2,4,6(1*H*,3*H*,5*H*)-trione (21)

^{1}H NMR (599 MHz, $CDCl_3$):

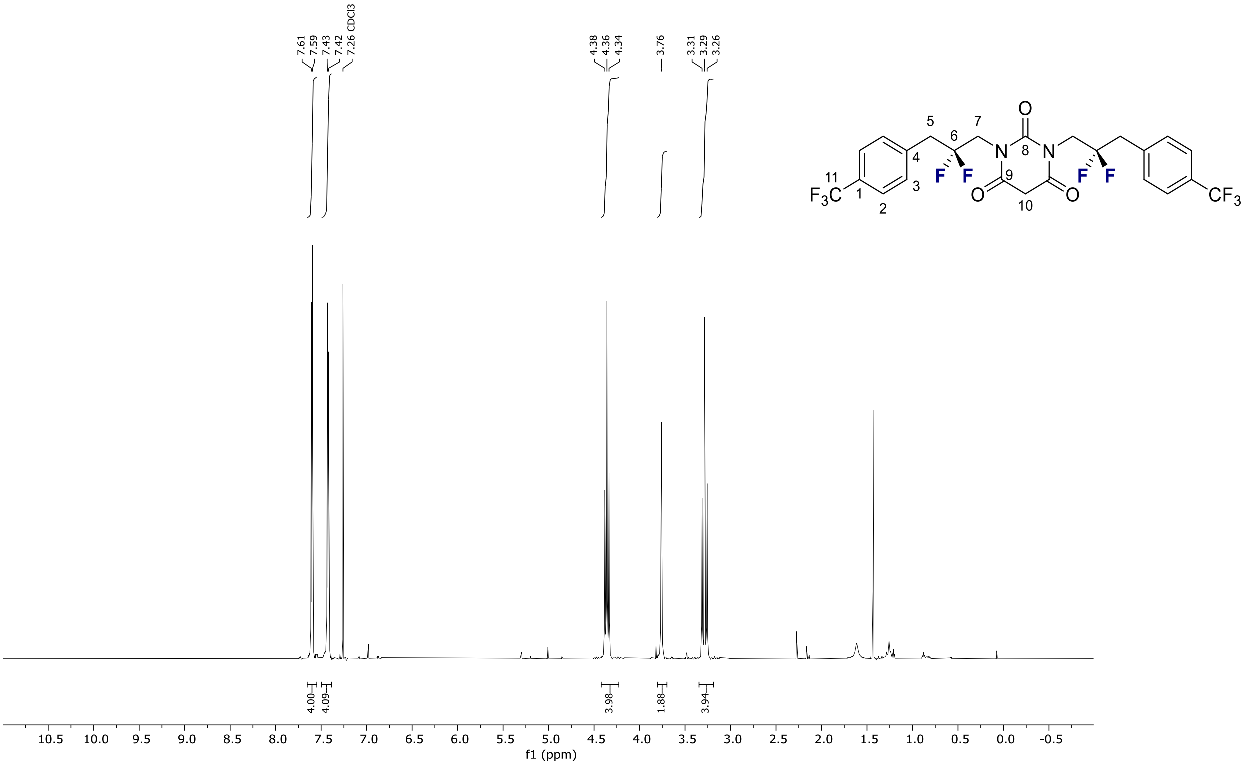

^{13}C{^{1}H} NMR (151 MHz, $CDCl_3$):

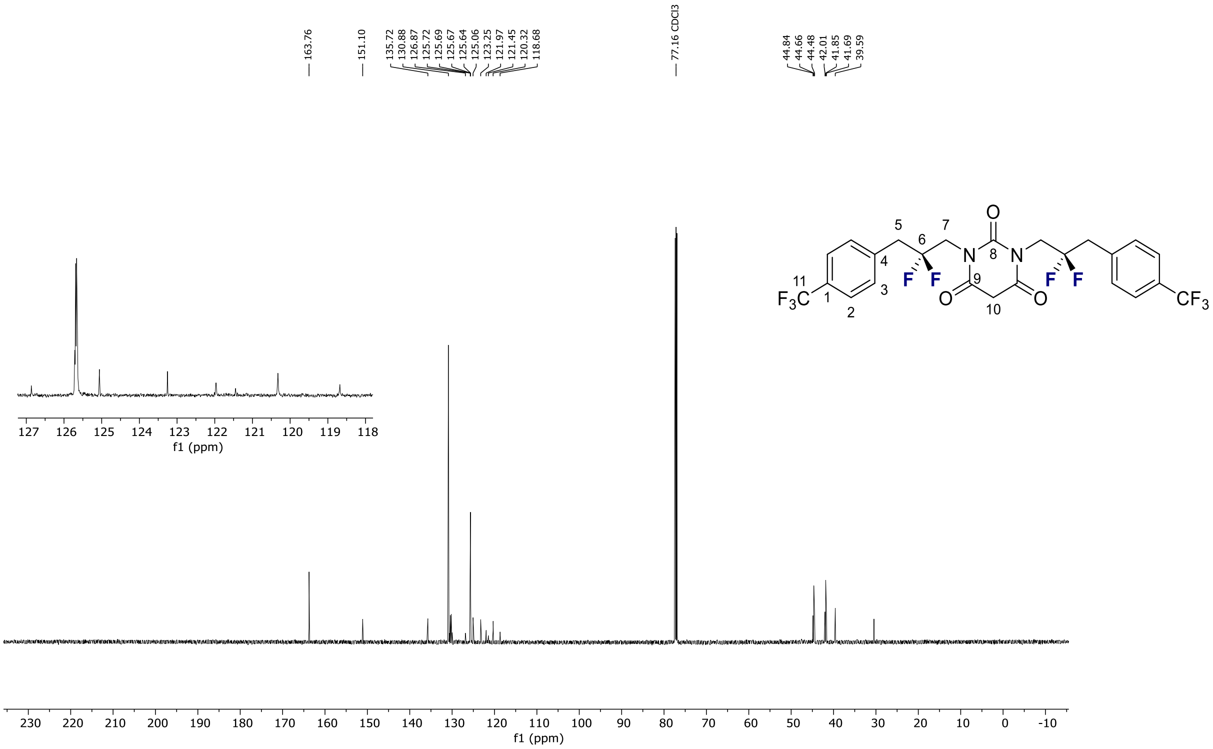

^{19}F NMR (377 MHz, $CDCl_3$):

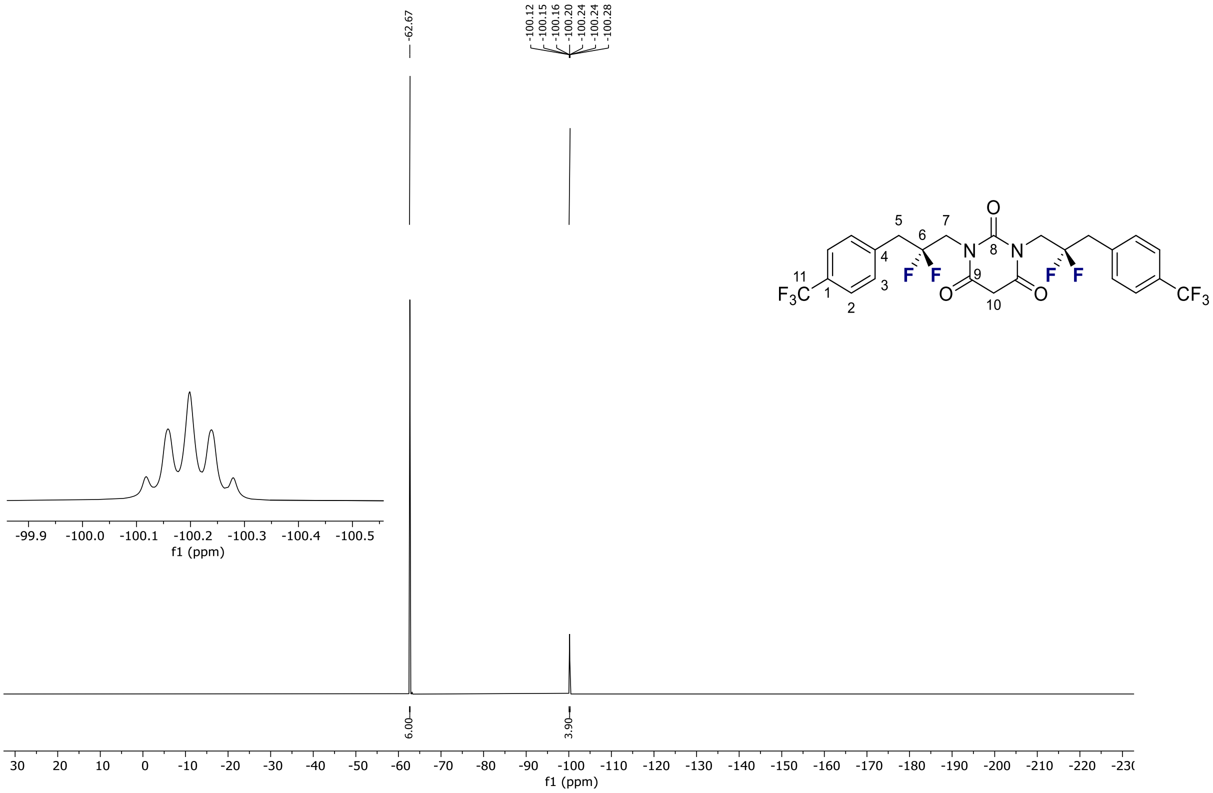

^{19}F{^{1}H} NMR (377 MHz, $CDCl_3$):

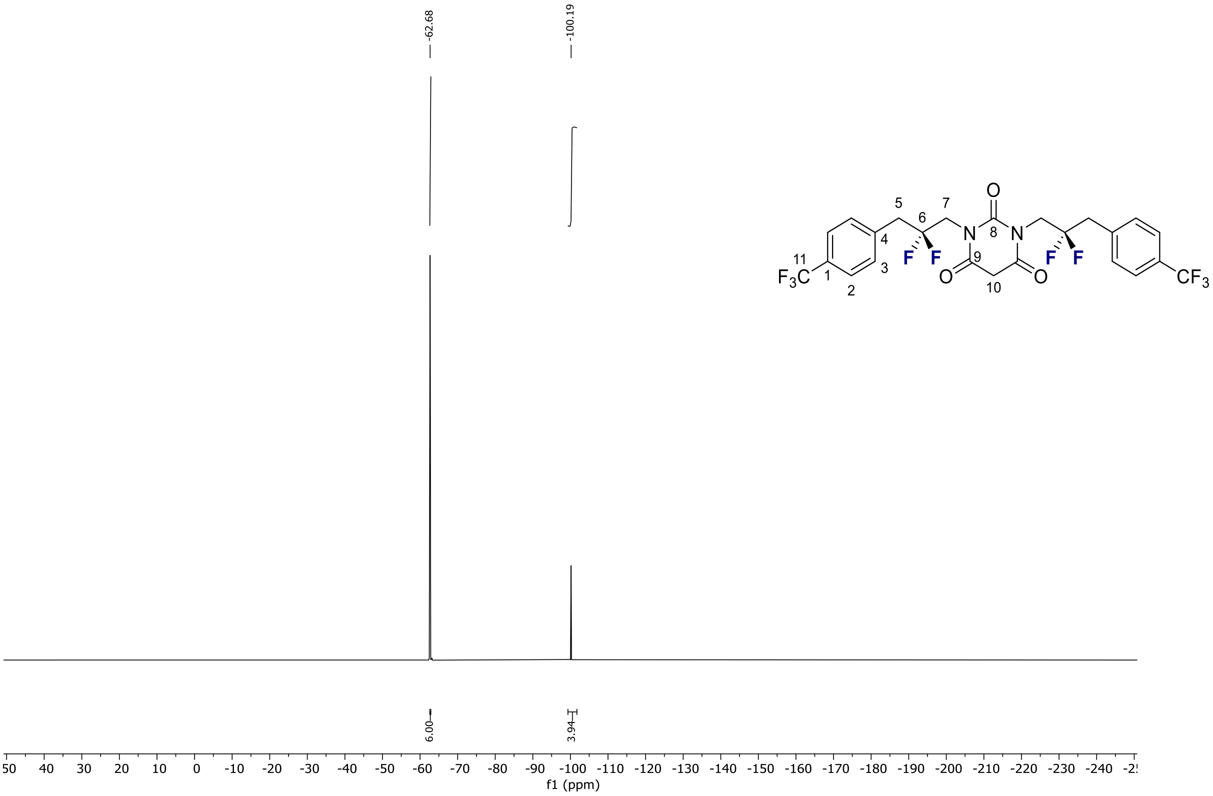

6. References

[1] M. A. Berliner; E. M. Cordi, J. R. Dunetz, K. E. Price, *Org. Process Res. Dev.* 2010, **14**, 180.

[2] A. Hasuoka, Y. Nishikimi, Y. Nakayama, K. Kamiyama, M. Nakao, K. Miyagawa, O. Nishimura, M. Fujino, *J. Antibiot.* 2002, **55(3)**, 322.

[3] J. C. Sarie, C. Thiehoff, J. Neufeld, C. G. Daniliuc, R. Gilmour, *Angew. Chem. Int. Ed.* 2020, **59(1)**, 15069.

[4] F. Scheidt, M. Schäfer, J. C. Sarie, C. G. Daniliuc, J. J. Molloy, R. Gilmour, *Angew. Chem. Int. Ed.* 2018, **57(1)**, 16431.

[5] S. Meyer, J. Häfliger, M. Schäfer, J. J. Molloy, C. G. Daniliuc, R. Gilmour, *Angew. Chem. Int. Ed.* 2021, DOI: doi.org/10.1002/anie.202015946

[6] X. Lei, A. Jalla, M. A. Abou Shama, J. M. Stafford, B. Cao, *Synthesis* 2015, **47(17)**, 2578.

[7] J. Duczynski, A. N. Sobolev, S. A. Moggach, R. Dorta S. G. Stewart, *Organometallics* 2020, **39(1)**, 105.

[8] A. L. Smith, P. E. Brennan, F. F. Demorin, G. Liu, N. A. Paras, D. M. Retz, D. M. (AMGEN INC.), WO pat., 2006066172A1, 2006.

[9] Davidson, A. H.; Moffat D. F. C.; Day, F. A.; Donald A. D. G. (CHROMA THERAPEUTICS LTD), WO pat., 2008040934A1, 2008.

[10] F. Hochberger-Roa, S.-C. Mendoza, D. G. Rosas, R. A. Toscano, M. C. Ortega-Alfaro, J.-G. López-Cortés, *Adv. Synth. Catal.* 2019, **361**, 4055.

[11] G. Pratsch, L. E. Overman, *J. Org. Chem.* 2015, **80**, 11388.

[12] F. Scheidt, J. Neufeld, M. Schäfer, C. Thiehoff, R. Gilmour, *Org. Lett.* 2018, **20(24)**, 8073.

[13] J. Huang, G. Hu, S. An, D. Chen, M. Li, P. Li, *J. Org. Chem.* 2019, **84**, 9758.

[14] R. J. Maza, J. Royes, J. J. Carbó, E. Fernández, *Chem. Commun.* 2020, **56**, 5973.

[15] E. M. Driggers, H. S. Cho, C. W. Liu, C. P. Katzka, A. C. Braisted, H. D. Ulrich, D. E. Wemmer, P. G. Schultz, *J. Am. Chem. Soc.* 1998, **120(9)**, 1945.

[16] J. M. Wiest, M. L. Conner, M. K. Brown, *J. Am. Chem. Soc.* 2018, **140(46)**, 15943.

[17] A. Boelke, L. D. Caspers, B. J. Nachtsheim, *Org. Lett.* 2017, **19(19)**, 5344.

[18] J. Amitabh, T. Y. Chou, Z. Al Jaroudi, B. D. Ellis, T. S. Cameron, *Beilstein J. Org. Chem.* 2014, **10**, 848.

[19] T. Fedynyshyn, K. Roderick, M. Sworin, R. Sinta, (MASSACHUSETTS INSTITUTE OF TECHNOLOGY); WO pat. 02/069043A2, 2002.

[20] X. Wang, Z. Wang, Y. Asanuma, Y. Nishihara, *Org. Lett.* 2019, **21(10)**, 3640.

[21] J. B. Metternich, R. Gilmour, *J. Am. Chem. Soc.* 2015, **137(35)**, 11254.

[22] Q.-J. Liang, C. Yang, F.-F. Meng, B. Jiang, Y.-H. Xu, T.-P. Loh, *Angew. Chem. Int. Ed.* 2017, **56**, 5091.

[23] T. Ohmura, K. Masuda, I. Takase, M. Suginome, *J. Am. Chem. Soc.* 2009, **131(46)**, 16624.

[24] M. M. Amer, O. Olaizola, J. Carter, H. Abas, J. Clayden, *Org. Lett.* 2020, **22(1)**, 253.

[25] M. M. Hansmann, R. L. Melen, M. Rudolph, F. Rominger, H. Wadepohl, D. W. Stephan, A. S. K. Hashmi, *J. Am. Chem. Soc.* 2015, **137(49)**, 15469.

[26] M. D. Levin, F. D. Toste, *Angew. Chem. Int. Ed.* 2014, **53(24)**, 6211.

[27]: K. Heckenbichler, A. Schweiger, L. A. Brandner, A. Binter, M. Toplak, P. Macheroux, K. Gruber, R. Breinbauer, *Angew. Chem. Int. Ed.* 2018, **57(7)**, 7240.

[28] H. Tsuji, K. Yamagata, Y. Itoh, K. Endo, M. Nakamura, E. Nakamura, *Angew. Chem. Int. Ed.* 2007, **46(42)**, 8060.

[29] G. Xia, R. Benmohamed, R. I. Morimoto, D. R. Kirsch, R. B. Silverman, *Bioorg. Med. Chem. Lett.* 2014, **24**, 5098.
